# Unusual Ratio between Free Thyroxine and Free Triiodothyronine in a Long-Lived Mole-Rat Species with Bimodal Ageing

**DOI:** 10.1371/journal.pone.0113698

**Published:** 2014-11-19

**Authors:** Yoshiyuki Henning, Christiane Vole, Sabine Begall, Martin Bens, Martina Broecker-Preuss, Arne Sahm, Karol Szafranski, Hynek Burda, Philip Dammann

**Affiliations:** 1 Department of General Zoology, Faculty of Biology, University of Duisburg-Essen, Essen, Germany; 2 Genome Analysis, Leibniz Institute for Age Research - Fritz Lipmann Institute, Jena, Germany; 3 Department of Endocrinology and Metabolism and Division of Laboratory Research, University Hospital, University of Duisburg-Essen, Essen, Germany; 4 Central Animal Laboratory, University Hospital, University of Duisburg-Essen, Essen, Germany; Rutgers University, United States of America

## Abstract

Ansell's mole-rats (*Fukomys anselli*) are subterranean, long-lived rodents, which live in eusocial families, where the maximum lifespan of breeders is twice as long as that of non-breeders. Their metabolic rate is significantly lower than expected based on allometry, and their retinae show a high density of S-cone opsins. Both features may indicate naturally low thyroid hormone levels. In the present study, we sequenced several major components of the thyroid hormone pathways and analyzed free and total thyroxine and triiodothyronine in serum samples of breeding and non-breeding *F. anselli* to examine whether *a*) their thyroid hormone system shows any peculiarities on the genetic level, *b*) these animals have lower hormone levels compared to euthyroid rodents (rats and guinea pigs), and *c*) reproductive status, lifespan and free hormone levels are correlated. Genetic analyses confirmed that Ansell's mole-rats have a conserved thyroid hormone system as known from other mammalian species. Interspecific comparisons revealed that free thyroxine levels of *F. anselli* were about ten times lower than of guinea pigs and rats, whereas the free triiodothyronine levels, the main biologically active form, did not differ significantly amongst species. The resulting fT4:fT3 ratio is unusual for a mammal and potentially represents a case of natural hypothyroxinemia. Comparisons with total thyroxine levels suggest that mole-rats seem to possess two distinct mechanisms that work hand in hand to downregulate fT4 levels reliably. We could not find any correlation between free hormone levels and reproductive status, gender or weight. Free thyroxine may slightly increase with age, based on sub-significant evidence. Hence, thyroid hormones do not seem to explain the different ageing rates of breeders and non-breeders. Further research is required to investigate the regulatory mechanisms responsible for the unusual proportion of free thyroxine and free triiodothyronine.

## Introduction

Most ageing theories assume a link between metabolism and ageing because of several inevitable side effects of metabolic processes that potentially impair somatic integrity in the long term. Examples of such side effects are the production of reactive oxygen species [1], [2], formation of advanced glycation end products [3], [4], [5], and telomere shortening with every cell proliferation cycle [6].

Thyroid hormones (THs) play a major role in development, differentiation and metabolism in vertebrates and are therefore assumed to affect ageing, too [7], [8]. Experimental as well as comparative studies on various mammal models support this assumption. For example, experimentally induced hypothyroidism increases lifespan in rats [9], whereas experimentally induced hyperthyroidism decreased lifespan in young and middle-aged rats [10]. Additionally, *Ames dwarf mice* and *Snell dwarf mice*, which have extraordinary low levels of THs, and other hormones related to growth and development (e.g., somatropin, insulin-like growth factor 1), live significantly longer than wild type mice [11]. Whereas the treatment with somatropin does not have any effect on the lifespan of *Snell dwarf mice*, the administration of THs via food throughout adult life diminishes their lifespan, although it is still longer than in non-treated wild type mice [7], [12]. Furthermore, longevity in vertebrate species is often associated with low metabolic rates, low TH levels, or both. For example, naked mole-rats (*Heterocephalus glaber*) are the longest-living rodent species (lifespan of >30 years) [13], showing only 79% of the allometrically expected resting metabolic rate of non-subterranean rodents [14] and very low levels of certain THs have been reported [15]. Also some long-lived bat species feature low metabolic rates [16], [17]. In humans, there is a significant correlation between low TH metabolism and longevity [7], [18].

In all vertebrates, the main THs are thyroxine (T4) and triiodothyronine (T3). Both THs are derivatives of the amino acid tyrosine and are synthesised in the thyroid gland. Synthesis of T4 and T3 is stimulated by the thyroid-stimulating hormone (TSH), which is released from the pituitary gland. The structures of T4 and T3 are strongly conserved in all mammalian species studied thus far, whereas TSH is species-specific. TSH consists of an unspecific alpha-subunit (TSHA), and a beta-subunit (TSHB), which is responsible for biological specificity [19]. TSH is stimulated by the thyrotropin-releasing hormone (TRH), which is secreted by the hypothalamus [20]. This hypothalamic-pituitary-thyroid (HPT) axis is regulated by THs exerting a negative feedback control over the secretion of TRH and TSH [21]. In peripheral tissues, THs are actively transported through the plasma membrane mainly by the monocarboxylate transporters 8 and 10 (MCT8 and MCT10) and, at least in mice and rats, the organic anion-transporting polypeptide 1C1 [22]. In the cytoplasm, specific deiodinases type 1 and 2 (D1, D2) convert T4 into T3 by deiodination of the outer ring of the T4 molecule [23], [24].

The main biologically active TH, namely T3, regulates gene expression in the nucleus at various loci by binding to two types of thyroid hormone receptors (THRA and THRB) [25]. In addition to these classical TH functions, some non-nuclear TH actions have been described recently [26]. T4 and T3 are typically secreted into the blood stream in a ratio of about 6∶1 (T4:T3) in rats and 14∶1 in humans [27], [28]. Thus, circulating T4 levels are manifold higher than T3 levels in healthy organisms. After secretion, less than 1% of the THs are circulating as biologically active free hormones (fT4 and fT3), while the major amount of T4 and T3 is bound to transport proteins [29], [30].

Ansell's mole-rats (*Fukomys anselli*) are subterranean rodents endemic to Zambia. They show some promising features for ageing and TH studies. Similar to naked mole-rats, which belong to the same family of African mole-rats (Bathyergidae), *F. anselli* live in eusocial families, in which reproduction is usually monopolized by a single breeding pair [31]. The species has an extraordinary maximum lifespan of more than 20 years, which is far more than expected based on their body weight (∼60–150 g). Remarkably, reproductive individuals live about twice as long as non-reproductive animals, regarding both their average and maximum lifespan (breeders: mean ca. 10 years, max. >20 years; non-breeders: mean lifespan ca. 4.8 years, max. 11.1 years ([32], own unpublished data). This bimodal ageing pattern of Ansell's mole-rats and other species of the same genus [33], [34] contradicts the classic model that assumes a trade-off between reproduction and somatic maintenance [35], [36]. Until now, conflicts to this trade-off model have only been reported about eusocial insect species like ants or termites [37], [38]. The mechanisms underlying the unusual ageing pattern of *Fukomys* mole-rats are largely unknown.

Several indications suggest that Ansell's mole-rats may be naturally hypothyroid. First, oxygen consumption of *F. anselli* is significantly lower than expected from allometric equations, suggesting a low metabolic rate [39]. Low resting metabolic rates are typical for bathyergid rodents and are probably a physiological adaptation to their subterranean, low-oxygen environment [40]. Second, the retinae of Ansell's mole-rats show a high density of short-wave sensitive S-cone opsin. This is untypical for rodents, as they usually show S-cone opsins, as well as middle-to-long-wave sensitive L-cone opsins in diverse arrangements [41]. Although the adaptive function of colour perception in mole-rats is not yet understood, the high S-cone opsin density in Ansell's mole-rats would be in line with the expected low TH levels, as fT3 is essential for the expression of L-cone opsins during the prenatal development [42]. Moreover, in athyroid mice, opsin expression can be restored with postnatal T4 treatment [43].

In order to characterize the TH system of Ansell's mole-rats qualitatively and quantitatively, we first sequenced mRNA of several major components of TH related pathways in order to find out whether the TH system of Ansell's mole-rats is evolutionary conserved, or whether it contains qualitative peculiarities compared to other mammalian species. Then, we determined serum TH levels in individuals of different age, sex, and breeding status. Here, we focused mainly on the following two questions: *i*) Do Ansell's mole-rats have lower circulating TH levels than unrelated, euthyroid rodent species (rats, guinea pigs)? *ii*) Are there differences in free TH levels between the slowly ageing reproductive and the faster ageing non-reproductive individuals? We hypothesized that *i*) Ansell's mole-rats have naturally low TH levels in comparison to euthyroid rodents, and *ii*) lower free TH levels in breeding animals (slow ageing) compared to non-breeders (faster ageing) as a possible molecular trigger for the lifespan differences between these two cohorts.

## Materials and Methods

### Animals

All Ansell's mole-rats used in this study were born, raised and maintained at the animal facilities of the Department of General Zoology, University of Duisburg-Essen, Germany. The age of the animals ranged from 1.2–10.2 years in non-breeders and 5.4–13.5 years in breeders at the day of serum sampling. They were housed as family groups in glass terraria on horticultural peat and fed *ad libitum* with carrots and potatoes every day, apples every second day, and grain and lettuce once a week. Room temperature and humidity were kept constant at 24±1°C and 40±3%, respectively.

*Wistar*-*Unilever* rats aged 6–9 months (Central Animal Laboratory of the University Hospital Essen, Germany) and *Dunkin Hartley* guinea pigs aged 12–24 months (Charles River, Wilmington, MA, USA) served as healthy, fully grown euthyroid controls. We did not introduce major age variation within these groups because the intraspecific variation of THs in these species has been summarized and studied elsewhere [44], [45] and was not the focus of our study. Both species were housed at 21±1°C and 55±5% humidity in standard macrolon cages and were fed commercial, species-specific food pellets (ssniff).

### Ethics Statement

Maintenance and all treatments of the animals were approved by the North Rhine-Westphalia State Environment Agency (Permit number: 87–51.04.2010.A359). Blood sampling was the only invasive treatment and was performed under deep anesthesia (ketamine and xylazine or isoflurane), except for guinea pigs, since anaesthesia is not necessary for blood sampling via the *vena saphena* if the procedure is sufficiently quick and appropriate restraining is possible [46]. All efforts were made to minimize suffering.

### Sequencing and sequence analysis

The *F. anselli* transcriptome was characterized by high-throughput sequencing, as will be reported elsewhere. From the resulting data, we extracted transcripts of twelve thyroid-relevant genes. In detail, one male *F. anselli* was deeply anesthetized with isoflurane and killed by cervical translocation. Tissues from thyroid gland, ventral skin, adrenal gland, pancreas, testis, and brain stem were homogenized in a Tissue Lyser (Qiagen), and total mRNA was isolated using RNeasy (Qiagen, Valencia, CA, USA). Thereof, mRNA-Seq libraries were generated using platform-specific chemistry, according to the supplier's instructions (Illumina). Sequencing was performed using an Illumina Genome Analyzer IIx, resulting in a total of 88.7 million (9492 Mbp) single-end reads. Adapter clipping and trimming of low-quality 3′ ends (error probability of 0.5%) was performed with the programs cutadapt [47] and sickle [48], respectively. Reads shorter than 35 nt were removed, resulting in a total of 82.6 million (8,202 Mbp) reads. The transcriptome reads were pooled for a *de-novo* assembly with the Trinity software [49]. Resulting transcript contigs were labelled with gene symbols and were annotated for coding sequences (CDS) based on best bidirectional BLAST mapping against human protein coding genes (National Center for Biotechnology Information [NCBI], *H. sapiens* Annotation Release 104) using in-house scripts. The mRNA sequences were deposited in NCBI GenBank under accession numbers KJ958510-KJ958520 and KM676335. For species comparisons, *F. anselli* mRNA sequences were translated into proteins. These were aligned with orthologous protein sequences from 11 to 17 other mammalian species (RefSeq-database, NCBI) using CLUSTAL W. For genome-wide analysis of evolutionary selection trends we created five-species alignments of the CDS (human, dog, rat, mouse and naked mole-rat) using CLUSTAL W. For the thyroid target genes we additionally created the five-species multiple alignment with the Ansell's mole-rat sequence as the bathyergid representative. We used a parametric model of evolution implemented in the CodeML program of the PAML package [50], [51] in performing the branch test on the mole-rat branch against all other branches as background (options CodonFreq  = 2 and Kappa  = 2). CodeML estimates the ratio of non-synonymous to synonymous mutations (Ka/Ks ratio), among several other parameters, separately for the mole-rat and all other branches. Additionally we used the “M0” model of CodeML to calculate the average Ka/Ks across the whole tree. We estimated the Ka/Ks ratios for the genome-wide orthologous gene set and used these to determine empirical probabilities (“percentiles”) for particular Ka/Ks values, as well as Ka/Ks differences between the mole-rat branch and outer branches. This allowed to relate the Ansell's mole-rat Ka/Ks values of specific genes to the genome-wide Ka/Ks spectrum.

### Sample sizes and sampling protocols

We sampled a total of 32 Ansell's mole-rats (12 breeders and 20 non-breeders, sex-balanced), 4 male rats and 4 male guinea pigs for the free TH measurements.

Additionally, we sampled a subgroup of 12 mole-rats (sex-balanced; both reproductive groups) plus further 7 rats (4 males, 3 females) and 4 guinea pigs (sex-balanced) to determine total TH levels (free and protein-bound fractions in total; tT4 and tT3). Again, free T4 and free T3 were determined in these serum samples in order to calculate the ratios of the free: total fractions for each hormone.

For blood sampling, mole-rats were anesthetized according to a standard protocol for mole-rats with an intramuscular injection of a 6 mg/kg dose of ketamine (10%, Ceva GmbH) and 2.5 mg/kg xylazine (2%, Ceva GmbH) [52]. Rats were isoflurane anesthetized, while guinea pigs did not receive any anaesthesia. To avoid hypothermia, animals were kept under a heat lamp before and after the treatment.

The mole-rat and guinea pig blood samples were taken from the *vena saphe*na of the hind paw with a capillary (Servoprax, 100 µl) and transferred into a serum test tube (Multivette 600, Sarstedt). The rat blood samples were taken by orbita puncture. All samples were taken at approximately the same daytime, in order to avoid any bias due to the circadian variation in TRH secretion [21], [53]. After approximately 20 minutes, the blood samples were centrifuged (Biofuge Pico, Heraeus Instruments) at 900×*g* for 5 minutes [15] and the serum (the clear top layer) was stored at −80°C until use.

### Quantification of thyroid hormones

Free and total thyroxine and triiodothyronine levels were quantified by means of a solid phase competitive enzyme immunoassay (EIA) for human serum (DRG Instruments GmbH). The use of a human serum EIA is justifiable since the molecular structures of T3 and T4 are not species specific ([29], results of the present study].

The accuracy of the fT3 and fT4 microplate EIA test system was confirmed by analyzing known hormone values, and by comparing the results with those of a reference method (radioimmunoassay). The correlation coefficient between the concentrations measured by the two methods was 0.95 (fT3) and 0.96 (fT4), which indicates a high accuracy of the test systems. The intra- and inter-assay variances are shown in Table S1. According to the manufacturer the cross-reactivities of the antibodies were as follows: *Triiodothyronine* − triiodothyronine: 100%; thyroxine: 0.02–0.37%; iodothyrosine, diiodothyrosine, phenylbutzone, sodium salicylate: 0.01–0.2%. *Thyroxine* − thyroxine 100%, triiodothyronine 3%, diiodothyronine, diiodotyrosine and iodotyrosine 0.01%. The assay sensitivities (i.e., detection limits) were 0.05 ng/dl (fT4), 8 nmol/l (tT4), 0.05 pg/ml (fT3), and 0.1 ng/ml (tT3).

### Statistical analyses

The statistical analyses of the thyroid hormone levels were conducted with the software SPSS Statistics v.20.0.0 (IBM Corp.). Normal distribution was tested with the Kolmogorov-Smirnov-test with Lilliefors correction. For interspecies comparisons, one-way ANOVA with Bonferroni post hoc tests were applied. For intraspecific comparisons, a generalized linear model (GLM) was run with sex, reproductive status, age, and weight as independent factors, and fT4 and fT3 values as dependent variables. We calculated *i*) the main effects of the independent factors alone and *ii*) a two factor model using the interaction of status × age as explaining variable.

In the present study, mole-rats proved to have very low fT4 levels in general: 8 out of 32 mole-rats showed fT4 levels below the detection limit (<0.05 ng/dl), and the majority of fT4 values fell relatively close to the detection limit of the assay. It was impossible to decide whether the 8 missing values were failures or represented fT4 levels lower than 0.05 ng/dl. Therefore, analyses of fT4 were run under two different scenarios: 1) treating missing values as failures (effective n = 24; “first scenario” henceforth), and 2) replacing the missing values with the value of the detection limit (0.05 ng/dl) (effective n = 32; “second scenario” henceforth).

## Results

### Molecular constituents of TH system are conserved in *F. anselli*

We first characterized the TH system of Ansell's mole-rats on the genetic level and compared protein sequences with those of several other species representing different mammalian subgroups. Starting with RNA-seq of five different *F. anselli* tissues, we obtained full protein sequences for the two TH receptors (THR alpha [THRA, Figure S1]; THR beta [THRB, Figure S2]), one member of the regulation cascade (TSH beta subunit [TSHB, Figure S3]), two metabolizing deiodinases (D1, Figure S4; D2, Figure S5), two members of the synthesis pathway (thyroglobulin [TG, Figure S6]; thyroperoxidase [TPO, Figure S7]), two transporter proteins (transthyretin [TTR, Figure S8]; thyroxine-binding globulin [TBG, coded by *Serpina7*, Figure S9]), one TH-regulated protein (hypoxia-inducible factor 1 alpha [HIF1A, Figure S10]), one TH transporter (monocarboxylate transporter 8 [MCT8, coded by *Slc16a2*, Figure S11]) and the sodium/iodid symporter (NIS, coded by *SLC5a5*, Figure S12). In order to analyze the conservation status of these molecular markers for the *F. anselli* TH system in comparison to other mammals, we analyzed the ratio of non-synonymous versus synonymous nucleotide changes in the CDS (Ka/Ks; Table 1). In all genes, the Ka/Ks ratio across the selected species was within the 95% percentile of genome-wide Ka/Ks values (≤0.443), indicating average levels of purifying selection. For only one gene (*Serpina7*), the Ka/Ks ratio was outside the 90% percentile (≤0.340), which is well within bounds accounting for multiple testing on eleven genes. Furthermore, in nearly all cases, the branch-specific Ka/Ks ratio of *F. anselli* was close to the ratio of the background branches as well as the average across the tree (Table 1). Exceptions were *Slc16a2*, which showed a slightly higher purifying selection pressure, and *Ttr*, which showed a much weaker purifying selection in the *F. anselli* branch. Nevertheless, for *Ttr*, the absolute Ka/Ks difference between foreground and background branch is within the 80% percentile (*p* = 0.201, one-sided) that is seen genome-wide.

**Table 1:** K_a_/K_s_ ratios indicate persisting purifying selection on proteins of the TH system in *F. anselli*.

Protein (Gene)	K_a_/K_s_ ratio across tree	K_a_/K_s_ ratio *F. anselli* branch	K_a_/K_s_ ratio background branches
THRA	0.011	0.017	0.008
THRB	0.036	0.054	0.033
TG	0.312	0.336	0.305
TPO	0.127	0.088	0.108
D1 (Dio1)	0.229	0.227	0.198
D2 (Dio2)	0.144	0.158	0.149
TSHB	0.209	0.176	0.189
TBG (Serpina7)	0.342	0.358	0.333
TTR	0.226	0.531	0.202
MCT8 (Slc16a2)	0.076	0.007	0.098
NIS (*Slc5a5*)	0.074	0.051	0.068

**Figure 1:**
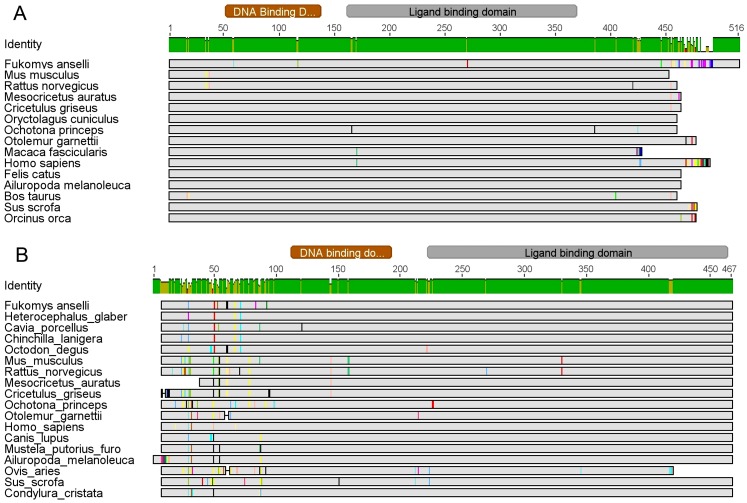
Protein sequence alignment for (A) TH receptor α (THRA) and (B) TH receptor β (THRB) of different mammalian species. *F. anselli* mRNA sequences were derived from RNA-seq and subsequently translated, the other sequences were retrieved from NCBI databases (accession numbers are given in Figures S1 and S2). Sequence differences are marked in gray. “Identity” shows the percentage of amino acid conformity for each position; the protein domain regions correspond to the human sequence entry. A fully resolved representation of the alignment is given as a supplement (Figures S1 and S2).

TSHB, an important upstream molecular target for the study of TH dynamics, shows three species-specific changes in *F. anselli* at positions that are conserved in all 16 other mammalian species (E46D, V112I and F157I; Figure S3). This is comparable to the rate of species-specific changes found in TSHB of other mammals, ranging from 0 to 9. In pairwise comparisons to the sequence of *F. anselli*, the number of amino acid differences varies from 10 (to thirteen-lined ground squirrel, *Ictidomys tridecemlineatus*) to 24 (guinea pig), which underlines that even closely related species (Ansell's mole-rats and guinea pigs) show high variations in the TSHB sequence. Finally, we analyzed the *F. anselli* TH receptors, particularly their ligand-binding domains, as specific markers for conservation of the TH molecules. The ligand-binding domain of *F. anselli* THRA (protein position 190–370) shows only one conservative change from glutamic acid to aspartic acid (E270D) when compared to the consensus of 14 other mammalian species (Figure 1a, Figure S1). The ligand-binding domain of THRB (position 222–464) does not show a single amino acid difference between *F. anselli* and the sequence consensus of 17 other mammalian species (Figure 1b, Figure S2).

### Free TH levels: Ansell's mole-rats have low fT4, but normal fT3

**Figure 2:**
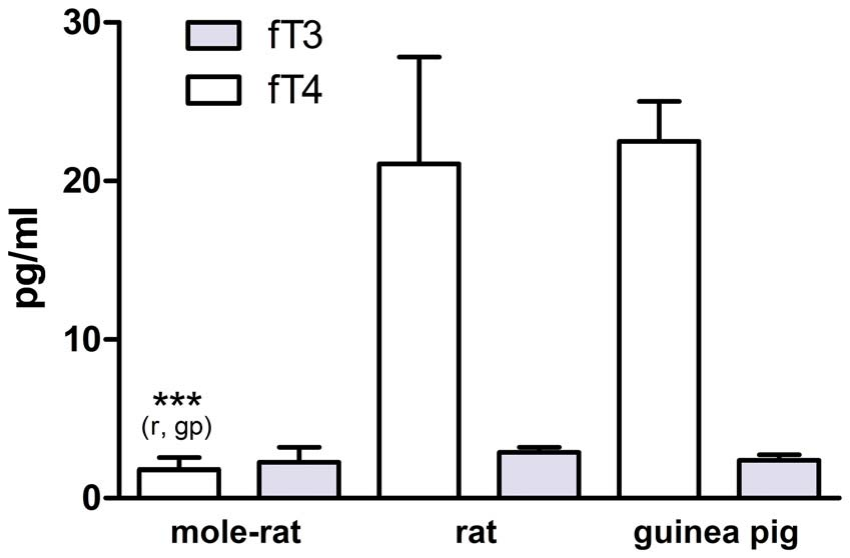
Free T4 and free T3 levels of Ansell's mole-rats (n = 24), rats (n = 4) and guinea pigs (n = 4). Mean ± SD; all data expressed in pg/ml. One-way ANOVA, fT4: *F* = 206.38, *p*<0.0001; fT3: *F* = 0.85, *p* = 0.44. Significant differences in the Bonferroni post hoc comparisons are indicated by asterisks coupled with the comparison species referred to (mr  =  mole-rat, r =  rat, gp  =  guinea pig) in parenthesis. See “Results” section for statistical details and Table 3 for TH ratios obtained from these data. Mole-rat fT4 data refer to scenario 1; applying the second scenario (not depicted here) created essentially the same result because mean fT4 values of mole-rats were slightly lower.

Under both scenarios, Ansell's mole-rat fT4 levels (0.18±0.08 ng/dl [n = 24] and 0.15±0.09 ng/dl [n = 32]; Table 2) were about 10 times lower than fT4 measured in rats (2.11±0.67 ng/dl) and guinea pigs (2.25±0.25 ng/dl; one-way ANOVA: *F* [first/second scenario]  = 206.38/273.96, *p*<0.0001; Bonferroni post hoc comparisons in both scenarios: mole-rat vs. rat and mole-rat vs. guinea-pig: *p*<0.0001; rat vs. guinea-pig: *p*>0.99); Figure 2). By contrast, fT3 levels did not differ significantly among the three species (mole-rat: 2.24±0.96 pg/ml; rat: 2.85±0.34 pg/ml; guinea pig: 2.36±0.35 pg/ml; one-way ANOVA: *F* = 0.85, *p* = 0.44; Figure 2).

**Table 2:** Mean (±SD) fT4 and fT3 values in Ansell's mole-rats.

	fT4 (ng/dl; n = 24)			fT4 (ng/dl; n = 32)			fT3 (pg/ml; n = 32)		
	female	male	all	female	male	all	female	male	all
**R** 1	0.20±0.11 (n = 5)	0.17±0.08 (n = 6)	0.18±0.09 (n = 11)	0.18±0.12 (n = 6)	0.17±0.08 (n = 6)	0.17±0.09 (n = 12)	3.01±2.02 (n = 6)	2.24±0.49 (n = 6)	2.63±1.45 (n = 12)
**NR** ^2^	0.18±0.07 (n = 9)	0.16±0.06 (n = 4)	0.17±0.06 (n = 13)	0.17±0.08 (n = 10)	0.09±0.07 (n = 10)	0.13±0.08 (n = 20)	1.97±0.23 (n = 10)	2.04±0.43 (n = 10)	2.01±0.34 (n = 20)

1 NR = non-reproductive; R = reproductive.

### Total TH levels: Ansell's mole-rats have low tT4 and low tT3

**Figure 3:**
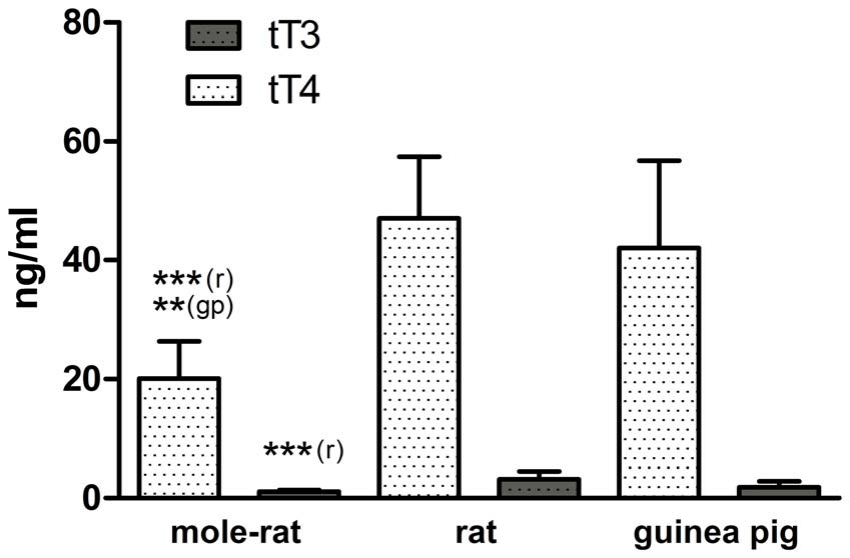
Total T4 and total T3 levels of Ansell's mole-rats (n = 12), rats (n = 7) and guinea pigs (n = 4). Mean ± SD; all data expressed in ng/ml. One-way ANOVA, tT4: *F = *19.51, p<0.0001; fT3: *F = *11.13, *p* = 0.001. Significant differences in the Bonferroni post hoc comparisons are indicated by asterisks coupled with the comparison species referred to (mr  =  mole-rat, r =  rat, gp  =  guinea pig) in parenthesis. See “Results” section for statistical details and Table 3 for TH ratios obtained from these data.

Ansell's mole-rat tT4 levels (20.07±6.32 ng/ml) were significantly lower than those of rats (47.05±10.39 ng/ml) and guinea pigs (42.01±14.77 ng/ml; one-way ANOVA, *F* = 19.51, *p*<0.0001; Bonferroni post hoc comparison: mole-rat vs. rat: *p*<0.0001; mole-rat vs. guinea pig: *p* = 0.003; rat vs. guinea pig: *p*>0.99; Figure 3). Levels of tT3 were significantly lower between mole-rats (1.13±0.25 ng/ml) and rats (3.14±1.34 ng/ml), but not guinea pigs (1.80±1.03 ng/ml; one-way ANOVA: *F* = 11.13, *p* = 0.001; Bonferroni post hoc comparison: mole-rat vs. rat: *p*<0.0001; mole-rat vs. guinea pig: *p* = 0.629; rat vs. guinea pig: *p* = 0.076; Figure 3).

### Hormone ratios

**Figure 4:**
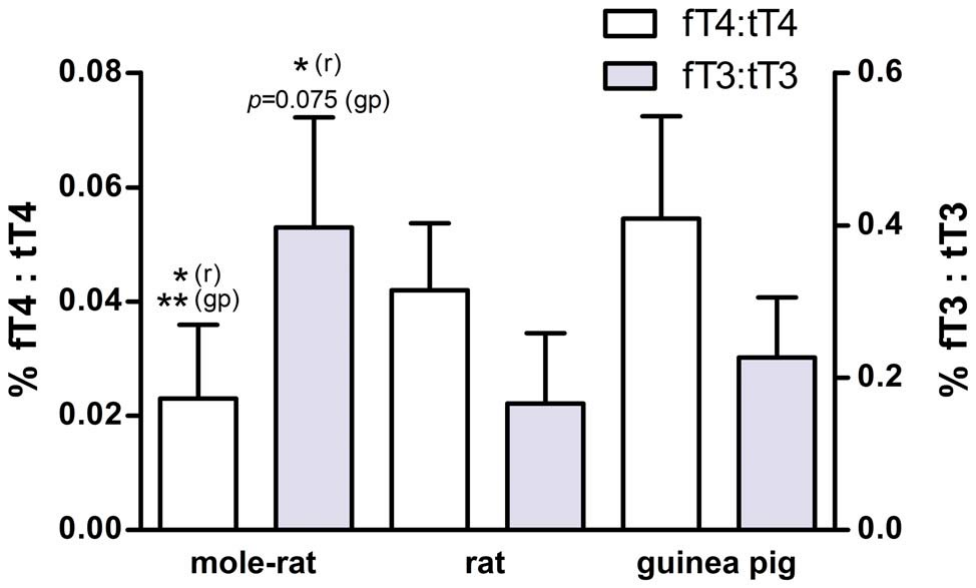
fT4:tT4 and fT3:tT3 ratios in Ansell's mole-rats (n = 12), rats (n = 7) and guinea pigs (n = 4). One-way ANOVA, fT4:tT4: *F = *9.60, *p* = 0.001; fT3:tT3: *F = *7.724, *p = *0.004. Significant differences in the Bonferroni post hoc comparisons are indicated by asterisks coupled with the comparison species referred to (mr  =  mole-rat, r =  rat, gp  =  guinea pig) in parenthesis. See “Results” section for statistical details.

We estimated the percentage of unbound hormone fractions by determining the proportion of fTH in relation to tTH per serum sample. The proportion of fT4 to tT4 was significantly lower in mole-rats (0.02±0.01%) compared to rats (0.04±0.01%) and guinea pigs (0.05±0.02%; one-way ANOVA: *F* = 9.60, *p* = 0.001; Bonferroni post hoc comparison: mole-rats vs. rat: *p* = 0.02; mole-rats vs. guinea pig: *p* = 0.002; rat vs. guinea pig: *p* = 0.496; Figure 4). In contrast, the proportion of fT3 to tT3 was highest in mole-rats (0.38±0.14%; rats: 0.17±0.09%; guinea pigs 0.23±0.08%), with differences being statistically significant in the comparison with rats and close to significance threshold in the comparison with guinea pigs (one-way ANOVA: *F* = 7.724, *p* = 0.004; Bonferroni post hoc comparison: mole-rats vs. rats: *p* = 0.004; mole-rats vs. guinea pig: *p* = 0.075; rat vs. guinea pig: *p*>0.99; Figure 4).

We also calculated the ratios of free T4:T3, and total T4:T3 in all three species (Table 3). Free T4:T3 ratios in mole-rats (0.70±0.45–0.85±0.43, depending on the scenario) were significantly lower than in both other species (rats: 7.28±1.87, guinea pigs: 9.63±1.50; one-way ANOVA [first/second scenario]: *F* = 240.53/309.71, *p*<0.0001 each; Bonferroni post hoc comparison: mole-rat vs. rat: *p*<0.0001 [both scenarios]; mole-rat vs. guinea pig: *p*<0.0001; rat vs. guinea pig: *p* = 0.002). In contrast, tT4:tT3 ratios did not differ between mole-rats and the other species (mole-rats: 18.60±6.98, rats: 16.24±4.28, guinea pigs: 26.43±9.51; one-way ANOVA: *F* = 3.00, *p* = 0.07; Bonferroni post hoc comparison: mole-rat vs. rat: *p*>0.99; mole-rat vs. guinea pig: *p* = 0.17; rat vs. guinea-pig: *p* = 0.08).

**Table 3:** Ratios (± SD) of free and total T4:T3 in interspecies comparison.

Species	Ratio fT4:fT3	Ratio tT4:tT3
Mole-rat	0.70±0.45* 0.85±0.43†	18.60±6.98
Rat	7.28±1.87	16.24±4.28
Guinea pig	9.63±1.50	26.43±9.51

fT4:fT3 ratios were obtained from the data shown in Figure 2 and tT4:tT3 ratios were obtained from data shown in Figure 3. See “Results” section for statistical details.

*: scenario 2 (n = 32).

† : scenario 1 (n = 24).

### Free thyroid hormone levels do not explain intraspecific ageing differences

Intraspecific comparisons revealed, that the fT4 levels of Ansell's mole-rats were not affected by any of the tested factors sex, reproductive status, age and weight alone in the first scenario (n = 24), and neither so by the interaction of reproductive status × age (Table 4). In the second scenario (n = 32), the fT4 levels were again not affected by sex, reproductive status and weight alone, nor by the interaction of status × age (Table 4). Levels fT4 increased significantly with age under this scenario (*p* = 0.041; Table 4).

**Table 4:** Intraspecific fT4 (both scenarios) and fT3 differences in Ansell's mole-rats.

Factor	fT4 first scenario (n = 24)		fT4 second scenario (n = 32)		fT3 (n = 32)	
	*F*	*p*	*F*	*p*	*F*	*p*
**Sex**	2.47	0.13	4.00	0.056	0.90	0.35
**Reproductive status**	0.54	0.47	0.14	0.71	0.62	0.44
**Age**	2.48	0.13	4.58	0.041*	0.63	0.44
**Weight**	2.04	0.17	0.59	0.45	0.32	0.58
**Reproductive status × age**	0.71	0.41	0.04	0.84	1.29	0.28

GLM main effects for all four factors alone and a GLM two factor model with reproductive status × age as independent variable. The *p*-values and the correlation coefficients *F* are shown.

* =  significant (*p*<0.05).

Free T3 levels were not influenced by any of the tested factors or interactions in the GLM (Table 4).

## Discussion

The aim of our study was to characterize the TH system of Ansell's mole-rats and to determine T4 and T3 levels in this species to investigate if these animals are hypothyroid, and if their hormone concentrations correlate with their extraordinary lifespan and the bimodal ageing pattern of reproductive and non-reproductive animals.

### Molecular constituents of TH system are conserved in *F. anselli*

In *F. anselli*, the major molecular constituents of the mammalian TH system could be identified via their mRNAs, namely TG, TPO, TSHB, D1, D2, TTR, MCT8, TBG and NIS. The mRNAs show full protein-coding capacity, and sequence substitution patterns suggest that purifying selection acts on these molecules to a similar extent as found in other mammal species (Table 1). However, the mutational drift for some proteins is such high that immunochemical detection methods, e.g. for TSHB, will require development of species-specific antibodies. In addition, preliminary work showed no results for *F. anselli* samples with commercially available TSH assays, both for human and guinea pig, which confirms the species-specificity of TSH as well.

Most notably, the two TH receptor isoforms, THRA and THRB, show a high level of sequence conservation in *F. anselli* compared to 15 mammalian species, especially within their hormone-binding domains (Figure 1, Figures S1 and S2). This finding supports the expectation that just as in other mammals, the structures of T4 and T3 are conserved in *F. anselli*, although these were not directly determined.

### Interspecies comparison of TH hormone levels

The first striking result of our study is that serum fT4 levels were about 10 times lower in mole-rats than in guinea pigs and rats, regardless of the scenario applied. Low circulating T4 levels are often caused by iodine deficiency in the diet [54], but this explanations appears unlikely in Ansell's mole-rats, because carrots, which they receive *ad libitum*, contain more iodine than required [54]. Moreover, the sequence analysis of the natrium/iodide symporter (NIS; Figure S12) appears to be under strong purifying selection in Ansell's mole-rats as in other mammals (Table 1), thus reducing the possibility of an iodide deficiency in the thyroid gland. Note that typical symptoms of a lifelong iodide deficiency like e.g. goiters [55] have not been observed in *Fukomys* (own unpublished data) or *Heterocephalus* mole-rats [15] so far. Thus it is plausible to assume that the low fT4 and tT4 levels reported here reflect the natural status of these animals, which provides principal support for our first hypothesis that Ansell's mole-rats have naturally low TH levels in comparison to euthyroid rodents. As shown in various vertebrate species, long lifespan is often correlated with low T4 levels, low metabolic rates, or both [7], [16]–[18]. Many long-lived bathyergid species, including *F. anselli*, have very low metabolic rates [39], [40], [56], and the only member of the family *Bathyergidae* in which TH levels have been quantified so far (the naked mole-rat) has also shown remarkably low fT4 [15]. The T4 levels observed in the present study are in good agreement with these findings.

Considering that the fT4 levels in mole-rats are by an order of magnitude lower than in rats and guinea pigs, it is interesting to note that after 30 years of maintaining and breeding mole-rats, we have no indications for developmental or cognitive impairments of the progeny. This is noteworthy, because in other animal models (chicken, rats and mice), induction of even mild fT4 deficits in the mother during prenatal development affects brain development, potentially leading to significant cognitive and/or motoric impairments in the progeny [57]–[59]. In humans, maternal fT4 deficits during pregnancy are associated with an elevated risk of cognitive impairments in the child, including severe disorders like e.g. autism [60], [61]. Preliminary own data suggests that female mole-rats do not elevate fT4 levels during pregnancy. Should this assumption be verified, it will be worthwhile to investigate the mechanisms that enable Ansell's mole-rats to deal with such low maternal fT4 levels during prenatal development without ontogenetic impairments.

The low fT4 values may, however, help to explain the unexpectedly high S-cone opsin concentration in the retina of Ansell's mole-rats [41], because THs are essentially involved in the expression of L-opsins in the mammalian retina by binding to a THRB isoform in the cones [42], [43]. The adaptive value of colour perception for a strictly subterranean rodent is probably residual; studies by Kott et al. [62] suggest that while rods play an important role in the subterranean habitat, cones, especially S-cones, have no specific adaptive function. Our results provide the alternative explanation that the expression of these S-opsins could be a side effect of a natural state of low T4, which has evolved for other reasons (in this case potentially metabolism).

Total thyroxine levels (tT4) were also significantly lower in mole-rats than in the controls, but the differences were less pronounced than in the free hormone fractions; on average, mole-rat tT4 levels reached about 50% of those measured in rats and guinea pigs. Of note, we found that also the fT4/tT4 ratio is significantly lower in mole-rats than in the two control species (Figure 4). It hence appears that mole-rats do not only produce less T4 in their thyroid glands, but also recruit lesser proportions of their total T4 resources into the active form. Taken together, mole-rats seem to possess two distinct mechanisms that work hand in hand to downregulate fT4 levels reliably, which indicates an adaptive function of low T4 levels in these animals. We will discuss potential proximate mechanisms for their maintenance later in this manuscript.

Interestingly, and in sharp contrast to the low T4, fT3 levels were undistinguishable between *F. anselli* and the euthyroid controls (Figure 2). Total T3 levels of mole-rats were also not statistically different from those measured in guinea pigs, but significantly lower than in rats (Figure 3). The ratios between free and total T3 (Figure 4) suggest that mole-rats recruit significantly higher portions of the available T3 into the active unbound form than the other two species, counteracting the much lower T4 levels. Although these results should be treated with some caution because our rat tT3 levels appear atypically high (see e.g. [29], [63], [64] where rat tT3 levels between 0.8 ng/ml–1.62 ng/ml have been reported), there is little doubt that the overall T3 pattern differs quite clearly compared to T4.

The combination of low fT4 and “normal” fT3 resulted in a very uncommon fT4:fT3 ratio of only 0.70–0.85 (depending on the scenario applied) in mole-rats, compared to 7.28 and 9.63 in rats and guinea pigs, respectively (Table 3), the latter being in good agreement with published data [28], [29]. This phenotype resembles that of hypothyroxinemia, a condition characterized by low levels of fT4 while TSH and often also fT3 are in a normal range or slightly elevated [57]. However, whether mole-rats are naturally hypothyroxinemic cannot be answered until TSH can be quantified reliably also in mole-rats.

Regardless of the terminology, our findings raise interesting questions about the proximate and ultimate mechanisms being responsible for this unusual and hitherto unreported hormone distribution. We have already discussed that mole-rats seem to recruit less T4 and more T3 from their respective resources than other rodents. Both may be linked to higher expression rates and/or binding affinities of the mole-rat TH binding proteins. In rodents, the main known binding proteins are albumin, TTR and TBG [65], and the combination of their expression rates and binding affinities have major influence on the half-life of circulating THs. TBG, for instance, has a high T4 binding affinity, but is expressed at very different levels across the lifetime of rats [66]. Specific expression rates and/or functional mutations affecting binding affinities of TBG and other involved proteins could potentially provide an explanation for the altered ratios between free and total TH fractions observed in Ansell's mole-rats and should therefore be focussed in future investigations. For instance, amino acid changes in TTR, at position 109 or 119, were shown to increase thyroxine affinity and decrease fT4/fT3 ratio in humans [67]. However, in the present study no such changes were observed in Ansell's mole-rats (Figure S8).

The observed TH pattern could be linked to alterations in deiodination rates in and/or efflux rates out of target cells. Deiodination of T4 to T3 takes place in the cytoplasm of target cells [24], [68]. A higher D1 and D2 activity, both responsible for converting T4 to T3 [24], and/or a high efflux of T3 out of the cells could lead to a relatively high T3 concentration in the blood stream [69] and help compensate for low levels of T4. Therefore, expression rates of the regulatory components of the TH system as well as D1, D2 and D3 activities in the brain, the thyroid and peripheral organs should be determined in further studies.

The “normal” fT3 concentration is rather unexpected on the basis of the low metabolic rate of Ansell's mole-rats [39], [40] and the low L-opsin density in the retina [41]. Thus, alternative functions of T3 could help explain these contradictions: For instance, novel signalling pathways of T3, which imply indirect activation of transcription as a non-nuclear activity, are discussed. One such pathway initiated by THs is the activation of the transcription of the alpha subunit of hypoxia-inducible factor 1 (HIF1A). It is a transcription factor found in all mammalian cells and responsible for a wide range of cellular responses to hypoxia [70], [71]. In human fibroblasts, HIF1A mRNA and protein concentration are upregulated by a pathway which is activated by T3 binding to THRB in the cytoplasm [72], [73] without being transported into the nucleus.

Therefore, the maintenance of normal T3 levels despite low T4 levels may be an adaptive cellular mechanism of animals living in hypoxic environments to assure a more specific and continuous availability of HIF1. Of course, this is speculative at the moment. However, the importance of HIF1 in subterranean environments is supported by findings from another strictly subterranean mammal, *Spalax ehrenbergi*. In the skeletal muscles of these animals, the concentration of HIF1A mRNA is significantly higher than in rats [74].

Not surprisingly, a remarkably high concentration of HIF1A was also detected in the brain of old naked mole-rats [75], [76]. This suggests that this kind of adaptation to a hypoxic environment is not restricted to *S. ehrenbergi* and may also be found in bathyergid species. Further research has to confirm whether these adaptations also occur in *F. anselli*.

### Intraspecies comparison of TH hormone levels

Intraspecific fT4 and fT3 comparisons suggest that THs are not the major determinants of the caste-specific ageing rates found in Ansell's mole-rats. In neither scenario was there a significant difference in hormone levels (fT4 or fT3) between non-breeders and breeders. Likewise, sex and weight of the animals did not have an influence on hormone levels. On the other hand fT4 levels did seem to increase with age, when applying the second scenario (Table 4). Age effects on TH levels are well-known, which is not surprising, because THs play a major role in development and metabolism. However, in other mammalian species, THs usually decline with age. In human for instance, fT3 levels usually decline with age, while fT4 levels remain more or less unchanged [77], [78]. Guinea pigs do not show an alteration in serum fT4 levels as well [45].

In summary, our results indicate that in *F. anselli*, euthyroid fT3 levels are coupled with lower circulating levels of T4, which, in combination with their low metabolic rate, may represent a novel mechanism to cope with the hypoxic subterranean environment these animals have adapted to. However, THs do not seem to have a major influence on the intraspecific ageing rates in these mole-rats.

## Supporting Information

Figure S1 **Protein alignment of thyroid hormone receptor α (THRA) from different mammal species.** The mRNA sequence of *F. anselli* was obtained from RNA-seq and subsequently translated, other sequences were retrieved from NCBI databases with the following accession numbers: Mus musculus (CAA30576), Rattus norvergicus (NP_112396), Mesocricetus auratus (XP_005076008), Cricetulus_griseus (XP_003510526), Oryctolagus cuniculus (XP_002719397), Ochotona princeps(XP_004591220), Otolemur garnettii (XP_003786450), Macaca fascicularis (NP_001270601),Homo sapiens (NP_003241),Felis catus (XP_003996845), Ailuropoda melanoleuca (XP_002924975), Bos Taurus (NP_001039794), Sus scrofa (O97716), Orcinus orca (XP_004282800).
(PDF)

Figure S2 **Protein alignment of thyroid hormone receptor β (THRB) from different mammal species.** The mRNA sequence of *F. anselli* was obtained from RNA-seq and subsequently translated, other sequences were retrieved from NCBI databases with the following accession numbers: Heterocephalus glaber (XP_004892721), Cavia porcellus (XP_005008354), Chinchilla lanigera (XP_005387539), Octodon degus (XP_004634245), Mus musculus (P37242), Rattus norvegicus (P18113), Mesocricetus auratus (XP_005081037), Cricetulus griseus (ERE87100), Ochotona princeps (XP_004588346), Otolemur garnettii (XP_003781795), Homo sapiens (P10828), Canis lupus (XP_862690), Mustela putorius furo (XP_004786805), Ailuropoda melanoleuca (XP_002928077), Ovis aries (Q28571), Sus scrofa (XP_001928500), Condylura cristata (XP_004692336).
(PDF)

Figure S3 **Protein alignment of thyreotropin β subunit (TSHB) from different mammal species.** The mRNA sequence of *F. anselli* was obtained from RNA-seq and subsequently translated, other sequences were retrieved from NCBI databases with the following accession numbers: Cavia porcellus (XP_003479306), Octodon degus (XP_004641725), Mus musculus (NP_001159412), Rattus norvegicus (NP_037248), Ictidomys tridecemlineatus (XP_005334966), Otolemur garnettii (XP_003793878), Macaca fascicularis (XP_001111873), Nomascus leucogenys (XP_003268073), Gorilla gorilla (XP_004026450), Pan paniscus (XP_003805682), Pan troglodytes (XP_001160337), Homo sapiens (AAB30828), Bos taurus (XP_005204060), Orcinus orca (XP_004263279), Echinops telfairi (XP_004714911).
(PDF)

Figure S4 **Protein alignment of Type I iodothyronine deiodinase (D1) from different mammal species.** The mRNA sequence of *F. anselli* was obtained from RNA-seq and subsequently translated, other sequences were retrieved from NCBI databases with the following accession numbers: Heterocephalus glaber (XP_004908861), Cavia porcellus (NP_001244903), Octodon degus (XP_004642620), Mus musculus (Q61153), Rattus norvegicus (CAA41063), Cricetulus griseus (NP_001243688), Ochotona princeps (XP_004588749), Otolemur garnettii (XP_003793192), Macaca mulatta (NP_001116124), Pan troglodytes (NP_001116123), Homo sapiens (NP_000783), Canis lupus (NP_001007127), Felis catus (NP_001009267), Bos taurus (NP_001116065), Sus scrofa (NP_001001627), Equus caballus (NP_001159924), Orcinus orca (XP_004273874).
(PDF)

Figure S5 **Protein alignment of Type II iodothyronine deiodinase (D2) from different mammal species.** The mRNA sequence of *F. anselli* was obtained from RNA-seq and subsequently translated, other sequences were retrieved from NCBI databases with the following accession numbers: Heterocephalus glaber (XP_004900438), Chinchilla lanigera (XP_005390287), Octodon degus (XP_004624767), Mus musculus (NP_034180), Rattus norvegicus (NP_113908), Ochotona princeps (XP_004584413), Homo sapiens (AAC95470), Canis lupus (NP_001116117), Ovis aries (XP_004011138), Sus scrofa (NP_001001626), Equus caballus (NP_001159927), Orcinus orca (XP_004262346), Condylura cristata (XP_004681708), Echinops telfairi (XP_004698804).
(PDF)

Figure S6 **Protein alignment of thyroglobulin (TG) from different mammal species.** The mRNA sequence of *F. anselli* was obtained from RNA-seq and subsequently translated, other sequences were retrieved from NCBI databases with the following accession numbers: Cavia porcellus (XP_003467392), Chinchilla lanigera (XP_005398080), Octodon degus (XP_004642544), Mus musculus (AAB53204), Rattus norvegicus (BAL14775), Ochotona princeps (XP_004580794), Otolemur garnettii (XP_003792914), Macaca mulatta (EHH28780), Pan troglodytes (XP_003311969), Homo sapiens (AAC51924), Canis lupus (XP_005627864), Felis catus (XP_004000173), Sus scrofa (NP_001161890), Equus caballus (XP_001916622), Orcinus orca (XP_004265356), Echinops telfairi (XP_004697442).
(PDF)

Figure S7 **Protein alignment of thyroperoxidase (TPO) from different mammal species.** The mRNA sequence of *F. anselli* was obtained from RNA-seq and subsequently translated, other sequences were retrieved from NCBI databases with the following accession numbers: Cavia porcellus (XP_003464975; patched), Octodon degus (XP_004644658), Mus musculus (EDL36934), Rattus norvegicus (EDM03234), Cricetulus griseus (XP_003501455), Ochotona princeps (XP_004582879), Otolemur garnettii (XP_003798602), Macaca mulatta (XP_001117795), Homo sapiens (XP_005264756), Canis lupus (Q8HYB7), Felis catus (XP_003984594), Bos taurus (XP_603356), Sus scrofa (P09933), Equus caballus (XP_001918216), Orcinus orca (XP_004274968), Echinops telfairi (XP_004709888).
(PDF)

Figure S8 **Protein alignment of transthyretin (TTR) from different mammal species.** The mRNA sequence of *F. anselli* was obtained from RNA-seq and subsequently translated, other sequences were retrieved from NCBI databases with the following accession numbers: Heterocephalus glaber (XP_004905241), Chinchilla lanigera (XP_005372800), Octodon degus (XP_004623610), Rattus norvegicus (AAA41801), Mesocricetus auratus (XP_005065406), Cricetulus griseus (XP_003510202), Ictidomys tridecemlineatus (XP_005337518), Oryctolagus cuniculus (XP_002713532), Chlorocebus aethiops (BAL44398), Homo sapiens (CAG33189), Equus caballus (XP_001495232), Echinops telfairi (XP_004702987).
(PDF)

Figure S9 **Protein alignment of thyroxine-binding globin (TBG) from different mammal species.** The mRNA sequence of *F. anselli* was obtained from RNA-seq and subsequently translated, other sequences were retrieved from NCBI databases with the following accession numbers: Heterocephalus glaber (EHB09876), Octodon degus (XP_004646260), Mus musculus (P61939), Rattus norvegicus (AAA42205), Cricetulus griseus (ERE65740), Otolemur garnettii (XP_003801681), Gorilla gorilla (XP_004064693), Pan troglodytes (NP_001009109), Homo sapiens (NP_783866), Canis lupus (XP_538128), Bos taurus (AAI03464), Ovis aries (NP_001094390), Sus scrofa (Q9TT35), Equus caballus (XP_001493492), Orcinus orca (XP_004285286), Echinops telfairi (XP_004710081).
(PDF)

Figure S10 **Protein alignment of hypoxia-induced factor (HIF1A) from different mammal species.** The mRNA sequence of *F. anselli* was obtained from RNA-seq and subsequently translated, other sequences were retrieved from NCBI databases with the following accession numbers: Heterocephalus glaber (XP_004837489), Octodon degus (XP_004624861), Mus musculus (CAA70305), Rattus norvegicus (O35800), Ochotona princeps (XP_004597684), Otolemur garnettii (XP_003794480), Pan troglodytes (XP_001168972), Homo sapiens (NP_001521), Canis lupus (XP_003639249), Felis catus (XP_003987765), Bos taurus (NP_776764), Sus scrofa (NP_001116596), Orcinus orca (XP_004262152).
(PDF)

Figure S11 **Protein alignment of Monocarboxylate transporter 8 (MCT8) from different mammal species.** The mRNA sequence of *F. anselli* was obtained from RNA-seq and subsequently translated, other sequences were retrieved from NCBI databases with the following accession numbers: Heterocephalus glaber (XP_004905154), Cavia porcellus (XP_005004229), Octodon degus (XP_004648070), Mus musculus (AAC40078), Rattus norvegicus (EDM07172), Ochotona princeps (XP_004592817), Otolemur garnettii (XP_003802275), Macaca mulatta (XP_001096017), Homo sapiens (NP_006508), Bos taurus (NP_001193868), Orcinus orca (XP_004283857). Suggestions of NCBI for translation starts of Octodon degus (XP_004648070), Ochotona princeps (XP_004592817), Macaca mulatta (XP_001096017) and Bos taurus (NP_001193868) were changed to the position of the orthologous sequences.
(PDF)

Figure S12 **Protein alignment of Natrium-Iodid-Symporter (NIS) from different mammal species.** The mRNA sequence of *F. anselli* was obtained from RNA-seq and subsequently translated, other sequences were retrieved from NCBI databases with the following accession numbers: Heterocephalus glaber (XP_004873534), Cavia porcellus (XP_003465226), Octodon degus (XP_004646953), Mus musculus (NP_444478), Rattus norvegicus (Q63008), Otolemur garnettii (XP_003796602), Macaca mulatta (EHH29802), Pan troglodytes (XP_524154), Homo sapiens (NP_000444), Canis lupus (XP_541946), Sus scrofa (NP_999575), Orcinus orca (XP_004277608).
(PDF)

Table S1 **Intra- and inter-assay variances of the Enzyme Immunoassays for fT3, fT4, tT3 and tT4.** Shown are the coefficients of variances (%CV) of the assays used in the present study, according to the manufacturer (DRG Instruments GmbH).
(PDF)
