# Supplementary material for: Unusual Ratio between Free Thyroxine and Free Triiodothyronine in a Long-Lived Mole-Rat Species with Bimodal Ageing
Source: PLoS One. 2014 Nov 19;9(11):e113698. doi: 10.1371/journal.pone.0113698 (PMC4237498; doi:10.1371/journal.pone.0113698)
Supplement: Figure S1 — Protein alignment of thyroid hormone receptor α (THRA) from different mammal species. The mRNA sequence of F. anselli was obtained from RNA-seq and subsequently translated, other sequences were retrieved from NCBI databases with the following accession numbers: Mus musculus (CAA30576), Rattus norvergicus (NP_112396), Mesocricetus auratus (XP_005076008), Cricetulus_griseus (XP_003510526), Oryctolagus cuniculus (XP_002719397), Ochotona princeps(XP_004591220), Otolemur garnettii (XP_003786450), Macaca fascicularis (NP_001270601),Homo sapiens (NP_003241),Felis catus (XP_003996845), Ailuropoda melanoleuca (XP_002924975), Bos Taurus (NP_001039794), Sus scrofa (O97716), Orcinus orca (XP_004282800). (PDF) [file pone.0113698.s001.pdf]

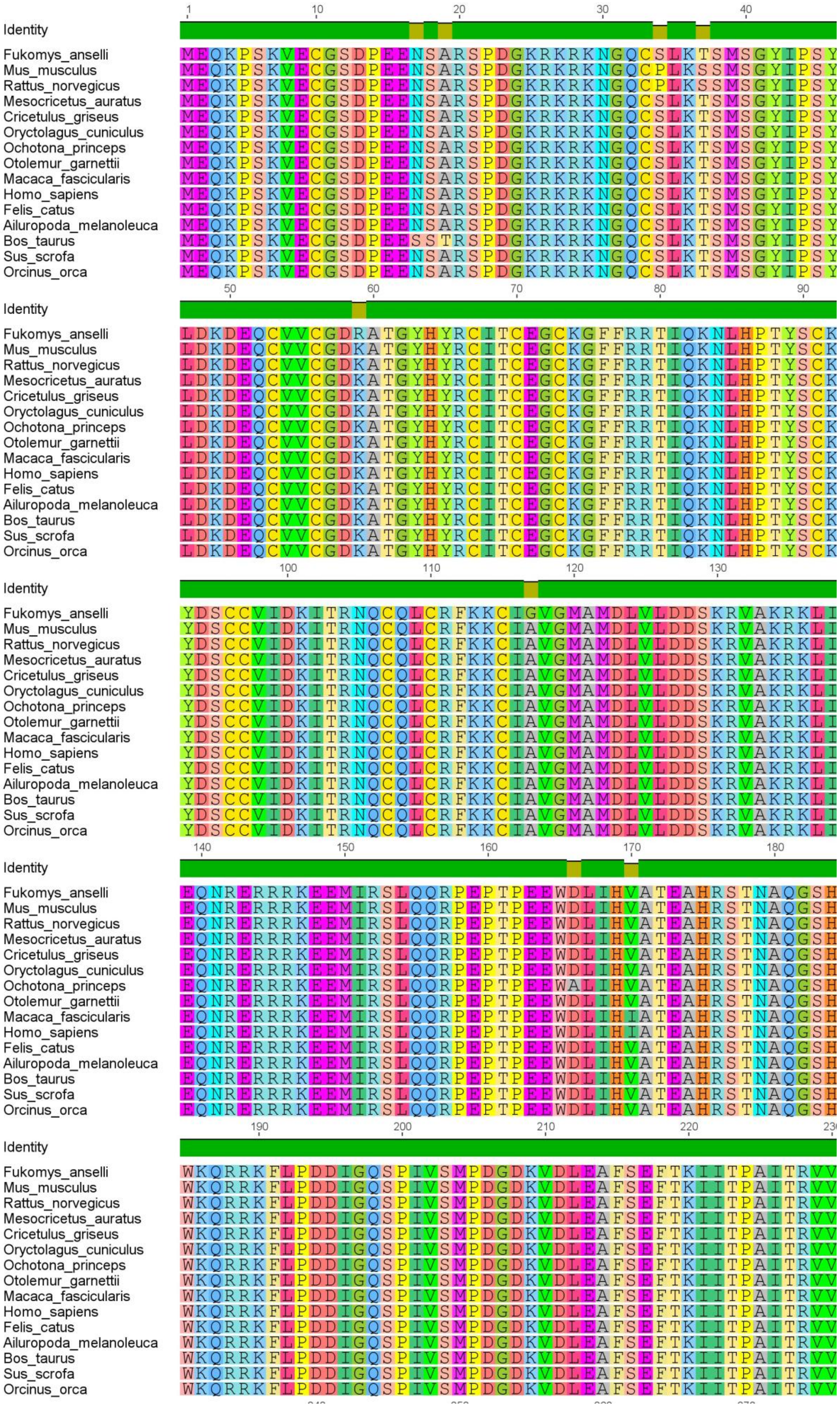

1 10 20 30 40
Identity
Fukomys_anselli MEQKPSKVECGSDPEENSARSPDGKRKRKNGQCSLKTSMSGYIPSY
Mus_musculus MEQKPSKVECGSDPEENSARSPDGKRKRKNGQCPLKSSMSGYIPSY
Rattus_norvegicus MEQKPSKVECGSDPEENSARSPDGKRKRKNGQCPLKSSMSGYIPSY
Mesocricetus_auratus MEQKPSKVECGSDPEENSARSPDGKRKRKNGQCSLKTSMSGYIPSY
Cricetulus_griseus MEQKPSKVECGSDPEENSARSPDGKRKRKNGQCSLKTSMSGYIPSY
Oryctolagus_cuniculus MEQKPSKVECGSDPEENSARSPDGKRKRKNGQCSLKTSMSGYIPSY
Ochotona_princeps MEQKPSKVECGSDPEENSARSPDGKRKRKNGQCSLKTSMSGYIPSY
Otolemur_garnettii MEQKPSKVECGSDPEENSARSPDGKRKRKNGQCSLKTSMSGYIPSY
Macaca_fascicularis MEQKPSKVECGSDPEENSARSPDGKRKRKNGQCSLKTSMSGYIPSY
Homo_sapiens MEQKPSKVECGSDPEENSARSPDGKRKRKNGQCSLKTSMSGYIPSY
Felis_catus MEQKPSKVECGSDPEENSARSPDGKRKRKNGQCSLKTSMSGYIPSY
Ailuropoda_melanoleuca MEQKPSKVECGSDPEENSARSPDGKRKRKNGQCSLKTSMSGYIPSY
Bos_taurus MEQKPSKVECGSDPEESSTRSPDGKRKRKNGQCSLKTSMSGYIPSY
Sus_scrofa MEQKPSKVECGSDPEENSARSPDGKRKRKNGQCSLKTSMSGYIPSY
Orcinus_orca MEQKPSKVECGSDPEENSARSPDGKRKRKNGQCSLKTSMSGYIPSY
50 60 70 80 90
Identity
Fukomys_anselli LDKDEQCVVCGDRATGYHYRCITCEGCKGFFRRTIQKNLHPTYSCK
Mus_musculus LDKDEQCVVCGDKATGYHYRCITCEGCKGFFRRTIQKNLHPTYSCK
Rattus_norvegicus LDKDEQCVVCGDKATGYHYRCITCEGCKGFFRRTIQKNLHPTYSCK
Mesocricetus_auratus LDKDEQCVVCGDKATGYHYRCITCEGCKGFFRRTIQKNLHPTYSCK
Cricetulus_griseus LDKDEQCVVCGDKATGYHYRCITCEGCKGFFRRTIQKNLHPTYSCK
Oryctolagus_cuniculus LDKDEQCVVCGDKATGYHYRCITCEGCKGFFRRTIQKNLHPTYSCK
Ochotona_princeps LDKDEQCVVCGDKATGYHYRCITCEGCKGFFRRTIQKNLHPTYSCK
Otolemur_garnettii LDKDEQCVVCGDKATGYHYRCITCEGCKGFFRRTIQKNLHPTYSCK
Macaca_fascicularis LDKDEQCVVCGDKATGYHYRCITCEGCKGFFRRTIQKNLHPTYSCK
Homo_sapiens LDKDEQCVVCGDKATGYHYRCITCEGCKGFFRRTIQKNLHPTYSCK
Felis_catus LDKDEQCVVCGDKATGYHYRCITCEGCKGFFRRTIQKNLHPTYSCK
Ailuropoda_melanoleuca LDKDEQCVVCGDKATGYHYRCITCEGCKGFFRRTIQKNLHPTYSCK
Bos_taurus LDKDEQCVVCGDKATGYHYRCITCEGCKGFFRRTIQKNLHPTYSCK
Sus_scrofa LDKDEQCVVCGDKATGYHYRCITCEGCKGFFRRTIQKNLHPTYSCK
Orcinus_orca LDKDEQCVVCGDKATGYHYRCITCEGCKGFFRRTIQKNLHPTYSCK
100 110 120 130
Identity
Fukomys_anselli YDSCCVIDKITRNQCQLCRFKKCIGVGMAMDLVLDDSKRVAKRKLI
Mus_musculus YDSCCVIDKITRNQCQLCRFKKCIAVGMAMDLVLDDSKRVAKRKLI
Rattus_norvegicus YDSCCVIDKITRNQCQLCRFKKCIAVGMAMDLVLDDSKRVAKRKLI
Mesocricetus_auratus YDSCCVIDKITRNQCQLCRFKKCIAVGMAMDLVLDDSKRVAKRKLI
Cricetulus_griseus YDSCCVIDKITRNQCQLCRFKKCIAVGMAMDLVLDDSKRVAKRKLI
Oryctolagus_cuniculus YDSCCVIDKITRNQCQLCRFKKCIAVGMAMDLVLDDSKRVAKRKLI
Ochotona_princeps YDSCCVIDKITRNQCQLCRFKKCIAVGMAMDLVLDDSKRVAKRKLI
Otolemur_garnettii YDSCCVIDKITRNQCQLCRFKKCIAVGMAMDLVLDDSKRVAKRKLI
Macaca_fascicularis YDSCCVIDKITRNQCQLCRFKKCIAVGMAMDLVLDDSKRVAKRKLI
Homo_sapiens YDSCCVIDKITRNQCQLCRFKKCIAVGMAMDLVLDDSKRVAKRKLI
Felis_catus YDSCCVIDKITRNQCQLCRFKKCIAVGMAMDLVLDDSKRVAKRKLI
Ailuropoda_melanoleuca YDSCCVIDKITRNQCQLCRFKKCIAVGMAMDLVLDDSKRVAKRKLI
Bos_taurus YDSCCVIDKITRNQCQLCRFKKCIAVGMAMDLVLDDSKRVAKRKLI
Sus_scrofa YDSCCVIDKITRNQCQLCRFKKCIAVGMAMDLVLDDSKRVAKRKLI
Orcinus_orca YDSCCVIDKITRNQCQLCRFKKCIAVGMAMDLVLDDSKRVAKRKLI
140 150 160 170 180
Identity
Fukomys_anselli EQNRERRRKEEMIRSLQQRPEPTPEEWDLIHVATEAHRSTNAQGSH
Mus_musculus EQNRERRRKEEMIRSLQQRPEPTPEEWDLIHVATEAHRSTNAQGSH
Rattus_norvegicus EQNRERRRKEEMIRSLQQRPEPTPEEWDLIHVATEAHRSTNAQGSH
Mesocricetus_auratus EQNRERRRKEEMIRSLQQRPEPTPEEWDLIHVATEAHRSTNAQGSH
Cricetulus_griseus EQNRERRRKEEMIRSLQQRPEPTPEEWDLIHVATEAHRSTNAQGSH
Oryctolagus_cuniculus EQNRERRRKEEMIRSLQQRPEPTPEEWDLIHVATEAHRSTNAQGSH
Ochotona_princeps EQNRERRRKEEMIRSLQQRPEPTPEEWALIHVATEAHRSTNAQGSH
Otolemur_garnettii EQNRERRRKEEMIRSLQQRPEPTPEEWDLIHVATEAHRSTNAQGSH
Macaca_fascicularis EQNRERRRKEEMIRSLQQRPEPTPEEWDLIHIATEAHRSTNAQGSH
Homo_sapiens EQNRERRRKEEMIRSLQQRPEPTPEEWDLIHIATEAHRSTNAQGSH
Felis_catus EQNRERRRKEEMIRSLQQRPEPTPEEWDLIHVATEAHRSTNAQGSH
Ailuropoda_melanoleuca EQNRERRRKEEMIRSLQQRPEPTPEEWDLIHVATEAHRSTNAQGSH
Bos_taurus EQNRERRRKEEMIRSLQQRPEPTPEEWDLIHVATEAHRSTNAQGSH
Sus_scrofa EQNRERRRKEEMIRSLQQRPEPTPEEWDLIHVATEAHRSTNAQGSH
Orcinus_orca EQNRERRRKEEMIRSLQQRPEPTPEEWDLIHVATEAHRSTNAQGSH
190 200 210 220
Identity
Fukomys_anselli WKQRRKFLPDDIGQSPIVSMPDGDKVDLEAFSEFTKIITPAITRVV
Mus_musculus WKQRRKFLPDDIGQSPIVSMPDGDKVDLEAFSEFTKIITPAITRVV
Rattus_norvegicus WKQRRKFLPDDIGQSPIVSMPDGDKVDLEAFSEFTKIITPAITRVV
Mesocricetus_auratus WKQRRKFLPDDIGQSPIVSMPDGDKVDLEAFSEFTKIITPAITRVV
Cricetulus_griseus WKQRRKFLPDDIGQSPIVSMPDGDKVDLEAFSEFTKIITPAITRVV
Oryctolagus_cuniculus WKQRRKFLPDDIGQSPIVSMPDGDKVDLEAFSEFTKIITPAITRVV
Ochotona_princeps WKQRRKFLPDDIGQSPIVSMPDGDKVDLEAFSEFTKIITPAITRVV
Otolemur_garnettii WKQRRKFLPDDIGQSPIVSMPDGDKVDLEAFSEFTKIITPAITRVV
Macaca_fascicularis WKQRRKFLPDDIGQSPIVSMPDGDKVDLEAFSEFTKIITPAITRVV
Homo_sapiens WKQRRKFLPDDIGQSPIVSMPDGDKVDLEAFSEFTKIITPAITRVV
Felis_catus WKQRRKFLPDDIGQSPIVSMPDGDKVDLEAFSEFTKIITPAITRVV
Ailuropoda_melanoleuca WKQRRKFLPDDIGQSPIVSMPDGDKVDLEAFSEFTKIITPAITRVV
Bos_taurus WKQRRKFLPDDIGQSPIVSMPDGDKVDLEAFSEFTKIITPAITRVV
Sus_scrofa WKQRRKFLPDDIGQSPIVSMPDGDKVDLEAFSEFTKIITPAITRVV
Orcinus_orca WKQRRKFLPDDIGQSPIVSMPDGDKVDLEAFSEFTKIITPAITRVV

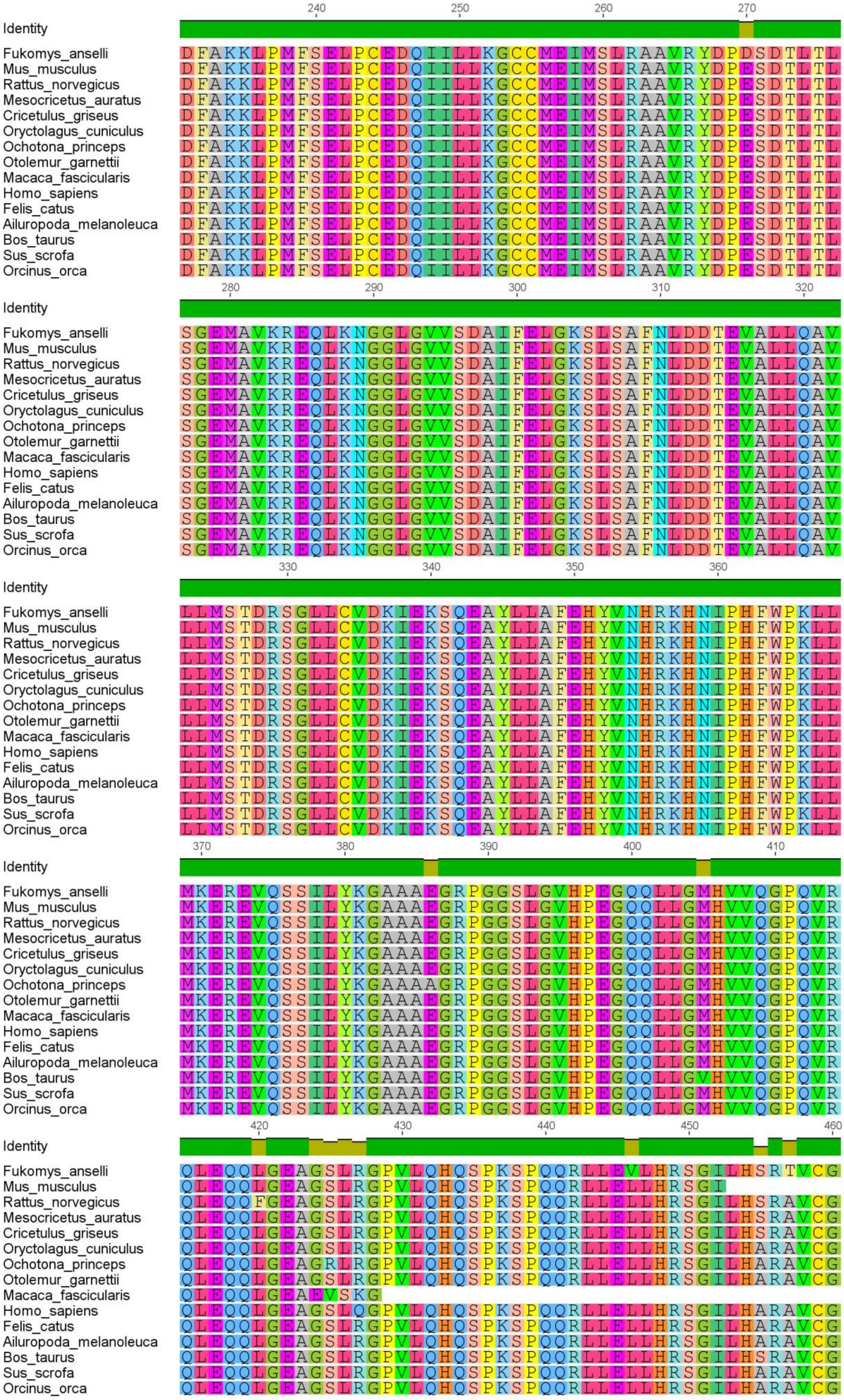
240 250 260 270
Identity
Fukomys_anselli DFAKKLPMFSELPCEDQIILLKGCCMEIMSLRAAVRYDPDSDTLTL
Mus_musculus DFAKKLPMFSELPCEDQIILLKGCCMEIMSLRAAVRYDPESDTLTL
Rattus_norvegicus DFAKKLPMFSELPCEDQIILLKGCCMEIMSLRAAVRYDPESDTLTL
Mesocricetus_auratus DFAKKLPMFSELPCEDQIILLKGCCMEIMSLRAAVRYDPESDTLTL
Cricetulus_griseus DFAKKLPMFSELPCEDQIILLKGCCMEIMSLRAAVRYDPESDTLTL
Oryctolagus_cuniculus DFAKKLPMFSELPCEDQIILLKGCCMEIMSLRAAVRYDPESDTLTL
Ochotona_princeps DFAKKLPMFSELPCEDQIILLKGCCMEIMSLRAAVRYDPESDTLTL
Otolemur_garnettii DFAKKLPMFSELPCEDQIILLKGCCMEIMSLRAAVRYDPESDTLTL
Macaca_fascicularis DFAKKLPMFSELPCEDQIILLKGCCMEIMSLRAAVRYDPESDTLTL
Homo_sapiens DFAKKLPMFSELPCEDQIILLKGCCMEIMSLRAAVRYDPESDTLTL
Felis_catus DFAKKLPMFSELPCEDQIILLKGCCMEIMSLRAAVRYDPESDTLTL
Ailuropoda_melanoleuca DFAKKLPMFSELPCEDQIILLKGCCMEIMSLRAAVRYDPESDTLTL
Bos_taurus DFAKKLPMFSELPCEDQIILLKGCCMEIMSLRAAVRYDPESDTLTL
Sus_scrofa DFAKKLPMFSELPCEDQIILLKGCCMEIMSLRAAVRYDPESDTLTL
Orcinus_orca DFAKKLPMFSELPCEDQIILLKGCCMEIMSLRAAVRYDPESDTLTL
280 290 300 310 320
Identity
Fukomys_anselli SGEMAVKREQLKNGGLGVVSDAIFELGKSLSAFNLDDTEVALLQAV
Mus_musculus SGEMAVKREQLKNGGLGVVSDAIFELGKSLSAFNLDDTEVALLQAV
Rattus_norvegicus SGEMAVKREQLKNGGLGVVSDAIFELGKSLSAFNLDDTEVALLQAV
Mesocricetus_auratus SGEMAVKREQLKNGGLGVVSDAIFELGKSLSAFNLDDTEVALLQAV
Cricetulus_griseus SGEMAVKREQLKNGGLGVVSDAIFELGKSLSAFNLDDTEVALLQAV
Oryctolagus_cuniculus SGEMAVKREQLKNGGLGVVSDAIFELGKSLSAFNLDDTEVALLQAV
Ochotona_princeps SGEMAVKREQLKNGGLGVVSDAIFELGKSLSAFNLDDTEVALLQAV
Otolemur_garnettii SGEMAVKREQLKNGGLGVVSDAIFELGKSLSAFNLDDTEVALLQAV
Macaca_fascicularis SGEMAVKREQLKNGGLGVVSDAIFELGKSLSAFNLDDTEVALLQAV
Homo_sapiens SGEMAVKREQLKNGGLGVVSDAIFELGKSLSAFNLDDTEVALLQAV
Felis_catus SGEMAVKREQLKNGGLGVVSDAIFELGKSLSAFNLDDTEVALLQAV
Ailuropoda_melanoleuca SGEMAVKREQLKNGGLGVVSDAIFELGKSLSAFNLDDTEVALLQAV
Bos_taurus SGEMAVKREQLKNGGLGVVSDAIFELGKSLSAFNLDDTEVALLQAV
Sus_scrofa SGEMAVKREQLKNGGLGVVSDAIFELGKSLSAFNLDDTEVALLQAV
Orcinus_orca SGEMAVKREQLKNGGLGVVSDAIFELGKSLSAFNLDDTEVALLQAV
330 340 350 360
Identity
Fukomys_anselli LLMSTDRSGLLCVDKIEKSQEAYLLAFEHYVNHRKHNIPHFWPKLL
Mus_musculus LLMSTDRSGLLCVDKIEKSQEAYLLAFEHYVNHRKHNIPHFWPKLL
Rattus_norvegicus LLMSTDRSGLLCVDKIEKSQEAYLLAFEHYVNHRKHNIPHFWPKLL
Mesocricetus_auratus LLMSTDRSGLLCVDKIEKSQEAYLLAFEHYVNHRKHNIPHFWPKLL
Cricetulus_griseus LLMSTDRSGLLCVDKIEKSQEAYLLAFEHYVNHRKHNIPHFWPKLL
Oryctolagus_cuniculus LLMSTDRSGLLCVDKIEKSQEAYLLAFEHYVNHRKHNIPHFWPKLL
Ochotona_princeps LLMSTDRSGLLCVDKIEKSQEAYLLAFEHYVNHRKHNIPHFWPKLL
Otolemur_garnettii LLMSTDRSGLLCVDKIEKSQEAYLLAFEHYVNHRKHNIPHFWPKLL
Macaca_fascicularis LLMSTDRSGLLCVDKIEKSQEAYLLAFEHYVNHRKHNIPHFWPKLL
Homo_sapiens LLMSTDRSGLLCVDKIEKSQEAYLLAFEHYVNHRKHNIPHFWPKLL
Felis_catus LLMSTDRSGLLCVDKIEKSQEAYLLAFEHYVNHRKHNIPHFWPKLL
Ailuropoda_melanoleuca LLMSTDRSGLLCVDKIEKSQEAYLLAFEHYVNHRKHNIPHFWPKLL
Bos_taurus LLMSTDRSGLLCVDKIEKSQEAYLLAFEHYVNHRKHNIPHFWPKLL
Sus_scrofa LLMSTDRSGLLCVDKIEKSQEAYLLAFEHYVNHRKHNIPHFWPKLL
Orcinus_orca LLMSTDRSGLLCVDKIEKSQEAYLLAFEHYVNHRKHNIPHFWPKLL
370 380 390 400 410
Identity
Fukomys_anselli MKEREVQSSILYKGAAAEGRPGGSLGVHPEGQQLLGMHVVQGPQVR
Mus_musculus MKEREVQSSILYKGAAAEGRPGGSLGVHPEGQQLLGMHVVQGPQVR
Rattus_norvegicus MKEREVQSSILYKGAAAEGRPGGSLGVHPEGQQLLGMHVVQGPQVR
Mesocricetus_auratus MKEREVQSSILYKGAAAEGRPGGSLGVHPEGQQLLGMHVVQGPQVR
Cricetulus_griseus MKEREVQSSILYKGAAAEGRPGGSLGVHPEGQQLLGMHVVQGPQVR
Oryctolagus_cuniculus MKEREVQSSILYKGAAAEGRPGGSLGVHPEGQQLLGMHVVQGPQVR
Ochotona_princeps MKEREVQSSILYKGAAAAGRPGGSLGVHPEGQQLLGMHVVQGPQVR
Otolemur_garnettii MKEREVQSSILYKGAAAEGRPGGSLGVHPEGQQLLGMHVVQGPQVR
Macaca_fascicularis MKEREVQSSILYKGAAAEGRPGGSLGVHPEGQQLLGMHVVQGPQVR
Homo_sapiens MKEREVQSSILYKGAAAEGRPGGSLGVHPEGQQLLGMHVVQGPQVR
Felis_catus MKEREVQSSILYKGAAAEGRPGGSLGVHPEGQQLLGMHVVQGPQVR
Ailuropoda_melanoleuca MKEREVQSSILYKGAAAEGRPGGSLGVHPEGQQLLGMHVVQGPQVR
Bos_taurus MKEREVQSSILYKGAAAEGRPGGSLGVHPEGQQLLGVHVVQGPQVR
Sus_scrofa MKEREVQSSILYKGAAAEGRPGGSLGVHPEGQQLLGMHVVQGPQVR
Orcinus_orca MKEREVQSSILYKGAAAEGRPGGSLGVHPEGQQLLGMHVVQGPQVR
420 430 440 450 460
Identity
Fukomys_anselli QLEQQLGEAGSLRGPVLQHQSPKSPQQRLLEVLHRSGILHSRTVCG
Mus_musculus QLEQQLGEAGSLRGPVLQHQSPKSPQQRLLELLHRSGI
Rattus_norvegicus QLEQQFGEAGSLRGPVLQHQSPKSPQQRLLELLHRSGILHSRAVCG
Mesocricetus_auratus QLEQQLGEAGSLRGPVLQHQSPKSPQQRLLELLHRSGILHSRAVCG
Cricetulus_griseus QLEQQLGEAGSLRGPVLQHQSPKSPQQRLLELLHRSGILHSRAVCG
Oryctolagus_cuniculus QLEQQLGEAGSLRGPVLQHQSPKSPQQRLLELLHRSGILHARAVCG
Ochotona_princeps QLEQQLGEAGRLRGPVLQHQSPKSPQQRLLELLHRSGILHARAVCG
Otolemur_garnettii QLEQQLGEAGSLRGPVLQHQSPKSPQQRLLELLHRSGILHARAVCG
Macaca_fascicularis QLEQQLGEAEVSKG
Homo_sapiens QLEQQLGEAGSLQGPVLQHQSPKSPQQRLLELLHRSGILHARAVCG
Felis_catus QLEQQLGEAGSLRGPVLQHQSPKSPQQRLLELLHRSGILHARAVCG
Ailuropoda_melanoleuca QLEQQLGEAGSLRGPVLQHQSPKSPQQRLLELLHRSGILHARAVCG
Bos_taurus QLEQQLGEAGSLRGPVLQHQSPKSPQQRLLELLHRSGILHSRAVCG
Sus_scrofa QLEQQLGEAGSLRGPVLQHQSPKSPQQRLLELLHRSGILHARAVCG
Orcinus_orca QLEQQLGEAGSLRGPVLQHQSPKSPQQRLLELLHRSGILHARAVCG

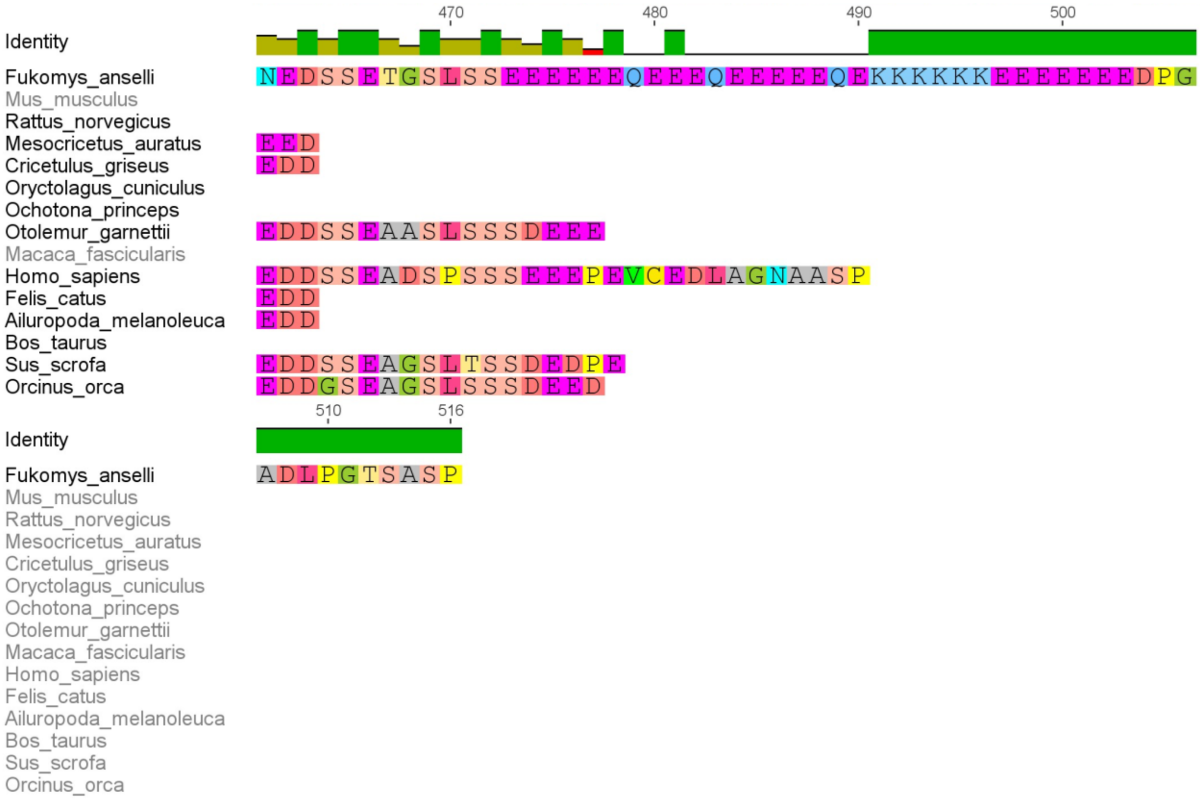

Figure S1. Protein alignment of thyroid hormone receptor α (THRA) from different mammal species. The mRNA sequence of *F. anselli* was obtained from RNA-seq and subsequently translated, other sequences were retrieved from NCBI databases with the following accession numbers: Mus musculus (CAA30576), Rattus norvergicus (NP_112396), Mesocricetus auratus (XP_005076008), Cricetulus_griseus (XP_003510526), Oryctolagus cuniculus (XP_002719397), Ochotona princeps(XP_004591220), Otolemur garnettii (XP_003786450), Macaca fascicularis (NP_001270601),Homo sapiens (NP_003241),Felis catus (XP_003996845), Ailuropoda melanoleuca (XP_002924975), Bos Taurus (NP_001039794), Sus scrofa (O97716), Orcinus orca (XP_004282800).
