# Supplementary material for: Unusual Ratio between Free Thyroxine and Free Triiodothyronine in a Long-Lived Mole-Rat Species with Bimodal Ageing
Source: PLoS One. 2014 Nov 19;9(11):e113698. doi: 10.1371/journal.pone.0113698 (PMC4237498; doi:10.1371/journal.pone.0113698)
Supplement: Figure S2 — Protein alignment of thyroid hormone receptor β (THRB) from different mammal species. The mRNA sequence of F. anselli was obtained from RNA-seq and subsequently translated, other sequences were retrieved from NCBI databases with the following accession numbers: Heterocephalus glaber (XP_004892721), Cavia porcellus (XP_005008354), Chinchilla lanigera (XP_005387539), Octodon degus (XP_004634245), Mus musculus (P37242), Rattus norvegicus (P18113), Mesocricetus auratus (XP_005081037), Cricetulus griseus (ERE87100), Ochotona princeps (XP_004588346), Otolemur garnettii (XP_003781795), Homo sapiens (P10828), Canis lupus (XP_862690), Mustela putorius furo (XP_004786805), Ailuropoda melanoleuca (XP_002928077), Ovis aries (Q28571), Sus scrofa (XP_001928500), Condylura cristata (XP_004692336). (PDF) [file pone.0113698.s002.pdf]

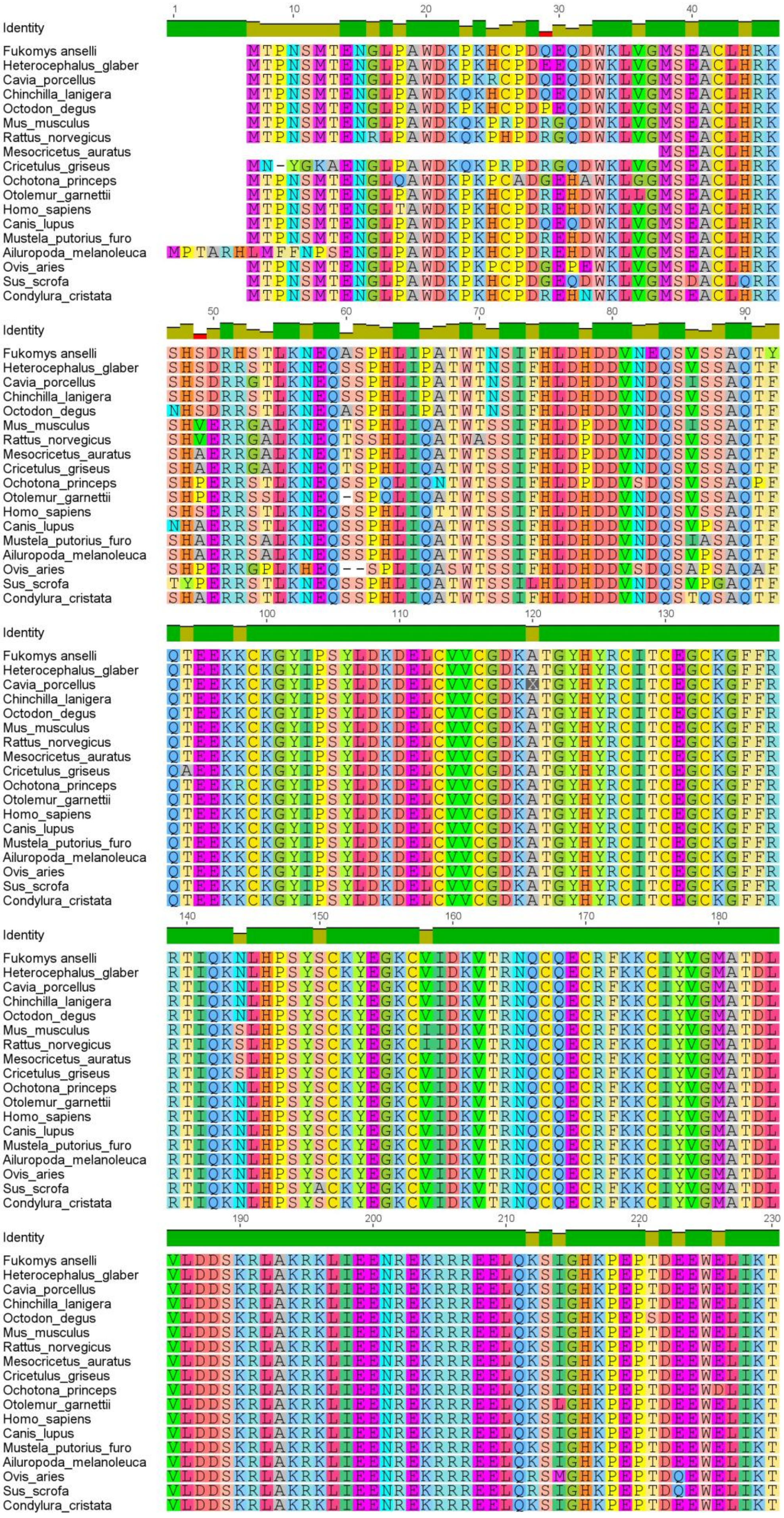

1 10 20 30 40
Identity
Fukomys anselli MTPNSMTENGLPAWDKPKHCPDQEQDWKLVGMSEACLHRK
Heterocephalus_glaber MTPNSMTENGLPAWDKPKHCPDEEQDWKLVGMSEACLHRK
Cavia_porcellus MTPNSMTENGLPAWDKPKRCPDQEQDWKLVGMSEACLHRK
Chinchilla_lanigera MTPNSMTENGLPAWDKQKHCPDQEQDWKLVGMSEACLHRK
Octodon_degus MTPNSMTENGLPAWDKPKHCPDPEQDWKLVGMSEACLHRK
Mus_musculus MTPNSMTENGLPAWDKQKPRPDRGQDWKLVGMSEACLHRK
Rattus_norvegicus MTPNSMTENRLPAWDKQKPHPDRGQDWKLVGMSEACLHRK
Mesocricetus_auratus MSEACLHRK
Cricetulus_griseus MN-YGKAENGLPAWDKQKPRPDRGQDWKLVGMSEACLHRK
Ochotona_princeps MTPNSMTENGLQAWDKPKPCADGEHAWKLGGMSEACLHRK
Otolemur_garnettii MTPNSMTENGLPAWDKPKHCPDREHDWKLLGMSEACLHRK
Homo_sapiens MTPNSMTENGLTAWDKPKHCPDREHDWKLVGMSEACLHRK
Canis_lupus MTPNSMTENGLPAWDKPKHCPDQEQDWKLVGMSEACLHRK
Mustela_putorius_furo MTPNSMTENGLPAWDKPKHCPDREHDWKLVGMSEACLHRK
Ailuropoda_melanoleuca MPTARHLMFFNPSENGLPAWDKPKHCPDREHDWKLVGMSEACLHRK
Ovis_aries MTPNSMTENGLPAWDKPKPCPDGEPEWKLVGMSEACLHRK
Sus_scrofa MTPNSMTENGLPAWDKPKHCPDGEQDWKLVGMSDACLQRK
Condylura_cristata MTPNSMTENGLPAWDKPKHCPDREHNWKLVGMSEACLHRK
50 60 70 80 90
Identity
Fukomys anselli SHSDRHSTLKNEQASPHLIPATWTNSIFHLDHDDVNEQSVSSAQTY
Heterocephalus_glaber SHSDRRSTLKNEQSSPHLIPATWTNSIFHLDHDDVNDQSVSSAQTF
Cavia_porcellus SHSDRRGTLKNEQSSPHLIPATWTNSIFHLDHDDVNDQSISSAQTF
Chinchilla_lanigera SHSDRRSTLKNEQSSPHLIPATWTNSIFHLDHDDVNDQSVSSAQTF
Octodon_degus NHSDRRSTLKNEQASPHLIPATWTNSIFHLDHDDVNDQSVSSAQTF
Mus_musculus SHVERRGALKNEQTSPHLIQATWTSSIFHLDPDDVNDQSISSAQTF
Rattus_norvegicus SHVERRGALKNEQTSSHLIQATWASSIFHLDPDDVNDQSISSAQTF
Mesocricetus_auratus SHAERRGALKNEQTSPHLIQATWTSSIFHLDPDDVNDQSVSSAQTF
Cricetulus_griseus SHAERRGALKNEQTSPHLIQATWTSSIFHLDPDDVNDQSVSSAQTF
Ochotona_princeps SHPERRSTLKNEQSSPQLIQNTWTSSIFHLDPDDVSDQSVSSAQPF
Otolemur_garnettii SHPERRSSLKNEQ-SPQLIQATWTSSIFHLDHDDVNDQSVSSAQTF
Homo_sapiens SHSERRSTLKNEQSSPHLIQTTWTSSIFHLDHDDVNDQSVSSAQTF
Canis_lupus NHAERRSTLKNEQSSPHLIQATWTSSIFHLDHDDVNDQSVPSAQTF
Mustela_putorius_furo SHAERRSALKNEQSSPHLIQATWTSSIFHLDHDDVNDQSIASAQTF
Ailuropoda_melanoleuca SHAERRSALKNEQSSPHLIQATWTSSIFHLDHDDVNDQSVPSAQTF
Ovis_aries SHPERRGPLKHEQ--SPLIQASWTSSIFHLDHDDVSDQSAPSAQAF
Sus_scrofa TYPERRSTLKNEQSSPHLIQATWTSSILHLDHDDVNDQSVPGAQTF
Condylura_cristata SHAERRSTLKNEQSSPHLIQATWTSSIFHLDHDDVNDQSTQSAQTF
100 110 120 130
Identity
Fukomys anselli QTEEKKCKGYIPSYLDKDELCVVCGDKATGYHYRCITCEGCKGFFR
Heterocephalus_glaber QTEEKKCKGYIPSYLDKDELCVVCGDKATGYHYRCITCEGCKGFFR
Cavia_porcellus QTEEKKCKGYIPSYLDKDELCVVCGDKXTGYHYRCITCEGCKGFFR
Chinchilla_lanigera QTEEKKCKGYIPSYLDKDELCVVCGDKATGYHYRCITCEGCKGFFR
Octodon_degus QTEEKKCKGYIPSYLDKDELCVVCGDKATGYHYRCITCEGCKGFFR
Mus_musculus QTEEKKCKGYIPSYLDKDELCVVCGDKATGYHYRCITCEGCKGFFR
Rattus_norvegicus QTEEKKCKGYIPSYLDKDELCVVCGDKATGYHYRCITCEGCKGFFR
Mesocricetus_auratus QTEEKKCKGYIPSYLDKDELCVVCGDKATGYHYRCITCEGCKGFFR
Cricetulus_griseus QAEEKKCKGYIPSYLDKDELCVVCGDKATGYHYRCITCEGCKGFFR
Ochotona_princeps QTEEKRCKGYIPSYLDKDELCVVCGDKATGYHYRCITCEGCKGFFR
Otolemur_garnettii QTEEKKCKGYIPSYLDKDELCVVCGDKATGYHYRCITCEGCKGFFR
Homo_sapiens QTEEKKCKGYIPSYLDKDELCVVCGDKATGYHYRCITCEGCKGFFR
Canis_lupus QTEEKKCKGYIPSYLDKDELCVVCGDKATGYHYRCITCEGCKGFFR
Mustela_putorius_furo QTEEKKCKGYIPSYLDKDELCVVCGDKATGYHYRCITCEGCKGFFR
Ailuropoda_melanoleuca QTEEKKCKGYIPSYLDKDELCVVCGDKATGYHYRCITCEGCKGFFR
Ovis_aries QTEEKKCKGYIPSYLDKDELCVVCGDKATGYHYRCITCEGCKGFFR
Sus_scrofa QTEEKKCKGYIPSYLDKDELCVVCGDKATGYHYRCITCEGCKGFFR
Condylura_cristata QTEEKKCKGYIPSYLDKDELCVVCGDKATGYHYRCITCEGCKGFFR
140 150 160 170 180
Identity
Fukomys anselli RTIQKNLHPSYSCKYEGKCVIDKVTRNQCQECRFKKCIYVGMATDL
Heterocephalus_glaber RTIQKNLHPSYSCKYEGKCVIDKVTRNQCQECRFKKCIYVGMATDL
Cavia_porcellus RTIQKNLHPSYSCKYEGKCVIDKVTRNQCQECRFKKCIYVGMATDL
Chinchilla_lanigera RTIQKNLHPSYSCKYEGKCVIDKVTRNQCQECRFKKCIYVGMATDL
Octodon_degus RTIQKNLHPSYSCKYEGKCVIDKVTRNQCQECRFKKCIYVGMATDL
Mus_musculus RTIQKSLHPSYSCKYEGKCIIDKVTRNQCQECRFKKCIYVGMATDL
Rattus_norvegicus RTIQKSLHPSYSCKYEGKCIIDKVTRNQCQECRFKKCIYVGMATDL
Mesocricetus_auratus RTIQKSLHPSYSCKYEGKCVIDKVTRNQCQECRFKKCIYVGMATDL
Cricetulus_griseus RTIQKSLHPSYSCKYEGKCVIDKVTRNQCQECRFKKCIYVGMATDL
Ochotona_princeps RTIQKNLHPSYSCKYEGKCVIDKVTRNQCQECRFKKCIYVGMATDL
Otolemur_garnettii RTIQKNLHPSYSCKYEGKCVIDKVTRNQCQECRFKKCIYVGMATDL
Homo_sapiens RTIQKNLHPSYSCKYEGKCVIDKVTRNQCQECRFKKCIYVGMATDL
Canis_lupus RTIQKNLHPSYSCKYEGKCVIDKVTRNQCQECRFKKCIYVGMATDL
Mustela_putorius_furo RTIQKNLHPSYSCKYEGKCVIDKVTRNQCQECRFKKCIYVGMATDL
Ailuropoda_melanoleuca RTIQKNLHPSYSCKYEGKCVIDKVTRNQCQECRFKKCIYVGMATDL
Ovis_aries RTIQKNLHPSYSCKYEGKCVIDKVTRNQCQECRFKKCIYVGMATDL
Sus_scrofa RTIQKNLHPSYACKYEGKCVIDKVTRNQCQECRFKKCIYVGMATDL
Condylura_cristata RTIQKNLHPSYSCKYEGKCVIDKVTRNQCQECRFKKCIYVGMATDL
190 200 210 220 230
Identity
Fukomys anselli VLDDSKRLAKRKLIEENREKRRREELQKSIGHKPEPTDEEWELIKT
Heterocephalus_glaber VLDDSKRLAKRKLIEENREKRRREELQKSIGHKPEPTDEEWELIKT
Cavia_porcellus VLDDSKRLAKRKLIEENREKRRREELQKSIGHKPEPTDEEWELIKT
Chinchilla_lanigera VLDDSKRLAKRKLIEENREKRRREELQKSIGHKPEPTDEEWELIKT
Octodon_degus VLDDSKRLAKRKLIEENREKRRREELQKSIGHKPEPSDEEWELIKT
Mus_musculus VLDDSKRLAKRKLIEENREKRRREELQKSIGHKPEPTDEEWELIKT
Rattus_norvegicus VLDDSKRLAKRKLIEENREKRRREELQKSIGHKPEPTDEEWELIKT
Mesocricetus_auratus VLDDSKRLAKRKLIEENREKRRREELQKSIGHKPEPTDEEWELIKT
Cricetulus_griseus VLDDSKRLAKRKLIEENREKRRREELQKSIGHKPEPTDEEWELIKT
Ochotona_princeps VLDDSKRLAKRKLIEENREKRRREELQKSIGHKPEPTDEEWDLIKT
Otolemur_garnettii VLDDSKRLAKRKLIEENREKRRREELQKSLGHKPEPTDEEWELIKT
Homo_sapiens VLDDSKRLAKRKLIEENREKRRREELQKSIGHKPEPTDEEWELIKT
Canis_lupus VLDDSKRLAKRKLIEENREKRRREELQKSIGHKPEPTDEEWELIKT
Mustela_putorius_furo VLDDSKRLAKRKLIEENREKRRREELQKSIGHKPEPTDEEWELIKT
Ailuropoda_melanoleuca VLDDSKRLAKRKLIEENREKRRREELQKSIGHKPEPTDEEWELIKT
Ovis_aries VLDDSKRLAKRKLIEENREKRRREELQRSMGHKPEPTDQEWELIKT
Sus_scrofa VLDDSKRLAKRKLIEENREKRRREELQRSIGHKPEPTDQEWELIKT
Condylura_cristata VLDDSKRLAKRKLIEENREKRRREELQKSIGHKPEPTDEEWELIKT

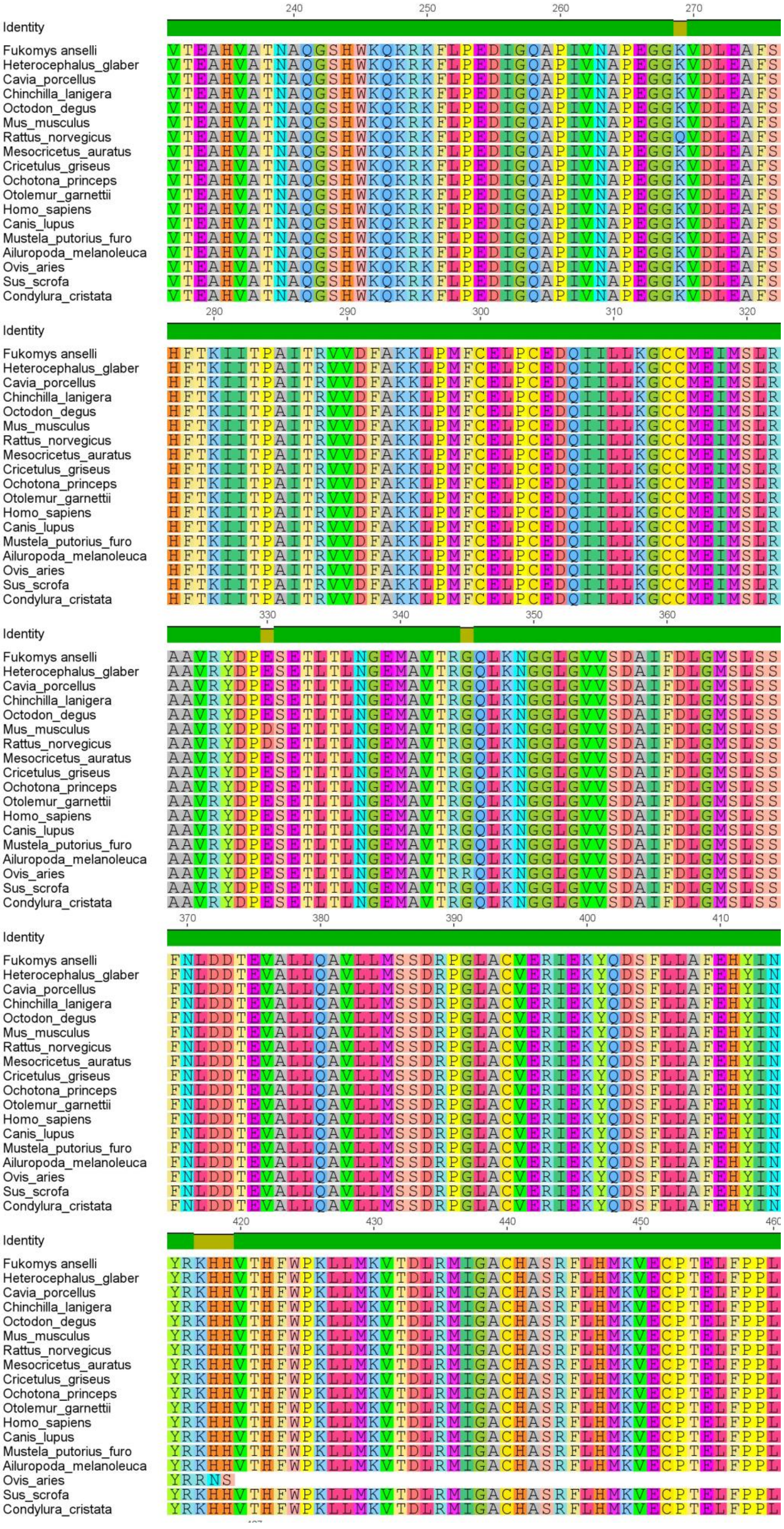

240 250 260 270
Identity
Fukomys anselli VTEAHVATNAQGSHWKQKRKFLPEDIGQAPIVNAPEGGKVDLEAFS
Heterocephalus_glaber VTEAHVATNAQGSHWKQKRKFLPEDIGQAPIVNAPEGGKVDLEAFS
Cavia_porcellus VTEAHVATNAQGSHWKQKRKFLPEDIGQAPIVNAPEGGKVDLEAFS
Chinchilla_lanigera VTEAHVATNAQGSHWKQKRKFLPEDIGQAPIVNAPEGGKVDLEAFS
Octodon_degus VTEAHVATNAQGSHWKQKRKFLPEDIGQAPIVNAPEGGKVDLEAFS
Mus_musculus VTEAHVATNAQGSHWKQKRKFLPEDIGQAPIVNAPEGGKVDLEAFS
Rattus_norvegicus VTEAHVATNAQGSHWKQKRKFLPEDIGQAPIVNAPEGGQVDLEAFS
Mesocricetus_auratus VTEAHVATNAQGSHWKQKRKFLPEDIGQAPIVNAPEGGKVDLEAFS
Cricetulus_griseus VTEAHVATNAQGSHWKQKRKFLPEDIGQAPIVNAPEGGKVDLEAFS
Ochotona_princeps VTEAHVATNAQGSHWKQKRKFLPEDIGQAPIVNAPEGGKVDLEAFS
Otolemur_garnettii VTEAHVATNAQGSHWKQKRKFLPEDIGQAPIVNAPEGGKVDLEAFS
Homo_sapiens VTEAHVATNAQGSHWKQKRKFLPEDIGQAPIVNAPEGGKVDLEAFS
Canis_lupus VTEAHVATNAQGSHWKQKRKFLPEDIGQAPIVNAPEGGKVDLEAFS
Mustela_putorius_furo VTEAHVATNAQGSHWKQKRKFLPEDIGQAPIVNAPEGGKVDLEAFS
Ailuropoda_melanoleuca VTEAHVATNAQGSHWKQKRKFLPEDIGQAPIVNAPEGGKVDLEAFS
Ovis_aries VTEAHVATNAQGSHWKQKRKFLPEDIGQAPIVNAPEGGKVDLEAFS
Sus_scrofa VTEAHVATNAQGSHWKQKRKFLPEDIGQAPIVNAPEGGKVDLEAFS
Condylura_cristata VTEAHVATNAQGSHWKQKRKFLPEDIGQAPIVNAPEGGKVDLEAFS
280 290 300 310 320
Identity
Fukomys anselli HFTKIITPAITRVVDFAKKLPMFCELPCEDQIILLKGCCMEIMSLR
Heterocephalus_glaber HFTKIITPAITRVVDFAKKLPMFCELPCEDQIILLKGCCMEIMSLR
Cavia_porcellus HFTKIITPAITRVVDFAKKLPMFCELPCEDQIILLKGCCMEIMSLR
Chinchilla_lanigera HFTKIITPAITRVVDFAKKLPMFCELPCEDQIILLKGCCMEIMSLR
Octodon_degus HFTKIITPAITRVVDFAKKLPMFCELPCEDQIILLKGCCMEIMSLR
Mus_musculus HFTKIITPAITRVVDFAKKLPMFCELPCEDQIILLKGCCMEIMSLR
Rattus_norvegicus HFTKIITPAITRVVDFAKKLPMFCELPCEDQIILLKGCCMEIMSLR
Mesocricetus_auratus HFTKIITPAITRVVDFAKKLPMFCELPCEDQIILLKGCCMEIMSLR
Cricetulus_griseus HFTKIITPAITRVVDFAKKLPMFCELPCEDQIILLKGCCMEIMSLR
Ochotona_princeps HFTKIITPAITRVVDFAKKLPMFCELPCEDQIILLKGCCMEIMSLR
Otolemur_garnettii HFTKIITPAITRVVDFAKKLPMFCELPCEDQIILLKGCCMEIMSLR
Homo_sapiens HFTKIITPAITRVVDFAKKLPMFCELPCEDQIILLKGCCMEIMSLR
Canis_lupus HFTKIITPAITRVVDFAKKLPMFCELPCEDQIILLKGCCMEIMSLR
Mustela_putorius_furo HFTKIITPAITRVVDFAKKLPMFCELPCEDQIILLKGCCMEIMSLR
Ailuropoda_melanoleuca HFTKIITPAITRVVDFAKKLPMFCELPCEDQIILLKGCCMEIMSLR
Ovis_aries HFTKIITPAITRVVDFAKKLPMFCELPCEDQIILLKGCCMEIMSLR
Sus_scrofa HFTKIITPAITRVVDFAKKLPMFCELPCEDQIILLKGCCMEIMSLR
Condylura_cristata HFTKIITPAITRVVDFAKKLPMFCELPCEDQIILLKGCCMEIMSLR
330 340 350 360
Identity
Fukomys anselli AAVRYDPESETLTLNGEMAVTRGQLKNGGLGVVSDAIFDLGMSLSS
Heterocephalus_glaber AAVRYDPESETLTLNGEMAVTRGQLKNGGLGVVSDAIFDLGMSLSS
Cavia_porcellus AAVRYDPESETLTLNGEMAVTRGQLKNGGLGVVSDAIFDLGMSLSS
Chinchilla_lanigera AAVRYDPESETLTLNGEMAVTRGQLKNGGLGVVSDAIFDLGMSLSS
Octodon_degus AAVRYDPESETLTLNGEMAVTRGQLKNGGLGVVSDAIFDLGMSLSS
Mus_musculus AAVRYDPDSETLTLNGEMAVTRGQLKNGGLGVVSDAIFDLGMSLSS
Rattus_norvegicus AAVRYDPDSETLTLNGEMAVTRGQLKNGGLGVVSDAIFDLGMSLSS
Mesocricetus_auratus AAVRYDPESETLTLNGEMAVTRGQLKNGGLGVVSDAIFDLGMSLSS
Cricetulus_griseus AAVRYDPESETLTLNGEMAVTRGQLKNGGLGVVSDAIFDLGMSLSS
Ochotona_princeps AAVRYDPESETLTLNGEMAVTRGQLKNGGLGVVSDAIFDLGMSLSS
Otolemur_garnettii AAVRYDPESETLTLNGEMAVTRGQLKNGGLGVVSDAIFDLGMSLSS
Homo_sapiens AAVRYDPESETLTLNGEMAVTRGQLKNGGLGVVSDAIFDLGMSLSS
Canis_lupus AAVRYDPESETLTLNGEMAVTRGQLKNGGLGVVSDAIFDLGMSLSS
Mustela_putorius_furo AAVRYDPESETLTLNGEMAVTRGQLKNGGLGVVSDAIFDLGMSLSS
Ailuropoda_melanoleuca AAVRYDPESETLTLNGEMAVTRGQLKNGGLGVVSDAIFDLGMSLSS
Ovis_aries AAVRYDPESETLTLNGEMAVTRRQLKNGGLGVVSDAIFDLGMSLSS
Sus_scrofa AAVRYDPESETLTLNGEMAVTRGQLKNGGLGVVSDAIFDLGMSLSS
Condylura_cristata AAVRYDPESETLTLNGEMAVTRGQLKNGGLGVVSDAIFDLGMSLSS
370 380 390 400 410
Identity
Fukomys anselli FNLDDTEVALLQAVLLMSSDRPGLACVERIEKYQDSFLLAFEHYIN
Heterocephalus_glaber FNLDDTEVALLQAVLLMSSDRPGLACVERIEKYQDSFLLAFEHYIN
Cavia_porcellus FNLDDTEVALLQAVLLMSSDRPGLACVERIEKYQDSFLLAFEHYIN
Chinchilla_lanigera FNLDDTEVALLQAVLLMSSDRPGLACVERIEKYQDSFLLAFEHYIN
Octodon_degus FNLDDTEVALLQAVLLMSSDRPGLACVERIEKYQDSFLLAFEHYIN
Mus_musculus FNLDDTEVALLQAVLLMSSDRPGLACVERIEKYQDSFLLAFEHYIN
Rattus_norvegicus FNLDDTEVALLQAVLLMSSDRPGLACVERIEKYQDSFLLAFEHYIN
Mesocricetus_auratus FNLDDTEVALLQAVLLMSSDRPGLACVERIEKYQDSFLLAFEHYIN
Cricetulus_griseus FNLDDTEVALLQAVLLMSSDRPGLACVERIEKYQDSFLLAFEHYIN
Ochotona_princeps FNLDDTEVALLQAVLLMSSDRPGLACVERIEKYQDSFLLAFEHYIN
Otolemur_garnettii FNLDDTEVALLQAVLLMSSDRPGLACVERIEKYQDSFLLAFEHYIN
Homo_sapiens FNLDDTEVALLQAVLLMSSDRPGLACVERIEKYQDSFLLAFEHYIN
Canis_lupus FNLDDTEVALLQAVLLMSSDRPGLACVERIEKYQDSFLLAFEHYIN
Mustela_putorius_furo FNLDDTEVALLQAVLLMSSDRPGLACVERIEKYQDSFLLAFEHYIN
Ailuropoda_melanoleuca FNLDDTEVALLQAVLLMSSDRPGLACVERIEKYQDSFLLAFEHYIN
Ovis_aries FNLDDTEVALLQAVLLMSSDRPGLACVERIEKYQDSFLLAFEHYIN
Sus_scrofa FNLDDTEVALLQAVLLMSSDRPGLACVERIEKYQDSFLLAFEHYIN
Condylura_cristata FNLDDTEVALLQAVLLMSSDRPGLACVERIEKYQDSFLLAFEHYIN
420 430 440 450 460
Identity
Fukomys anselli YRKHHVTHFWPKLLMKVTDLRMIGACHASRFLHMKVECPTELFPPL
Heterocephalus_glaber YRKHHVTHFWPKLLMKVTDLRMIGACHASRFLHMKVECPTELFPPL
Cavia_porcellus YRKHHVTHFWPKLLMKVTDLRMIGACHASRFLHMKVECPTELFPPL
Chinchilla_lanigera YRKHHVTHFWPKLLMKVTDLRMIGACHASRFLHMKVECPTELFPPL
Octodon_degus YRKHHVTHFWPKLLMKVTDLRMIGACHASRFLHMKVECPTELFPPL
Mus_musculus YRKHHVTHFWPKLLMKVTDLRMIGACHASRFLHMKVECPTELFPPL
Rattus_norvegicus YRKHHVTHFWPKLLMKVTDLRMIGACHASRFLHMKVECPTELFPPL
Mesocricetus_auratus YRKHHVTHFWPKLLMKVTDLRMIGACHASRFLHMKVECPTELFPPL
Cricetulus_griseus YRKHHVTHFWPKLLMKVTDLRMIGACHASRFLHMKVECPTELFPPL
Ochotona_princeps YRKHHVTHFWPKLLMKVTDLRMIGACHASRFLHMKVECPTELFPPL
Otolemur_garnettii YRKHHVTHFWPKLLMKVTDLRMIGACHASRFLHMKVECPTELFPPL
Homo_sapiens YRKHHVTHFWPKLLMKVTDLRMIGACHASRFLHMKVECPTELFPPL
Canis_lupus YRKHHVTHFWPKLLMKVTDLRMIGACHASRFLHMKVECPTELFPPL
Mustela_putorius_furo YRKHHVTHFWPKLLMKVTDLRMIGACHASRFLHMKVECPTELFPPL
Ailuropoda_melanoleuca YRKHHVTHFWPKLLMKVTDLRMIGACHASRFLHMKVECPTELFPPL
Ovis_aries YRRNS
Sus_scrofa YRKHHVTHFWPKLLMKVTDLRMIGACHASRFLHMKVECPTELFPPL
Condylura_cristata YRKHHVTHFWPKLLMKVTDLRMIGACHASRFLHMKVECPTELFPPL

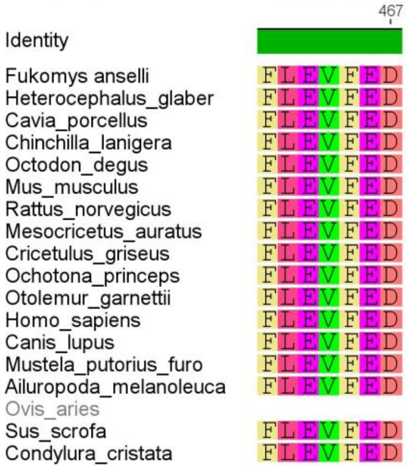

Figure S2. Protein alignment of thyroid hormone receptor β (THRB) from different mammal species. The mRNA sequence of *F. anselli* was obtained from RNA-seq and subsequently translated, other sequences were retrieved from NCBI databases with the following accession numbers: Heterocephalus glaber (XP_004892721), Cavia porcellus (XP_005008354), Chinchilla lanigera (XP_005387539), Octodon degus (XP_004634245), Mus musculus (P37242), Rattus norvegicus (P18113), Mesocricetus auratus (XP_005081037), Cricetulus griseus (ERE87100), Ochotona princeps (XP_004588346), Otolemur garnettii (XP_003781795), Homo sapiens (P10828), Canis lupus (XP_862690), Mustela putorius furo (XP_004786805), Ailuropoda melanoleuca (XP_002928077), Ovis aries (Q28571), Sus scrofa (XP_001928500), Condylura cristata (XP_004692336).
