# Supplementary material for: Unusual Ratio between Free Thyroxine and Free Triiodothyronine in a Long-Lived Mole-Rat Species with Bimodal Ageing
Source: PLoS One. 2014 Nov 19;9(11):e113698. doi: 10.1371/journal.pone.0113698 (PMC4237498; doi:10.1371/journal.pone.0113698)
Supplement: Figure S3 — Protein alignment of thyreotropin β subunit (TSHB) from different mammal species. The mRNA sequence of F. anselli was obtained from RNA-seq and subsequently translated, other sequences were retrieved from NCBI databases with the following accession numbers: Cavia porcellus (XP_003479306), Octodon degus (XP_004641725), Mus musculus (NP_001159412), Rattus norvegicus (NP_037248), Ictidomys tridecemlineatus (XP_005334966), Otolemur garnettii (XP_003793878), Macaca fascicularis (XP_001111873), Nomascus leucogenys (XP_003268073), Gorilla gorilla (XP_004026450), Pan paniscus (XP_003805682), Pan troglodytes (XP_001160337), Homo sapiens (AAB30828), Bos taurus (XP_005204060), Orcinus orca (XP_004263279), Echinops telfairi (XP_004714911). (PDF) [file pone.0113698.s003.pdf]

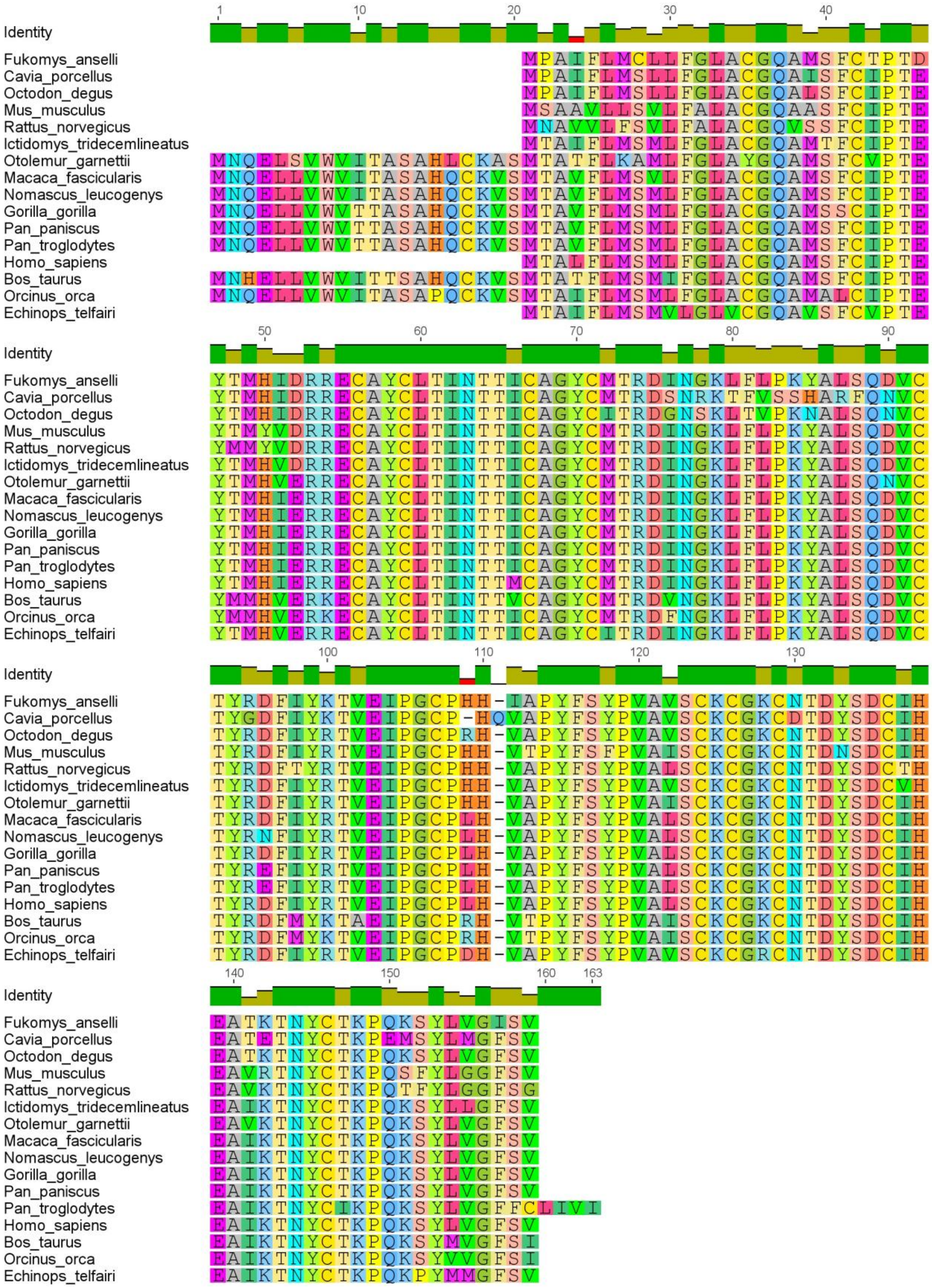
1 10 20 30 40
Identity
Fukomys_anselli MPAIFLMCLLFGLACGQAMSFCTPTD
Cavia_porcellus MPAIFLMSLLFGLACGQAISFCIPTE
Octodon_degus MPAIFLMSLLFGLACGQALSFCIPTE
Mus_musculus MSAAVLLSVLFALACGQAASFCIPTE
Rattus_norvegicus MNAVVLFSVLFALACGQVSSFCIPTE
Ictidomys_tridecemlineatus MTAIFLMSMLFGLACGQAMTFCIPTE
Otolemur_garnettii MNQELSVWVITASAHLCKASMTATFLKAMLFGLAYGQAMSFCVPTE
Macaca_fascicularis MNQELLVWVITASAHQCKVSMTAVFLMSVLFGLACGQAMSFCIPTE
Nomascus_leucogenys MNQELLVWVITASAHQCKVSMTAVFLMSMLFGLACGQAMSFCIPTE
Gorilla_gorilla MNQELLVWVTTASAHQCKVSMTAVFLMSMLFGLACGQAMSSCIPTE
Pan_paniscus MNQELLVWVTTASAHQCKVSMTAVFLMSMLFGLACGQAMSFCIPTE
Pan_troglodytes MNQELLVWVTTASAHQCKVSMTAVFLMSMLFGLACGQAMSFCIPTE
Homo_sapiens MTALFLMSMLFGLACGQAMSFCIPTE
Bos_taurus MNHELLVWVITTSAHQCKVSMTATFLMSMIFGLACGQAMSFCIPTE
Orcinus_orca MNQELLVWVITASAPQCKVSMTAIFLMSMLFGLACGQAMALCIPTE
Echinops_telfairi MTAIFLMSMVLGLVCGQAVSFCVPTE
50 60 70 80 90
Identity
Fukomys_anselli YTMHIDRRECAYCLTINTTICAGYCMTRDINGKLFLPKYALSQDVC
Cavia_porcellus YTMHIDRRECAYCLTINTTICAGYCMTRDSNRKTFVSSHARFQNVC
Octodon_degus YTMHIDRRECAYCLTINTTICAGYCITRDGNSKLTVPKNALSQNVC
Mus_musculus YTMYVDRRECAYCLTINTTICAGYCMTRDINGKLFLPKYALSQDVC
Rattus_norvegicus YMMYVDRRECAYCLTINTTICAGYCMTRDINGKLFLPKYALSQDVC
Ictidomys_tridecemlineatus YTMHVDRRECAYCLTINTTICAGYCMTRDINGKLFLPKYALSQDVC
Otolemur_garnettii YTMHVERRECAYCLTINTTICAGYCMTRDINGKLFLPKYALSQNVC
Macaca_fascicularis YTMHIERRECAYCLTINTTICAGYCMTRDINGKLFLPKYALSQDVC
Nomascus_leucogenys YTMHIERRECAYCLTINTTICAGYCMTRDINGKLFLPKYALSQDVC
Gorilla_gorilla YTMHIERRECAYCLTINTTICAGYCMTRDINGKLFLPKYALSQDVC
Pan_paniscus YTMHIERRECAYCLTINTTICAGYCMTRDINGKLFLPKYALSQDVC
Pan_troglodytes YTMHIERRECAYCLTINTTICAGYCMTRDINGKLFLPKYALSQDVC
Homo_sapiens YTMHIERRECAYCLTINTTMCAGYCMTRDINGKLFLPKYALSQDVC
Bos_taurus YMMHVERKECAYCLTINTTVCAGYCMTRDVNGKLFLPKYALSQDVC
Orcinus_orca YMMHVERKECAYCLTINTTICAGYCMTRDFNGKLFLPKYALSQDVC
Echinops_telfairi YTMHVERRECAYCLTINTTICAGYCITRDINGKLFLPKYALSQDVC
100 110 120 130
Identity
Fukomys_anselli TYRDFIYKTVEIPGCPHH-IAPYFSYPVAVSCKCGKCNTDYSDCIH
Cavia_porcellus TYGDFIYKTVEIPGCP-HQVAPYFSYPVAVSCKCGKCDTDYSDCIH
Octodon_degus TYRDFIYRTVEIPGCPRH-VAPYFSYPVAVSCKCGKCNTDYSDCIH
Mus_musculus TYRDFIYRTVEIPGCPHH-VTPYFSFPVAISCKCGKCNTDNSDCIH
Rattus_norvegicus TYRDFTYRTVEIPGCPHH-VAPYFSYPVALSCKCGKCNTDYSDCTH
Ictidomys_tridecemlineatus TYRDFIYRTVEIPGCPHH-VAPYFSYPVAVSCKCGKCNTDYSDCVH
Otolemur_garnettii TYRDFIYRTVEIPGCPHH-VAPYFSYPVAISCKCGKCNTDYSDCIH
Macaca_fascicularis TYRDFIYRTVEIPGCPLH-VAPYFSYPVALSCKCGKCNTDYSDCIH
Nomascus_leucogenys TYRNFIYRTVEIPGCPLH-VAPYFSYPVALSCKCGKCNTDYSDCIH
Gorilla_gorilla TYRDFIYRTVEIPGCPLH-VAPYFSYPVALSCKCGKCNTDYSDCIH
Pan_paniscus TYREFIYRTVEIPGCPLH-VAPYFSYPVALSCKCGKCNTDYSDCIH
Pan_troglodytes TYREFIYRTVEIPGCPLH-VAPYFSYPVALSCKCGKCNTDYSDCIH
Homo_sapiens TYRDFIYRTVEIPGCPLH-VAPYFSYPVALSCKCGKCNTDYSDCIH
Bos_taurus TYRDFMYKTAEIPGCPRH-VTPYFSYPVAISCKCGKCNTDYSDCIH
Orcinus_orca TYRDFMYKTVEIPGCPRH-VTPYFSYPVAISCKCGKCNTDYSDCIH
Echinops_telfairi TYRDFIYRTVEIPGCPDH-VAPYFSYPVAVSCKCGRCNTDYSDCIH
140 150 160 163
Identity
Fukomys_anselli EATKTNYCTKPQKSYLVGISV
Cavia_porcellus EATETNYCTKPEMSYLMGFSV
Octodon_degus EATKTNYCTKPQKSYLVGFSV
Mus_musculus EAVRTNYCTKPQSFYLGGFSV
Rattus_norvegicus EAVKTNYCTKPQTFYLGGFSG
Ictidomys_tridecemlineatus EAIKTNYCTKPQKSYLLGFSV
Otolemur_garnettii EAVKTNYCTKPQKSYLVGFSV
Macaca_fascicularis EAIKTNYCTKPQKSYLVGFSV
Nomascus_leucogenys EAIKTNYCTKPQKSYLVGFSV
Gorilla_gorilla EAIKTNYCTKPQKSYLVGFSV
Pan_paniscus EAIKTNYCTKPQKSYLVGFSV
Pan_troglodytes EAIKTNYCIKPQKSYLVGFFCLIVI
Homo_sapiens EAIKTNYCTKPQKSYLVGFSV
Bos_taurus EAIKTNYCTKPQKSYMVGFSI
Orcinus_orca EAIKTNYCTKPQKSYVVGFSI
Echinops_telfairi EAIKTNYCTKPQKPYMMGFSV

Figure S3. Protein alignment of thyreotropin β subunit (TSHB) from different mammal species.

The mRNA sequence of *F. anselli* was obtained from RNA-seq and subsequently translated, other sequences were retrieved from NCBI databases with the following accession numbers: Cavia porcellus (XP_003479306), Octodon degus (XP_004641725), Mus musculus (NP_001159412), Rattus norvegicus (NP_037248), Ictidomys tridecemlineatus (XP_005334966), Otolemur garnettii (XP_003793878), Macaca fascicularis (XP_001111873), Nomascus leucogenys (XP_003268073), Gorilla gorilla (XP_004026450), Pan paniscus (XP_003805682), Pan troglodytes (XP_001160337), Homo sapiens (AAB30828), Bos taurus (XP_005204060), Orcinus orca (XP_004263279), Echinops telfairi (XP_004714911).
