# Supplementary material for: Unusual Ratio between Free Thyroxine and Free Triiodothyronine in a Long-Lived Mole-Rat Species with Bimodal Ageing
Source: PLoS One. 2014 Nov 19;9(11):e113698. doi: 10.1371/journal.pone.0113698 (PMC4237498; doi:10.1371/journal.pone.0113698)
Supplement: Figure S4 — Protein alignment of Type I iodothyronine deiodinase (D1) from different mammal species. The mRNA sequence of F. anselli was obtained from RNA-seq and subsequently translated, other sequences were retrieved from NCBI databases with the following accession numbers: Heterocephalus glaber (XP_004908861), Cavia porcellus (NP_001244903), Octodon degus (XP_004642620), Mus musculus (Q61153), Rattus norvegicus (CAA41063), Cricetulus griseus (NP_001243688), Ochotona princeps (XP_004588749), Otolemur garnettii (XP_003793192), Macaca mulatta (NP_001116124), Pan troglodytes (NP_001116123), Homo sapiens (NP_000783), Canis lupus (NP_001007127), Felis catus (NP_001009267), Bos taurus (NP_001116065), Sus scrofa (NP_001001627), Equus caballus (NP_001159924), Orcinus orca (XP_004273874). (PDF) [file pone.0113698.s004.pdf]

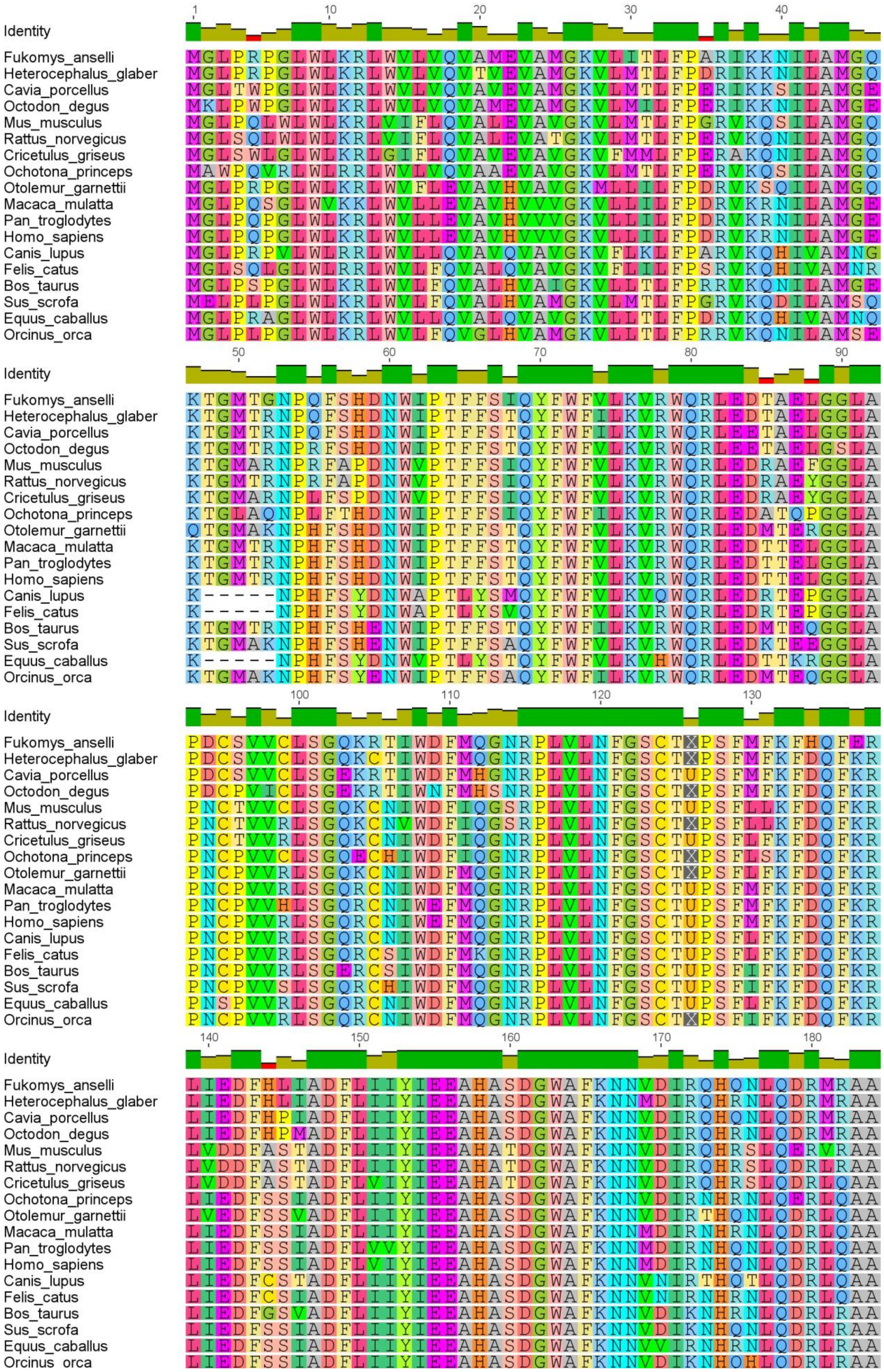
1 10 20 30 40
Identity
Fukomys_anselli MGLPRPGLWLKRLWVLVQVAMEVAMGKVLITLFPARIKKNILAMGQ
Heterocephalus_glaber MGLPRPGLWLKRLWVLVQVTVEVAMGKVLMTLFPDRIKKNILAMGQ
Cavia_porcellus MGLTWPGLWLKRLWVLVQVAVEVAMGKVLMTLFPERIKKSILAMGE
Octodon_degus MKLPWPGLWLKRLWVLVQVAMEVAMGKVLMILFPERIKKNILAMGE
Mus_musculus MGLPQLWLWLKRLVIFLQVALEVAVGKVLMTLFPGRVKQSILAMGQ
Rattus_norvegicus MGLSQLWLWLKRLVIFLQVALEVATGKVLMTLFPERVKQNILAMGQ
Cricetulus_griseus MGLSWLGLWLKRLGIFLQVAVEVAVGKVFMMLFPERAKQNILAMGQ
Ochotona_princeps MAWPQVRLWLRRLWVLVQVAAEVAVGKVLMTLFPERVKQSILAMGQ
Otolemur_garnettii MGLPRPGLWLKRLWVFLEVAVHVAVGKMLLILFPDRVKSQILAMGQ
Macaca_mulatta MGLPQSGLWVKKLWVLLEVAVHVVVGKVLLILFPDRVKRNILAMGE
Pan_troglodytes MGLPQPGLWLKRLWVLLEVAVHVVVGKVLLILFPDRVKRNILAMGE
Homo_sapiens MGLPQPGLWLKRLWVLLEVAVHVVVGKVLLILFPDRVKRNILAMGE
Canis_lupus MGLPRPVLWLRRLWVLLQVAVQVAVGKVFLKLFPARVKQHIVAMNG
Felis_catus MGLSQLGLWLRRLWVLFQVALQVAVGKVFLILFPSRVKQHIVAMNR
Bos_taurus MGLPSPGLWLKRLWVLFQVALHVAIGKVLLTLFPRRVKQNILAMGE
Sus_scrofa MELPLPGLWLKRLWVLFQVALHVAMGKVLMTLFPGRVKQDILAMSQ
Equus_caballus MGLPRAGLWLKRLWVLLQVALQVAVGKVLLTLFPDRVKQHIVAMNQ
Orcinus_orca MGLPLPGLWLKRLWVLFQVGLHVAMGKVLLTLFPRRVKQNILAMSE
50 60 70 80 90
Identity
Fukomys_anselli KTGMTGNPQFSHDNWIPTFFSIQYFWFVLKVRWQRLEDTAELGGLA
Heterocephalus_glaber KTGMTRNPQFSHDNWIPTFFSTQYFWFILKVRWQRLEDTAELGGLA
Cavia_porcellus KTGMTRNPQFSHDNWIPTFFSTQYFWFILKVRWQRLEETAELGGLA
Octodon_degus KTGMTRNPRFSHDNWIPTFFSTQYFWFVLKVRWQRLEETAELGSLA
Mus_musculus KTGMARNPRFAPDNWVPTFFSIQYFWFVLKVRWQRLEDRAEFGGLA
Rattus_norvegicus KTGMTRNPRFAPDNWVPTFFSIQYFWFVLKVRWQRLEDRAEYGGLA
Cricetulus_griseus KTGMARNPLFSPDNWVPTFFSIQYFWFVLKVRWQRLEDRAEYGGLA
Ochotona_princeps KTGLAQNPLFTHDNWIPTFFSIQYFWFILKVRWQRLEDATQPGGLA
Otolemur_garnettii QTGMAKNPHFSHDNWIPTFFSTQYFWFVLKVRWQRLEDMTERGGLA
Macaca_mulatta KTGMTRNPHFSHDNWIPTFFSTQYFWFVLKVRWQRLEDTTELGGLA
Pan_troglodytes KTGMTRNPHFSHDNWIPTFFSTQYFWFVLKVRWQRLEDTTELGGLA
Homo_sapiens KTGMTRNPHFSHDNWIPTFFSTQYFWFVLKVRWQRLEDTTELGGLA
Canis_lupus K-----NPHFSYDNWAPTLYSMQYFWFVLKVQWQRLEDRTEPGGLA
Felis_catus K-----NPHFSYDNWAPTLYSVQYFWFVLKVRWQRLEDRTEPGGLA
Bos_taurus KTGMTRNPHFSHENWIPTFFSTQYFWFILKVRWQRLEDMTEQGGLA
Sus_scrofa KTGMAKNPHFSHENWIPTFFSAQYFWFVLKVRWQRLEDKTEEGGLA
Equus_caballus K-----NPHFSYDNWVPTLYSTQYFWFVLKVHWQRLEDTTKRGGLA
Orcinus_orca KTGMAKNPHFSYENWIPTFFSAQYFWFILKVRWQRLEDMTEQGGLA
100 110 120 130
Identity
Fukomys_anselli PDCSVVCLSGQKRTIWDFMQGNRPLVLNFGSCTXPSFMFKFHQFER
Heterocephalus_glaber PDCSVVCLSGQKCTIWDFMQGNRPLVLNFGSCTXPSFMFKFDQFKR
Cavia_porcellus PDCSVVCLSGEKRTIWDFMHGNRPLVLNFGSCTUPSFMFKFDQFKR
Octodon_degus PDCPVICLSGEKRTIWNFMHSNRPLVLNFGSCTXPSFMFKFDQFKR
Mus_musculus PNCTVVCLSGQKCNIWDFIQGSRPLVLNFGSCTUPSFLLKFDQFKR
Rattus_norvegicus PNCTVVRLSGQKCNVWDFIQGSRPLVLNFGSCTXPSFLLKFDQFKR
Cricetulus_griseus PNCTVVRLSGQKCNIWDFIQGNRPLVLNFGSCTUPSFLFKFDQFKR
Ochotona_princeps PNCPVVCLSGQECHIWDFIQGNRPLVLNFGSCTXPSFLSKFDQFKR
Otolemur_garnettii PNCPVVRLSGQKCNIWDFMQGNRPLVLNFGSCTXPSFLFKFDQFKR
Macaca_mulatta PNCPVVRLSGQRCNIWDFMQGNRPLVLNFGSCTUPSFMFKFDQFKR
Pan_troglodytes PNCPVVHLSGQRCNIWEFMQGNRPLVLNFGSCTUPSFMFKFDQFKR
Homo_sapiens PNCPVVRLSGQRCNIWEFMQGNRPLVLNFGSCTUPSFMFKFDQFKR
Canis_lupus PNCPVVRLSGQRCNIWDFMQGNRPLVLNFGSCTUPSFLFKFDQFKR
Felis_catus PNCPVVRLSGQRCSIWDFMKGNRPLVLNFGSCTUPSFLFKFDQFKR
Bos_taurus PNCPVVRLSGERCSIWDFMQGNRPLVLNFGSCTUPSFIFKFDQFKR
Sus_scrofa PNCPVVSLSGQRCHIWDFMQGNRPLVLNFGSCTUPSFIFKFDQFKR
Equus_caballus PNSPVVRLSGQRCNIWDFMQGNRPLVLNFGSCTUPSFLFKFDQFKR
Orcinus_orca PNCPVVRLSGQRCNIWDFMQGNRPLVLNFGSCTXPSFIFKFDQFKR
140 150 160 170 180
Identity
Fukomys_anselli LIEDFHLIADFLIIYIEEAHASDGWAFKNNVDIRQHQNLQDRMRAA
Heterocephalus_glaber LIEDFHLIADFLIIYIEEAHASDGWAFKNNMDIRQHRNLQDRMRAA
Cavia_porcellus LIEDFHPIADFLIIYIEEAHASDGWAFKNNVDIRQHQNLQDRMRAA
Octodon_degus LIEDFHPMADFLIIYIEEAHASDGWAFKNNVDIRQHRNLQDRMRAA
Mus_musculus LVDDFASTADFLIIYIEEAHATDGWAFKNNVDIRQHRSLQERVRAA
Rattus_norvegicus LVDDFASTADFLIIYIEEAHATDGWAFKNNVDIRQHRSLQDRLRAA
Cricetulus_griseus LVDDFASTADFLVIYIEEAHATDGWAFKNNVDIRQHRSLQDRLQAA
Ochotona_princeps LIEDFSSIADFLIIYIEEAHASDGWAFKNNVDIRNHRNLQERLQAA
Otolemur_garnettii LVEDFSSVADFLIIYIEEAHASDGWAFKNNVDIRTHQNLQDRLQAA
Macaca_mulatta LIEDFSSIADFLIIYIEEAHASDGWAFKNNMDIRNHRNLQDRLQAA
Pan_troglodytes LIEDFSSIADFLVVYIEEAHASDGWAFKNNMDIRNHQNLQDRLQAA
Homo_sapiens LIEDFSSIADFLVIYIEEAHASDGWAFKNNMDIRNHQNLQDRLQAA
Canis_lupus LIEDFCSTADFLIIYIEEAHASDGWAFKNNVNIRTHQTLQDRLQAA
Felis_catus LIEDFCSIADFLIIYIEEAHASDGWAFKNNVNIRNHRNLQDRLQAA
Bos_taurus LIEDFGSVADFLIIYIEEAHASDGWAFKNNVDIKNHRNLQDRLRAA
Sus_scrofa LIEDFSSIADFLIIYIEEAHASDGWAFKNNVDIKNHQNLQDRLRAA
Equus_caballus LIEDFSSIADFLIIYIEEAHASDGWAFKNNVVIRNHRNLQDRLRAA
Orcinus_orca LIEDFSSIADFLIIYIEEAHASDGWAFKNNVDIKNHQHLQDRLRAA

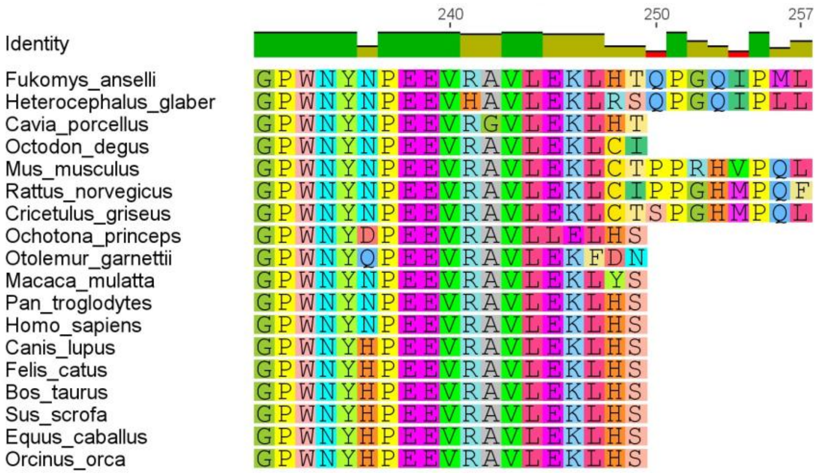

Figure S4. Protein alignment of Type I iodothyronine deiodinase (D1) from different mammal species. The mRNA sequence of *F. anselli* was obtained from RNA-seq and subsequently translated, other sequences were retrieved from NCBI databases with the following accession numbers: Heterocephalus glaber (XP_004908861), Cavia porcellus (NP_001244903), Octodon degus (XP_004642620), Mus musculus (Q61153), Rattus norvegicus (CAA41063), Cricetulus griseus (NP_001243688), Ochotona princeps (XP_004588749), Otolemur garnettii (XP_003793192), Macaca mulatta (NP_001116124), Pan troglodytes (NP_001116123), Homo sapiens (NP_000783), Canis lupus (NP_001007127), Felis catus (NP_001009267), Bos taurus (NP_001116065), Sus scrofa (NP_001001627), Equus caballus (NP_001159924), Orcinus orca (XP_004273874).
