# Supplementary material for: Unusual Ratio between Free Thyroxine and Free Triiodothyronine in a Long-Lived Mole-Rat Species with Bimodal Ageing
Source: PLoS One. 2014 Nov 19;9(11):e113698. doi: 10.1371/journal.pone.0113698 (PMC4237498; doi:10.1371/journal.pone.0113698)
Supplement: Figure S5 — Protein alignment of Type II iodothyronine deiodinase (D2) from different mammal species. The mRNA sequence of F. anselli was obtained from RNA-seq and subsequently translated, other sequences were retrieved from NCBI databases with the following accession numbers: Heterocephalus glaber (XP_004900438), Chinchilla lanigera (XP_005390287), Octodon degus (XP_004624767), Mus musculus (NP_034180), Rattus norvegicus (NP_113908), Ochotona princeps (XP_004584413), Homo sapiens (AAC95470), Canis lupus (NP_001116117), Ovis aries (XP_004011138), Sus scrofa (NP_001001626), Equus caballus (NP_001159927), Orcinus orca (XP_004262346), Condylura cristata (XP_004681708), Echinops telfairi (XP_004698804). (PDF) [file pone.0113698.s005.pdf]

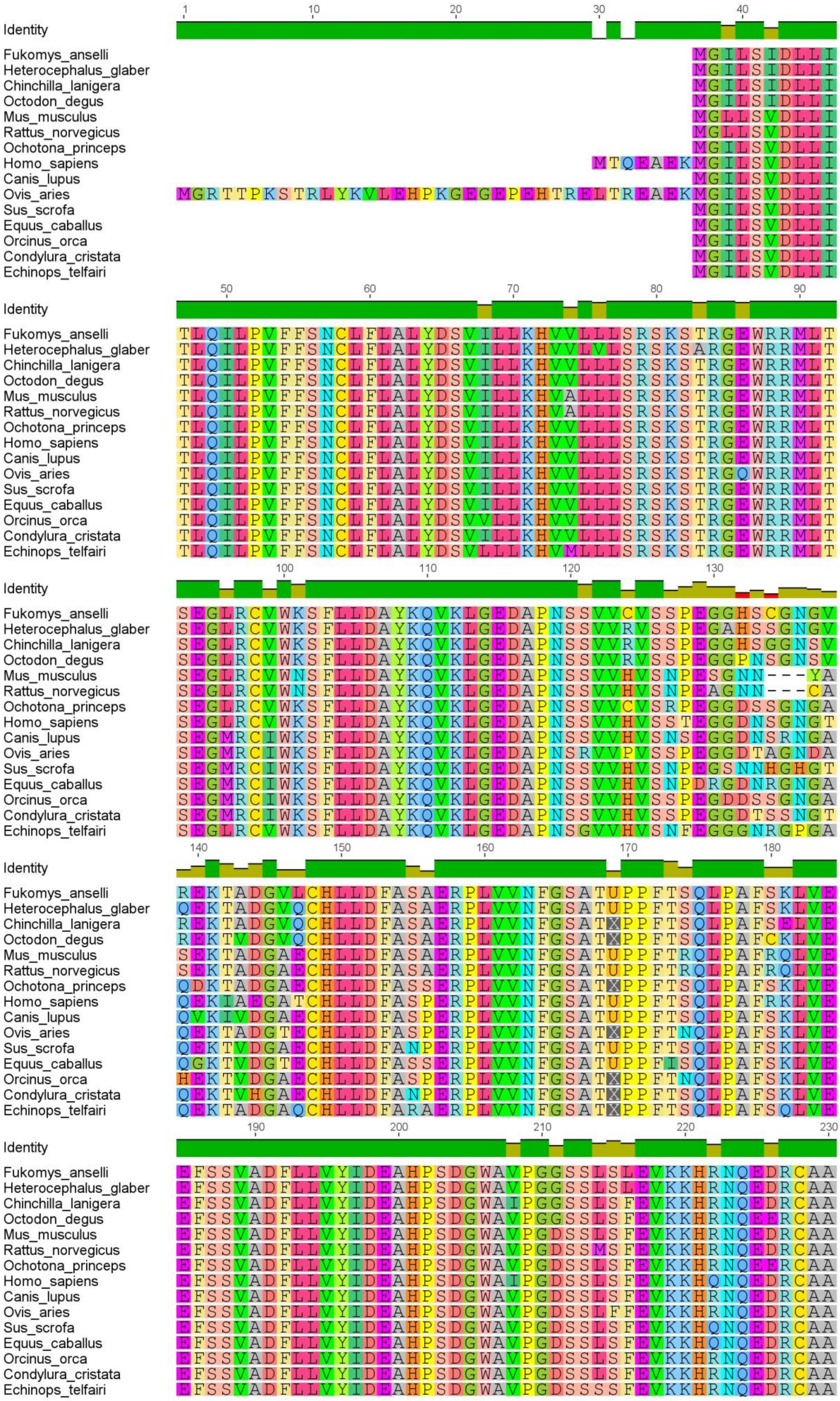

1 10 20 30 40
Identity
Fukomys_anselli MGILSIDLLI
Heterocephalus_glaber MGILSIDLLI
Chinchilla_lanigera MGILSIDLLI
Octodon_degus MGILSIDLLI
Mus_musculus MGLLSVDLLI
Rattus_norvegicus MGLLSVDLLI
Ochotona_princeps MGILSVDLLI
Homo_sapiens MTQEAEKMGILSVDLLI
Canis_lupus MGILSVDLLI
Ovis_aries MGRTTPKSTRLYKVLEHPKGEGEPEHTRELTREAEKMGILSVDLLI
Sus_scrofa MGILSVDLLI
Equus_caballus MGILSVDLLI
Orcinus_orca MGILSVDLLI
Condylura_cristata MGILSVDLLI
Echinops_telfairi MGILSVDLLI
50 60 70 80 90
Identity
Fukomys_anselli TLQILPVFFSNCLFLALYDSVILLKHVVLLLSRSKSTRGEWRRMLT
Heterocephalus_glaber TLQILPVFFSNCLFLALYDSVILLKHVVLVLSRSKSARGEWRRMLT
Chinchilla_lanigera TLQILPVFFSNCLFLALYDSVILLKHVVLLLSRSKSTRGEWRRMLT
Octodon_degus TLQILPVFFSNCLFLALYDSVILLKHVVLLLSRSKSTRGEWRRMLT
Mus_musculus TLQILPVFFSNCLFLALYDSVILLKHVALLLSRSKSTRGEWRRMLT
Rattus_norvegicus TLQILPVFFSNCLFLALYDSVILLKHVALLLSRSKSTRGEWRRMLT
Ochotona_princeps TLQILPVFFSNCLFLALYDSVILLKHVVLLLSRSKSTRGEWRRMLT
Homo_sapiens TLQILPVFFSNCLFLALYDSVILLKHVVLLLSRSKSTRGEWRRMLT
Canis_lupus TLQILPVFFSNCLFLALYDSVILLKHVVLLLSRSKSTRGEWRRMLT
Ovis_aries TLQILPVFFSNCLFLALYDSVILLKHVVLLLSRSKSTRGQWRRMLT
Sus_scrofa TLQILPVFFSNCLFLALYDSVILLKHVVLLLSRSKSTRGEWRRMLT
Equus_caballus TLQILPVFFSNCLFLALYDSVILLKHVVLLLSRSKSTRGEWRRMLT
Orcinus_orca TLQILPVFFSNCLFLALYDSVVLLKHVVLLLSRSKSTRGEWRRMLT
Condylura_cristata TLQILPVFFSNCLFLALYDSVILLKHVVLLLSRSKSTRGEWRRMLT
Echinops_telfairi TLQILPVFFSNCLFLALYDSVLLLKHVMLLLSRSKSTRGEWRRMLT
100 110 120 130
Identity
Fukomys_anselli SEGLRCVWKSFLLDAYKQVKLGEDAPNSSVVCVSSPEGGHSCGNGV
Heterocephalus_glaber SEGLRCVWKSFLLDAYKQVKLGEDAPNSSVVRVSSPEGAHSSGNGV
Chinchilla_lanigera SEGLRCVWKSFLLDAYKQVKLGEDAPNSSVVRVSSPEGGHSGGNSV
Octodon_degus SEGLRCVWKSFLLDAYKQVKLGEDAPNSSVVRVSSPEGGPNSGNSV
Mus_musculus SEGLRCVWNSFLLDAYKQVKLGEDAPNSSVVHVSNPESGNN---YA
Rattus_norvegicus SEGLRCVWNSFLLDAYKQVKLGEDAPNSSVVHVSNPEAGNN---CA
Ochotona_princeps SEGLRCVWKSFLLDAYKQVKLGEDAPNSSVVCVSRPEGGDSSGNGA
Homo_sapiens SEGLRCVWKSFLLDAYKQVKLGEDAPNSSVVHVSSTEGGDNSGNGT
Canis_lupus SEGMRCIWKSFLLDAYKQVKLGEDAPNSSVVHVSNSEGGDNSRNGA
Ovis_aries SEGMRCIWKSFLLDAYKQVKLGEDAPNSRVVPVSSPEGGDTAGNDA
Sus_scrofa SEGMRCIWKSFLLDAYKQVKLGEDAPNSSVVHVSNPEGSNNHGHGT
Equus_caballus SEGMRCIWKSFLLDAYKQVKLGEDAPNSSVVHVSNPDRGDNRGNGA
Orcinus_orca SEGMRCIWKSFLLDAYKQVKLGEDAPNSSVVHVSSPEGDDSSGNGA
Condylura_cristata SEGMRCIWKSFLLDAYKQVKLGEDAPNSSVVHVSSPEGGDTSSNGT
Echinops_telfairi SEGLRCVWKSFLLDAYKQVKLGEDAPNSGVVHVSNFEGGGNRGPGA
140 150 160 170 180
Identity
Fukomys_anselli REKTADGVLCHLLDFASAERPLVVNFGSATUPPFTSQLPAFSKLVE
Heterocephalus_glaber QEKTADGVQCHLLDFASAERPLVVNFGSATUPPFTSQLPAFSKLVE
Chinchilla_lanigera REKTADGVQCHLLDFASAERPLVVNFGSATXPPFTSQLPAFSELVE
Octodon_degus REKTVDGVQCHLLDFASAERPLVVNFGSATXPPFTSQLPAFCKLVE
Mus_musculus SEKTADGAECHLLDFASAERPLVVNFGSATUPPFTRQLPAFRQLVE
Rattus_norvegicus SEKTADGAECHLLDFASAERPLVVNFGSATUPPFTRQLPAFRQLVE
Ochotona_princeps QDKTADGAECHLLDFASSERPLVVNFGSATXPPFTSQLPAFSKLVE
Homo_sapiens QEKIAEGATCHLLDFASPERPLVVNFGSATUPPFTSQLPAFRKLVE
Canis_lupus QVKIVDGAECHLLDFASPERPLVVNFGSATUPPFTSQLPAFSKLVE
Ovis_aries QEKTADGTECHLLDFASPERPLVVNFGSATXPPFTNQLPAFSKLVE
Sus_scrofa QEKTVDGAECHLLDFANPERPLVVNFGSATUPPFTSQLPAFSKLVE
Equus_caballus QGKTVDGTECHLLDFASSERPLVVNFGSATUPPFISQLPAFSKLVE
Orcinus_orca HEKTVDGAECHLLDFASPERPLVVNFGSATXPPFTNQLPAFSKLVE
Condylura_cristata QEKTVHGAECHLLDFANPERPLVVNFGSATXPPFTSQLPAFSKLVE
Echinops_telfairi QEKTADGAQCHLLDFARAERPLVVNFGSATXPPFTSQLPAFSQLVE
190 200 210 220 230
Identity
Fukomys_anselli EFSSVADFLLVYIDEAHPSDGWAVPGGSSLSLEVKKHRNQEDRCAA
Heterocephalus_glaber EFSSVADFLLVYIDEAHPSDGWAVPGGSSLSLEVKKHRNQEDRCAA
Chinchilla_lanigera EFSSVADFLLVYIDEAHPSDGWAIPGGSSLSFEVKKHRNQEDRCAA
Octodon_degus EFSSVADFLLVYIDEAHPSDGWAVPGGSSLSFEVKKHRNQEERCAA
Mus_musculus EFSSVADFLLVYIDEAHPSDGWAVPGDSSLSFEVKKHRNQEDRCAA
Rattus_norvegicus EFSSVADFLLVYIDEAHPSDGWAVPGDSSMSFEVKKHRNQEDRCAA
Ochotona_princeps EFSSVADFLLVYIDEAHPSDGWAVPGDSSLSFEVKKHRNQEERCAA
Homo_sapiens EFSSVADFLLVYIDEAHPSDGWAIPGDSSLSFEVKKHQNQEDRCAA
Canis_lupus EFSSVADFLLVYIDEAHPSDGWAVPGDSSLSFEVKKHRNQEDRCAA
Ovis_aries EFSSVADFLLVYIDEAHPSDGWAVPGDSSLFFEVKKHRNQEDRCAA
Sus_scrofa EFSSVADFLLVYIDEAHPSDGWAVPGDSSLSFEVKKHQNQEDRCAA
Equus_caballus EFSSVADFLLVYIDEAHPSDGWAVPGDSSLSFEVKKHQNQEDRCAA
Orcinus_orca EFSSVADFLLVYIDEAHPSDGWAVPGDSSLSFEVKKHRNQEDRCAA
Condylura_cristata EFSSVADFLLVYIDEAHPSDGWAVPGDSSLSFEVKKHRNQEDRCAA
Echinops_telfairi EFSSVADFLLVYIDEAHPSDGWAVPGDSSSSFEVKKHRNQEDRCAA

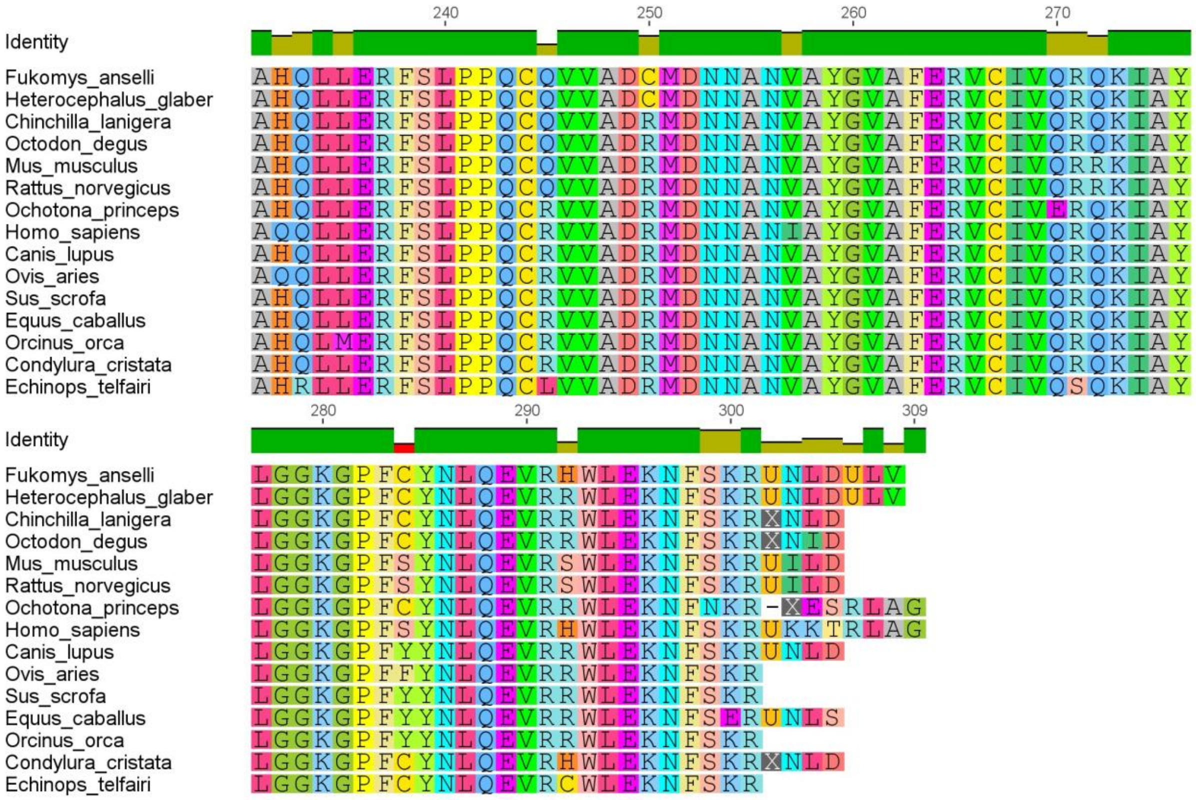

Figure S5. Protein alignment of Type II iodothyronine deiodinase (D2) from different mammal species. The mRNA sequence of *F. anselli* was obtained from RNA-seq and subsequently translated, other sequences were retrieved from NCBI databases with the following accession numbers: Heterocephalus glaber (XP_004900438), Chinchilla lanigera (XP_005390287), Octodon degus (XP_004624767), Mus musculus (NP_034180), Rattus norvegicus (NP_113908), Ochotona princeps (XP_004584413), Homo sapiens (AAC95470), Canis lupus (NP_001116117), Ovis aries (XP_004011138), Sus scrofa (NP_001001626), Equus caballus (NP_001159927), Orcinus orca (XP_004262346), Condylura cristata (XP_004681708), Echinops telfairi (XP_004698804).
