# Supplementary material for: Unusual Ratio between Free Thyroxine and Free Triiodothyronine in a Long-Lived Mole-Rat Species with Bimodal Ageing
Source: PLoS One. 2014 Nov 19;9(11):e113698. doi: 10.1371/journal.pone.0113698 (PMC4237498; doi:10.1371/journal.pone.0113698)
Supplement: Figure S6 — Protein alignment of thyroglobulin (TG) from different mammal species. The mRNA sequence of F. anselli was obtained from RNA-seq and subsequently translated, other sequences were retrieved from NCBI databases with the following accession numbers: Cavia porcellus (XP_003467392), Chinchilla lanigera (XP_005398080), Octodon degus (XP_004642544), Mus musculus (AAB53204), Rattus norvegicus (BAL14775), Ochotona princeps (XP_004580794), Otolemur garnettii (XP_003792914), Macaca mulatta (EHH28780), Pan troglodytes (XP_003311969), Homo sapiens (AAC51924), Canis lupus (XP_005627864), Felis catus (XP_004000173), Sus scrofa (NP_001161890), Equus caballus (XP_001916622), Orcinus orca (XP_004265356), Echinops telfairi (XP_004697442). (PDF) [file pone.0113698.s006.pdf]

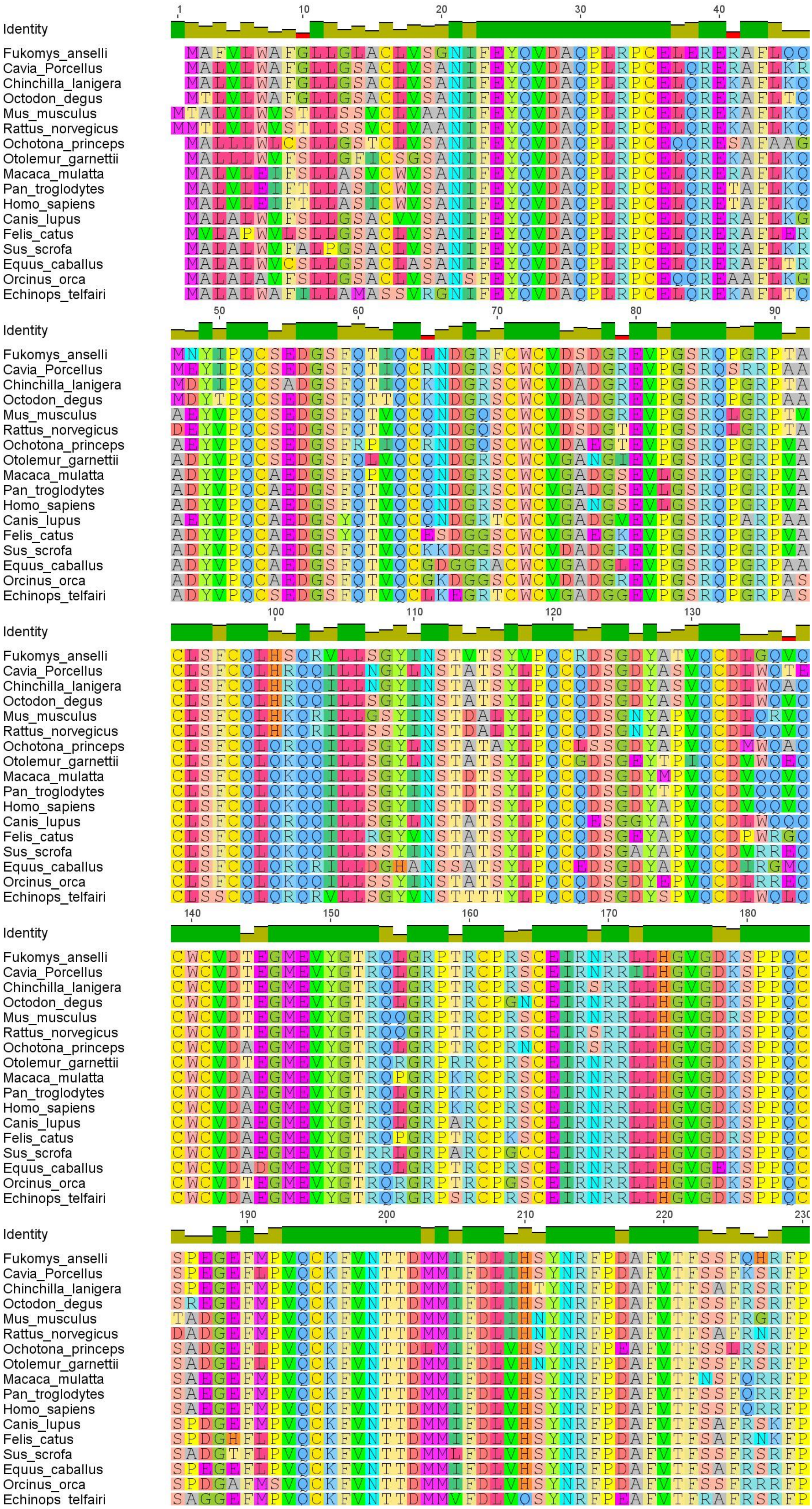
Identity
Fukomys_anselli
Cavia_Porcellus
Chinchilla_lanigera
Octodon_degus
Mus_musculus
Rattus_norvegicus
Ochotona_princeps
Otolemur_garnettii
Macaca_mulatta
Pan_troglodytes
Homo_sapiens
Canis_lupus
Felis_catus
Sus_scrofa
Equus_caballus
Orcinus_orca
Echinops_telfairi

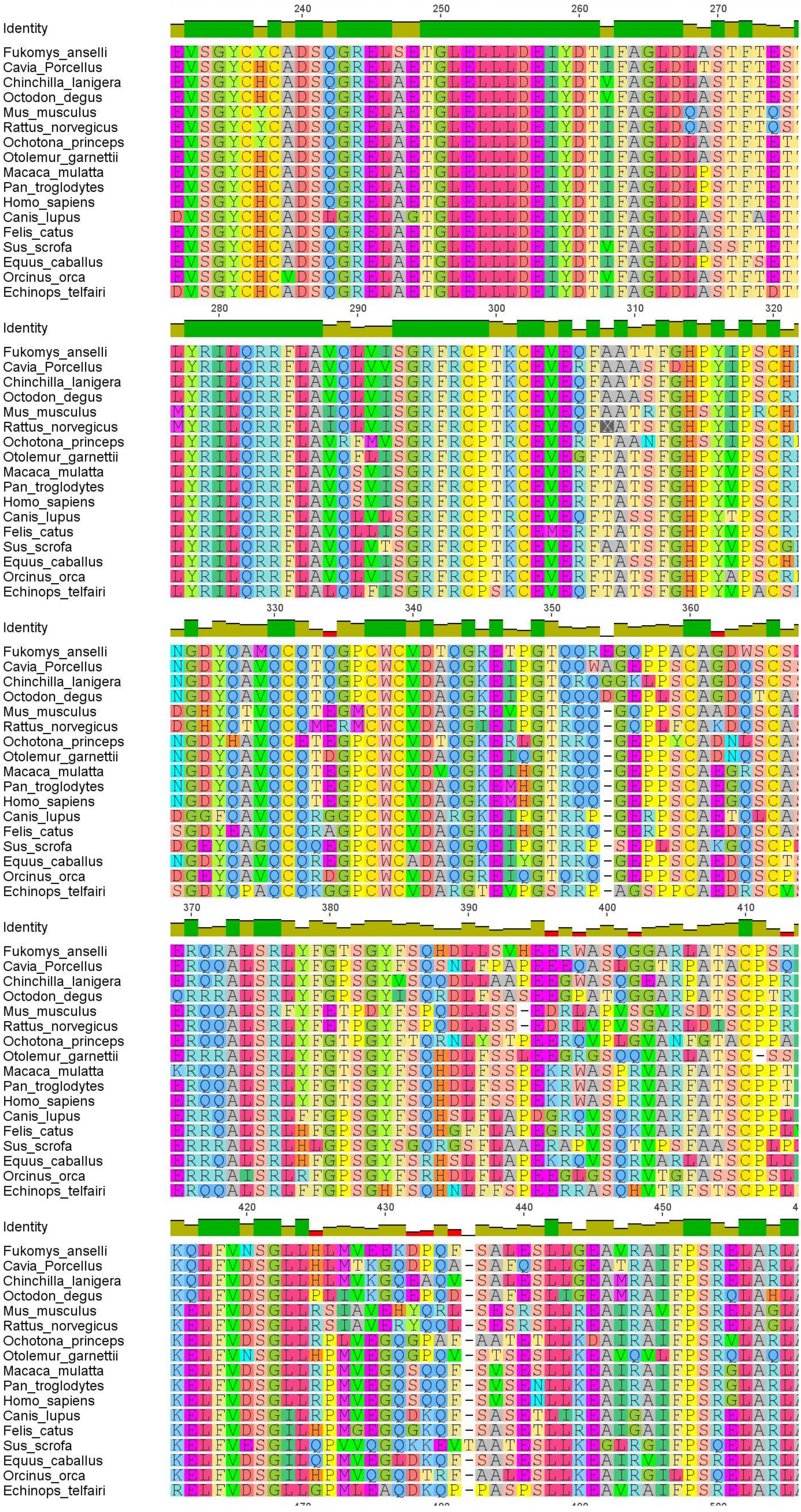
240
250
260
270
Identity
Fukomys_anselli
Cavia_Porcellus
Chinchilla_lanigera
Octodon_degus
Mus_musculus
Rattus_norvegicus
Ochotona_princeps
Otolemur_garnettii
Macaca_mulatta
Pan_troglodytes
Homo_sapiens
Canis_lupus
Felis_catus
Sus_scrofa
Equus_caballus
Orcinus_orca
Echinops_telfairi
280
290
300
310
320
330
340
350
360
370
380
390
400
410
420
430
440
450

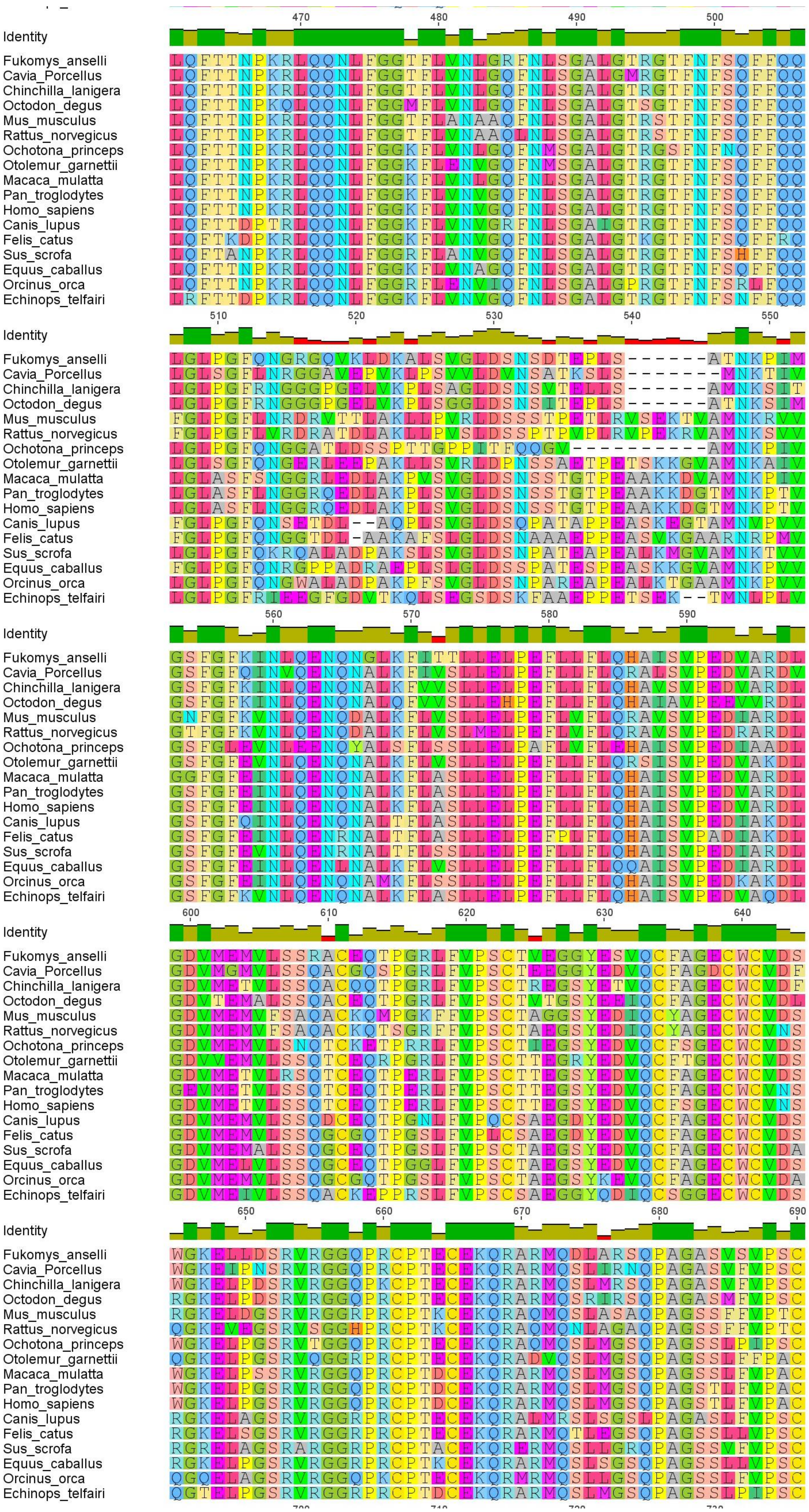
Identity
Fukomys_anselli
Cavia_Porcellus
Chinchilla_lanigera
Octodon_degus
Mus_musculus
Rattus_norvegicus
Ochotona_princeps
Otolemur_garnettii
Macaca_mulatta
Pan_troglodytes
Homo_sapiens
Canis_lupus
Felis_catus
Sus_scrofa
Equus_caballus
Orcinus_orca
Echinops_telfairi
470
480
490
500
510
520
530
540
550
560
570
580
590
600
610
620
630
640
650
660
670
680
690

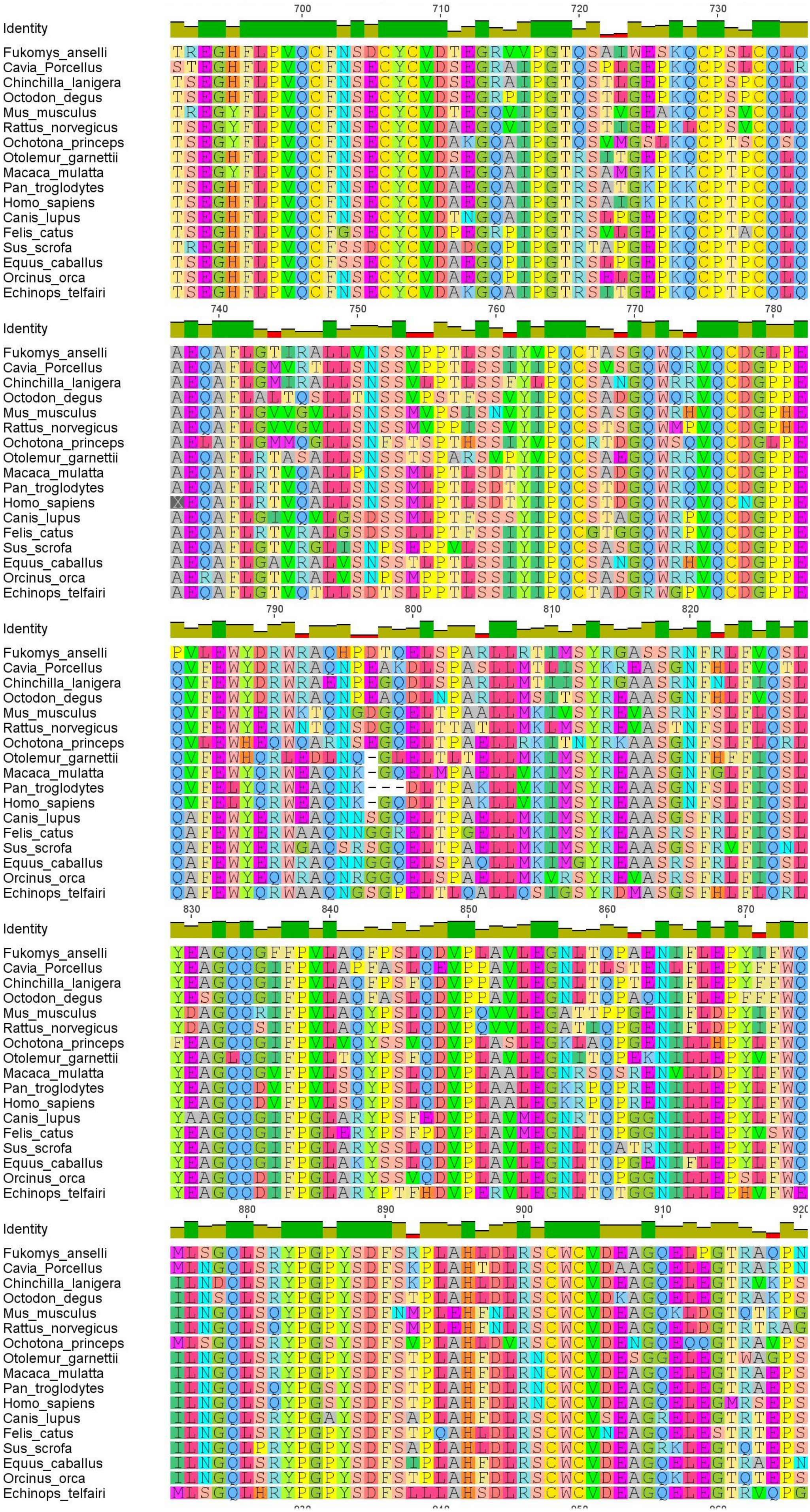
Identity
Fukomys_anselli
Cavia_Porcellus
Chinchilla_lanigera
Octodon_degus
Mus_musculus
Rattus_norvegicus
Ochotona_princeps
Otolemur_garnettii
Macaca_mulatta
Pan_troglodytes
Homo_sapiens
Canis_lupus
Felis_catus
Sus_scrofa
Equus_caballus
Orcinus_orca
Echinops_telfairi

```
                          930       940       950       960
Identity
Fukomys_anselli       QVPACPGSCEEAKLRVLQFIKETEEIVSVSNTSRFPLGESFLVAKG
Cavia_Porcellus       QVPACPGSCEEVKLDVLQFIKETEEIVSAFNTSRFLVGESFLIAKG
Chinchilla_lanigera   QVPACLGSCEETKLYVLQFIKETEEIVSASNTSQFPLGESFLVAKG
Octodon_degus         QIPACPGACEEAKLYVLQFIKETEEIVSASNTSRFPLGESFLVAKG
Mus_musculus          EIPACPGPCEEVKLRVLKFIKETEEIVSASNASSFPLGESFLVAKG
Rattus_norvegicus     EIPACPGPCEEVKFRVLKFIKETEEIVSASNASSFPLGESFLVAKG
Ochotona_princeps     KVPSCPGSCEEVKLHVLKFIKEAEELVSASNSSWFPLGESFLVAKG
Otolemur_garnettii    TFPACAGSCAEVKLRVLQLMRETEEIASASNSSRFPLGESFLVAKG
Macaca_mulatta        KLPTCPGSCEEAKLLVLQFIRETEEIVSASNSSRFPLGESFLVAKG
Pan_troglodytes       KLPT------------------------------------------
Homo_sapiens          KLPTCPGSCEEAKLRVLQFIRETEEIVSASNSSRFPLGESFLVAKG
Canis_lupus           KVPACPGSCEEVKLRVLQFLKETEEIVLASNSSWFPLGESFLAAKG
Felis_catus           KVPACPGSCEEVKFRVLQFIKETEDIVLASNSSWFPLGESFLAAKG
Sus_scrofa            KVPACPGSCEEVKLRVLQFIKEAEEIVMVSNSSQFPLGESFLAAKG
Equus_caballus        KVPACPGSCEEVKLHILRFIEETEEIVLASNSSQFPLGESFLAAKG
Orcinus_orca          KVPACPGSCEEVKLRVLQFIKEAEEIVMDSNSSRFPLGESFLAAKG
Echinops_telfairi     AVPACPGSCEGAKLRVLQFIRETEAVVSASNSSRFPLGESFLAAKG

                         970       980       990       1,000     1,010
Identity
Fukomys_anselli       IWLTNEELSLPRFFPSREAFFEKFLHGS-DYTIQLAAQSTLNFYQR
Cavia_Porcellus       IRLTSEELRLPPHFPSQEAFFEKFLRGR-DYAIRLAAQSTSNLYQR
Chinchilla_lanigera   IRLTSEELSLPPQFPCREAFFEKFLRGS-DYAIRLAAESTLNFYQR
Octodon_degus         IRLTSKELSLAPQLLPQEAFFERFLRGS-DYAIQLAAQSTLNFYQR
Mus_musculus          IQLTSEELDLPPQFPSRDAFSEKFLRGG-EYAIRLAAQSTLTFYQS
Rattus_norvegicus     IQLTSEELGLPPLYPSREAFSEKFLRGS-EYAIRLAAQSTLTFYQK
Ochotona_princeps     IQLTNEELGLSPVYPSRKALSENFLRGG-DGAIRLAAQSTFAFYQR
Otolemur_garnettii    IRLSRAELGLPQGAPSWETFSERFLRGDDDHAVLLAALSALTFYQR
Macaca_mulatta        IRLRNEDLGLPPLFPPREALAEQFLRGS-DYAIRLAAQSTLSFYQR
Pan_troglodytes       ----------------------------------------SLSFYQR
Homo_sapiens          IRLRNEDLGLPPLFPPREAFAEQFLRGS-DYAIRLAAQSTLSFYQR
Canis_lupus           IWLTDEELSLPRLSPSRETFSEKFLSGG-DYALRLAAQSTLDFYQR
Felis_catus           ILLTDEELSLPQLSPSRATFSEKFLSGG-DYAIRLAAQSTLDFYQR
Sus_scrofa            IRLTDEELALPPLSPSRETFLEKFLSGS-DYAIRLAAQSTFSFYQR
Equus_caballus        IWLTSEELALPRLSPSWETVSEKFLSGN-DYAIRLAAQSTFDFYQR
Orcinus_orca          IRLTDEELALPRLSPPRETFLEKFLSGS-DYAIRLAALSTFDFYQR
Echinops_telfairi     IRLTGEE------------FSETFLSGS-EYALRLAAQSTFTFYQR

                             1,020     1,030     1,040     1,050
Identity
Fukomys_anselli       YRAYQG-DLTATAAPRWPDPYMPQCDAFGSWEPRQCHMGTGYCWCV
Cavia_Porcellus       LRTSQD-DWSETAVPLRSGPYVPQCDASGNWEPRQCHMGTGYCWCM
Chinchilla_lanigera   HRASRS-DWVGAAAPLRSGPYVPQCDAFGGWEPRQCHLGTGYCWCV
Octodon_degus         RHASLN-DWIGAASALGSGPYVPQCDAFGSWEPRQCHTGTGYCWCV
Mus_musculus          LRASLG-KSDGAASLLWSGPYMPQCNMIGGWEPVQCHAGTGQCWCV
Rattus_norvegicus     LRASLG-ESNGTASLLWSGPYMPQCNTIGGWEPVQCHPGTGQCWCV
Ochotona_princeps     RRSSLG-NSARMPSLLQPGPYVPQCNAFGSWEPVQCHAATGHCWCV
Otolemur_garnettii    RRSSLD-NWVEVPTLPRSGPYMPQCDAFGNWEPVQCHAGTGHCWCV
Macaca_mulatta        RRFSLD-DSAGASALLQLGPYVPQCDAFGSWEPVQCHTGTGHCWCV
Pan_troglodytes       RRFSPD-DSAGASALLRSGPYVPQCDAFGSWEPVQCHAGTGHCWCV
Homo_sapiens          RRFSPD-DSAGASALLRSGPYXPQCDAFGSWEPVQCHAGTGHCWCV
Canis_lupus           RGFLLG-DSTRTSALLRPVPYVPQCDMWGGWEPVQCHARTGYCWCV
Felis_catus           RAFLQGDDSTRASALLRPGPYVPQCDAWGRWEPVQCHARTGYCWCV
Sus_scrofa            RRVALS-DAPRTSGPLQPYPYVPQCDALGSWEPVQCHAATGHCWCV
Equus_caballus        RRFALG-DSARTSALLPPSPYVPQCDALGRWEPVQCYARTGHCWCV
Orcinus_orca          LRVHLG-RSPRASTPLRSSTYVPQCDAFGSWEPVQCHAATGLCWCV
Echinops_telfairi     RGTSPG-GWAGAATLSSKDPYVPQCDASGNWEPTQCYDRTGHCWCV

                       1,060     1,070     1,080     1,090     1,100
Identity
Fukomys_anselli       DGTGQFVPGSLTVYSPQVLQCASRCEQSRANGLLSGWKQAGSQGDP
Cavia_Porcellus       DAKGEFIPNSLTARSPQVLQCPSPCEQSRANGLLSGWKQAGSQGDP
Chinchilla_lanigera   DAKGKFVPNSLTAHSPQLLQCPTPCEQSRANGLLSGWKQAGSQGDP
Octodon_degus         DVMGNFVPNSLTARSPQLLQCPSPCEQSRANALLSGWKQAGSQGDP
Mus_musculus          DGRGEFIPGSLMSRSSQMPQCPTNCELSRASGLISAWKQAGPQRNP
Rattus_norvegicus     DGWGELIPGSLMARSSQMPQCPTSCELSRANGLISAWKQAGHQRNP
Ochotona_princeps     DGRGETIPASLTVHSTQALQCPTTCERSRANGLLSSWKQAATQVNP
Otolemur_garnettii    DGKGEFVPASLTTRSPQIPQCPTTCEKSRANGLLSGWKQAGSQGNP
Macaca_mulatta        DEKGGFIPASLTARSLQIPQCPTTCEKSRTSGLLSSWKQARSQGNP
Pan_troglodytes       DEKGGFIPASLTARSLQIPQCPTTCEKSRTSGLLSSWKQARSQENP
Homo_sapiens          DEKGGFIPGSLTARSLQIPQCPTTCEKSRTSGLLSSWKQARSQENP
Canis_lupus           DGKGEYVPASLTARSPRILRCPTACEKSRASGLLSSWKQAGSQGNL
Felis_catus           DRRGEYVPASLTARSSQIPQCPTACEASRTSGLLSSWKQAGSQGSP
Sus_scrofa            DGEGAYLPASLAARSPQVLQCPSPCETSRVRGLLSAWKQAGSQVRP
Equus_caballus        DGKGEYVPASLTARSPQMPQCPTTCEKSRASGLLASWKQAGSQGNP
Orcinus_orca          DGKGEYVPTSLAARSPQIPQCPTSCEKSRARALLSSWKQAGSQENP
Echinops_telfairi     DGKGEYIPASLATRPAQVPQCPRPCEAARASGLVSLWKQAAGQASP

                            1,110     1,120     1,130     1,140     1,15
Identity
Fukomys_anselli       SPEDLFTPTCHETGEFVMMQASGTGTWCVDPVSGEGMWPSTNGSTQ
Cavia_Porcellus       SPEGLFIPTCLETGEFARMQVSGAGAWCVDPVSGEGMQPSTNGSTQ
Chinchilla_lanigera   SPEDLFTPSCLSTGEFARTQVSGTGAWCVDPVSGEGMRPSTNDNAQ
Octodon_degus         SPEDLFTPTCTETGEFARRQVSGTGAWCVDPASGEGTQRSANGSAP
Mus_musculus          GPGDLFIPVCLQTGEYVRKQTSGTGTWCVDPASGEGMPVNTNGSAQ
Rattus_norvegicus     GPGDLFTPVCLQTGEYVRQQTSGTGAWCVDPSSGEGVPTNTNSSAQ
Ochotona_princeps     SPEDLFTPTCLATGEYASLQKSEAGPWCVDPKTGEGMPPDASSRSH
Otolemur_garnettii    SPKDLFIPACLETGEHSRLQASEAGIWCVDPTSGERMPPGSNSSAQ
Macaca_mulatta        SPKDLFIPACLETGEYARLQASEAGTWCVDPASGEELLPGSNSSAQ
Pan_troglodytes       SPRDLFVPACLETGEYARLQASGAGTWCVDPASGEELRPGSNSSAQ
Homo_sapiens          SPKDLFVPACLETGEYARLQASGAGTWCVDPASGEELRPGSSSSAQ
Canis_lupus           SPKDLFIPTCLETGEFARRQESEGGTWCVDPGSGAGRPPGTDSSAP
Felis_catus           SPKDLFIPTCLETGEFARLQESEAGAWCVDPASGAGLPLATNSSAP
Sus_scrofa            SPKDLFIPACTETGEFARLQASEASTWCVDPASGEATPPGTNSSAP
Equus_caballus        SPEDLFIPTCLETGEFARLQASEAGTWCVDPATGEGMPPGTNSTAQ
Orcinus_orca          SPKDLFIPTCLETGEFARLQVSEASTWCVDPASGEGTPPGTNGSAQ
Echinops_telfairi     SPKELFVPTCLETGEFARLQESEGSTWCVDPMSGEGEGQEAGSDAP
```

```
                     1,160     1,170     1,180     1,190
Identity
Fukomys_anselli      CPGLCDALRSGALSRRVGPGYIPACREQDGSFSRVQCDPARGSCWC
Cavia_Porcellus      CPGLCSVLRNRVLSRTVSPGYTPTCGTEDGAFSLVQCDPAQGSCWC
Chinchilla_lanigera  CPGLCNVLRSGVLSRLVSPGYIPACRAQDGAFSLVQCDPAQGSCWC
Octodon_degus        CPGLCDVLTNGVLSRRVGTGHMPACRSQDGAFSLVQCDPAQGSCWC
Mus_musculus         CPGLCDVLKSRALSRKVGLGYSPVCEALDGAFSPVQCDLAQGSCWC
Rattus_norvegicus    CPGLCDALKSRVLSRKVGLGYTPVCEALDGGFSPVQCDLAQGSCWC
Ochotona_princeps    CLGLCEVLKSEVLFRRVGPGYIPACQEQDGSFAPVQCESTQGSCWC
Otolemur_garnettii   CPGLCDVLKSGVLSRTVGLGFIPDCDAQNGGFSPMQCDPDQSSCWC
Macaca_mulatta       CPSLCNVLKSGVLSRRVSSGYVPACREEDGGFSPVQCDQAQGSCWC
Pan_troglodytes      CPSLCNVLKSGVLSRRVSPGYVPACRAEDGGFSPVQCDQAQGSCWC
Homo_sapiens         CPSLCNVLKSGVLSRRVSPGYVPACRAEDGGFSPVQCDQAQGSCWC
Canis_lupus          CPSLCEELSSGTLSRRASSGYSPACRAEDGGFSPMQCDLAQGSCWC
Felis_catus          CPSLCDVLPSGVLPRRASSGSTPACGAEDGRFSPVRCDPAQGSCWC
Sus_scrofa           CPGLCEVLQRGVPSRRASPGTTPACRAEDGGFAPVQCDPAQGSCWC
Equus_caballus       CLSRCEGLQRGDPSRRASPGYTPACRAEDGGFSPVQCDLDQGSCWC
Orcinus_orca         CPSLCEVLQSGVPSRRASPGYAPACTAEDGGFSPVQCDPSQGSCWC
Echinops_telfairi    CPSLCEALRSRVLARRAGPGYTPACEAGPGGFSPVQCDPVGGSCWC

                     1,200     1,210     1,220     1,230     1,240
Identity
Fukomys_anselli      VLDSGEEVPGTRVVGSQPACERPQCPLPFLSVSDVPGGEILCERAL
Cavia_Porcellus      VLDSGEEVPGTHVVGSQPACERPQCPLPFLSLSDMPGGVILCKRAS
Chinchilla_lanigera  VLDSGEEVPGTRVVGSQPACERPQCPLPFSSGSDMPGGVILCKTTS
Octodon_degus        VLDSGEEVPGTRVVGHQPACERPQCPLPYMNVSDMPGGVILCERAS
Mus_musculus         VLGSGEEVPGTRVVGTQPACESPQCPLPF-SGSDVADGVIFCETAS
Rattus_norvegicus    VLGSGEEVPGTRVVGTQPACESPQCPLPF-SGSDVTDGVVFCETAS
Ochotona_princeps    VLGSGEEVPGTRVSSRQPACESPQCPLPF-NMSEVPGGVILCGPAL
Otolemur_garnettii   VTGSGEEVSETRVAGSKPACESPQCRLPF-DASGLAGGAILCESVL
Macaca_mulatta       VTDSGEEVPETRVAGSQPACESPRCPLPF-NTLEVVGGTILCETAS
Pan_troglodytes      VMDSGEEVPGTRVAGGQPACESPRCPLPF-NASEVVGGTILCETTS
Homo_sapiens         VMDSGEEVPGTRVTGGQPACESPRCPLPF-NASEVVGGTILCETIS
Canis_lupus          VLDSGEEVPGTRVAGSQLACESPRCPLPF-NTSDVDGGVIVCERAS
Felis_catus          VLATGEEVPGTREAGRQPACESPQCPLPF-NTSDVAGGAILCERAA
Sus_scrofa           VLGSGEEVPGTRVAGSQPACE-------------------------
Equus_caballus       VLGSGEEVPGTHVAGSQPACESPQCPLPF-NVSEVAGGVILCERAS
Orcinus_orca         VLGSGEEVPGTRVAGSQPACESPQCPLPF-NVSDVAGGALLCAPAS
Echinops_telfairi    ILDNGEEVPGTRVIGSQPTCERPQCPLPF--VAAIADGMVLCEEDS

                     1,250     1,260     1,270     1,280
Identity
Fukomys_anselli      DLG-EATVQQCRLWCRQGYRSIVPSGLLECSLQSKHWVSQPPQPLA
Cavia_Porcellus      GLG-EATVQQCQLFCRQGYRSAFPSGLLVCNLESKRWVSQPPQPLA
Chinchilla_lanigera  GPGEAALVQQCQLFCRQGYHSVFPLGLLVCSLESKRWVSQLPQPLA
Octodon_degus        GPG-AVTVQQCQLFCRQGYQSVFPSGVLECSLESKRWVSQLPQPLA
Mus_musculus         SSG-VTTVQQCQLLCRQGLRSAFSPGPLICSLESQHWVT-LPPPRA
Rattus_norvegicus    SSG-VTTVQQCQLFCRQGLRNVFSPGPLICNLESQRWVT-LPLPRA
Ochotona_princeps    GSP-GATVQQCRLLCLWGYQNAFLPQPLVCSLESRRWVSPPPQPRA
Otolemur_garnettii   GPA-GAVVQQCRLLCRQGYRSVFPEGLLVCSLESGRWESPPPQPRA
Macaca_mulatta       GPT-GAAIQQCQLLCRQGSRSVFPRGPLICSLESRRWESQLPQPRA
Pan_troglodytes      GPT-GAAIQQCQLLCRQGSWSVFPPGPLICSLESGRWESQPPQPRA
Homo_sapiens         GPT-GSAMQQCQLLCRQGSWSVFPPGPLICSLESGRWESQLPQPRA
Canis_lupus          GPG-GAPVQRCQLLCRRGYRSAFLPGPLVCSLEEGRWLSQPPQPQA
Felis_catus          GPG-GSAIQRCQLRCLRGYWSAFPPGPLLCGLEEGRWVSQPPQPHA
Sus_scrofa           ----------------------------------------------
Equus_caballus       GE---ATVXRCQLLCRWGYRSAFPPGPLVCSPQRRLWVSQPPQPRA
Orcinus_orca         GPG-GAVVQRCQLLCRPGYRSAFLPGPLLCSLQRRRWESQPPQPRA
Echinops_telfairi    GPG-DAAVQRCQLKCRQGFQSAFPPRVLVCSLGHRRWETGPPPAHA

                     1,290     1,300     1,310     1,320     1,330
Identity
Fukomys_anselli      CQRPQPWQTVQTGVHFRLQLPPGKMCSADYKGLLQAFQVFIQDELM
Cavia_Porcellus      CQRPQLWQTVQTGAHFQILLPPGKRCSADYSGLLQAFRVFILDELM
Chinchilla_lanigera  CQSPQPWQTVQTGAHFQLQLPPGKMCSADYTGLLRAFQVFILDELM
Octodon_degus        CQWPQPWQTVQTGANFQLQLPPGQMCSADYTGLLQAFQVFISDALV
Mus_musculus         CQRPQLWQTMQTQAHFQLLLPPGKMCSVDYSGLLQAFQVFILDELI
Rattus_norvegicus    CQRPQLWQTMQTQAHFQLLLPPGKMCSIDYSGLLQAFQVFILDELI
Ochotona_princeps    CQRPQLWQSLQAQGHIQLRLPPGKMCSVDYAGLLQAFQVFILDELT
Otolemur_garnettii   CQRPQPWQTIQTQAHFQLQLPPGKMCSVDYAGLLWAFRVFIGDELM
Macaca_mulatta       CQRPQLWQTIQTQGHFQLQLPPGKMCSADYAGLLQAFQVFILDELT
Pan_troglodytes      CQRPQLWQTMQTQGHFQLQLPPGKMCSADYASLLQTFQVFILDELT
Homo_sapiens         CQRPQLWQTIQTQGHFQLQLPPGKMCSADYAGLLQTFQVFILDELT
Canis_lupus          CQRPHLWQTFQTQGQFQLQLPPGKMCSADYAGLLPAFQVFLADELE
Felis_catus          CQRPQLWQTVQTRARVLLRLPPGKMCSADYAGLLPALRVFISDELV
Sus_scrofa           --RPQLWQTIQTRGQFQLQLPPGKVCSADYAGLLPTFQVVILDELT
Equus_caballus       CQRPQLWQTLQTGAQIQLQLPPGSLCRADDAGLLPVFQGFILDELM
Orcinus_orca         CQRPQLWQTLWTQGQLQLQLPPGKVCHADYAGLLPAFQHFLLDELT
Echinops_telfairi    CQRVQPWQTVQTEAQFQLHLPPGKMCSADYAGLLRAFQVFLLDELT

                     1,340     1,350     1,360     1,370     1,380
Identity
Fukomys_anselli      ARGFCHIQVWKTGAPTSHPVCDSSSVQVGCLTTERLGVNITWKSQF
Cavia_Porcellus      ARGFCHVQVPTAGTLVSHPVCDNSSVQVGCLTAEHLEVNVTWKSQL
Chinchilla_lanigera  ARGFCHVQVRTTGTPVSHPVCDNSSVQVGCLTAERLQVNVTWKSQL
Octodon_degus        AHGLCHMQVRTAGTVVSYPVCDNSSVQVGCLTAERLGVNVTWTSQL
Mus_musculus         TRGFCQIQVKTFGTLVSSTVCDNSSIQVGCLTAERLGVNVTWKLQL
Rattus_norvegicus    TRGFCQIQVKTFGTLVSRTVCDNSSIQVGCLTAERLGVNATWKLQL
Ochotona_princeps    ARGFCQIQVKTLGTPLSIPACDNSSVQVWCLTGERLGVNITWNSRL
Otolemur_garnettii   ARGFCQIQVKTFDTLVSIPVCDDSSVQVGCVTGERLGVNVTWKSRL
Macaca_mulatta       ARGFCQIQVKTFGTLVSVPVCDNSSVQVGCLTREYLGVNVTWKSRL
Pan_troglodytes      ARGFCQIQVKTFGTLVSIPVCNNSSVQVGCLTSERLGVNVTWKSRL
Homo_sapiens         ARGFCQIQVKTFGTLVSIPVCNNSSVQVGCLTRERLGVNVTWKSRL
Canis_lupus          ARGFCQIQAKTLGTPISIPVCDSSTVQVECVTGERLGVNVTWKLHL
Felis_catus          ARGFCQIQAKTFGSPVSIPVCDGSTVQVECVSGERLGVNVTWKLRL
Sus_scrofa           ARGFCRIQVTTARTPVSIPVCDDSTVRVGCLSLDRLGVNVTWTLRL
Equus_caballus       ARGFCQIQVKTYGTPVSIPVCNDSTVRVECLTQERVGVNVTWKLRL
Orcinus_orca         ARGFCQIQVKMAGTPVSIPVCDDFTVRVECLSGERLGANVTWKLQL
Echinops_telfairi    SRGFCEIQVKTFGSSVSIPVCDESTVQVGCATTERLGVNVTWTSRL
```

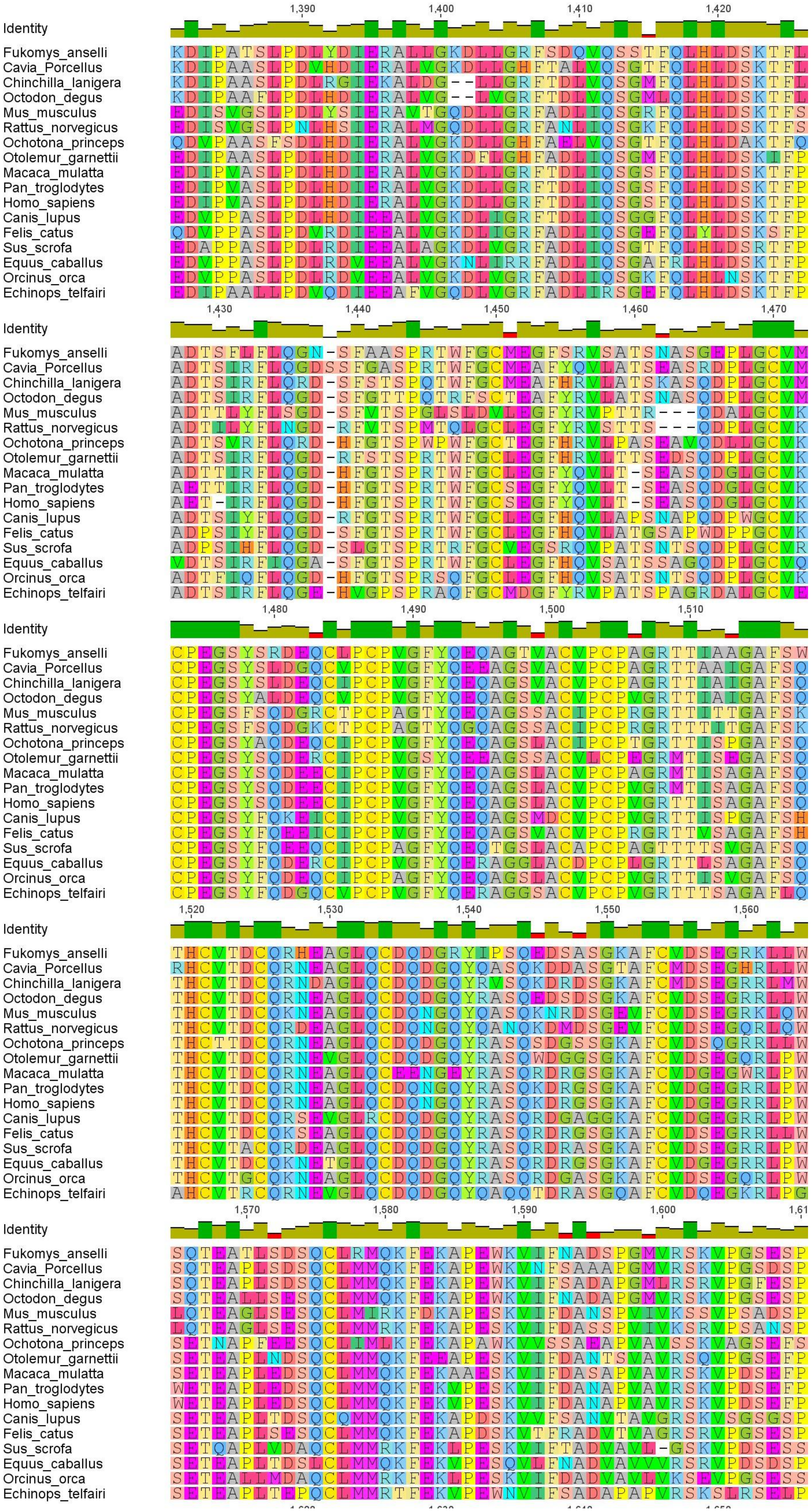
1,390 1,400 1,410 1,420
Identity
Fukomys_anselli KDIPATSLPDLYDIERALLGKDLLGRFSDQVQSSTFQLHLDSKTFL
Cavia_Porcellus KDIPAASLPDVHDIERALVGKDLLGHFTALVQSGTFQLHLDSKTFL
Chinchilla_lanigera KDIPAASLPDLRGIEKALDG--LLGRFTDLVQSGMFQLHLDSKTFL
Octodon_degus KDIPAAFLPDLHDIERALVG--LVGRFTDLVQSGMLQLHLDSKTFL
Mus_musculus EDISVGSLPDLYSIERAVTGQDLLGRFADLIQSGRFQLHLDSKTFS
Rattus_norvegicus EDISVGSLPNLHSIERALMGQDLLGRFANLIQSGKFQLHLDSKTFS
Ochotona_princeps QDVPAASFSDLHDIERALVGKDLLGHFAELVQSGTFQLHLDAKTFQ
Otolemur_garnettii EDIPAASLPDLHDIERALVGKDFLGHFADLIQSGMFQLHLDSKIFP
Macaca_mulatta EDIPVASLPDLHDIERALVGKDLLGRFTDLIQSGSFQLHLDSKTFP
Pan_troglodytes EDIPVASLPDLHDIERALVGKDLLGRFTDLIQSGSFQLHLDSKTFP
Homo_sapiens EDIPVASLPDLHDIERALVGKDLLGRFTDLIQSGSFQLHLDSKTFP
Canis_lupus EDVPPASLPDLHDIEEALVGKDLIGRFTDLIQSGGFQLHLDSKTFP
Felis_catus QDVPPASLPDVRDIEEALVGKDLIGRFADLIQSGEFQLYLDSKSFP
Sus_scrofa EDAPPASLPDLRDIEEALAGKDLVGRFADLIQSGTFQLHLDSRTFP
Equus_caballus EDVPPASLPDLRDVEEALVGKNLIRRFADLIQSGAFRLHLDSKTFP
Orcinus_orca EDVPPASLPDLRDVEEALVGKDLVGRFADLIQSGKFQLHLNSKTFP
Echinops_telfairi EDIPAALLPDVQDIEEAFVGQDLVGRFADLIRSGEFQLHLDSKTFP
1,430 1,440 1,450 1,460 1,470
Identity
Fukomys_anselli ADTSFLFLQGN-SFAASPRTWFGCMEGFSRVSATSNASGEPLGCVM
Cavia_Porcellus ADTSIRFLQGDSSFGASPRTWFGCMEAFYQVLATSEASRDPLGCVM
Chinchilla_lanigera ADTSIRFLQRD-SFSTSPQTWFGCMEAFHRVLATSKASQDPLGCVM
Octodon_degus ADTSIRFLQGD-SFGTTPQTRFSCTEAFYRVLATSNASQDPLGCVM
Mus_musculus ADTTLYFLSGD-SFVTSPGLSLDVLEGFYRVPTTR---QDALGCVK
Rattus_norvegicus ADTILYFLNGD-RFVTSPMTQLGCLEGFYRVSTTS---QDPLGCVK
Ochotona_princeps ADTSVRFLQGD-HFGTSPWPWFGCTEGFHRVLPASEAVQDLLGCVK
Otolemur_garnettii ADTSIRFLQGD-RFSTSPRTWFGCLEGFHRVLTTSEDSQDPLGCVK
Macaca_mulatta ADTTIRFLQGD-HFGTSPRTWFGCLEGFYQVLT-SEASQDGLGCVK
Pan_troglodytes AETTIRFLQGD-HFGTSPRTWFGCSEGFYQVLT-SEASQDGLGCVK
Homo_sapiens AET-IRFLQGD-HFGTSPRTWFGCSEGFYQVLT-SEASQDGLGCVK
Canis_lupus ADTSIYFLQGD-RFGTSPRTWFGCLEGFHQVLAPSNAPQDPWGCVK
Felis_catus ADPSIYFLQGD-SFGTSPRTWFGCLEGFHQVLATGSAPWDPPGCVK
Sus_scrofa ADPSIHFLQGD-SLGTSPRTRFGCVEGSRQVPATSNTSQDPLGCVR
Equus_caballus VDTSIRFIQGA-SFGTSPRTWFGCLEGFHQVSATSSAGQDPLGCVQ
Orcinus_orca ADTFIQFLQGD-HFGTSPRSQFGCLEGFHQVSATSNTSQDPLGCVK
Echinops_telfairi ADTSIRFLQGE-HVGPSPRAQFGCMDGFYRVPATSPAGRDALGCVE
1,480 1,490 1,500 1,510
Identity
Fukomys_anselli CPEGSYSRDEQCLPCPVGFYQEQAGTVACVPCPAGRTTIAAGAFSW
Cavia_Porcellus CPEGSYSLDGQCVPCPVGFYQEEAGSVACVPCPAGRTTAAIGAFSQ
Chinchilla_lanigera CPEGSYSLDEQCIPCPVGFYQEQAGSVACVPCPAGRTTIAIGAFSQ
Octodon_degus CPEGSYALDEQCVPCPVGFYQEQAGSVACVPCPVGRTTIAIGAFSQ
Mus_musculus CPEGSFSQDGRCTPCPAGTYQEQAGSSACIPCPRGRTTITTGAFSK
Rattus_norvegicus CPEGSFSQDGKCTPCPAGTYQGQAGSSACIPCPRGRTTTITGAFSK
Ochotona_princeps CPEGSYAQDEQCIPCPVGFYQEQAGSLACIPCPTGRTTISPGAFSQ
Otolemur_garnettii CPEGSYSQDERCIPCPVGSYQEEAGSSACVLCPEGRMTISEGAFSQ
Macaca_mulatta CPEGSYSQDEECIPCPVGFYQEQAGSLACVPCPAGRMTISAGAFSQ
Pan_troglodytes CPEGSYSQDEECIPCPVGFYQEQAGSLACVPCPVGRMTISAGAFSQ
Homo_sapiens CPEGSYSQDEECIPCPVGFYQEQAGSLACVPCPVGRTTISAGAFSQ
Canis_lupus CPEGSYFQKEICIPCPVGFYQEQAGSMDCVPCPVGRTTISPGAFSH
Felis_catus CPEGSYFQEEICIPCPVGFYQEQAGSVACVPCPRGRTTVSAGAFSH
Sus_scrofa CPEGSYFQEEQCIPCPAGFYQEQTGSLACAPCPAGTTTTSVGAFSQ
Equus_caballus CPEGSYFQDERCIPCPVGFYQERAGGLACDPCPLGRTTLSAGAFSQ
Orcinus_orca CPEGSYFQDEQCIPCPAGFYQEQAGSLACVPCPVGRTTISVGAFSQ
Echinops_telfairi CPEGSYFQDGQCVPCPVGFYQERAGGSACVPCPVGRTTTSAGAFLQ
1,520 1,530 1,540 1,550 1,560
Identity
Fukomys_anselli THCVTDCQRHEAGLQCDQDGRYIPSQEDSASGKAFCVDSEGRKLLW
Cavia_Porcellus RHCVTDCQRNEAGLQCDQDGQYQASQKDDASGTAFCMDSEGHRLLW
Chinchilla_lanigera THCVTDCQRNDAGLQCDQDGQYRVSQKDRDSGKAFCMDSEGRRLMW
Octodon_degus THCVTDCQRNEAGLQCDQDGQYRASQEDSDSGKAFCVDSEGRRLLW
Mus_musculus THCVTDCQKNEAGLQCDQNGQYQASQKNRDSGEVFCVDSEGRKLQW
Rattus_norvegicus THCVTDCQRDEAGLQCDQNGQYQANQKDMDSGEVFCVDSEGQRLQW
Ochotona_princeps THCTTDCQRNEAGLQCDQDGQYRASQSDGSSGKAFCVDSQGRRLLW
Otolemur_garnettii THCVTDCQRNEVGLQCDQDGQYRASQWDGGSGKAFCVDSEGQRLPW
Macaca_mulatta THCVTDCQRNEAGLQCEENGEYRASQRDRGSGKAFCVDGEGWRLPW
Pan_troglodytes THCVTDCQRNEAGLQCDQNGQYRASQKDRGSGKAFCVDGEGRRLPW
Homo_sapiens THCVTDCQRNEAGLQCDQNGQYRASQKDRGSGKAFCVDGEGRRLPW
Canis_lupus THCVTDCQRSEVGLRCDQDGQYRASQRDGAGGKAFCVDGEGRRLPW
Felis_catus THCVTDCQKSEAGLQCDQDGQYRASQRDRGSGKAFCVDSEGRRLLW
Sus_scrofa THCVTACQRDEAGLQCDQDGQYRASQRDRASGKAFCVDSEGRRLPW
Equus_caballus THCVTDCQKNETGLQCDQDGQYRASQRDRGSGKAFCVDGEGRRLPW
Orcinus_orca THCVTGCQKNEAGLQCDQDGQYRASQRDGASGKAFCVDSEGQRLPW
Echinops_telfairi AHCVTRCQRNEVGLQCDQDGQYQAQQTDRASGQAFCVDQEGKRLPG
1,570 1,580 1,590 1,600 1,61
Identity
Fukomys_anselli SQTEATLSDSQCLRMQKFEKAPEWKVIFNADSPGMVRSKVPGSESP
Cavia_Porcellus SQTEAPLSDSQCLMMQKFEKAPEWKVNFSAAAPGMVRSKVPGSDSP
Chinchilla_lanigera SQTEAPLSDSQCLMMQKFEKAPEWKVIFSADAPGMLRSRVPGFESP
Octodon_degus SQTEALLSESQCLMMQKFEKAPEWKVIFNADAPGMVRSKVPGSESP
Mus_musculus LQTEAGLSESQCLMIRKFDKAPESKVIFDANSPVIVKSSVPSADSP
Rattus_norvegicus LQTEAPLSESQCLMMRKFEKAPESKVIFDASSPVIVKSRVPSANSP
Ochotona_princeps SETNAPFEESQCLIMLKFEKAPAWKVVSSAEAPVAVSSKVAGSEFS
Otolemur_garnettii SETEAPLNDSQCLMMQKFEEAPESKVIFDANTSVAVRSQVPGSEFP
Macaca_mulatta SETEAPLEDSQCLMMQKFEKAAESKVIFDASAPVAVRSKVPDSEFP
Pan_troglodytes WETEAPLEDSQCLMMQKFEKVPESKVIFDANAPVAVRSKVPDSEFP
Homo_sapiens WETEAPLEDSQCLMMQKFEKVPESKVVFDANAPVAVRSKVPDSEFP
Canis_lupus SETEAPLTDSQCQMMQKFEKAPDSKVVFSANVTAVGRSKVSGSGSP
Felis_catus SETEAPLSESQCLMMQKFEKAPDSKVTFRADVTVAGRSRVPGSESP
Sus_scrofa SETQAPLVDAQCLMMRKFEKLPESKVIFTADVAVL-GSKVPDSESS
Equus_caballus SETEAPLTDSLCLMMQKFEKVPESQVLFNADVAVVVRSRVPDSDSP
Orcinus_orca SETEALLMDAQCLMMRKFEKLPESKVIFDADVAVLVKSEVPGSESS
Echinops_telfairi SETEAPLTEPQCLMMRTFEKVPEWNVIFSADAPAPVRSKSLRSELP

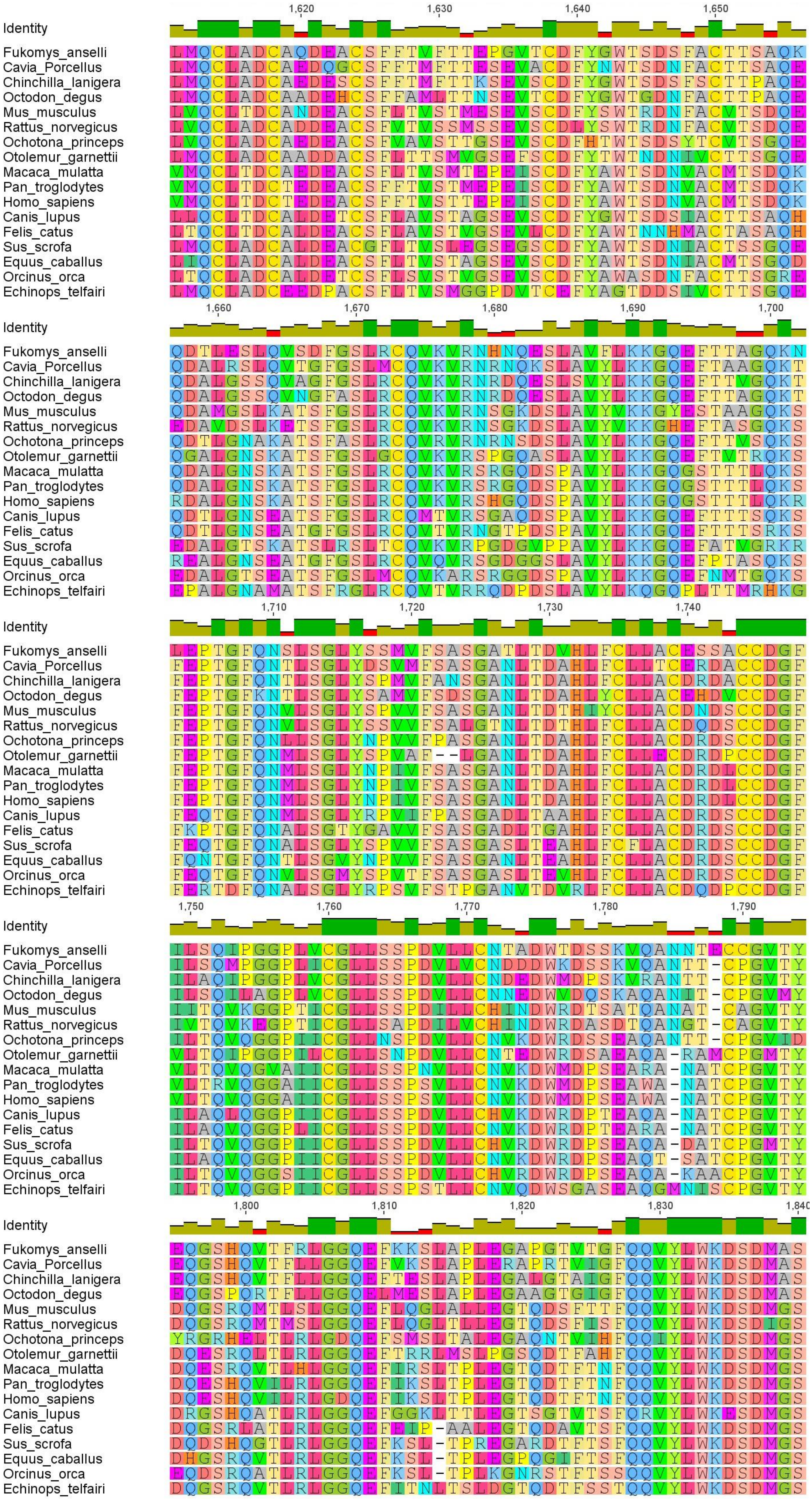
1,620
1,630
1,640
1,650
1,660
1,670
1,680
1,690
1,700
1,710
1,720
1,730
1,740
1,750
1,760
1,770
1,780
1,790
1,800
1,810
1,820
1,830
1,840
Identity
Fukomys_anselli
Cavia_Porcellus
Chinchilla_lanigera
Octodon_degus
Mus_musculus
Rattus_norvegicus
Ochotona_princeps
Otolemur_garnettii
Macaca_mulatta
Pan_troglodytes
Homo_sapiens
Canis_lupus
Felis_catus
Sus_scrofa
Equus_caballus
Orcinus_orca
Echinops_telfairi

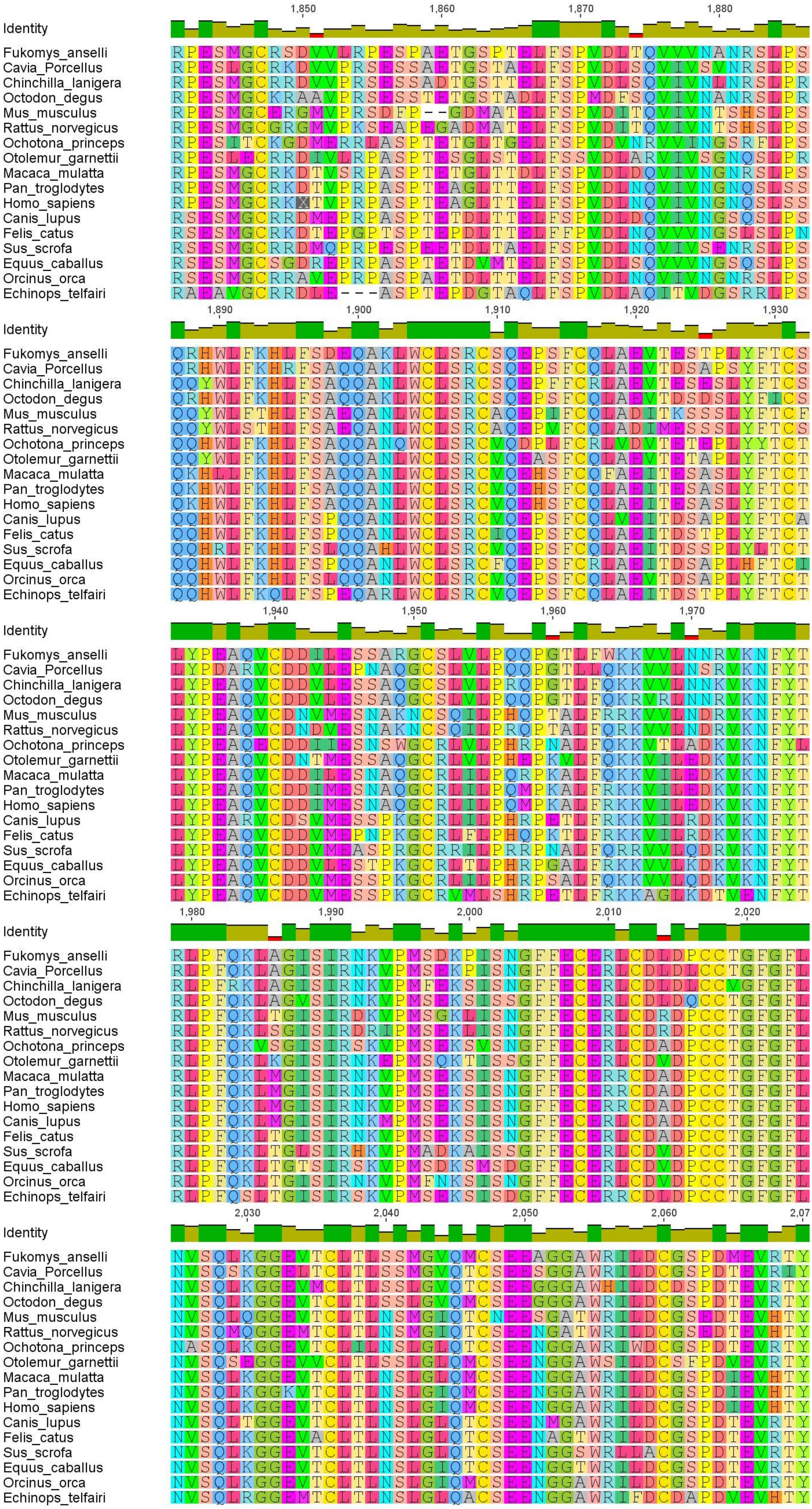
Identity
Fukomys_anselli
Cavia_Porcellus
Chinchilla_lanigera
Octodon_degus
Mus_musculus
Rattus_norvegicus
Ochotona_princeps
Otolemur_garnettii
Macaca_mulatta
Pan_troglodytes
Homo_sapiens
Canis_lupus
Felis_catus
Sus_scrofa
Equus_caballus
Orcinus_orca
Echinops_telfairi

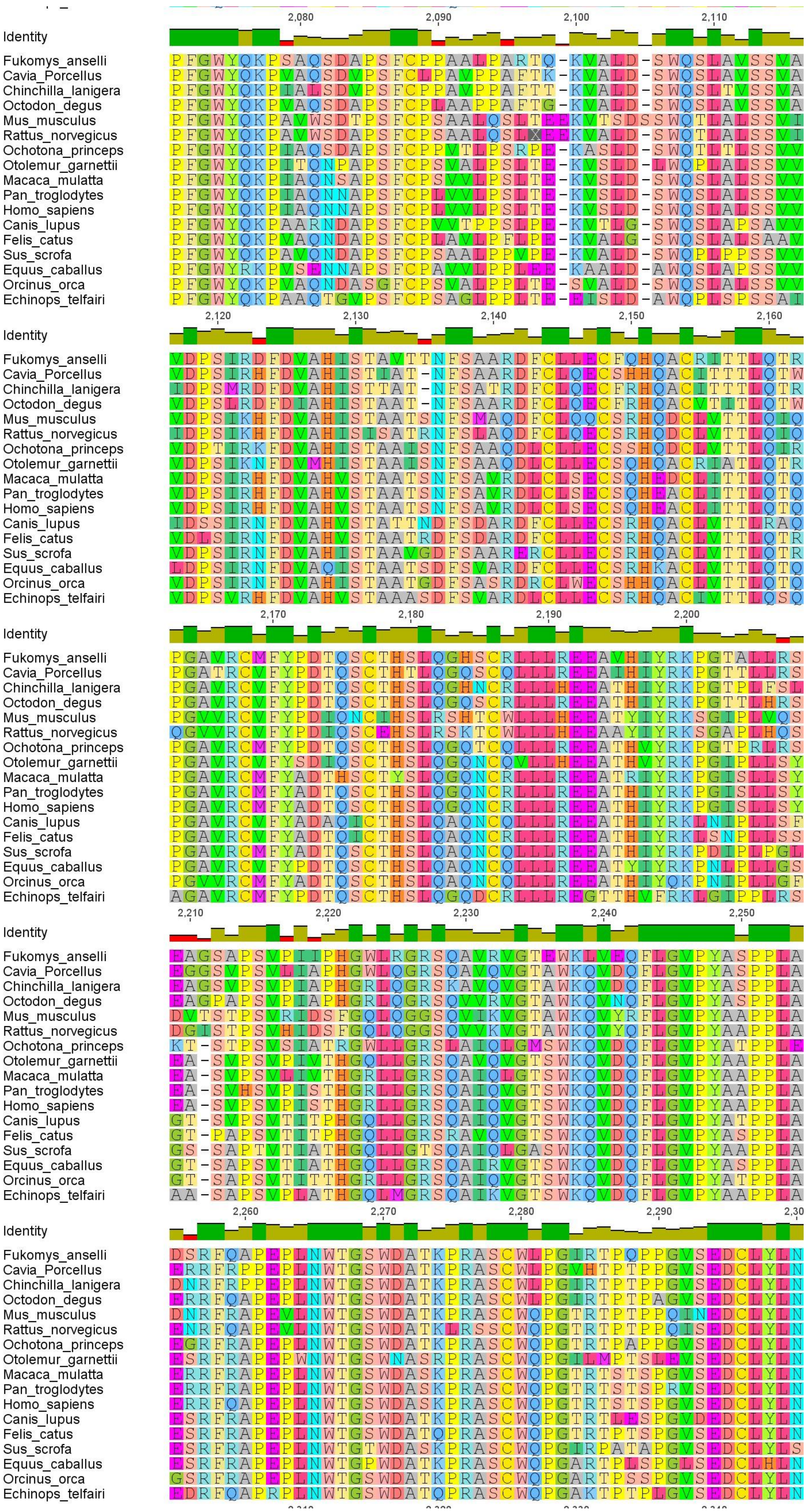
2,080
2,090
2,100
2,110
Identity
Fukomys_anselli
Cavia_Porcellus
Chinchilla_lanigera
Octodon_degus
Mus_musculus
Rattus_norvegicus
Ochotona_princeps
Otolemur_garnettii
Macaca_mulatta
Pan_troglodytes
Homo_sapiens
Canis_lupus
Felis_catus
Sus_scrofa
Equus_caballus
Orcinus_orca
Echinops_telfairi
2,120
2,130
2,140
2,150
2,160
2,170
2,180
2,190
2,200
2,210
2,220
2,230
2,240
2,250
2,260
2,270
2,280
2,290

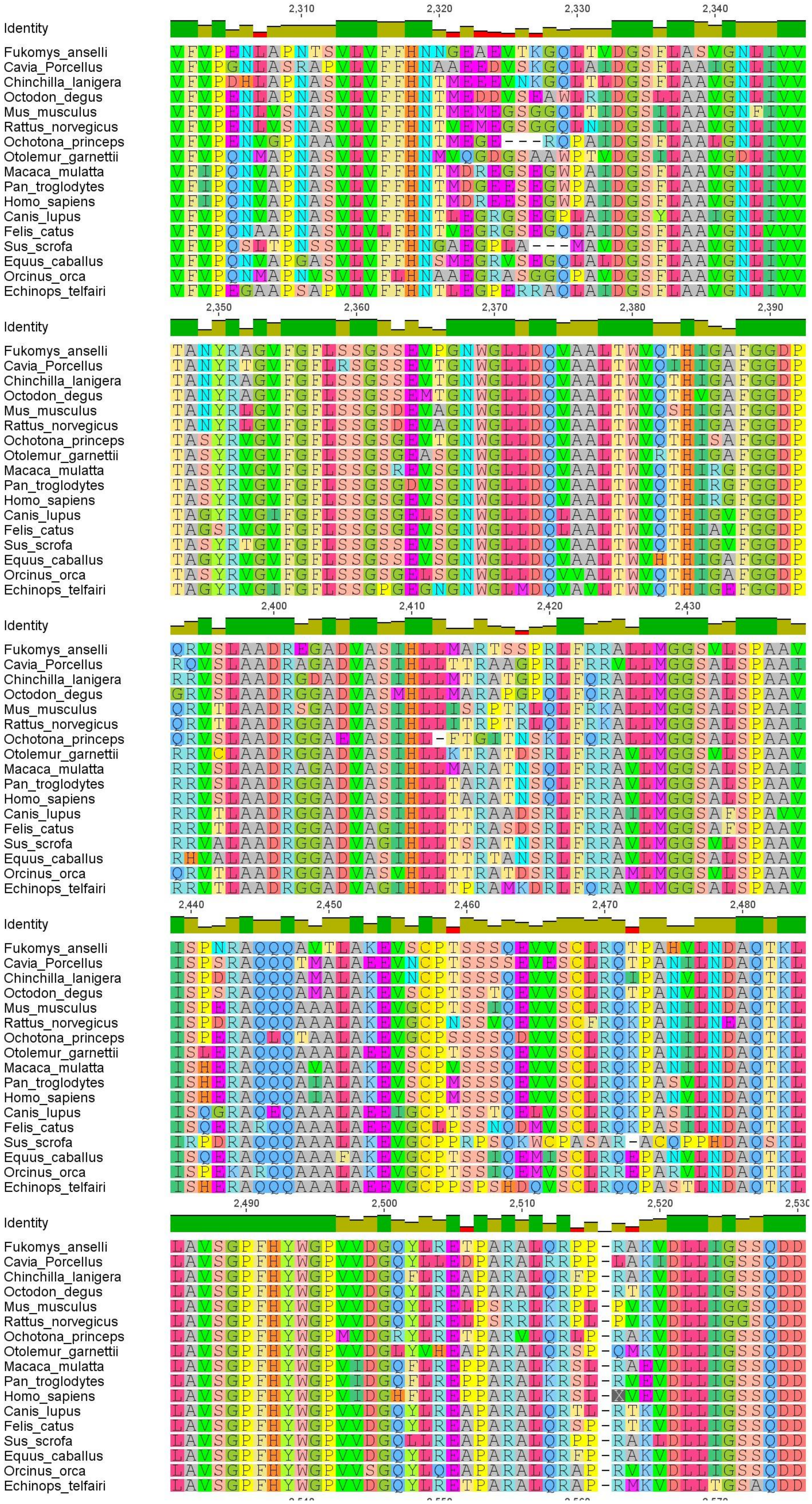
2,310 2,320 2,330 2,340
Identity
Fukomys_anselli VFVPENLAPNTSVLVFFHNNGEAEVTKGQLTVDGSFLASVGNLIVV
Cavia_Porcellus VFVPGNLASRAPVLVFFHNAAEEDVSKGQLAIDGSFLAAIGNLIVV
Chinchilla_lanigera VFVPDHLAPNASVLVFFHNTMEEEVNKGQLTLDGSFLAAVGNLIVV
Octodon_degus VFVPENLAPNASVLVFFHNTMEDDVSEAWLRIDGSLLAAVGNLIVV
Mus_musculus VFVPENLVSNASVLVFFHNTMEMEGSGGQLTIDGSILAAVGNFIVV
Rattus_norvegicus VFVPENLVSNASVLVFFHNTVEMEGSGGQLNIDGSILAAVGNLIVV
Ochotona_princeps VFVPENVGPNAAVLVFFHNTMEGE---RQPAIDGSFLAALGNLIVV
Otolemur_garnettii VFVPQNMAPNASVLVFFHNMVQGDGSAAWPTVDGSILAAVGDLIVV
Macaca_mulatta VFIPQNVAPNASVLVFFHNTMDREGSEGWPAIDGSFLAAVGNLIVV
Pan_troglodytes VFIPQNVAPNASVLVFFHNTMDGEESEGWPAIDGSFLAAVGNLIVV
Homo_sapiens VFIPQNVAPNASVLVFFHNTMDREESEGWPAIDGSFLAAVGNLIVV
Canis_lupus VFVPQNVAPNASVLVFFHNTLEGRGSEGPLAIDGSYLAAIGNLIVV
Felis_catus VFVPQNAAPNASVLVLFHNTVEGRGSEGQLAVDGSFLAAVGNLVVV
Sus_scrofa VFVPQSLTPNSSVLVFFHNGAEGPLA---MAVDGSFLAAVGNLIVV
Equus_caballus VFVPQNVAPGASVLVFFHNSMEGRVSEGQLALDGSFLAAVGNLIVV
Orcinus_orca VFVPQNMAPNVSVLVFLHNAAEGRASGGQPAVDGSFLAAVGNLIVV
Echinops_telfairi VFVPEGAAPSAPVLVFFHNTLEGPERRAQLAIDGSFLAAVGNLIVV
2,350 2,360 2,370 2,380 2,390
Identity
Fukomys_anselli TANYRAGVFGFLSSGSSEVPGNWGLLDQVAALTWVQTHIGAFGGDP
Cavia_Porcellus TANYRTGVFGFLRSGSSEVTGNWGLLDQVAALTWVQIHIGAFGGDP
Chinchilla_lanigera TANYRAGVFGFLSSGSSEVTGNWGLLDQVAALTWVQTHIGAFGGDP
Octodon_degus TANYRAGVFGFLSSGSSEMTGNWGLLDQVAALTWVQTHVGAFGGDP
Mus_musculus TANYRLGVFGFLSSGSDEVAGNWGLLDQVAALTWVQSHIGAFGGDP
Rattus_norvegicus TANYRLGVFGFLSSGSDEVAGNWGLLDQVAALTWVQTHIGAFGGDP
Ochotona_princeps TASYRVGVFGFLSSGSGEVTGNWGLLDQVAALTWVQTHISAFGGDP
Otolemur_garnettii TASYRVGVFGFLSSGSGEASGNWGLLDQVAALTWVRTHIGAFGGDP
Macaca_mulatta TASYRVGVFGFLSSGSREVSGNWGLLDQVAALTWVQTHIRGFGGDP
Pan_troglodytes TASYRVGVFGFLSSGSGDVSGNWGLLDQVAALTWVQTHIRGFGGDP
Homo_sapiens TASYRVGVFGFLSSGSGEVSGNWGLLDQVAALTWVQTHIRGFGGDP
Canis_lupus TAGYRVGIFGFLSSGSGELSGNWGLLDQLAALTWVQTHIGVFGGDP
Felis_catus TAGSRVGVFGFLSSGSGEVSGNWGLLDQLAALTWVQTHIAVFGGDP
Sus_scrofa TASYRTGVFGFLSSGSSEVSGNWGLLDQVAALTWVQTHIGVFGGDP
Equus_caballus TAGYRVGVFGFLSSGSSEVSGNWGLLDQVAALTWVHTHIGAFGGDP
Orcinus_orca TASYRVGVFGFLSSGSGELSGNWGLLDQVVALTWVQTHIGAFGGDP
Echinops_telfairi TAGYRVGIFGFLSSGPGEGNGNWGLMDQVAVLTWVQTHIGEFGGDP
2,400 2,410 2,420 2,430
Identity
Fukomys_anselli QRVSLAADREGADVASIHLLMARTSSPRLFRRALLMGGSVLSPAAV
Cavia_Porcellus RQVSLAADRAGADVASIHLLTTRAAGPRLFRRVLLMGGSALSPAAI
Chinchilla_lanigera RRVSLAADRGDADVASIHLLMTRATGPRLFQRALLMGGSALSPAAV
Octodon_degus GRVSLAADRGGADVASMHLLMARAPGPQLFQRALLMGGSALSPAAV
Mus_musculus QRVTLAADRSGADVASIHLLISRPTRLQLFRKALLMGGSALSPAAI
Rattus_norvegicus QRVTLAADRGGADVASIHLLITRPTRLQLFRKALLMGGSALSPAAI
Ochotona_princeps QRVSLAADRGGAEVASIHL-FTGITNSKLFQRALLMGGSALSPAAV
Otolemur_garnettii RRVCLAADRGGADVASIHLLKTRATDSRLFRRAVLMGGSVLSPAAV
Macaca_mulatta RRVSLAADRAGADVASIHLLMARATNSQLFRRAVLMGGSALSPAAI
Pan_troglodytes RRVSLAADRGGADVASIHLLTARATNSQLFRRAVLMGGSALSPAAV
Homo_sapiens RRVSLAADRGGADVASIHLLTARATNSQLFRRAVLMGGSALSPAAV
Canis_lupus RRVTLAADRGGADVAGIHLLTTRAADSRLFRRAILMGGSAFSPAVV
Felis_catus RRVTLAADRGGADVAGIHLLTTRASDSRLFRRAVLMGGSAFSPAAV
Sus_scrofa RRVALAADRGGADVAGIHLLTSRATNSRLFRRAVLMGGSVLSPAAV
Equus_caballus RHVALAADRGGADVASIHLLTTRTANSRLFRRAVLMGGSALSPAAV
Orcinus_orca QRVTLAADRGGADVASVHLLTTRATDSRLFRRAMLMGGSVLSPAAV
Echinops_telfairi RRVTLAADRGGADVAGIHLLTPRAMKDRLFQRAVLMGGSALSPAAV
2,440 2,450 2,460 2,470 2,480
Identity
Fukomys_anselli ISPNRAQQQAVTLAKEVSCPTSSSQEVVSCLRQTPAHVLNDAQTKL
Cavia_Porcellus ISPSRAQQQTMALAEEVNCPTSSSSEVESCLRQTPANILNDAQTKL
Chinchilla_lanigera ISPDRAQQQAMALAKEVNCPTSSSQEVVSCLRQIPANVLNDAQTKL
Octodon_degus ISPSRAQQQAMALAKEVSCPTSSTQEVVSCLRQTPANVLNDAQTKL
Mus_musculus ISPERAQQQAAALAKEVGCPTSSIQEVVSCLRQKPANILNDAQTKL
Rattus_norvegicus ISPDRAQQQAAALAKEVGCPNSSVQEVVSCFRQKPANILNEAQTKL
Ochotona_princeps ISPERAQLQTAALAKEVGCPSSSSQDVVSCLRQKPANILNDAQTKL
Otolemur_garnettii ISLERAQQQAAALAEEVSCPTSSSQEVVSCLRQKPANILNDAQTKL
Macaca_mulatta ISHERAQQQAVALAKEVSCPVSSSQEVVSCLRQKPANILNDAQTKL
Pan_troglodytes ISHERAQQQAIALAKEVSCPMSSSQEVVSCLRQKPASVLNDAQTKL
Homo_sapiens ISHERAQQQAIALAKEVSCPMSSSQEVVSCLRQKPANVLNDAQTKL
Canis_lupus ISQGRAQEQAAALAEEIGCPTSSTQELVSCLRQKPASILNDAQTKL
Felis_catus ISQERARQQAAALAEEVGCLPSSNQDMVSCLRQKPASILNDAQTKL
Sus_scrofa IRPDRAQQQAAALAKEVGCPPRPSQKWCPASAR-ACQPPHDAQSKL
Equus_caballus ISQERAQQQAAAFAKEVGCPTSSIQEMISCLRQEPANVLNDAQTKL
Orcinus_orca ISPEKARQQAAALAKEVGCPTSSIQEMVSCLRREPARVLNDAQTKL
Echinops_telfairi ISHERAQQQAAALAEEVGCPPSPSHDQVSCLRQQPASTLNDAQTKL
2,490 2,500 2,510 2,520 2,530
Identity
Fukomys_anselli LAVSGPFHYWGPVVDGQYLRETPARALQRPP-RAKVDLLIGSSQDD
Cavia_Porcellus LAVSGPFHYWGPVVDGQYLLEDPARALRRPP-LAKIDLLIGSSQDD
Chinchilla_lanigera LAVSGPFHYWGPVVDGQFLREAPARALQRFP-RAKVDLLIGSSQDD
Octodon_degus LAVSGPFHYWGPVVDGQYLREAPARALQRPP-RTKVDLLIGSSQDD
Mus_musculus LAVSGPFHYWGPVVDGQYLRELPSRRLKRPL-PVKVDLLIGGSQDD
Rattus_norvegicus LAVSGPFHYWGPVVDGQYLRELPSRRLKRPL-PVKVDLLIGGSQDD
Ochotona_princeps LAVSGPFHYWGPMVDGRYLRETPARVLQRLP-RAKVDLLIGSSQDD
Otolemur_garnettii LAVSGPFHYWGPVVDGLYVHEAPARALQRSP-QMKVDLLIGSSQDD
Macaca_mulatta LAVSGPFHYWGPVIDGQFLREPPARALKRSL-RAEVDLLIGSSQDD
Pan_troglodytes LAVSGPFHYWGPVIDGQFLREPPARALKRSL-RVEVDLLIGSSQDD
Homo_sapiens LAVSGPFHYWGPVIDGHFLREPPARALKRSL-XVEVDLLIGSSQDD
Canis_lupus LAVSGPFHYWGPVVDGQYLREAPARALQRTL-RTKVDLLIGSSQDD
Felis_catus LAVSGPFHYWGPVVDGQYLREAPARALQRSP-RTKVDLLIGSSQDD
Sus_scrofa LAVSGPFHYWGPVVDGQLLREAPARALQRPP-RAKLDLLIGSSQDD
Equus_caballus LAVSGPFHYWGPVVDGQYLREAPARALQRFP-RAKVDLLIGSSQDD
Orcinus_orca LAVSGPFHYWGPVVSGQYLQEAPARALQRAP-RVKVDLLIGSSQDD
Echinops_telfairi LAVSGPFHYWGPVVDGQYLRETPARALQRAP-RMKVDLLTGSAQDD

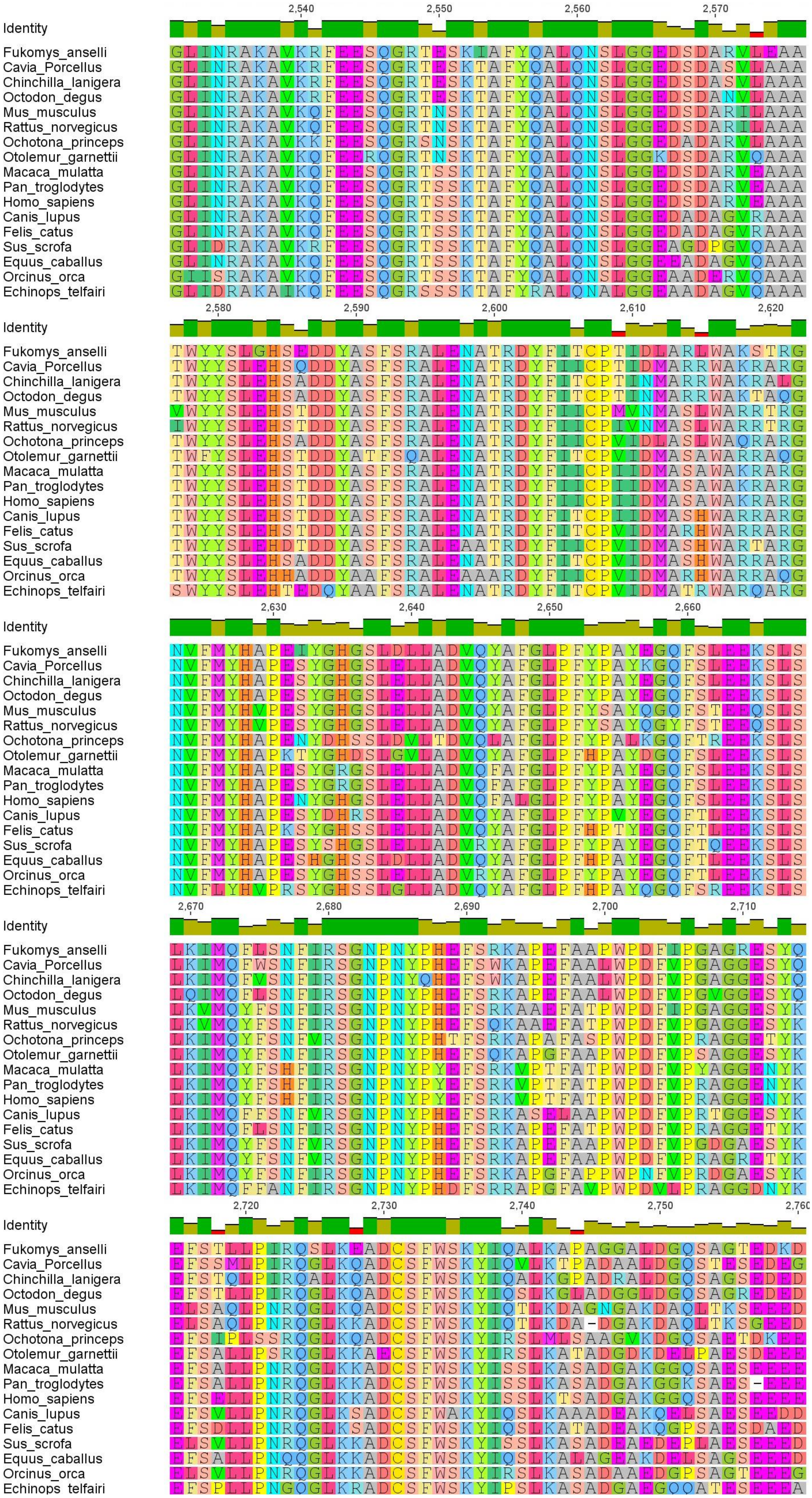
2,540
2,550
2,560
2,570
Identity
Fukomys_anselli
Cavia_Porcellus
Chinchilla_lanigera
Octodon_degus
Mus_musculus
Rattus_norvegicus
Ochotona_princeps
Otolemur_garnettii
Macaca_mulatta
Pan_troglodytes
Homo_sapiens
Canis_lupus
Felis_catus
Sus_scrofa
Equus_caballus
Orcinus_orca
Echinops_telfairi
2,580
2,590
2,600
2,610
2,620
2,630
2,640
2,650
2,660
2,670
2,680
2,690
2,700
2,710
2,720
2,730
2,740
2,750
2,760

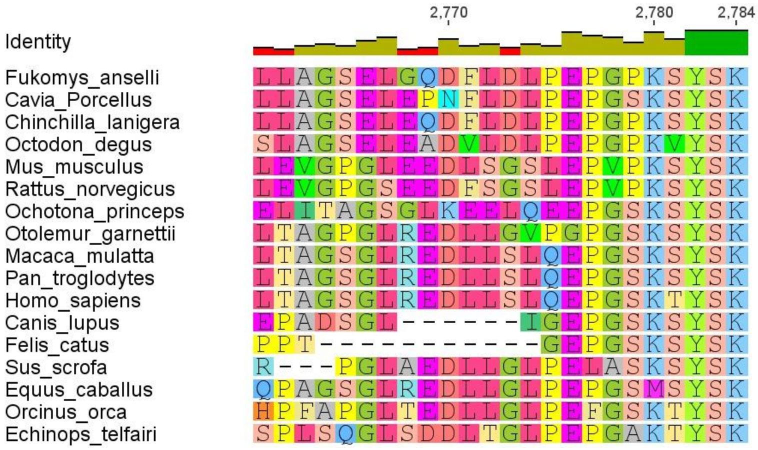

Figure S6. Protein alignment of thyroglobulin (TG) from different mammal species.

The mRNA sequence of *F. anselli* was obtained from RNA-seq and subsequently translated, other sequences were retrieved from NCBI databases with the following accession numbers: Cavia porcellus (XP_003467392), Chinchilla lanigera (XP_005398080), Octodon degus (XP_004642544), Mus musculus (AAB53204), Rattus norvegicus (BAL14775), Ochotona princeps (XP_004580794), Otolemur garnettii (XP_003792914), Macaca mulatta (EHH28780), Pan troglodytes (XP_003311969), Homo sapiens (AAC51924), Canis lupus (XP_005627864), Felis catus (XP_004000173), Sus scrofa (NP_001161890), Equus caballus (XP_001916622), Orcinus orca (XP_004265356), Echinops telfairi (XP_004697442).
