# Supplementary material for: Unusual Ratio between Free Thyroxine and Free Triiodothyronine in a Long-Lived Mole-Rat Species with Bimodal Ageing
Source: PLoS One. 2014 Nov 19;9(11):e113698. doi: 10.1371/journal.pone.0113698 (PMC4237498; doi:10.1371/journal.pone.0113698)
Supplement: Figure S7 — Protein alignment of thyroperoxidase (TPO) from different mammal species. The mRNA sequence of F. anselli was obtained from RNA-seq and subsequently translated, other sequences were retrieved from NCBI databases with the following accession numbers: Cavia porcellus (XP_003464975; patched), Octodon degus (XP_004644658), Mus musculus (EDL36934), Rattus norvegicus (EDM03234), Cricetulus griseus (XP_003501455), Ochotona princeps (XP_004582879), Otolemur garnettii (XP_003798602), Macaca mulatta (XP_001117795), Homo sapiens (XP_005264756), Canis lupus (Q8HYB7), Felis catus (XP_003984594), Bos taurus (XP_603356), Sus scrofa (P09933), Equus caballus (XP_001918216), Orcinus orca (XP_004274968), Echinops telfairi (XP_004709888). (PDF) [file pone.0113698.s007.pdf]

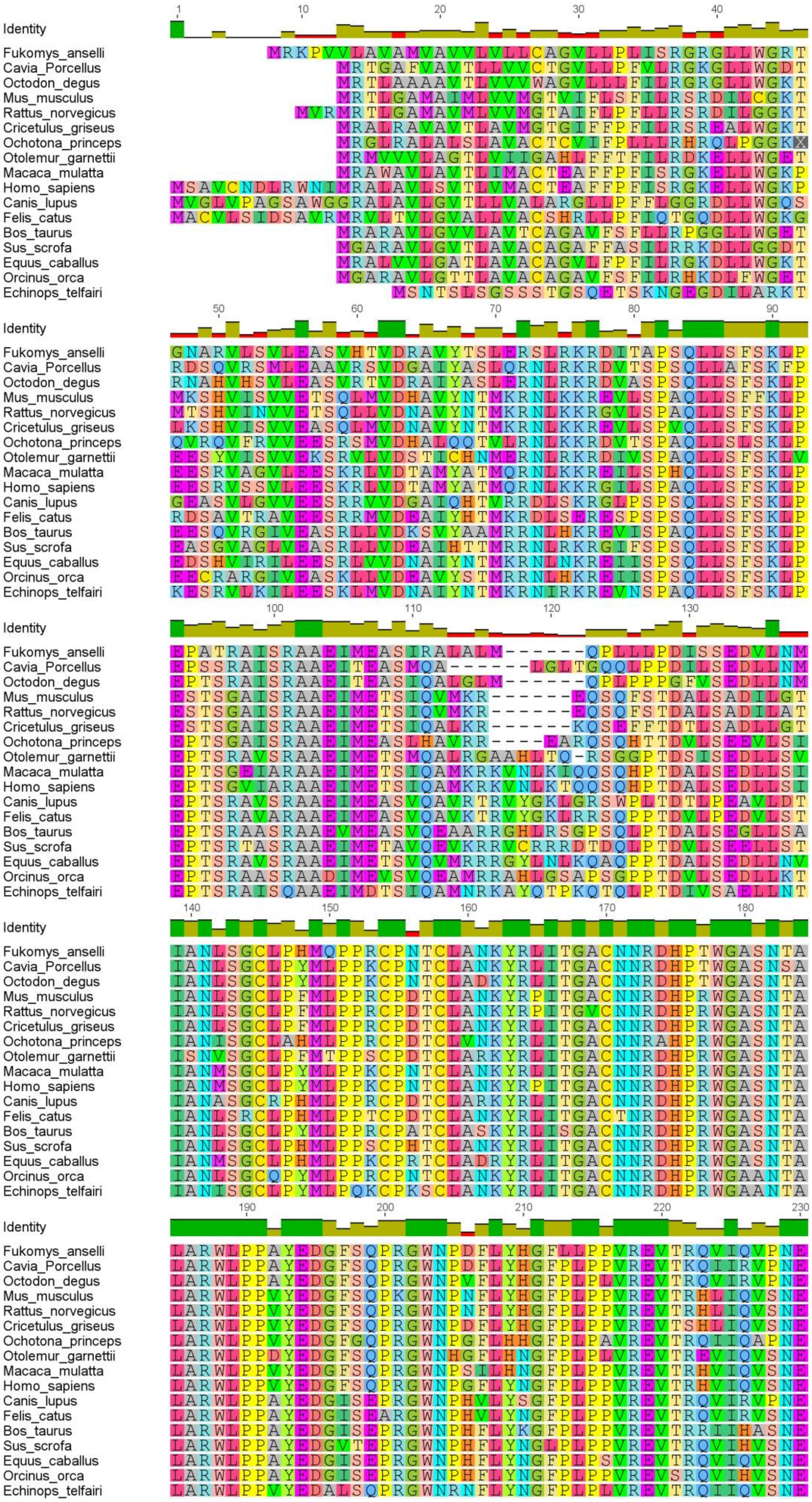
Identity
1
10
20
30
40
50
60
70
80
90
100
110
120
130
140
150
160
170
180
190
200
210
220
230
Fukomys_anselli
Cavia_Porcellus
Octodon_degus
Mus_musculus
Rattus_norvegicus
Cricetulus_griseus
Ochotona_princeps
Otolemur_garnettii
Macaca_mulatta
Homo_sapiens
Canis_lupus
Felis_catus
Bos_taurus
Sus_scrofa
Equus_caballus
Orcinus_orca
Echinops_telfairi

```
                   240       250       260       270
Identity
Fukomys_anselli    AVTEDTQYSDFLTAWGQYIDHDMALTPQSTAA------ADCQLSCK
Cavia_Porcellus    VVTEDDQYSDLLTAWGQYIDHDIALTPQSTS-------ASCQQTCE
Octodon_degus      AVTEDDQYSDFLTVWGQYIDHDIALTPQSTS-------ADCQLTCE
Mus_musculus       AVTEDDQYSDFLPVWGQYIDHDIALTPQSTSTAAFWGGVDCQLTCE
Rattus_norvegicus  AVTEDDQYSDFLPVWGQYIDHDIALTPQSTSTAAFWGGVDCQLTCE
Cricetulus_griseus AVTEDDQYSDFLTVWGQYIDHDIALTPQSTSTTAFWEGVDCQLTCE
Ochotona_princeps  AVTEDERHSDFLTAWGQYISHDIAFTPQSLSRAAFSAGIDCQLTCE
Otolemur_garnettii AVTEDDQYSDLLTVWGQYIAHDLALTPQSTSRAAFGGGADCLLTCE
Macaca_mulatta     VVTDDDRYSDLLMAWGQYIDHDIAFTPQSTSKAAFRGGADCQVTCE
Homo_sapiens       VVTDDDRYSDLLMAWGQYIDHDIAFTPQSTSKAAFGGGADCQMTCE
Canis_lupus        AVTEDDQYSDLLTVWGQYIDHDVAFTPQSASGAAFGAGADCQLTCE
Felis_catus        AVTEDDQYSDLLMVWGQYIDHDIAFTPQSTRKVASEGEADCQLTCK
Bos_taurus         AVTGDDRYSDLLTAWGQYIDHDIAFTPQSAAPSAPWVGADCQLTCE
Sus_scrofa         AVTEDGQYSDLLMAWGQYIDHDIAFTPQSTSKAAFAGGADCQLTCE
Equus_caballus     AVTEDDQYSDLLTAWGQYIDHDIAFTPQSTSRAAFRGGADCQLTCE
Orcinus_orca       AVTEDAQYSDLLMAWGQYTDHDLAFTPQSASHAAFGGGADCQRTCE
Echinops_telfairi  AVTEDGQYSDLLMVWGQYIDHDIAFTPQSTSKAAFWGGIDCQLTCV

                   280       290       300       310       320
Identity
Fukomys_anselli    NQSPCFPIQLPA-----APQGPGCLPFSRSAASCSSGTRGALLANLS
Cavia_Porcellus    NQNPCFPIQLPADV---PPGPRCLPFSRSAAACGLGLRDAFPGNLS
Octodon_degus      SRSACFPIQLPAAD--ASPGPQCLPFSRSAAACGSWPRSALLANLS
Mus_musculus       NQNPCFPIQLPSNSS-GTT--ACLPFYRSSAACGTGDQGALFGNLS
Rattus_norvegicus  NQNPCFPIQLPSNSS-RTT--ACLPFYRSSAACGTGDQGALFGNLS
Cricetulus_griseus NQNPCFPIQLPSNSS-GTT--ACLPFYRSSAACGTGDQGVLFGNLS
Ochotona_princeps  NRSPCFPIQLPGNAS----PAACLPFYRSSPACGTGDQGALFANLS
Otolemur_garnettii NRSPCFPIQLPENTL-LAAGPACLPFYRSSAACGTGDQGALFGNLS
Macaca_mulatta     NQNPCFPIQLPEEAR-PAAGTACLPFYRSSAACGTGDQGALFGNLS
Homo_sapiens       NQNPCFPIQLPEEAR-PAAGTACLPFYRSSAACGTGDQGALFGNLS
Canis_lupus        NRSPCFPIQLPPDAS-GP---ACLPFSRSSAACGTGIQGAFFGNLS
Felis_catus        NQDPCFPIQLPTNGS-GTSGRSCLPFYRSSAACGTGIQGAFFGNLT
Bos_taurus         PRSPCFPIQLPAN-----AAGPACLPFYRSTAACGTGTQGALFGNLS
Sus_scrofa         NRSPCFPIQLPTNAS-GAAGATCLPFYRSSAACGSGRQGALVGNLS
Equus_caballus     TRSPCFPIQLPAADASLTAGPACLPFYRSSAACGTGTQGALFGNVS
Orcinus_orca       NRSPCFPIQLQANAS-PAAGATCLPFYRSSPTCGSGTQGAFFGNVS
Echinops_telfairi  NQNPCFPIQLPLQDS-LTTGAECWPFYRSSAACGTGDQAAVFGNLS

                   330       340       350       360
Identity
Fukomys_anselli    AAATRQQVNALTSFLDASTVYGSSAAAERDLRDWAGAAGRLRVNAR
Cavia_Porcellus    AAAARQQVNALTSFLDASTVYGSSPAAERDLRDWADAAGRLRVNAR
Octodon_degus      VVAARQQVNAFTSFLDASTVYGSSAAAERSLRDWTGAPGRLRVNAR
Mus_musculus       AANPRQQMNGLTSFLDASTVYGSSPGVEKQLRNWSSSAGLLRVNTL
Rattus_norvegicus  AANPRQQMNGLTSFLDASTVYGSSPGVEKQLRNWSSSAGLLRVNTL
Cricetulus_griseus AANPRQQMNGLTSFLDASTVYGSSPGIEKQLRNWSSPAGLLRVNX-
Ochotona_princeps  QANPRQQMNGLTSFLDASTVYGSSPASEKLLRNWTSAEGLLRVNVR
Otolemur_garnettii KANPRQQMNGLTSFLDASTVYGSSPASEKQLRNWTSAEGLLRVNTR
Macaca_mulatta     TANPRQQMNGLTSFLDASTVYGSSPALERQLRNWTSAEGLLRVHAR
Homo_sapiens       TANPRQQMNGLTSFLDASTVYGSSPALERQLRNWTSAEGLLRVHAR
Canis_lupus        SANPRQQMNGLTSFLDASTVYGSSPALEKQLRNWTSAEGLLRVNTR
Felis_catus        SANPRQQMNGLTSFLDASTVYGSSPALEKQLRNWTSAEGLLRVNTR
Bos_taurus         SANPRQQMNGLTSFLDASTVYGSSAASERRLRNWTSAEGLLRVNAR
Sus_scrofa         WAAPRQQMNGLTSFLDASTVYGSSPAQEQRLRNWTSAEGLLRVNTR
Equus_caballus     SAHPRQQMNGLTSFLDASTVYGSSPASEKLLRNWTSAEGLLRVNTR
Orcinus_orca       WANPRQQMNGLTSFLDASTVYGSSPASEQRLRNWTSAEGLLLVNAR
Echinops_telfairi  GSNPRQQMNGLTSFLDASTVYGSTPALEKQLRNWSSEEGQLKVNQR

                   370       380       390       400       410
Identity
Fukomys_anselli    HSDAGRALLPFTPPRALAACAPESESGAPRAPRAPCFLAGDGRASE
Cavia_Porcellus    HHDDGRALLPFTPPRAPAACAPAP--GTPGPTRSPCFLAGDARASE
Octodon_degus      HHDSGRAFLPFTPPSKPAACAPAP--GSPNQTRVPCFLAGDGRASE
Mus_musculus       HLDAGRAYLPFATA----ACAPEP--GTPRTNRTPCFLAGDGRASE
Rattus_norvegicus  HLDSGRAYLPFASA----ACAPEP--GAPHANRTPCFLAGDGRASE
Cricetulus_griseus ---------PFASA----ACAPDP--GAPRATRTPCFLAGDGRASE
Ochotona_princeps  HWDSGRAFLPFAPR-VPAACVPGP--GAPAEARAPCFLAGDGRASE
Otolemur_garnettii HQDAGRAYLPFAPPPAPAVCAPDP--SVPGAPRAPCFLAGDGRASE
Macaca_mulatta     LRDSGRAYLPFAPPRAPAACAPEP--GIPGETRGPCFLAGDGRASE
Homo_sapiens       LRDSGRAYLPFVPPRAPAACAPEP--GIPGETRGPCFLAGDGRASE
Canis_lupus        HWDAGRAHLPFMRPPAPLACVPEP--GTRGTAGAPCFLAGDSRASE
Felis_catus        HWDAGRAYLPFARPPGPTGCAPEP--GTRGTAQAPCFLAGDGRASE
Bos_taurus         YRDAGRAFLPFAPPSAPPACAPQP--GA-PGARAPCFLAGDGRASE
Sus_scrofa         HRDAGRAFLPFAPPPAPPACAPEP--GT-PAARAPCFLAGDSRASE
Equus_caballus     HQDAGRAYLPFVPPPTPSACAPEP--GADPAARAPCFLAGDGRASE
Orcinus_orca       YRDAGRAFLPFAPPPAPPACAARP--GA-PGARAPCFLAGDGRASE
Echinops_telfairi  YRDDGRAYLPFMRPRGPSPCAREP--GAAGAGRIECFQAGDSRASE

                   420       430       440       450       460
Identity
Fukomys_anselli    VPALAALHTLWLREHNRLAAALGALNPHWSSDTVYQEARKVVGALH
Cavia_Porcellus    XPALAALHSLXXXXXXXXXXXXXXXXXXXXXXXXXXXXXXXXXXXX
Octodon_degus      VPGLAALHTLWLREHNRLAAALEALNPHWSADTVYQEARKVVGALH
Mus_musculus       VPALAAVHTLWLREHNRLASAFKAINKHWSANTAYQEARKVVGALH
Rattus_norvegicus  VPALAAVHTLWLREHNRLATAFKAINTHWSANTAYQEARKVVGALH
Cricetulus_griseus VPALAAVHTLWLREHNRLAATFKAINSHWSAETVYQEARKVVGALH
Ochotona_princeps  VPSLTAVHTLWLREHNRLAVKLKALNAHWSADTAYQEARKVVGALH
Otolemur_garnettii APSLTAVHTLWLREHNRLALALKALNAHWSADTVYQEARKVVGALH
Macaca_mulatta     VPSLTALHTLWLREHNRLAAALKALNAHWSADAVYQEARKVVGALH
Homo_sapiens       VPSLTALHTLWLREHNRLAAALKALNAHWSADAVYQEARKVVGALH
Canis_lupus        VPTLAALHTLWLREHNRLASALKALNAHWSADTAYQEARKVVGALH
Felis_catus        VPALTALHTLWLREHNRLAVALKALNAHWSANTAYQEARKVVGALH
Bos_taurus         VPALAALHTLWLREHNRLATALKALNAHWSADTAYQEARKVVGALH
Sus_scrofa         VPGLTALHTLWLREHNRLAAAFKALNAHWSADTVYQEARKVVGALH
Equus_caballus     IPSLAAVHTLWLREHNRVAAALKXLNAHXAPTPAYQDARKILGCTA
Orcinus_orca       VPALAALHTLWLREHNRLAAALKALNAHWSADTAYQEARKVVGALH
Echinops_telfairi  VLSLTALHTLWLREHNRLAAALKALNPHWSADTVYQEARKIVGALH
```

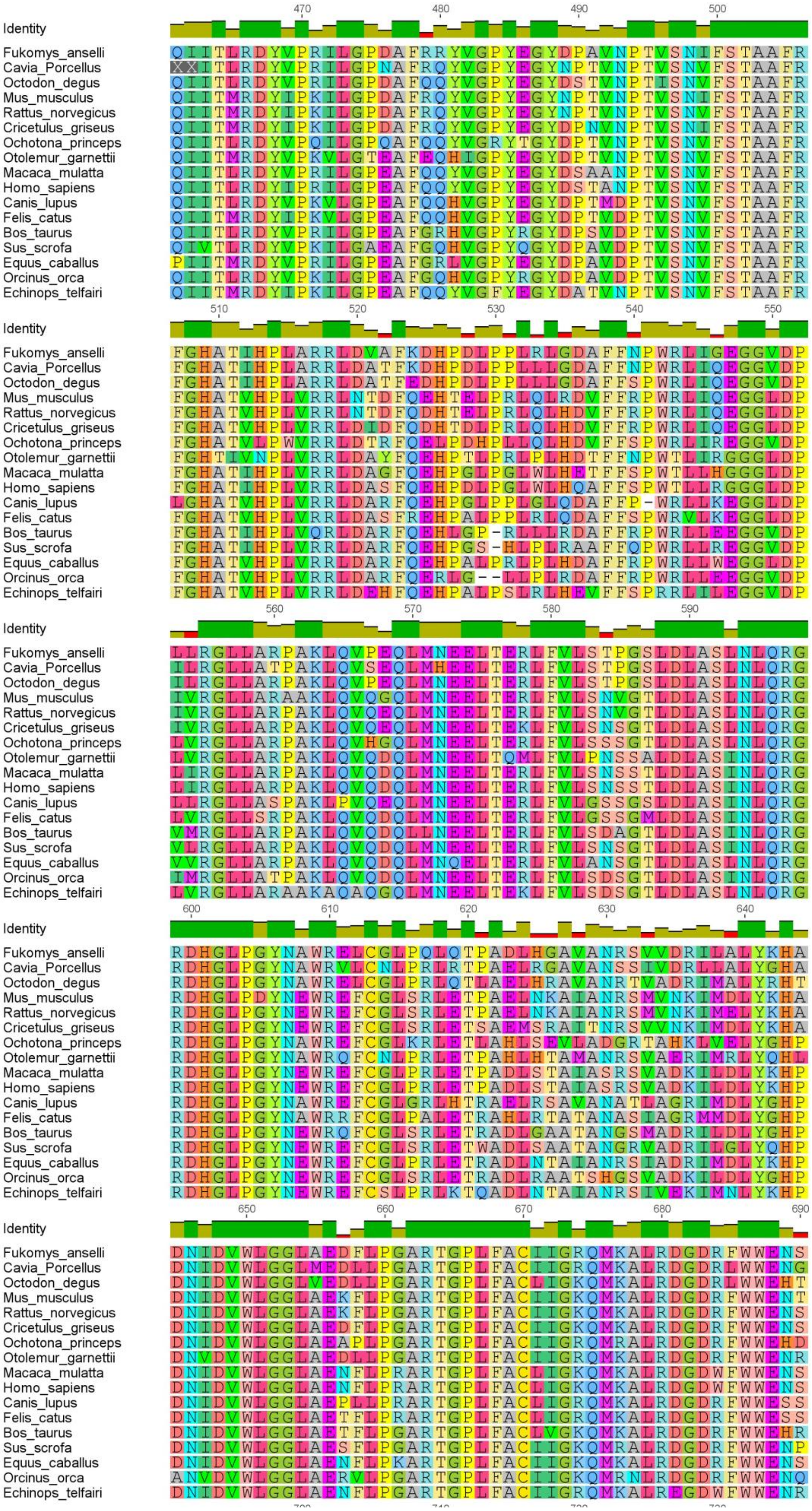
470 480 490 500
Identity
Fukomys_anselli QIITLRDYVPRILGPDAFRRYVGPYEGYDPAVNPTVSNIFSTAAFR
Cavia_Porcellus XXITLRDYVPRILGPNAFRQYVGPYEGYNPTVNPTVSNVFSTAAFR
Octodon_degus QIITLRDYVPRILGPDAFQQYVGPYEGYDSTVNPTISNVFSTAAFR
Mus_musculus QIITMRDYIPKILGPDAFRQYVGPYEGYNPTVNPTVSNIFSTAAFR
Rattus_norvegicus QIITMRDYIPKILGPDAFRQYVGPYEGYNPTVNPTVSNVFSTAAFR
Cricetulus_griseus QIITMRDYIPKILGPDAFRQYVGPYEGYDPNVNPTVSNIFSTAAFR
Ochotona_princeps QIITLRDYVPQILGPQAFQQYVGRYTGYDPTVNPTVSNVFSTAAFR
Otolemur_garnettii QIITMRDYVPKVLGTEAFEQHIGPYEGYDPTVNPTVSNVFSTAAFR
Macaca_mulatta QIITLRDYVPRILGPEAFQQYVGPYEGYDSAANPTVSNVFSTAAFR
Homo_sapiens QIITLRDYIPRILGPEAFQQYVGPYEGYDSTANPTVSNVFSTAAFR
Canis_lupus QIITLRDYVPKVLGPEAFQQHVGPYEGYDPTMDPTVSNVFSTAAFR
Felis_catus QIITMRDYIPKVLGPEAFQQHVGPYEGYDPTVDPTVSNVFSTAAFR
Bos_taurus QIITLRDYVPRILGPEAFGRHVGPYRGYDPSVDPTVSNVFSTAAFR
Sus_scrofa QIVTLRDYVPKILGAEAFGQHVGPYQGYDPAVDPTVSNVFSTAAFR
Equus_caballus PIITMRDYVPRILGPEAFGRLVGPYEGYDPAVDPTVSNVFSTAAFR
Orcinus_orca QIITLRDYVPKILGPEAFGQHVGPYRGYDPAVDPTVSNVFSTAAFR
Echinops_telfairi QIITMRDYIPKILGPEAFQQYVGFYEGYDATVNPTVSNVFSTAAFR
510 520 530 540 550
Identity
Fukomys_anselli FGHATIHPLARRLDVAFKDHPDLPPLRLGDAFFNPWRLIGEGGVDP
Cavia_Porcellus FGHATIHPLARRLDATFKDHPDLPPLLLGDAFFNPWRLIQEGGVDP
Octodon_degus FGHATIHPLARRLDATFEDHPDLPPLLLGDAFFSPWRLIQEGGVDP
Mus_musculus FGHATVHPLVRRLNTDFQEHTELPRLQLRDVFFRPWRLIQEGGLDP
Rattus_norvegicus FGHATVHPLVRRLNTDFQDHTELPRLQLHDVFFRPWRLIQEGGLDP
Cricetulus_griseus FGHATVHPLVRRLDIDFQDHTDLPRLQLHDVFFRPWRLIQEGGLDP
Ochotona_princeps FGHATVLPWVRRLDTRFQELPDHPLLQLHDVFFSPWRLIREGGVDP
Otolemur_garnettii FGHTIVNPLVRRLDAYFQEHPTLPRLPLHDTFFNPWTLIRGGGLDP
Macaca_mulatta FGHATIHPLVRRLDAGFQEHPGLPGLWLHETFFSPWTLLHGGGLDP
Homo_sapiens FGHATIHPLVRRLDASFQEHPDLPGLWLHQAFFSPWTLLRGGGLDP
Canis_lupus LGHATVHPLVRRLDARFQEHPGLPPLGLQDAFFP-WRLLKEGGLDP
Felis_catus FGHATVHPLVRRLDASFREHPALPPLRLQDAFFSPWRVLKEGGLDP
Bos_taurus FGHATIHPLVQRLDARFQEHLGP-RLLLRDAFFRPWRLLEEGGVDP
Sus_scrofa FGHATIHPLVRRLDARFQEHPGS-HLPLRAAFFQPWRLLREGGVDP
Equus_caballus FGHATVHPLVRRLDARFQEHPALPRLPLHDAFFRPWRLLWEGGLDP
Orcinus_orca FGHATVHPLVRRLDARFQERLG--LLPLRDAFFRPWRLLEEGGVDP
Echinops_telfairi FGHATVHPLVRRLDEHFQEHPALPSLRLHEVFFSPRRLILEGGVDP
560 570 580 590
Identity
Fukomys_anselli LLRGLLARPAKLQVPEQLMNEELTERLFVLSTPGSLDLASLNLQRG
Cavia_Porcellus ILRGLLATPAKLQVSEQLMHEELTERLFVLSTPGSLDLASLNLQRG
Octodon_degus ILRGLLARPAKLQVPEQLMNEELTERLFVLSTPGSLDLASLNLQRG
Mus_musculus IVRGLLARAAKLQVQGQLMNEELTERLFVLSNVGTLDLASLNLQRG
Rattus_norvegicus IVRGLLARPAKLQVQEQLMNEELTERLFVLSNVGTLDLASLNLQRG
Cricetulus_griseus IVRGLLARPAKLQVQEQLMNEELTEKLFVLSNSGTLDLASLNLQRG
Ochotona_princeps LVRGLLARPAKLQVHGQLMNEELTERLFVLSSSGTLDLASLNLQRG
Otolemur_garnettii LVRGLLARPAKLQVQDQLMNEELTQMLFVLPNSSALDLASINLQRG
Macaca_mulatta LIRGLLARPAKLQVQDQLMNEELTERLFVLSNSSTLDLASINLQRG
Homo_sapiens LIRGLLARPAKLQVQDQLMNEELTERLFVLSNSSTLDLASINLQRG
Canis_lupus LLRGLLASPAKLPVQEQLMNEELTERLFVLGSSGSLDLASINLQRG
Felis_catus LVRGLLSRPAKLQVQDQLMNEELTERLFVLGSSGMLDLASINLQRG
Bos_taurus VMRGLLARPAKLQVQDQLLNEELTERLFVLSDAGTLDLASINLQRG
Sus_scrofa VLRGLLARPAKLQVQDQLMNEELTERLFVLSNSGTLDLASINLQRG
Equus_caballus VVRGLLARPAKLQVQDQLMNQELTERLFVLANSGTLDLASLNLQRG
Orcinus_orca IMRGLLATPAKLQVQDQLMNEELTERLFVLSDSGTLDLASINLQRG
Echinops_telfairi LVRGLLARAAKAQAQGQLMNEELTEKLFVLSDSGTLDLASLNLQRG
600 610 620 630 640
Identity
Fukomys_anselli RDHGLPGYNAWRELCGLPQLQTPADLHGAVANRSVVDRILALYKHA
Cavia_Porcellus RDHGLPGYNAWRVLCNLPRLRTPAELRGAVANSSIVDRLLALYGHA
Octodon_degus RDHGLPGYNAWRELCGLPRLQTLAELHRAVANRTVADRIMALYRHT
Mus_musculus RDHGLPDYNEWREFCGLSRLETPAELNKAIANRSMVNKIMDLYKHA
Rattus_norvegicus RDHGLPGYNEWREFCGLSRLETPAELNKAIANRSMVNKIMELYKHA
Cricetulus_griseus RDHGLPGYNEWREFCGLSRLETSAEMSRAITNRSVVNKIMDLYKHA
Ochotona_princeps RDHGLPGYNAWREFCGLKRLETLAHLSEVLADGRTAHKLVELYGHP
Otolemur_garnettii RDHGLPGYNAWRQFCNLPRLETPAHLHTAMANRSVAERIMRLYQHL
Macaca_mulatta RDHGLPGYNEWREFCGLPRLETPADLSTAIASRSVADKILDLYKHP
Homo_sapiens RDHGLPGYNEWREFCGLPRLETPADLSTAIASRSVADKILDLYKHP
Canis_lupus RDHGLPGYNAWREFCGLGRLHTRAELRSAVANATLAGRIMDLYGHP
Felis_catus RDHGLPGYNAWRRFCGLPALETRAHLRTATANASIAGRMMDLYGHP
Bos_taurus RDHGLPGYNEWRQFCGLSRLETRADLGAATANGSMADRILDLYGHP
Sus_scrofa RDHGLPGYNEWREFCGLSRLETWADLSAATANGRVADRILGLYQHP
Equus_caballus RDHGLPGYNEWREFCGLPRLETRADLNTAIANRSIADRIMDLYKHP
Orcinus_orca RDHGLPGYNEWREFCGLSRLETRADLRAATSHGSVADKILDLYGHP
Echinops_telfairi RDHGLPGYNEWREFCSLPRLKTQADLNTAIANRSIVEKIMNLYKHP
650 660 670 680 690
Identity
Fukomys_anselli DNIDVWLGGLAEDFLPGARTGPLFACIIGRQMKALRDGDRFWWENS
Cavia_Porcellus DNIDVWLGGLMEDLLPGARTGPLFACIIGRQMKALRDGDRLWWENG
Octodon_degus DNIDVWLGGLVEDLLPGARTGPLFACLIGKQMKALRDGDRLWWEHT
Mus_musculus DNIDVWLGGLAEKFLPGARTGPLFACIIGKQMKALRDGDRFWWENT
Rattus_norvegicus DNIDVWLGGLAEKFLPGARTGPLFACIIGKQMKALRDGDRFWWENS
Cricetulus_griseus DNIDVWLGGLAEDFLPGARTGPLFACIIGKQMKALRDGDRFWWENS
Ochotona_princeps DNIDVWLGGLAEAPLPGARTGPLFACIIGRQMRALRDGDRFWWEHD
Otolemur_garnettii DNVDVWLGGLAEDLLPGARTGPLFACIIGRQMKALRDGDRFWWENR
Macaca_mulatta DNIDVWLGGLAENFLPRARTGPLFACLIGKQMKALRDGDWFWWENS
Homo_sapiens DNIDVWLGGLAENFLPRARTGPLFACLIGKQMKALRDGDWFWWENS
Canis_lupus DNIDVWLGGLAEPLLPRARTGPLFACLIGRQMKALRDGDRFWWESS
Felis_catus DNIDVWLGGLAETFLPRARTGPLFACLIGRQMKALRDGDRFWWESS
Bos_taurus DNIDVWLGGLAETFLPGARTGPLFACLVGKQMKALRDGDRFWWEHR
Sus_scrofa DNIDVWLGGLAESFLPGARTGPLFACIIGKQMRALRDGDRFWWENP
Equus_caballus DNIDVWLGGLAENFLPKARTGPLFACIIGKQMKALRDGDRFWWENS
Orcinus_orca ANVDVWLGGLAERVLPGARTGPLFACIIGKQMRNLRDGDRFWWENQ
Echinops_telfairi DNIDVWLGGLAENFLPGARTGPLFACIIGRQMKALREGDWFWWENR

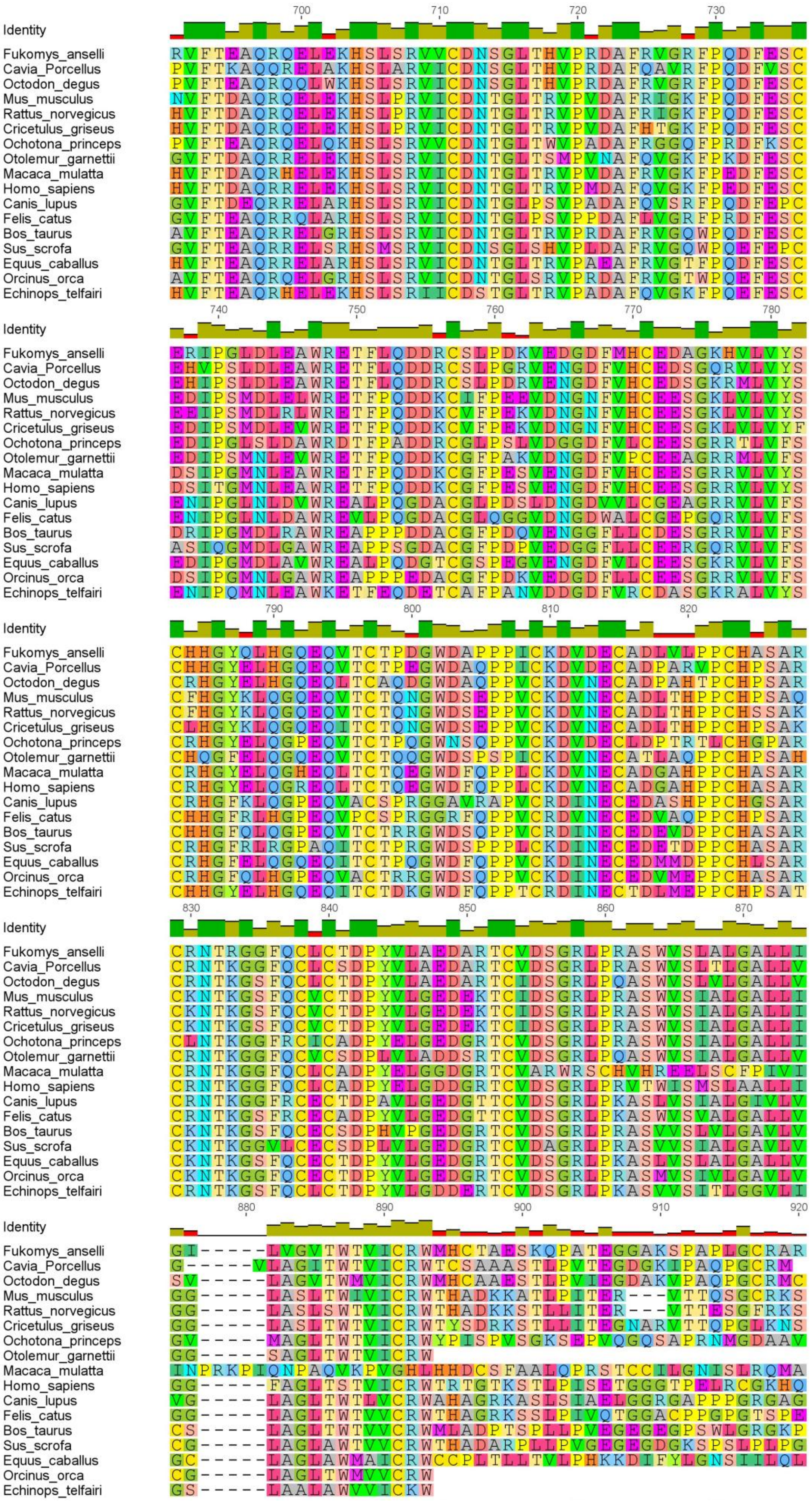

700 710 720 730
Identity
Fukomys_anselli RVFTEAQRQELEKHSLSRVVCDNSGLTHVPRDAFRVGRFPQDFESC
Cavia_Porcellus PVFTKAQQRELAKHSLARVICDNSGLTHVPRDAFQAVRFPQDFVSC
Octodon_degus PVFTEAQRQQLWKHSLSRVICDNSGLTHVPRDAFRVGRFPQDFESC
Mus_musculus NVFTDAQRQELEKHSLPRVICDNTGLTRVPVDAFRIGKFPQDFESC
Rattus_norvegicus HVFTDAQRQELEKHSLPRVICDNTGLTRVPVDAFRIGKFPQDFESC
Cricetulus_griseus HVFTDAQRQELEKHSLPRVICDNTGLTRVPVDAFHTGKFPQDFESC
Ochotona_princeps PVFTEAQRQELQKHSLSRVVCDNTGLTWVPADAFRGGQFPRDFKSC
Otolemur_garnettii GVFTDAQRRELEKHSLSRVICDNTGLTSMPVNAFQVGKFPKDFESC
Macaca_mulatta HVFTDAQRHELEKHSLSRVICDNTGLTRVPVDAFRVGKFPEDFESC
Homo_sapiens HVFTDAQRRELEKHSLSRVICDNTGLTRVPMDAFQVGKFPEDFESC
Canis_lupus GVFTDEQRRELARHSLSRVICDNTGLPSVPADAFQVSRFPQDFEPC
Felis_catus GVFTEAQRRQLARHSLSRVICDNTGLPSVPPDAFLVGRFPRDFESC
Bos_taurus AVFTEAQRRELGRHSLSRVICDNTGLTRVPRDAFRVGQWPQDFESC
Sus_scrofa GVFTEAQRRELSRHSMSRVICDNSGLSHVPLDAFRVGQWPQEFEPC
Equus_caballus HVFTEAQRRELARHSLSRVICDNTGLTRVPAEAFRVGTFPQDFESC
Orcinus_orca AVFTEAQRQELGRHSLSRVICDNTGLSRVPRDAFRVGTWPQEFESC
Echinops_telfairi HVFTEAQRHELEKHSLSRIICDSTGLTRVPADAFQVGKFPQEFESC
740 750 760 770 780
Identity
Fukomys_anselli ERIPGLDLEAWRETFLQDDRCSLPDKVEDGDFMHCEDAGKHVLVYS
Cavia_Porcellus EHVPSLDLEAWRETFLQDDRCSLPGRVENGDFVHCEDSGQRVLVYS
Octodon_degus EHIPSLDLEAWRETFLQDDRCSLPDRVENGDFVHCEDSGKRMLVYS
Mus_musculus EDIPSMDLELWRETFPQDDKCIFPEEVDNGNFVHCEESGKLVLVYS
Rattus_norvegicus EEIPSMDLRLWRETFPQDDKCVFPEKVDNGNFVHCEESGKLVLVYS
Cricetulus_griseus EDIPSMDLEVWRETFPQDDKCVFPEKVDNGNFVHCEESGKLVLVYF
Ochotona_princeps EDIPGLSLDAWRDTFPADDRCGLPSLVDGGDFVLCEESGRRTLVFS
Otolemur_garnettii EDIPSMNLEVWRETFPQDDKCGFPAKVDNGDFVPCEEAGRRMLVFS
Macaca_mulatta DSIPGMNLEAWRETFPQDDKCGFPESVENGDFVHCEESGRRVLVYS
Homo_sapiens DSITGMNLEAWRETFPQDDKCGFPESVENGDFVHCEESGRRVLVYS
Canis_lupus ENIPGLNLDVWREALPQGDACGLPDSLDNGDVVLCGEAGRRVLVFS
Felis_catus ENIPGLNLDAWREVLPQGDACGLQGGVDNGDWALCGEPGQRVLVFS
Bos_taurus DRIPGMDLRAWREAPPPDDACGFPDQVENGGFLLCDESGRRVLVFS
Sus_scrofa ASIQGMDLGAWREAPPSGDACGFPDPVEDGGFLLCEERGQRVLVFS
Equus_caballus EDIPGMDLAVWREALPQDGTCGSPEGVENGDFVLCEESGKRVLVFS
Orcinus_orca DSIPGMNLGAWREAPPPEDACGFPDKVEDGDFLLCEESGRRVLVFS
Echinops_telfairi ENIPQMNLEAWKETFEQDETCAFPANVDDGDFVRCDASGKRALVYS
790 800 810 820
Identity
Fukomys_anselli CHHGYQLHGQEQVTCTPDGWDAPPPICKDVDECADLVLPPCHASAR
Cavia_Porcellus CHHGYELHGQEQVTCTPEGWDAQPPICKDVDECADPARVPCHPSAR
Octodon_degus CRHGYELHGQEQLTCAQDGWDAQPPVCKDVNECADPAHTPCHPSAR
Mus_musculus CFHGYKLQGQEQVTCTQNGWDSEPPVCKDVNECADLTHPPCHPSAQ
Rattus_norvegicus CFHGYKLQGQEQVTCTQNGWDSEPPVCKDVNECADLTHPPCHSSAK
Cricetulus_griseus CLHGYKLQGQEQITCTQNGWDSEPPVCKDVNECADLTHPPCHPSAK
Ochotona_princeps CRHGYELQGPEQVTCTPQGWNSQPPVCKDVDECLDPTRTLCHGPAR
Otolemur_garnettii CHQGFELQGQEQVTCTQQGWDSPSPICKDVNECATLAQPPCHPSAH
Macaca_mulatta CRHGYELQGHEQLTCTQEGWDFQPPLCKDVNECADGAHPPCHASAR
Homo_sapiens CRHGYELQGREQLTCTQEGWDFQPPLCKDVNECADGAHPPCHASAR
Canis_lupus CRHGFKLQGPEQVACSPRGGAVRAPVCRDINECEDASHPPCHGSAR
Felis_catus CHHGFRLHGPEQVPCSPRGGRFQPPVCRDVNECEDVAQPPCHPSAR
Bos_taurus CHHGFQLQGPEQVTCTRRGWDSQPPVCRDINECEDEVDPPCHASAR
Sus_scrofa CRHGFRLRGPAQITCTPRGWDSPPPLCKDINECEDETDPPCHASAR
Equus_caballus CRHGFELQGQEQITCTPQGWDFQPPVCKDINECEDMMDPPCHLSAR
Orcinus_orca CRHGFQLHGPEQVACTRRGWDSQPPVCKDINECEDVMEPPCHASAR
Echinops_telfairi CHHGYELHGQEQITCTDKGWDFQPPTCRDINECTDLMEPPCHPSAT
830 840 850 860 870
Identity
Fukomys_anselli CRNTRGGFQCLCTDPYVLAEDARTCVDSGRLPRASWVSLALGALLI
Cavia_Porcellus CRNTKGGFQCLCSDPYVLAEDARTCVDSGRLPRASWVSLTLGALLV
Octodon_degus CRNTKGSFQCLCTDPYVLAEDARTCIDSGRLPQASWVSLVLGALLV
Mus_musculus CKNTKGSFQCVCTDPYVLGEDEKTCIDSGRLPRASWVSIALGALLI
Rattus_norvegicus CKNTKGSFQCVCTDPYVLGEDEKTCIDSGRLPRASWVSIALGALLI
Cricetulus_griseus CKNTKGSFQCVCTDPYVLGEDEKTCIDSGRLPRASWVSIALGALLI
Ochotona_princeps CLNTKGGFRCICADPYELGEDGRTCVDSGRLPRASWVSIALGALLI
Otolemur_garnettii CRNTKGGFQCVCSDPLVLADDSRTCVDSGRLPQASWVSIALGALLV
Macaca_mulatta CRNTKGGFQCLCADPYELGGDGRTCVARWRSCHVHREELSCFPIVI
Homo_sapiens CRNTKGGFQCLCADPYELGDDGRTCVDSGRLPRVTWISMSLAALLI
Canis_lupus CRNTKGGFRCECTDPAVLGEDGTTCVDSGRLPKASLVSIALGIVLV
Felis_catus CRNTKGSFRCECADPYVLGEDGTTCVDSGRLPKASWVSVALGALLV
Bos_taurus CKNTKGSFQCECSDPHVPGEDGRTCVDSGRLPRASVVSLVLGAVLV
Sus_scrofa CKNTKGGVLCECSDPLVLGEDGRTCVDAGRLPRASVVSIALGAVLV
Equus_caballus CKNTKGSFQCECTDPYVLGEDGRTCVDSGRLPKASLVSLALGALLV
Orcinus_orca CKNTKGGFQCECTDPYVLGEDGRTCVDSGRLPRASMVSIVLGAVLV
Echinops_telfairi CRNTKGSFQCLCTDPYVLGDDERTCVDSGRLPKASVVSITLGGVLI
880 890 900 910 920
Identity
Fukomys_anselli GI------LVGVTWTVICRWMHCTAESKQPATEGGAKSPAPLGCRAR
Cavia_Porcellus G------VLAGITWTVICRWTCSAAASTLPVTEGDGKIPAQPGCRM
Octodon_degus SV------LAGVTWMVICRWMHCAAESTLPVIEGDAKVPAQPGCRMC
Mus_musculus GG------LASLTWIVICRWTHADKKATLPITER---VTTQSGCRKS
Rattus_norvegicus GG------LASLSWTVICRWTHADKKSTLLITER---VTTESGFRKS
Cricetulus_griseus GG------LASLTWTVICRWTYSDRKSTLLITEGNARVTTQPGLKNS
Ochotona_princeps GV------MAGLTWTVICRWYPISPVSGKSEPVQGQSAPRNMGDAAV
Otolemur_garnettii GG------SAGLTWTVICRW
Macaca_mulatta INPRKPIQNPAQVKPVGHLHHDCSFAALQPRSTCCILGNISLRQMA
Homo_sapiens GG------FAGLTSTVICRWTRTGTKSTLPISETGGGTPELRCGKHQ
Canis_lupus VG------LAGLTWTLVCRWAHAGRKASLSIAELGGRGAPPPGRGAG
Felis_catus GG------LAGLTWTVVCRWTHAGRKSSLPIVQTGGACPPGPGTSPE
Bos_taurus CS------LAGLTWTVVCRWMLADPTSPLLPVEGEGEGPSWLGRGKP
Sus_scrofa CG------LAGLAWTVVCRWTHADARPLLPVGEGEGDGKSPSLPLPG
Equus_caballus GC------LAGLAWMAICRWCCPLTLLTVLPHKKDIFYLGNSIILQL
Orcinus_orca CG------LAGLTWMVVCRW
Echinops_telfairi GS------LAALAWVVICKW

```
                       930       940       950       960
Identity
Fukomys_anselli        PDMGTSLQSAQHPEKGPACGSQIPLCM
Cavia_Porcellus
Octodon_degus          REAGTSMCSAAALDLEQPPALMLALRARAVTKPRGLLELRNCNEVT
Mus_musculus           QGRGISPHKAAAQDTGQEPASGSRVLLCE
Rattus_norvegicus      QESGISPQKAEVQDAEQEPAYGSRVLLCE
Cricetulus_griseus     QERGISPHKATAEDTGQEPAYGSRVLLCE
Ochotona_princeps      AGAPGRRMCCGDSDGDSVWCLSQETFQFCIHTADHGE
Otolemur_garnettii
Macaca_mulatta         PWGPRKHFYCCPPAEATALCPPSVFFWTHPQKDGERRPSGSQEQPW
Homo_sapiens           AVGTSPQRAAAQDSEQESAGMEGRDTHRLPRAL
Canis_lupus            QDGASGSLVPPLGPQGRTRAVDPTSSRSHVAQGSPA
Felis_catus            TGAQPGACTRGAGQALVLGFPASGPVRVGPAGLIGSADEQVMSPKD
Bos_taurus             RDVCTPPRDASAGLAEQGPACGSQVLLCK
Sus_scrofa             CGNRRDVGAAPALEVEQDLSCGSRGLCE
Equus_caballus         PAPRVIVCELVGPRVPEALLDPWRSPGGGSRPHPEPTPSLVERGPT
Orcinus_orca
Echinops_telfairi
```

```
                       970       980       990       1,000     1,010
Identity
Fukomys_anselli
Cavia_Porcellus
Octodon_degus          IKTDIFKSKTKWKDFCGKIPTENKKATFPEREGRLGPERRRLASHA
Mus_musculus
Rattus_norvegicus
Cricetulus_griseus
Ochotona_princeps
Otolemur_garnettii
Macaca_mulatta         ESTEMNLCLRGLTHYLSSSSRRAPRDEVVPKRTCTPPLVLLGPL
Homo_sapiens
Canis_lupus
Felis_catus            EPSSHGPDGSRSDDPSLALPVHTSVPGEPHRRPGPPVGQDQAS
Bos_taurus
Sus_scrofa
Equus_caballus         STAKLALPMEPSPTQH
Orcinus_orca
Echinops_telfairi
```

```
                       1,020     1,030     1,040     1,050
Identity
Fukomys_anselli
Cavia_Porcellus
Octodon_degus          EAVLAGVGRRRRRLWLRGPRTAITVGNPLVRRQSCELSKSRCRSHR
Mus_musculus
Rattus_norvegicus
Cricetulus_griseus
Ochotona_princeps
Otolemur_garnettii
Macaca_mulatta
Homo_sapiens
Canis_lupus
Felis_catus
Bos_taurus
Sus_scrofa
Equus_caballus
Orcinus_orca
Echinops_telfairi
```

```
                       1,060     1,069
Identity
Fukomys_anselli
Cavia_Porcellus
Octodon_degus          PAASLSLDHLQ
Mus_musculus
Rattus_norvegicus
Cricetulus_griseus
Ochotona_princeps
Otolemur_garnettii
Macaca_mulatta
Homo_sapiens
Canis_lupus
Felis_catus
Bos_taurus
Sus_scrofa
Equus_caballus
Orcinus_orca
Echinops_telfairi
```

Figure S7. Protein alignment of thyroperoxidase (TPO) from different mammal species.

The mRNA sequence of *F. anselli* was obtained from RNA-seq and subsequently translated, other sequences were retrieved from NCBI databases with the following accession numbers: Cavia porcellus (XP_003464975; patched), Octodon degus (XP_004644658), Mus musculus (EDL36934), Rattus norvegicus (EDM03234), Cricetulus griseus (XP_003501455), Ochotona princeps (XP_004582879), Otolemur garnettii (XP_003798602), Macaca mulatta (XP_001117795), Homo sapiens (XP_005264756), Canis lupus (Q8HYB7), Felis catus (XP_003984594), Bos taurus (XP_603356), Sus scrofa (P09933), Equus caballus (XP_001918216), Orcinus orca (XP_004274968), Echinops telfairi (XP_004709888).
