# Supplementary material for: Unusual Ratio between Free Thyroxine and Free Triiodothyronine in a Long-Lived Mole-Rat Species with Bimodal Ageing
Source: PLoS One. 2014 Nov 19;9(11):e113698. doi: 10.1371/journal.pone.0113698 (PMC4237498; doi:10.1371/journal.pone.0113698)
Supplement: Figure S8 — Protein alignment of transthyretin (TTR) from different mammal species. The mRNA sequence of F. anselli was obtained from RNA-seq and subsequently translated, other sequences were retrieved from NCBI databases with the following accession numbers: Heterocephalus glaber (XP_004905241), Chinchilla lanigera (XP_005372800), Octodon degus (XP_004623610), Rattus norvegicus (AAA41801), Mesocricetus auratus (XP_005065406), Cricetulus griseus (XP_003510202), Ictidomys tridecemlineatus (XP_005337518), Oryctolagus cuniculus (XP_002713532), Chlorocebus aethiops (BAL44398), Homo sapiens (CAG33189), Equus caballus (XP_001495232), Echinops telfairi (XP_004702987). (PDF) [file pone.0113698.s008.pdf]

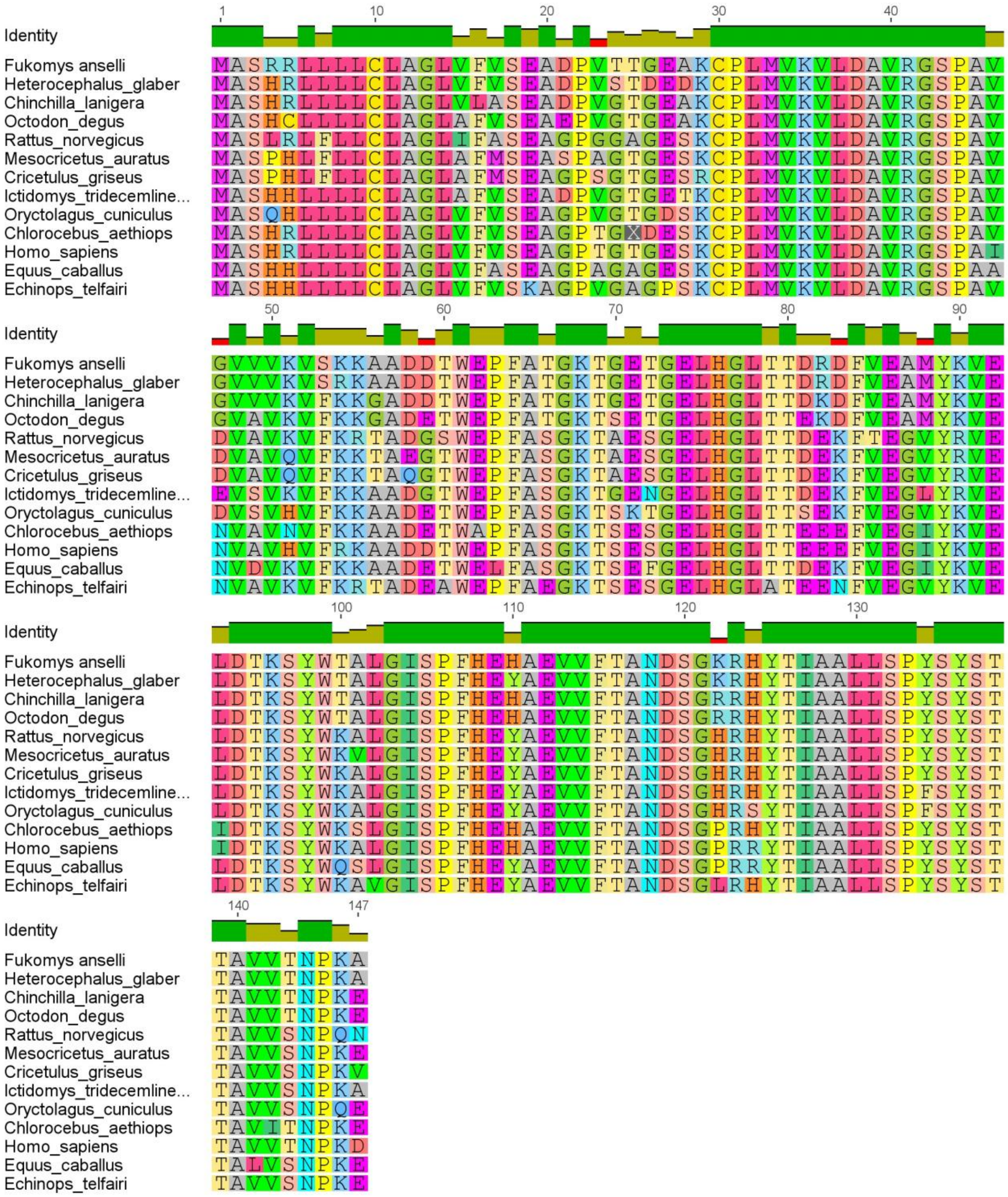
1 10 20 30 40
Identity
Fukomys anselli MASRRLLLLCLAGLVFVSEADPVTTGEAKCPLMVKVLDAVRGSPAV
Heterocephalus_glaber MASHRLLLLCLAGLVFVSEADPVSTDEDKCPLMVKVLDAVRGSPAV
Chinchilla_lanigera MASHRLLLLCLAGLVLASEADPVGTGEAKCPLMVKVLDAVRGSPAV
Octodon_degus MASHCLLLLCLAGLAFVSEAEPVGTGEAKCPLMVKVLDAVRGSPAV
Rattus_norvegicus MASLRLFLLCLAGLIFASEAGPGGAGESKCPLMVKVLDAVRGSPAV
Mesocricetus_auratus MASPHLFLLCLAGLAFMSEASPAGTGESKCPLMVKVLDAVRGSPAV
Cricetulus_griseus MASPHLFLLCLAGLAFMSEAGPSGTGESRCPLMVKVLDAVRGSPAV
Ictidomys_tridecemline... MASHHLLLLCLAGLAFVSEADPVGTGETKCPLMVKVLDAVRGSPAV
Oryctolagus_cuniculus MASQHLLLLCLAGLVFVSEAGPVGTGDSKCPLMVKVLDAVRGSPAV
Chlorocebus_aethiops MASHRLLLLCLAGLVFVSEAGPTGXDESKCPLMVKVLDAVRGSPAV
Homo_sapiens MASHRLLLLCLAGLVFVSEAGPTGTGESKCPLMVKVLDAVRGSPAI
Equus_caballus MASHHLLLLCLAGLVFASEAGPAGAGESKCPLMVKVLDAVRGSPAA
Echinops_telfairi MASHHLLLLCLAGLVFVSKAGPVGAGPSKCPLMVKVLDAVRGSPAV
50 60 70 80 90
Identity
Fukomys anselli GVVVKVSKKAADDTWEPFATGKTGETGELHGLTTDRDFVEAMYKVE
Heterocephalus_glaber GVVVKVSRKAADDTWEPFATGKTGETGELHGLTTDRDFVEAMYKVE
Chinchilla_lanigera GVVVKVFKKGADDTWEPFATGKTGETGELHGLTTDKDFVEAMYKVE
Octodon_degus GVAVKVFKKGADETWEPFATGKTSETGELHGLTTEKDFVEAMYKVE
Rattus_norvegicus DVAVKVFKRTADGSWEPFASGKTAESGELHGLTTDEKFTEGVYRVE
Mesocricetus_auratus DVAVQVFKKTAEGTWEPFASGKTAESGELHGLTTDEKFVEGVYRVE
Cricetulus_griseus DVAVQVFKKTAQGTWEPFASGKTAESGELHGLTTDEKFVEGVYRVE
Ictidomys_tridecemline... EVSVKVFKKAADGTWEPFASGKTGENGELHGLTTDEKFVEGLYRVE
Oryctolagus_cuniculus DVSVHVFKKAADETWEPFASGKTSKTGELHGLTTSEKFVEGVYKVE
Chlorocebus_aethiops NVAVNVFKKAADETWAPFASGKTSESGELHGLTTEEEFVEGIYKVE
Homo_sapiens NVAVHVFRKAADDTWEPFASGKTSESGELHGLTTEEEFVEGIYKVE
Equus_caballus NVDVKVFKKAADETWELFASGKTSEFGELHGLTTDEKFVEGIYKVE
Echinops_telfairi NVAVKVFKRTADEAWEPFAEGKTSESGELHGLATEENFVEGVYKVE
100 110 120 130
Identity
Fukomys anselli LDTKSYWTALGISPFHEHAEVVFTANDSGKRHYTIAALLSPYSYST
Heterocephalus_glaber LDTKSYWTALGISPFHEYAEVVFTANDSGKRHYTIAALLSPYSYST
Chinchilla_lanigera LDTKSYWTALGISPFHEHAEVVFTANDSGRRHYTIAALLSPYSYST
Octodon_degus LDTKSYWTALGISPFHEHAEVVFTANDSGRRHYTIAALLSPYSYST
Rattus_norvegicus LDTKSYWKALGISPFHEYAEVVFTANDSGHRHYTIAALLSPYSYST
Mesocricetus_auratus LDTKSYWKVLGISPFHEYAEVVFTANDSGHRHYTIAALLSPYSYST
Cricetulus_griseus LDTKSYWKALGISPFHEYAEVVFTANDSGHRHYTIAALLSPYSYST
Ictidomys_tridecemline... LDTKSYWKALGISPFHEYAEVVFTANDSGHRHYTIAALLSPFSYST
Oryctolagus_cuniculus LDTKSYWKALGISPFHEYAEVVFTANDSGHRSYTIAALLSPFSYST
Chlorocebus_aethiops IDTKSYWKSLGISPFHEHAEVVFTANDSGPRHYTIAALLSPYSYST
Homo_sapiens IDTKSYWKALGISPFHEHAEVVFTANDSGPRRYTIAALLSPYSYST
Equus_caballus LDTKSYWQSLGISPFHEYAEVVFTANDSGPRRYTIAALLSPYSYST
Echinops_telfairi LDTKSYWKAVGISPFHEYAEVVFTANDSGLRHYTIAALLSPYSYST
140 147
Identity
Fukomys anselli TAVVTNPKA
Heterocephalus_glaber TAVVTNPKA
Chinchilla_lanigera TAVVTNPKE
Octodon_degus TAVVTNPKE
Rattus_norvegicus TAVVSNPQN
Mesocricetus_auratus TAVVSNPKE
Cricetulus_griseus TAVVSNPKV
Ictidomys_tridecemline... TAVVSNPKA
Oryctolagus_cuniculus TAVVSNPQE
Chlorocebus_aethiops TAVITNPKE
Homo_sapiens TAVVTNPKD
Equus_caballus TALVSNPKE
Echinops_telfairi TAVVSNPKE

Figure S8. Protein alignment of transthyretin (TTR) from different mammal species.

The mRNA sequence of *F. anselli* was obtained from RNA-seq and subsequently translated, other sequences were retrieved from NCBI databases with the following accession numbers: Heterocephalus glaber (XP_004905241), Chinchilla lanigera (XP_005372800), Octodon degus (XP_004623610), Rattus norvegicus (AAA41801), Mesocricetus auratus (XP_005065406), Cricetulus griseus (XP_003510202), Ictidomys tridecemlineatus (XP_005337518), Oryctolagus cuniculus (XP_002713532), Chlorocebus aethiops (BAL44398), Homo sapiens (CAG33189), Equus caballus (XP_001495232), Echinops telfairi (XP_004702987).
