# Supplementary material for: Unusual Ratio between Free Thyroxine and Free Triiodothyronine in a Long-Lived Mole-Rat Species with Bimodal Ageing
Source: PLoS One. 2014 Nov 19;9(11):e113698. doi: 10.1371/journal.pone.0113698 (PMC4237498; doi:10.1371/journal.pone.0113698)
Supplement: Figure S9 — Protein alignment of thyroxine-binding globin (TBG) from different mammal species. The mRNA sequence of F. anselli was obtained from RNA-seq and subsequently translated, other sequences were retrieved from NCBI databases with the following accession numbers: Heterocephalus glaber (EHB09876), Octodon degus (XP_004646260), Mus musculus (P61939), Rattus norvegicus (AAA42205), Cricetulus griseus (ERE65740), Otolemur garnettii (XP_003801681), Gorilla gorilla (XP_004064693), Pan troglodytes (NP_001009109), Homo sapiens (NP_783866), Canis lupus (XP_538128), Bos taurus (AAI03464), Ovis aries (NP_001094390), Sus scrofa (Q9TT35), Equus caballus (XP_001493492), Orcinus orca (XP_004285286), Echinops telfairi (XP_004710081). (PDF) [file pone.0113698.s009.pdf]

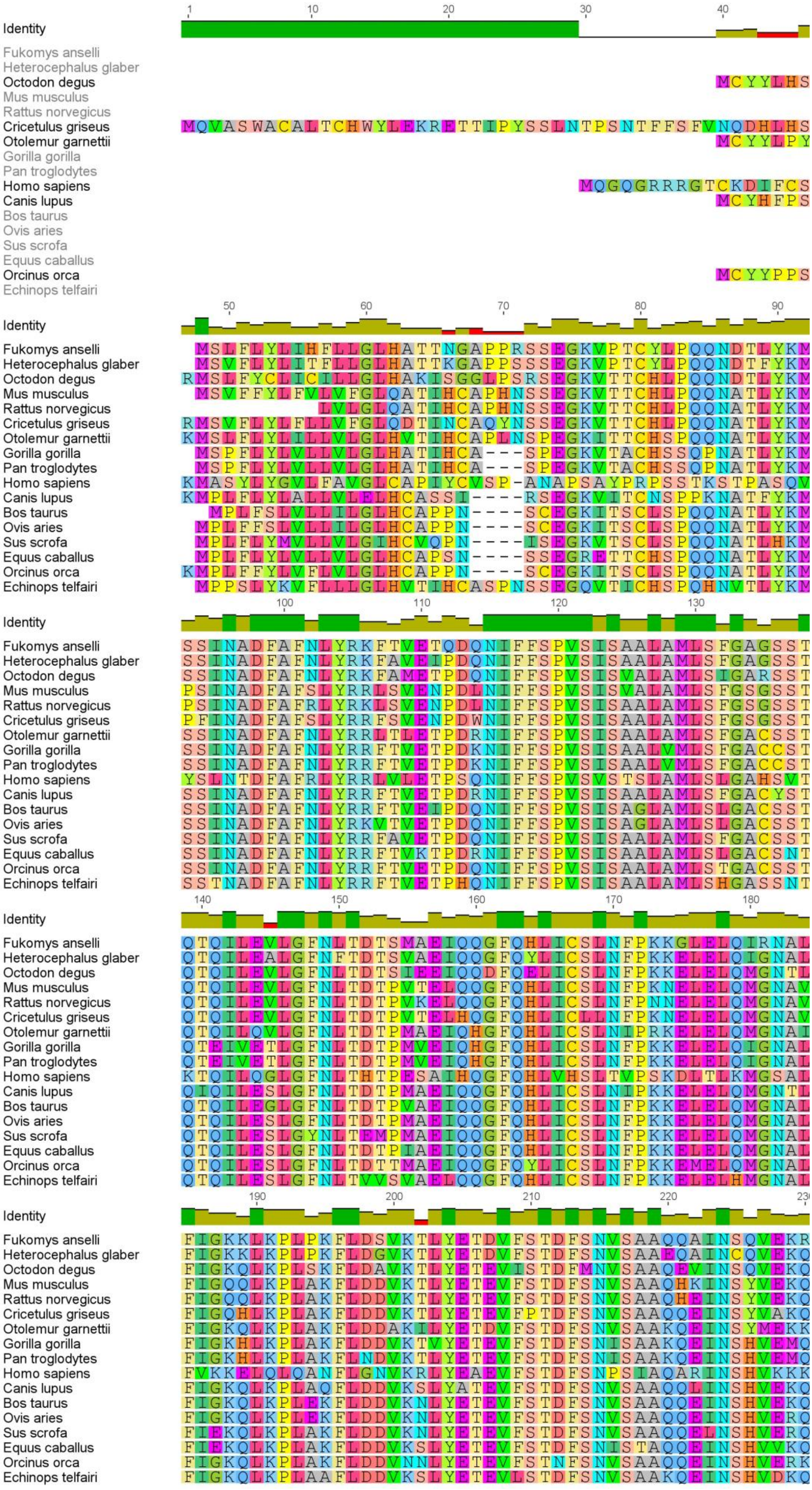

Identity
Fukomys anselli
Heterocephalus glaber
Octodon degus
Mus musculus
Rattus norvegicus
Cricetulus griseus
Otolemur garnettii
Gorilla gorilla
Pan troglodytes
Homo sapiens
Canis lupus
Bos taurus
Ovis aries
Sus scrofa
Equus caballus
Orcinus orca
Echinops telfairi

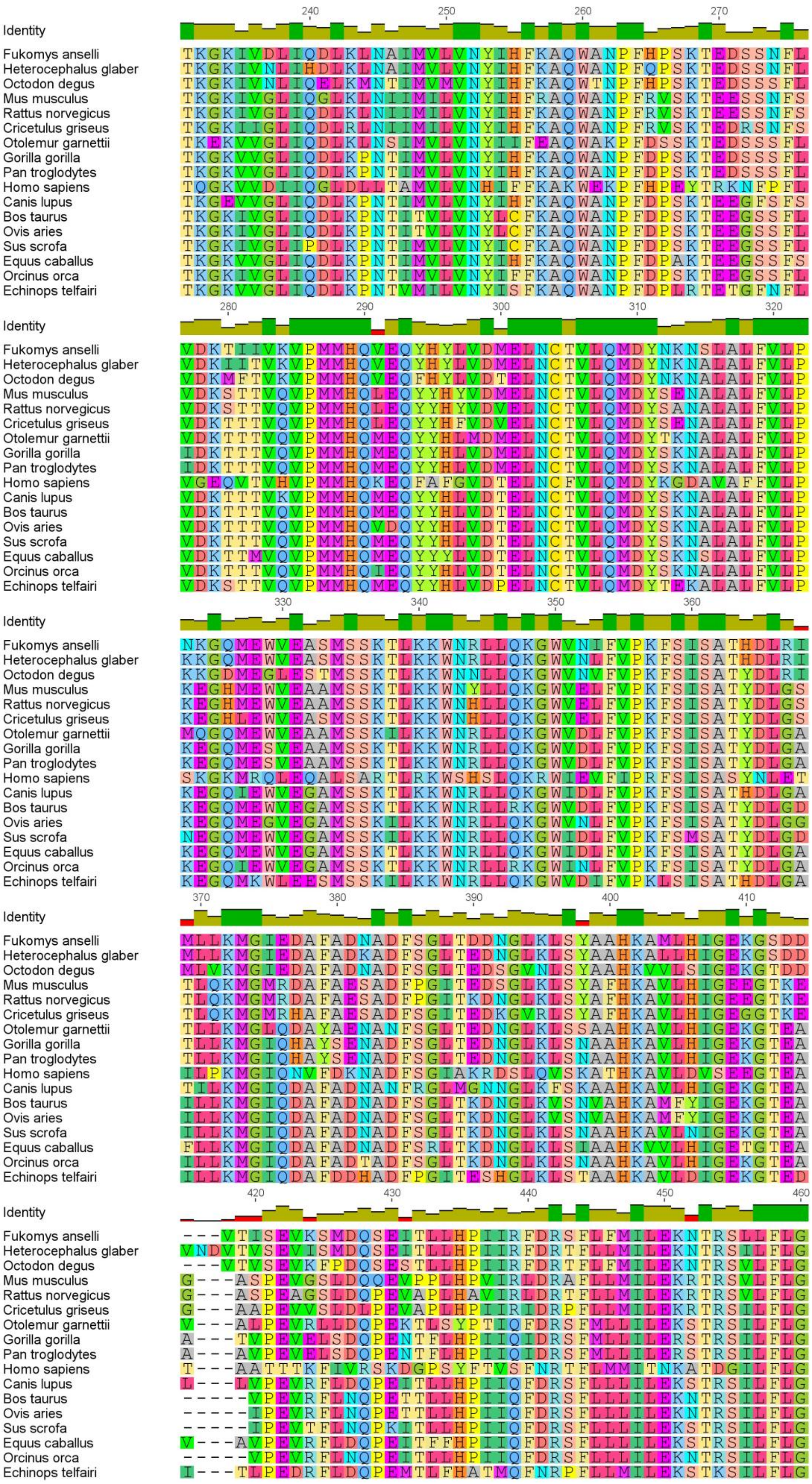

240 250 260 270
Identity
Fukomys anselli TKGKIVDLIQDLKLNAIMVLVNYIHFKAQWANPFHPSKTEDSSNFL
Heterocephalus glaber TKGKIVNLIHDLKLNAIMVLVNYIHFKAQWANPFQPSKTEDSSNFL
Octodon degus TKGKIVNLIQELKMNTIMVMVNYIHFKAQWTNPFHPSKTEDSSSFL
Mus musculus TGGKIVGLIQGLKLNIIMILVNYIHFRAQWANPFRVSKTEESSNFS
Rattus norvegicus TKGKIVGLIQDLKLNIIMILVNYIHFKAQWANPFRVSKTEESSNFS
Cricetulus griseus TKGKIIGLIQDLRLNIIMILVNYIHFKAQWANPFRVSKTEDRSNFS
Otolemur garnettii TKEKVVGLIQDLKLNSIMVLVNYIIFEAQWAKPFDSSKTEDSSSFL
Gorilla gorilla TKGKVVGLIQDLKPNTIMVLVNYIHFKAQWANPFDPSKTEDSSSFL
Pan troglodytes TKGKVVGLIQDLKPNTIMVLVNYIHFKAQWANPFDPSKTEDSSSFL
Homo sapiens TQGKVVDIIQGLDLLTAMVLVNHIFFKAKWEKPFHPEYTRKNFPFL
Canis lupus TKGEVVGLIQDLKPNTIMVLVNYIHFKAQWANPFDPSKTEEGFSFS
Bos taurus TKGKIVGLIQDLKPNTITVLVNYLCFKAQWANPFDPSKTEEGSSFL
Ovis aries TKGKIVGLIQDLKPNTITVLVNYLCFKAQWANPFDPSKTEEGSSFL
Sus scrofa TKGKIVGLIPDLKPNTIMVLVNYICFKAQWANPFDPSKTEEGSSFL
Equus caballus TKGKVVGLIQDLKPNTIMVLVNYIHFKAQWANPFDPAKTEEGSSFS
Orcinus orca TKGKIVGLIQDLKPNTIMVLVNYIFFKAQWANPFDPSKTEEGSSFL
Echinops telfairi TKGKVVGLIQDLKPNTVMILVNYISFKAQWANPFDPLRTETGFNFL
280 290 300 310 320
Identity
Fukomys anselli VDKTIIVKVPMMHQVEQYHYLVDMELNCTVLQMDYNKNSLALFVLP
Heterocephalus glaber VDKIITVKVPMMHQVEQYHYLVDMELNCTVLQMDYNKNALALFVLP
Octodon degus VDKMFTVKVPMMHQVEQFHYLVDTELNCTVLQMDYNKNALALFVLP
Mus musculus VDKSTTVQVPMMHQLEQYYHYVDMELNCTVLQMDYSENALALFVLP
Rattus norvegicus VDKSTTVQVPMMHQLEQYYHYVDVELNCTVLQMDYSANALALFVLP
Cricetulus griseus VDKTTTVQVPMMHQLEQYYHFVDVELNCTVLQMDYSENALALFVLP
Otolemur garnettii VDKTTTVQVPMMHQMEQYYHLMDMELNCTVLQMDYTKNALALFVLP
Gorilla gorilla IDKTTTVQVPMMHQMEQYYHLVDMELNCTVLQMDYSKNALALFVLP
Pan troglodytes IDKTTTVQVPMMHQMEQYYHLVDMELNCTVLQMDYSKNALALFVLP
Homo sapiens VGEQVTVHVPMMHQKEQFAFGVDTELNCFVLQMDYKGDAVAFFVLP
Canis lupus VDKTTTVKVPMMHQMEQYYHLVDTELNCTVLQMDYSKNALALFVLP
Bos taurus VDKTTTVQVPMMHQMEQYYHLVDTELNCTVLQMDYSKNALALFVLP
Ovis aries VDKTTTVQVPMMHQVDQYYHLVDTELNCTVLQMDYSKNALALFVLP
Sus scrofa VDKTTTVQVPMMHQMEQYYHLVDTELNCTVLQMDYSKNALALFVLP
Equus caballus VDKTTMVQVPMMHQMEQYYYLVDTELNCTVLQMDYSKNSLALFVLP
Orcinus orca VDKTTTVQVPMMHQIEQYYHLVDTELNCTVLQMDYSKNALALFVLP
Echinops telfairi VDKSTTVQVPMMHQMEQYYHLVDPELNCTVLQMDYTEKALALFVLP
330 340 350 360
Identity
Fukomys anselli NKGQMEWVEASMSSKTLKKWNRLLQKGWVNIFVPKFSISATHDLRI
Heterocephalus glaber KKGQMEWVEASMSSKTLKKWNRLLQKGWVNLFVPKFSISATHDLRI
Octodon degus KKGDMEGLESTMSSKTLKKWNNLLQKGWVNVFVPKFSISATYDLRI
Mus musculus KEGHMEWVEAAMSSKTLKKWNYLLQKGWVELFVPKFSISATYDLGS
Rattus norvegicus KEGHMEWVEAAMSSKTLKKWNHLLQKGWVELFVPKFSISATYDLGS
Cricetulus griseus KEGHLEWVEASMSSKTLKKWNHLLQKGWVELFVPKFSISATYDLGS
Otolemur garnettii MQGQMEWVEAAMSSKILKKWNRLLQKGWVDLFVPKFSISATYDLGA
Gorilla gorilla KEGQMESVEAAMSSKTLKKWNRLLQKGWVDLFVPKFSISATYDLGA
Pan troglodytes KEGQMESVEAAMSSKTLKKWNRLLQKGWVDLFVPKFSISATYDLGA
Homo sapiens SKGKMRQLEQALSARTLRKWSHSLQKRWIEVFIPRFSISASYNLET
Canis lupus KEGQIEWVEGAMSSKTLKKWNRLLQKGWIDLFVPKFSISATHDLGA
Bos taurus KEGQMEWVEGAMSSKTLKKWNRLLRKGWVDLFVPKFSISATYDLGD
Ovis aries KEGQMEGVEGAMSSKILKKWNRLLQKGWVNLFVPKFSISATYDLGG
Sus scrofa NEGQMEWVEGAMSSKILKKWNRLLQKGWIDLFVPKFSMSATYDLGD
Equus caballus KEGQMEWVEGAMSSKTLKKWNRLLQKGWIDLFVPKFSISATYDLGA
Orcinus orca KEGQIEWVEGAMSSKTLKKWNRLLRKGWINLFVPKFSISATYDLGA
Echinops telfairi KEGQMKWLEESMSSKILKKWNRLLQKGWVDIFVPKLSISATHDLGA
370 380 390 400 410
Identity
Fukomys anselli MLLKMGIEDAFADNADFSGLTDDNGLKLSYAAHKAMLHIGEKGSDD
Heterocephalus glaber MLLKMGIEDAFADKADFSGLTEDNGLKLSYAAHKALLHIGEKGSDD
Octodon degus MLVKMGIEDAFADNADFSGLTEDSGVNLSYAAHKVVLSIGEKGTDD
Mus musculus TLQKMGMRDAFAESADFPGITEDSGLKLSYAFHKAVLHIGEEGTKE
Rattus norvegicus TLQKMGMRDAFAESADFPGITKDNGLKLSYAFHKAVLHIGEEGTKE
Cricetulus griseus TLQKMGMRHAFAESADFSGITEDKGVRLSYAFHKAVLHIGEGGTKE
Otolemur garnettii TLLKMGLQDAYAENANFSGLTEDNGLKLSSAAHKAVLHIGEKGTEA
Gorilla gorilla TLLKMGIQHAYSENADFSGLTEDNGLKLSNAAHKAVLHIGEKGTEA
Pan troglodytes TLLKMGIQHAYSENADFSGLTEDNGLKLSNAAHKAVLHIGEKGTEA
Homo sapiens ILPKMGIQNVFDKNADFSGIAKRDSLQVSKATHKAVLDVSEEGTEA
Canis lupus TILKMGIQDAFADNANFRGLMGNNGLKFSKAAHKAVLHIGEKGTEA
Bos taurus ILLKMGIQDAFADNADFSGLTKDNGLKVSNVAHKAMFYIGEKGTEA
Ovis aries ILLKMGIQDAFADNADFSGLTKDNGLKVSNVAHKAMFYIGEKGTEA
Sus scrofa ILLKMGIQDAFADNADFSGLTKDNGLKLSNAAHKAVLNIGEKGTEA
Equus caballus FLLKMGIQDAFADNADFSRLTKDNGLKLSIAAHKVVLHIGETGTEA
Orcinus orca ILLKMGIQDAFADTADFSGLTKDNGLKLSNAAHKAVLHIGEKGTEA
Echinops telfairi ILLKMGIQDAFDDHADFPGITESHGLKLSTAAHKAVLDIGEKGTED
420 430 440 450 460
Identity
Fukomys anselli ---VTISEVKSMDQSEITLLHPIIRFDRSFLFMILEKNTRSLLFLG
Heterocephalus glaber VNDVTVSEVISMDQSEITLLHPIIRFDRTFLLMILEKNTRSILFLG
Octodon degus ---VTVSEVKFPDQSESTLLHPIIRFDRTFLFMILEKNTRSVLFLG
Mus musculus G---ASPEVGSLDQQEVPPLHPVIRLDRAFLLMILEKRTRSVLFLG
Rattus norvegicus G---ASPEAGSLDQPEVAPLHAVIRLDRTFLLMILEKRTRSVLFLG
Cricetulus griseus G---AAPEVVSLDLPEVAPLHPIIRIDRPFLLMILEKRTRSILFLG
Otolemur garnettii V---ALPEVRLLDQPEKTLSYPTIQFDRSFMLLILEKSTRSILFLG
Gorilla gorilla A---TVPEVELSDQPENTFLHPIIQIDRSFMLLILERSTRSILFLG
Pan troglodytes A---AVPEVELSDQPENTFLHPIIQIDRSFMLLILERSTRSILFLG
Homo sapiens T---AATTTKFIVRSKDGPSYFTVSFNRTFLMMITNKATDGILFLG
Canis lupus L---LVPEVRFLDQPEITLLHPIIQFDRSFLLLILEKSTRSILFLG
Bos taurus -----VPEVRFLNQPETTLLHPIIQFDRSFLLLILEKNTRSILFLG
Ovis aries -----IPEVRFLNQPETTLLHPIIQFDRSFLLLILEKNTRSILFLG
Sus scrofa -----IPEVTFLNQPKITLLHPIIQFDRSFLLLILEKSTRSILFLG
Equus caballus V---AVPEVRFLDQPEITFFHPIIQFDRSFLLLILEKSTRSILFLG
Orcinus orca -----VPEVRFLNQPEITLLHPIIQFDRSFLLLILEKNTRSILFLG
Echinops telfairi I---TLPEDRFLDQPEMTLFHATMQFNRPFLLMILEKSTKSILFLG

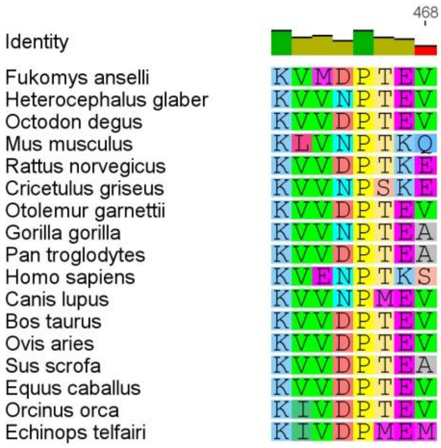

Figure S9. Protein alignment of thyroxine-binding globin (TBG) from different mammal species.
The mRNA sequence of *F. anselli* was obtained from RNA-seq and subsequently translated, other sequences were retrieved from NCBI databases with the following accession numbers: Heterocephalus glaber (EHB09876), Octodon degus (XP_004646260), Mus musculus (P61939), Rattus norvegicus (AAA42205), Cricetulus griseus (ERE65740), Otolemur garnettii (XP_003801681), Gorilla gorilla (XP_004064693), Pan troglodytes (NP_001009109), Homo sapiens (NP_783866), Canis lupus (XP_538128), Bos taurus (AAI03464), Ovis aries (NP_001094390), Sus scrofa (Q9TT35), Equus caballus (XP_001493492), Orcinus orca (XP_004285286), Echinops telfairi (XP_004710081).
