# Supplementary material for: Unusual Ratio between Free Thyroxine and Free Triiodothyronine in a Long-Lived Mole-Rat Species with Bimodal Ageing
Source: PLoS One. 2014 Nov 19;9(11):e113698. doi: 10.1371/journal.pone.0113698 (PMC4237498; doi:10.1371/journal.pone.0113698)
Supplement: Figure S10 — Protein alignment of hypoxia-induced factor (HIF1A) from different mammal species. The mRNA sequence of F. anselli was obtained from RNA-seq and subsequently translated, other sequences were retrieved from NCBI databases with the following accession numbers: Heterocephalus glaber (XP_004837489), Octodon degus (XP_004624861), Mus musculus (CAA70305), Rattus norvegicus (O35800), Ochotona princeps (XP_004597684), Otolemur garnettii (XP_003794480), Pan troglodytes (XP_001168972), Homo sapiens (NP_001521), Canis lupus (XP_003639249), Felis catus (XP_003987765), Bos taurus (NP_776764), Sus scrofa (NP_001116596), Orcinus orca (XP_004262152). (PDF) [file pone.0113698.s010.pdf]

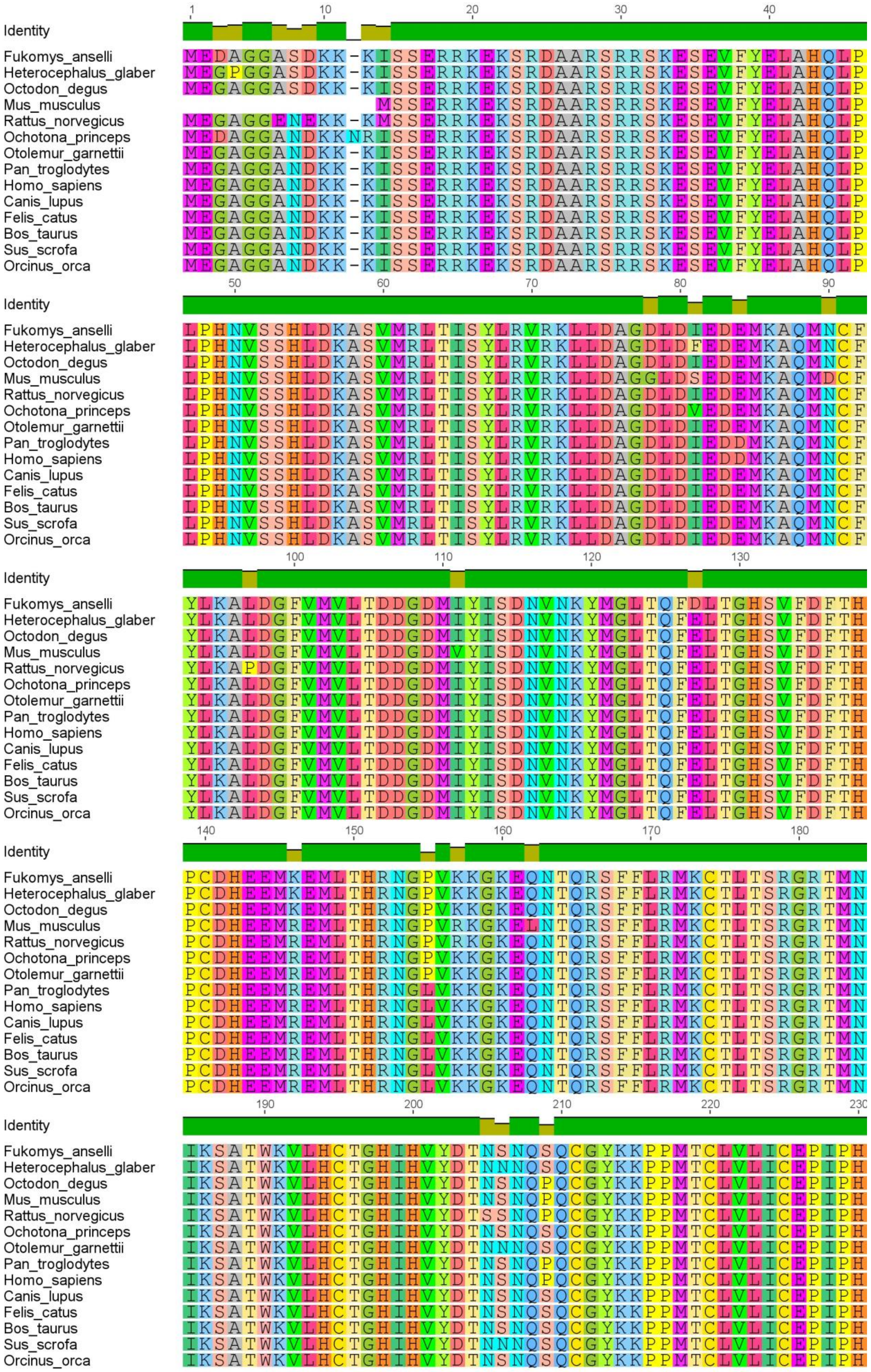
Identity
1 10 20 30 40
Fukomys_anselli MEDAGGASDKK-KISSERRKEKSRDAARSRRSKESEVFYELAHQLP
Heterocephalus_glaber MEGPGGASDKK-KISSERRKEKSRDAARSRRSKESEVFYELAHQLP
Octodon_degus MEGAGGASDKK-KISSERRKEKSRDAARSRRSKESEVFYELAHQLP
Mus_musculus MSSERRKEKSRDAARSRRSKESEVFYELAHQLP
Rattus_norvegicus MEGAGGENEKK-KMSSERRKEKSRDAARSRRSKESEVFYELAHQLP
Ochotona_princeps MEDAGGANDKKNRISSERRKEKSRDAARSRRSKESEVFYELAHQLP
Otolemur_garnettii MEGAGGANDKK-KISSERRKEKSRDAARSRRSKESEVFYELAHQLP
Pan_troglodytes MEGAGGANDKK-KISSERRKEKSRDAARSRRSKESEVFYELAHQLP
Homo_sapiens MEGAGGANDKK-KISSERRKEKSRDAARSRRSKESEVFYELAHQLP
Canis_lupus MEGAGGANDKK-KISSERRKEKSRDAARSRRSKESEVFYELAHQLP
Felis_catus MEGAGGANDKK-KISSERRKEKSRDAARSRRSKESEVFYELAHQLP
Bos_taurus MEGAGGANDKK-KISSERRKEKSRDAARSRRSKESEVFYELAHQLP
Sus_scrofa MEGAGGANDKK-KISSERRKEKSRDAARSRRSKESEVFYELAHQLP
Orcinus_orca MEGAGGANDKK-KISSERRKEKSRDAARSRRSKESEVFYELAHQLP
50 60 70 80 90
Identity
Fukomys_anselli LPHNVSSHLDKASVMRLTISYLRVRKLLDAGDLDIEDEMKAQMNCF
Heterocephalus_glaber LPHNVSSHLDKASVMRLTISYLRVRKLLDAGDLDFEDEMKAQMNCF
Octodon_degus LPHNVSSHLDKASVMRLTISYLRVRKLLDAGDLDIEDEMKAQMNCF
Mus_musculus LPHNVSSHLDKASVMRLTISYLRVRKLLDAGGLDSEDEMKAQMDCF
Rattus_norvegicus LPHNVSSHLDKASVMRLTISYLRVRKLLDAGDLDIEDEMKAQMNCF
Ochotona_princeps LPHNVSSHLDKASVMRLTISYLRVRKLLDAGDLDVEDEMKAQMNCF
Otolemur_garnettii LPHNVSSHLDKASVMRLTISYLRVRKLLDAGDLDIEDEMKAQMNCF
Pan_troglodytes LPHNVSSHLDKASVMRLTISYLRVRKLLDAGDLDIEDDMKAQMNCF
Homo_sapiens LPHNVSSHLDKASVMRLTISYLRVRKLLDAGDLDIEDDMKAQMNCF
Canis_lupus LPHNVSSHLDKASVMRLTISYLRVRKLLDAGDLDIEDEMKAQMNCF
Felis_catus LPHNVSSHLDKASVMRLTISYLRVRKLLDAGDLDIEDEMKAQMNCF
Bos_taurus LPHNVSSHLDKASVMRLTISYLRVRKLLDAGDLDIEDEMKAQMNCF
Sus_scrofa LPHNVSSHLDKASVMRLTISYLRVRKLLDAGDLDIEDEMKAQMNCF
Orcinus_orca LPHNVSSHLDKASVMRLTISYLRVRKLLDAGDLDIEDEMKAQMNCF
100 110 120 130
Identity
Fukomys_anselli YLKALDGFVMVLTDDGDMIYISDNVNKYMGLTQFDLTGHSVFDFTH
Heterocephalus_glaber YLKALDGFVMVLTDDGDMIYISDNVNKYMGLTQFELTGHSVFDFTH
Octodon_degus YLKALDGFVMVLTDDGDMIYISDNVNKYMGLTQFELTGHSVFDFTH
Mus_musculus YLKALDGFVMVLTDDGDMVYISDNVNKYMGLTQFELTGHSVFDFTH
Rattus_norvegicus YLKAPDGFVMVLTDDGDMIYISDNVNKYMGLTQFELTGHSVFDFTH
Ochotona_princeps YLKALDGFVMVLTDDGDMIYISDNVNKYMGLTQFELTGHSVFDFTH
Otolemur_garnettii YLKALDGFVMVLTDDGDMIYISDNVNKYMGLTQFELTGHSVFDFTH
Pan_troglodytes YLKALDGFVMVLTDDGDMIYISDNVNKYMGLTQFELTGHSVFDFTH
Homo_sapiens YLKALDGFVMVLTDDGDMIYISDNVNKYMGLTQFELTGHSVFDFTH
Canis_lupus YLKALDGFVMVLTDDGDMIYISDNVNKYMGLTQFELTGHSVFDFTH
Felis_catus YLKALDGFVMVLTDDGDMIYISDNVNKYMGLTQFELTGHSVFDFTH
Bos_taurus YLKALDGFVMVLTDDGDMIYISDNVNKYMGLTQFELTGHSVFDFTH
Sus_scrofa YLKALDGFVMVLTDDGDMIYISDNVNKYMGLTQFELTGHSVFDFTH
Orcinus_orca YLKALDGFVMVLTDDGDMIYISDNVNKYMGLTQFELTGHSVFDFTH
140 150 160 170 180
Identity
Fukomys_anselli PCDHEEMKEMLTHRNGPVKKGKEQNTQRSFFLRMKCTLTSRGRTMN
Heterocephalus_glaber PCDHEEMKEMLTHRNGPVKKGKEQNTQRSFFLRMKCTLTSRGRTMN
Octodon_degus PCDHEEMKEMLTHRNGPVKKGKEQNTQRSFFLRMKCTLTSRGRTMN
Mus_musculus PCDHEEMREMLTHRNGPVRKGKELNTQRSFFLRMKCTLTSRGRTMN
Rattus_norvegicus PCDHEEMREMLTHRNGPVRKGKEQNTQRSFFLRMKCTLTSRGRTMN
Ochotona_princeps PCDHEEMREMLTHRNGPVKKGKEQNTQRSFFLRMKCTLTSRGRTMN
Otolemur_garnettii PCDHEEMREMLTHRNGPVKKGKEQNTQRSFFLRMKCTLTSRGRTMN
Pan_troglodytes PCDHEEMREMLTHRNGLVKKGKEQNTQRSFFLRMKCTLTSRGRTMN
Homo_sapiens PCDHEEMREMLTHRNGLVKKGKEQNTQRSFFLRMKCTLTSRGRTMN
Canis_lupus PCDHEEMREMLTHRNGLVKKGKEQNTQRSFFLRMKCTLTSRGRTMN
Felis_catus PCDHEEMREMLTHRNGLVKKGKEQNTQRSFFLRMKCTLTSRGRTMN
Bos_taurus PCDHEEMREMLTHRNGLVKKGKEQNTQRSFFLRMKCTLTSRGRTMN
Sus_scrofa PCDHEEMREMLTHRNGLVKKGKEQNTQRSFFLRMKCTLTSRGRTMN
Orcinus_orca PCDHEEMREMLTHRNGLVKKGKEQNTQRSFFLRMKCTLTSRGRTMN
190 200 210 220 230
Identity
Fukomys_anselli IKSATWKVLHCTGHIHVYDTNSNQSQCGYKKPPMTCLVLICEPIPH
Heterocephalus_glaber IKSATWKVLHCTGHIHVYDTNNNQSQCGYKKPPMTCLVLICEPIPH
Octodon_degus IKSATWKVLHCTGHIHVYDTNSNQPQCGYKKPPMTCLVLICEPIPH
Mus_musculus IKSATWKVLHCTGHIHVYDTNSNQPQCGYKKPPMTCLVLICEPIPH
Rattus_norvegicus IKSATWKVLHCTGHIHVYDTSSNQPQCGYKKPPMTCLVLICEPIPH
Ochotona_princeps IKSATWKVLHCTGHIHVYDTNSNQSQCGYKKPPMTCLVLICEPIPH
Otolemur_garnettii IKSATWKVLHCTGHIHVYDTNNNQSQCGYKKPPMTCLVLICEPIPH
Pan_troglodytes IKSATWKVLHCTGHIHVYDTNSNQPQCGYKKPPMTCLVLICEPIPH
Homo_sapiens IKSATWKVLHCTGHIHVYDTNSNQPQCGYKKPPMTCLVLICEPIPH
Canis_lupus IKSATWKVLHCTGHIHVYDTNSNQSQCGYKKPPMTCLVLICEPIPH
Felis_catus IKSATWKVLHCTGHIHVYDTNSNQSQCGYKKPPMTCLVLICEPIPH
Bos_taurus IKSATWKVLHCTGHIHVYDTNSNQSQCGYKKPPMTCLVLICEPIPH
Sus_scrofa IKSATWKVLHCTGHIHVYDTNNNQSQCGYKKPPMTCLVLICEPIPH
Orcinus_orca IKSATWKVLHCTGHIHVYDTNSNQSQCGYKKPPMTCLVLICEPIPH

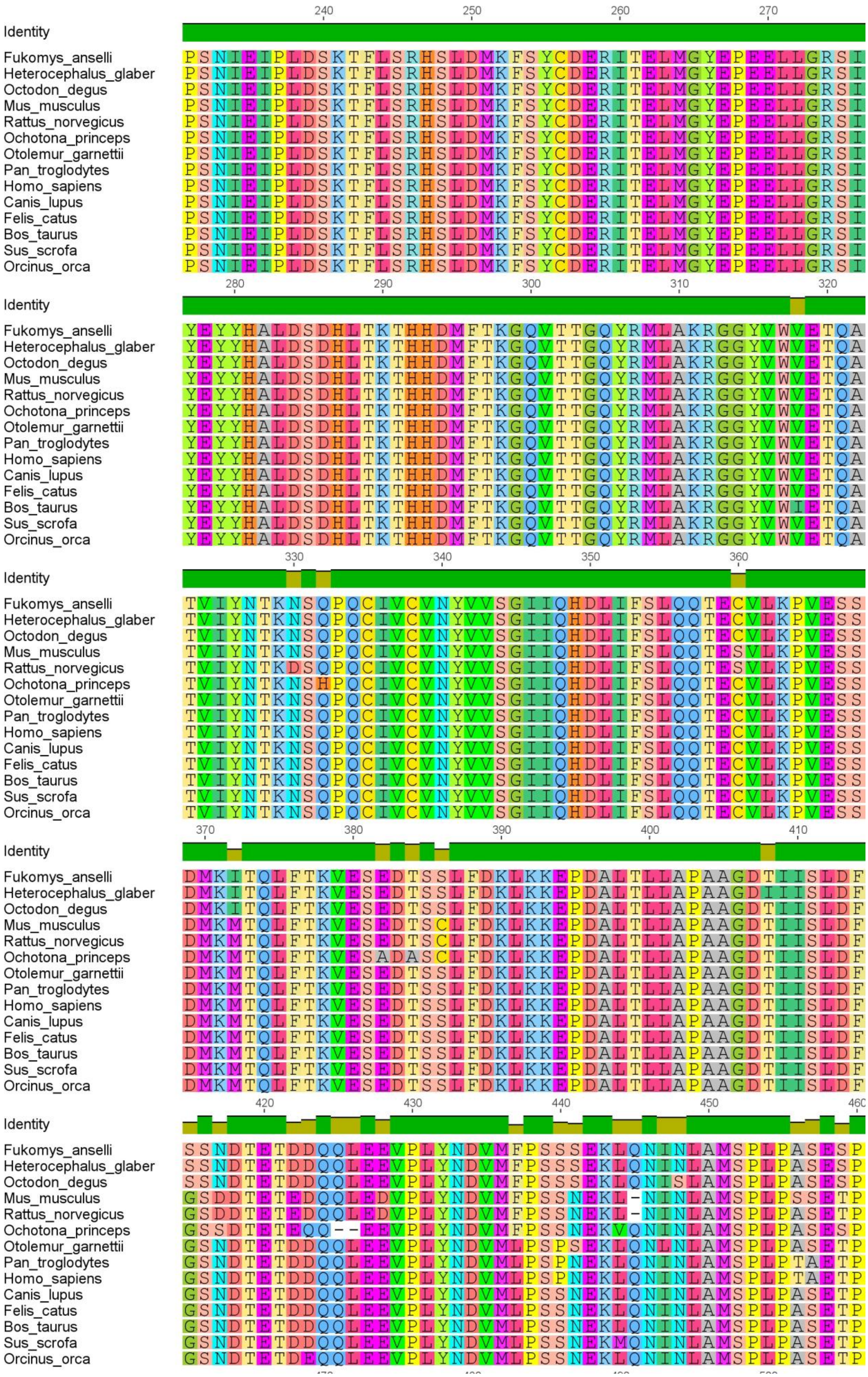

240 250 260 270
Identity
Fukomys_anselli PSNIEIPLDSKTFLSRHSLDMKFSYCDERITELMGYEPEELLGRSI
Heterocephalus_glaber PSNIEIPLDSKTFLSRHSLDMKFSYCDERITELMGYEPEELLGRSI
Octodon_degus PSNIEIPLDSKTFLSRHSLDMKFSYCDERITELMGYEPEELLGRSI
Mus_musculus PSNIEIPLDSKTFLSRHSLDMKFSYCDERITELMGYEPEELLGRSI
Rattus_norvegicus PSNIEIPLDSKTFLSRHSLDMKFSYCDERITELMGYEPEELLGRSI
Ochotona_princeps PSNIEIPLDSKTFLSRHSLDMKFSYCDERITELMGYEPEELLGRSI
Otolemur_garnettii PSNIEIPLDSKTFLSRHSLDMKFSYCDERITELMGYEPEELLGRSI
Pan_troglodytes PSNIEIPLDSKTFLSRHSLDMKFSYCDERITELMGYEPEELLGRSI
Homo_sapiens PSNIEIPLDSKTFLSRHSLDMKFSYCDERITELMGYEPEELLGRSI
Canis_lupus PSNIEIPLDSKTFLSRHSLDMKFSYCDERITELMGYEPEELLGRSI
Felis_catus PSNIEIPLDSKTFLSRHSLDMKFSYCDERITELMGYEPEELLGRSI
Bos_taurus PSNIEIPLDSKTFLSRHSLDMKFSYCDERITELMGYEPEELLGRSI
Sus_scrofa PSNIEIPLDSKTFLSRHSLDMKFSYCDERITELMGYEPEELLGRSI
Orcinus_orca PSNIEIPLDSKTFLSRHSLDMKFSYCDERITELMGYEPEELLGRSI
280 290 300 310 320
Identity
Fukomys_anselli YEYYHALDSDHLTKTHHDMFTKGQVTTGQYRMLAKRGGYVWVETQA
Heterocephalus_glaber YEYYHALDSDHLTKTHHDMFTKGQVTTGQYRMLAKRGGYVWVETQA
Octodon_degus YEYYHALDSDHLTKTHHDMFTKGQVTTGQYRMLAKRGGYVWVETQA
Mus_musculus YEYYHALDSDHLTKTHHDMFTKGQVTTGQYRMLAKRGGYVWVETQA
Rattus_norvegicus YEYYHALDSDHLTKTHHDMFTKGQVTTGQYRMLAKRGGYVWVETQA
Ochotona_princeps YEYYHALDSDHLTKTHHDMFTKGQVTTGQYRMLAKRGGYVWVETQA
Otolemur_garnettii YEYYHALDSDHLTKTHHDMFTKGQVTTGQYRMLAKRGGYVWVETQA
Pan_troglodytes YEYYHALDSDHLTKTHHDMFTKGQVTTGQYRMLAKRGGYVWVETQA
Homo_sapiens YEYYHALDSDHLTKTHHDMFTKGQVTTGQYRMLAKRGGYVWVETQA
Canis_lupus YEYYHALDSDHLTKTHHDMFTKGQVTTGQYRMLAKRGGYVWVETQA
Felis_catus YEYYHALDSDHLTKTHHDMFTKGQVTTGQYRMLAKRGGYVWVETQA
Bos_taurus YEYYHALDSDHLTKTHHDMFTKGQVTTGQYRMLAKRGGYVWIETQA
Sus_scrofa YEYYHALDSDHLTKTHHDMFTKGQVTTGQYRMLAKRGGYVWVETQA
Orcinus_orca YEYYHALDSDHLTKTHHDMFTKGQVTTGQYRMLAKRGGYVWVETQA
330 340 350 360
Identity
Fukomys_anselli TVIYNTKNSQPQCIVCVNYVVSGIIQHDLIFSLQQTECVLKPVESS
Heterocephalus_glaber TVIYNTKNSQPQCIVCVNYVVSGIIQHDLIFSLQQTECVLKPVESS
Octodon_degus TVIYNTKNSQPQCIVCVNYVVSGIIQHDLIFSLQQTECVLKPVESS
Mus_musculus TVIYNTKNSQPQCIVCVNYVVSGIIQHDLIFSLQQTESVLKPVESS
Rattus_norvegicus TVIYNTKDSQPQCIVCVNYVVSGIIQHDLIFSLQQTESVLKPVESS
Ochotona_princeps TVIYNTKNSHPQCIVCVNYVVSGIIQHDLIFSLQQTECVLKPVESS
Otolemur_garnettii TVIYNTKNSQPQCIVCVNYVVSGIIQHDLIFSLQQTECVLKPVESS
Pan_troglodytes TVIYNTKNSQPQCIVCVNYVVSGIIQHDLIFSLQQTECVLKPVESS
Homo_sapiens TVIYNTKNSQPQCIVCVNYVVSGIIQHDLIFSLQQTECVLKPVESS
Canis_lupus TVIYNTKNSQPQCIVCVNYVVSGIIQHDLIFSLQQTECVLKPVESS
Felis_catus TVIYNTKNSQPQCIVCVNYVVSGIIQHDLIFSLQQTECVLKPVESS
Bos_taurus TVIYNTKNSQPQCIVCVNYVVSGIIQHDLIFSLQQTECVLKPVESS
Sus_scrofa TVIYNTKNSQPQCIVCVNYVVSGIIQHDLIFSLQQTECVLKPVESS
Orcinus_orca TVIYNTKNSQPQCIVCVNYVVSGIIQHDLIFSLQQTECVLKPVESS
370 380 390 400 410
Identity
Fukomys_anselli DMKITQLFTKVESEDTSSLFDKLKKEPDALTLLAPAAGDTIISLDF
Heterocephalus_glaber DMKITQLFTKVESEDTSSLFDKLKKEPDALTLLAPAAGDIIISLDF
Octodon_degus DMKITQLFTKVESEDTSSLFDKLKKEPDALTLLAPAAGDTIISLDF
Mus_musculus DMKMTQLFTKVESEDTSCLFDKLKKEPDALTLLAPAAGDTIISLDF
Rattus_norvegicus DMKMTQLFTKVESEDTSCLFDKLKKEPDALTLLAPAAGDTIISLDF
Ochotona_princeps DMKMTQLFTKVESADASCLFDKLKKEPDALTLLAPAAGDTIISLDF
Otolemur_garnettii DMKMTQLFTKVESEDTSSLFDKLKKEPDALTLLAPAAGDTIISLDF
Pan_troglodytes DMKMTQLFTKVESEDTSSLFDKLKKEPDALTLLAPAAGDTIISLDF
Homo_sapiens DMKMTQLFTKVESEDTSSLFDKLKKEPDALTLLAPAAGDTIISLDF
Canis_lupus DMKMTQLFTKVESEDTSSLFDKLKKEPDALTLLAPAAGDTIISLDF
Felis_catus DMKMTQLFTKVESEDTSSLFDKLKKEPDALTLLAPAAGDTIISLDF
Bos_taurus DMKMTQLFTKVESEDTSSLFDKLKKEPDALTLLAPAAGDTIISLDF
Sus_scrofa DMKMTQLFTKVESEDTSSLFDKLKKEPDALTLLAPAAGDTIISLDF
Orcinus_orca DMKMTQLFTKVESEDTSSLFDKLKKEPDALTLLAPAAGDTIISLDF
420 430 440 450 460
Identity
Fukomys_anselli SSNDTETDDQQLEEVPLYNDVMFPSSSEKLQNINLAMSPLPASESP
Heterocephalus_glaber SSNDTETDDQQLEEVPLYNDVMFPSSSEKLQNINLAMSPLPASESP
Octodon_degus SSNDTETDDQQLEEVPLYNDVMFPSSSEKLQNISLAMSPLPASESP
Mus_musculus GSDDTETEDQQLEDVPLYNDVMFPSSNEKL-NINLAMSPLPSSETP
Rattus_norvegicus GSDDTETEDQQLEDVPLYNDVMFPSSNEKL-NINLAMSPLPASETP
Ochotona_princeps GSSDTETEQQ--EEVPLYNDVMFPSSNEKVQNINLAMSPLPASESP
Otolemur_garnettii GSNDTETDDQQLEEVPLYNDVMLPSPSEKLQNLNLAMSPLPASETP
Pan_troglodytes GSNDTETDDQQLEEVPLYNDVMLPSPNEKLQNINLAMSPLPTAETP
Homo_sapiens GSNDTETDDQQLEEVPLYNDVMLPSPNEKLQNINLAMSPLPTAETP
Canis_lupus GSNDTETDDQQLEEVPLYNDVMLPSSNEKLQNINLAMSPLPASETP
Felis_catus GSNDTETDDQQLEEVPLYNDVMLPSSNEKLQNINLAMSPLPASETP
Bos_taurus GSNDTETDDQQLEEVPLYNDVMLPSSNEKLQNINLAMSPLPASETP
Sus_scrofa GSNDTETDDQQLEEVPLYNDVMLPSSNEKMQNINLAMSPLPASETP
Orcinus_orca GSNDTETDEQQLEEVPLYNDVMLPSSNEKLQNINLAMSPLPASETP

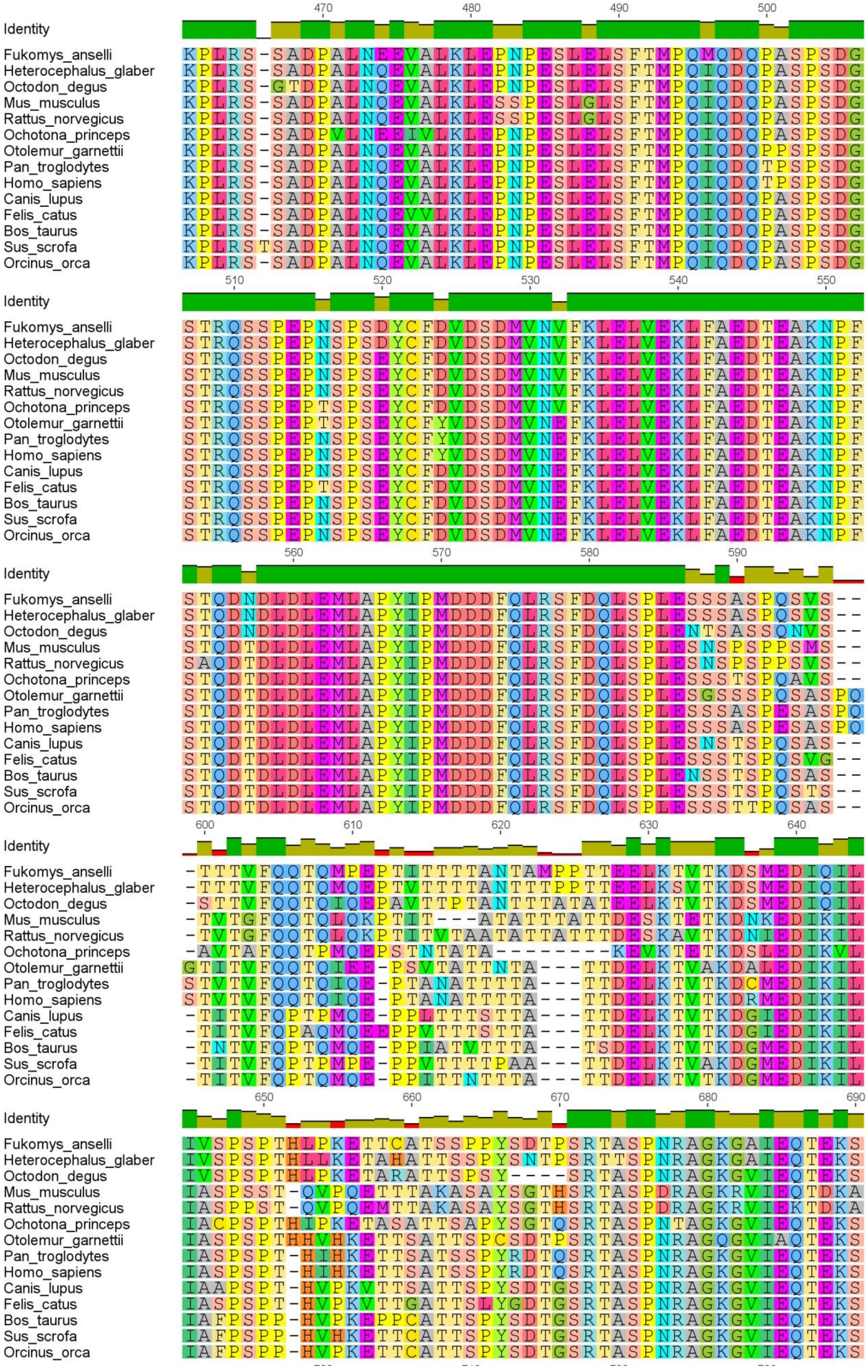

470 480 490 500
Identity
Fukomys_anselli KPLRS-SADPALNEEVALKLEPNPESLELSFTMPQMQDQPASPSDG
Heterocephalus_glaber KPLRS-SADPALNQEVALKLEPNPESLELSFTMPQIQDQPASPSDG
Octodon_degus KPLRS-GTDPALNQEVALKLEPNPESLELSFTMPQIQDQPASPSDG
Mus_musculus KPLRS-SADPALNQEVALKLESSPESLGLSFTMPQIQDQPASPSDG
Rattus_norvegicus KPLRS-SADPALNQEVALKLESSPESLGLSFTMPQIQDQPASPSDG
Ochotona_princeps KPLRS-SADPVLNEEIVLKLEPNPESLELSFTMPQIQDQPASPSDG
Otolemur_garnettii KPLRS-SADPALNQEVALKLEPNPESLELSFTMPQIQDQPPSPSDG
Pan_troglodytes KPLRS-SADPALNQEVALKLEPNPESLELSFTMPQIQDQTPSPSDG
Homo_sapiens KPLRS-SADPALNQEVALKLEPNPESLELSFTMPQIQDQTPSPSDG
Canis_lupus KPLRS-SADPALNQEVALKLEPNPESLELSFTMPQIQDQPASPSDG
Felis_catus KPLRS-SADPALNQEVVLKLEPNPESLELSFTMPQIQDQPASPSDG
Bos_taurus KPLRS-SADPALNQEVALKLEPNPESLELSFTMPQIQDQPASPSDG
Sus_scrofa KPLRSTSADPALNQEVALKLEPNPESLELSFTMPQIQDQPASPSDG
Orcinus_orca KPLRS-SADPALNQEVALKLEPNPESLELSFTMPQIQDQPASPSDG
510 520 530 540 550
Identity
Fukomys_anselli STRQSSPEPNSPSDYCFDVDSDMVNVFKLELVEKLFAEDTEAKNPF
Heterocephalus_glaber STRQSSPEPNSPSDYCFDVDSDMVNVFKLELVEKLFAEDTEAKNPF
Octodon_degus STRQSSPEPNSPSEYCFDVDSDMVNVFKLELVEKLFAEDTEAKNPF
Mus_musculus STRQSSPEPNSPSEYCFDVDSDMVNVFKLELVEKLFAEDTEAKNPF
Rattus_norvegicus STRQSSPEPNSPSEYCFDVDSDMVNVFKLELVEKLFAEDTEAKNPF
Ochotona_princeps STRQSSPEPTSPSEYCFDVDSDMVNVFKLELVEKLFAEDTEAKNPF
Otolemur_garnettii STRQSSPEPTSPSEYCFYVDSDMVNEFKLELVEKLFAEDTEAKNPF
Pan_troglodytes STRQSSPEPNSPSEYCFYVDSDMVNEFKLELVEKLFAEDTEAKNPF
Homo_sapiens STRQSSPEPNSPSEYCFYVDSDMVNEFKLELVEKLFAEDTEAKNPF
Canis_lupus STRQSSPEPNSPSEYCFDVDSDMVNEFKLELVEKLFAEDTEAKNPF
Felis_catus STRQSSPEPTSPSEYCFDVDSDMVNEFKLELVEKLFAEDTEAKNPF
Bos_taurus STRQSSPEPNSPSEYCFDVDSDMVNEFKLELVEKLFAEDTEAKNPF
Sus_scrofa STRQSSPEPNSPSEYCFDVDSDMVNEFKLELVEKLFAEDTEAKNPF
Orcinus_orca STRQSSPEPNSPSEYCFDVDSDMVNEFKLELVEKLFAEDTEAKNPF
560 570 580 590
Identity
Fukomys_anselli STQDNDLDLEMLAPYIPMDDDFQLRSFDQLSPLESSSASPQSVS--
Heterocephalus_glaber STQDNDLDLEMLAPYIPMDDDFQLRSFDQLSPLESSSASPQSVS--
Octodon_degus STQDNDLDLEMLAPYIPMDDDFQLRSFDQLSPLENTSASSQNVS--
Mus_musculus STQDTDLDLEMLAPYIPMDDDFQLRSFDQLSPLESNSPSPPSMS--
Rattus_norvegicus SAQDTDLDLEMLAPYIPMDDDFQLRSFDQLSPLESNSPSPPSVS--
Ochotona_princeps STQDTDLDLEMLAPYIPMDDDFQLRSFDQLSPLESSSTSPQAVS--
Otolemur_garnettii STQDTDLDLEMLAPYIPMDDDFQLRSFDQLSPLESGSSSPQSASPQ
Pan_troglodytes STQDTDLDLEMLAPYIPMDDDFQLRSFDQLSPLESSSASPESASPQ
Homo_sapiens STQDTDLDLEMLAPYIPMDDDFQLRSFDQLSPLESSSASPESASPQ
Canis_lupus STQDTDLDLEMLAPYIPMDDDFQLRSFDQLSPLESNSTSPQSAS--
Felis_catus STQDTDLDLEMLAPYIPMDDDFQLRSFDQLSPLESSSTSPQSVG--
Bos_taurus STQDTDLDLEMLAPYIPMDDDFQLRSFDQLSPLENSSTSPQSAS--
Sus_scrofa STQDTDLDLEMLAPYIPMDDDFQLRSFDQLSPLESSSTSPQSTS--
Orcinus_orca STQDTDLDLEMLAPYIPMDDDFQLRSFDQLSPLESSSTTPQSAS--
600 610 620 630 640
Identity
Fukomys_anselli -TTTVFQQTQMPEPTITTTTANTAMPPTTEELKTVTKDSMEDIQIL
Heterocephalus_glaber -TTTVFQQTQMQEPTVTTTTANTTTPPTTEELKSVTKDSMEDIQIL
Octodon_degus -STTVFQQTQIQEPAVTTPTANTTTATATEELKTVTKDSMEDIQIL
Mus_musculus -TVTGFQQTQLQKPTIT---ATATTTATTDESKTETKDNKEDIKIL
Rattus_norvegicus -TVTGFQQTQLQKPTITVTAATATTATTTDESKAVTKDNIEDIKIL
Ochotona_princeps -AVTAFQQTPMQEPSTNTATA--------KEVKTETKDSLEDIKVL
Otolemur_garnettii GTITVFQQTQIEE-PSVTATTNTA---TTDELKTVAKDALEDIKIL
Pan_troglodytes STVTVFQQTQIQE-PTANATTTTA---TTDELKTVTKDCMEDIKIL
Homo_sapiens STVTVFQQTQIQE-PTANATTTTA---TTDELKTVTKDRMEDIKIL
Canis_lupus -TITVFQPTPMQE-PPLTTTSTTA---TTDELKTVTKDGIEDIKIL
Felis_catus -TITVFQPAQMQEEPPVTTTSTTA---TTDELKTVTKDGIEDIKIL
Bos_taurus -TNTVFQPTQMQE-PPIATVTTTA---TSDELKTVTKDGMEDIKIL
Sus_scrofa -TITVFQPTPMPE-PPVTTTTPAA---TTDELKTVAKDGMEDIKIL
Orcinus_orca -TITVFQPTQMQE-PPITTNTTTA---TTDELKTVTKDGMEDIKIL
650 660 670 680 690
Identity
Fukomys_anselli IVSPSPTHLPKETTCATSSPPYSDTPSRTASPNRAGKGAIEQTEKS
Heterocephalus_glaber IVSPSPTHLLKETAHATTSSPYSNTPSRTTSPNRAGKGAIEQTEKS
Octodon_degus IVSPSPTHLPKETARATTSPSY----SRTASPNRAGKGVIEQTEKS
Mus_musculus IASPSST-QVPQETTTAKASAYSGTHSRTASPDRAGKRVIEQTDKA
Rattus_norvegicus IASPPST-QVPQEMTTAKASAYSGTHSRTASPDRAGKRVIEKTDKA
Ochotona_princeps IACPSPTHIPKETASATTSAPYSGTQSRTASPNTAGKGVIEQTEKS
Otolemur_garnettii IASPSPTHHVHKETTSATTSPCSDTPSRTASPNRAGQGVIAQTEKS
Pan_troglodytes IASPSPT-HIHKETTSATSSPYRDTQSRTASPNRAGKGVIEQTEKS
Homo_sapiens IASPSPT-HIHKETTSATSSPYRDTQSRTASPNRAGKGVIEQTEKS
Canis_lupus IAAPSPT-HVPKVTTSATTSPYSDTGSRTASPNRAGKGVIEQTEKS
Felis_catus IASPSPT-HVPKVTTGATTSLYGDTGSRTASPNRAGKGVIEQTEKS
Bos_taurus IAFPSPP-HVPKEPPCATTSPYSDTGSRTASPNRAGKGVIEQTEKS
Sus_scrofa IAFPSPP-HVHKETTCATTSPYSDTGSRTASPNRAGKGVIEQTEKS
Orcinus_orca IAFPSPP-HVPKETTCATTSPYSDTGSRTASPNRAGKGVIEQTEKS

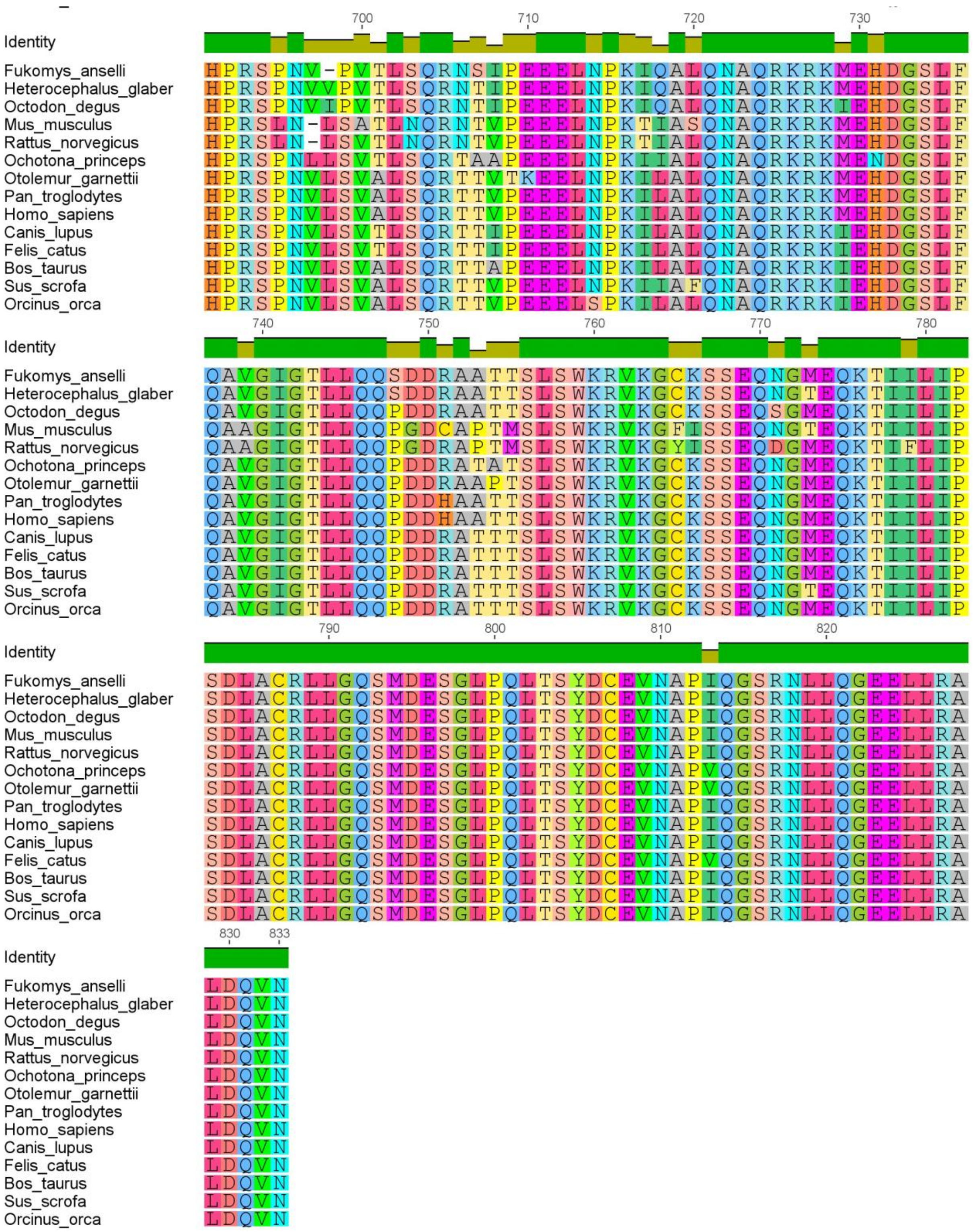

Figure S10. Protein alignment of hypoxia-induced factor (HIF1A) from different mammal species. The mRNA sequence of *F. anselli* was obtained from RNA-seq and subsequently translated, other sequences were retrieved from NCBI databases with the following accession numbers: Heterocephalus glaber (XP_004837489), Octodon degus (XP_004624861), Mus musculus (CAA70305), Rattus norvegicus (O35800), Ochotona princeps (XP_004597684), Otolemur garnettii (XP_003794480), Pan troglodytes (XP_001168972), Homo sapiens (NP_001521), Canis lupus (XP_003639249), Felis catus (XP_003987765), Bos taurus (NP_776764), Sus scrofa (NP_001116596), Orcinus orca (XP_004262152).
