# Supplementary material for: Unusual Ratio between Free Thyroxine and Free Triiodothyronine in a Long-Lived Mole-Rat Species with Bimodal Ageing
Source: PLoS One. 2014 Nov 19;9(11):e113698. doi: 10.1371/journal.pone.0113698 (PMC4237498; doi:10.1371/journal.pone.0113698)
Supplement: Figure S11 — Protein alignment of Monocarboxylate transporter 8 (MCT8) from different mammal species. The mRNA sequence of F. anselli was obtained from RNA-seq and subsequently translated, other sequences were retrieved from NCBI databases with the following accession numbers: Heterocephalus glaber (XP_004905154), Cavia porcellus (XP_005004229), Octodon degus (XP_004648070), Mus musculus (AAC40078), Rattus norvegicus (EDM07172), Ochotona princeps (XP_004592817), Otolemur garnettii (XP_003802275), Macaca mulatta (XP_001096017), Homo sapiens (NP_006508), Bos taurus (NP_001193868), Orcinus orca (XP_004283857). Suggestions of NCBI for translation starts of Octodon degus (XP_004648070), Ochotona princeps (XP_004592817), Macaca mulatta (XP_001096017) and Bos taurus (NP_001193868) were changed to the position of the orthologous sequences. (PDF) [file pone.0113698.s011.pdf]

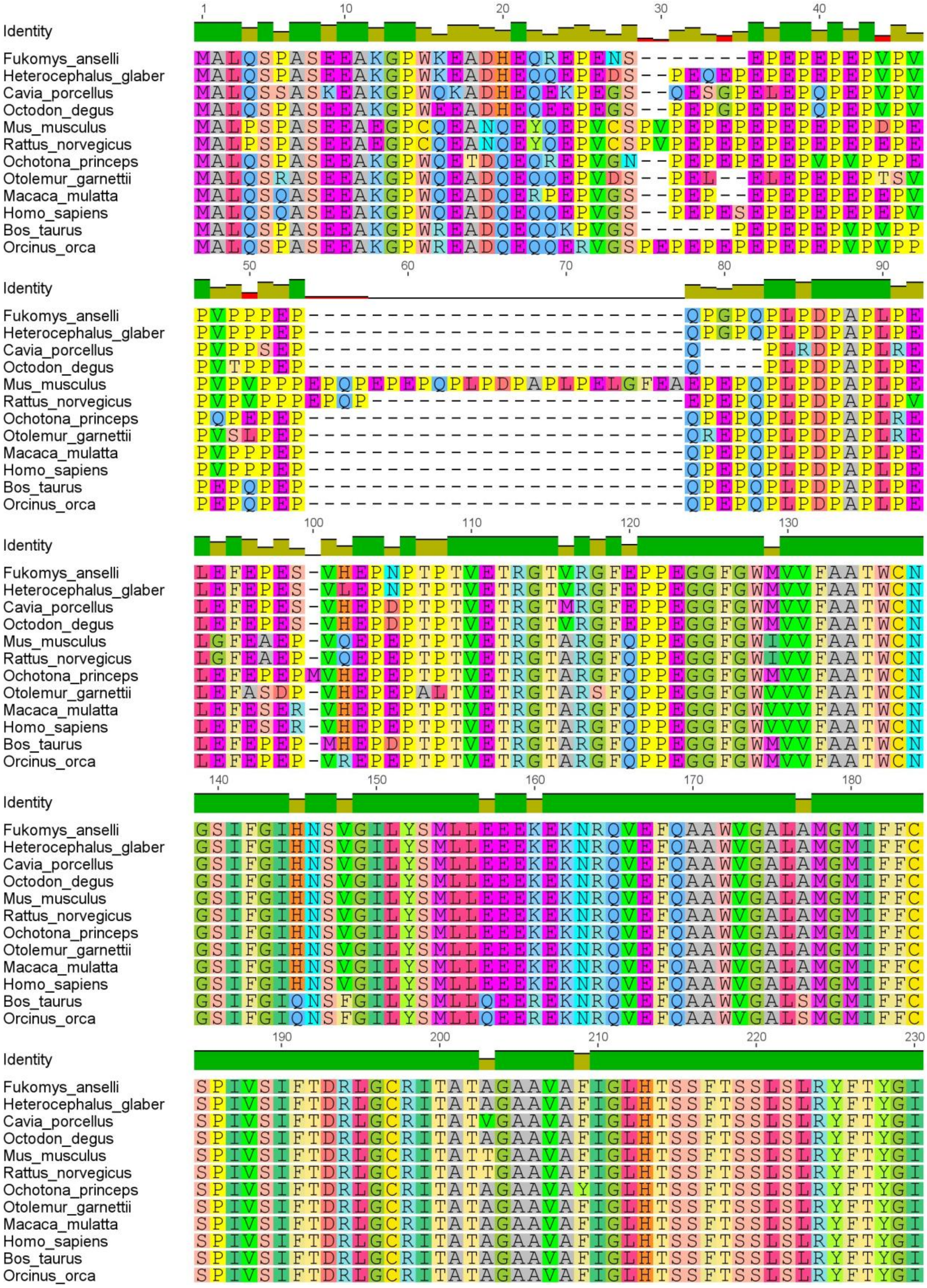
1 10 20 30 40
Identity
Fukomys_anselli MALQSPASEEAKGPWKEADHEQREPENS-------EPEPEPEPVPV
Heterocephalus_glaber MALQSPASEEAKGPWKEADHEQQEPEDS--PEQEPEPEPEPEPVPV
Cavia_porcellus MALQSSASKEAKGPWQKADHEQEKPEGS--QESGPELEPQPEPVPV
Octodon_degus MALQSPASEEAKGPWEEADHEQEEPEGS--PEPGPEPEPQPEPVPV
Mus_musculus MALPSPASEEAEGPCQEANQEYQEPVCSPVPEPEPEPEPEPEPDPE
Rattus_norvegicus MALPSPASEEAEGPCQEANQEYQEPVCSPVPEPEPEPEPEPEPEPE
Ochotona_princeps MALQSPASEEAKGPWQETDQEQREPVGN--PEPEPEPEPVPVPPPE
Otolemur_garnettii MALQSRASEEAKGPWQEADQEQQEPVDS--PEL--ELEPEPEPTSV
Macaca_mulatta MALQSQASEEAKGPWQEADQERPEPVGS--PEP--EPEPEPEPEPV
Homo_sapiens MALQSQASEEAKGPWQEADQEQQEPVGS--PEPESEPEPEPEPEPV
Bos_taurus MALQSPASEEAKGPWREADQEQQKPVGS------PEPEPEPVPVPP
Orcinus_orca MALQSPASEEAKGPWREADQEQQERVGSPEPEPEPEPEPEPVPVPP
50 60 70 80 90
Identity
Fukomys_anselli PVPPPEP------------------------QPGPQPLPDPAPLPE
Heterocephalus_glaber PVPPPEP------------------------QPGPQPLPDPAPLPE
Cavia_porcellus PVPPSEP------------------------Q----PLRDPAPLRE
Octodon_degus PVTPPEP------------------------Q----PLPDPAPLPE
Mus_musculus PVPVPPPEPQPEPEPQPLPDPAPLPELGFEAEPEPQPLPDPAPLPE
Rattus_norvegicus PVPVPPPEPQP--------------------EPEPQPLPDPAPLPV
Ochotona_princeps PQPEPEP------------------------QPEPQPLPDPAPLRE
Otolemur_garnettii PVSLPEP------------------------QREPQPLPDPAPLRE
Macaca_mulatta PVPPPEP------------------------QPEPQPLPDPAPLPE
Homo_sapiens PVPPPEP------------------------QPEPQPLPDPAPLPE
Bos_taurus PEPQPEP------------------------QPEPQPLPDPAPLPE
Orcinus_orca PEPQPEP------------------------QPEPQPLPDPAPLPE
100 110 120 130
Identity
Fukomys_anselli LEFEPES-VHEPNPTPTVETRGTVRGFEPPEGGFGWMVVFAATWCN
Heterocephalus_glaber LEFEPES-VLEPNPTPTVETRGTVRGFEPPEGGFGWMVVFAATWCN
Cavia_porcellus LEFEPES-VHEPDPTPTVETRGTMRGFEPPEGGFGWMVVFAATWCN
Octodon_degus LEFEPES-VHEPDPTPTVETRGTVRGFEPPEGGFGWMVVFAATWCN
Mus_musculus LGFEAEP-VQEPEPTPTVETRGTARGFQPPEGGFGWIVVFAATWCN
Rattus_norvegicus LGFEAEP-VQEPEPTPTVETRGTARGFQPPEGGFGWIVVFAATWCN
Ochotona_princeps LEFEPEPMVHEPEPTPTVETRGTARGFQPPEGGFGWMVVFAATWCN
Otolemur_garnettii LEFASDP-VHEPEPALTVETRGTARSFQPPEGGFGWVVVFAATWCN
Macaca_mulatta LEFESER-VHEPEPTPTVETRGTARGFQPPEGGFGWVVVFAATWCN
Homo_sapiens LEFESER-VHEPEPTPTVETRGTARGFQPPEGGFGWVVVFAATWCN
Bos_taurus LEFEPEP-MHEPDPTPTVETRGTARGFQPPEGGFGWMVVFAATWCN
Orcinus_orca LEFEPEP-VREPEPTPTVETRGTARGFQPPEGGFGWMVVFAATWCN
140 150 160 170 180
Identity
Fukomys_anselli GSIFGIHNSVGILYSMLLEEEKEKNRQVEFQAAWVGALAMGMIFFC
Heterocephalus_glaber GSIFGIHNSVGILYSMLLEEEKEKNRQVEFQAAWVGALAMGMIFFC
Cavia_porcellus GSIFGIHNSVGILYSMLLEEEKEKNRQVEFQAAWVGALAMGMIFFC
Octodon_degus GSIFGIHNSVGILYSMLLEEEKEKNRQVEFQAAWVGALAMGMIFFC
Mus_musculus GSIFGIHNSVGILYSMLLEEEKEKNRQVEFQAAWVGALAMGMIFFC
Rattus_norvegicus GSIFGIHNSVGILYSMLLEEEKEKNRQVEFQAAWVGALAMGMIFFC
Ochotona_princeps GSIFGIHNSVGILYSMLLEEEKEKNRQVEFQAAWVGALAMGMIFFC
Otolemur_garnettii GSIFGIHNSVGILYSMLLEEEKEKNRQVEFQAAWVGALAMGMIFFC
Macaca_mulatta GSIFGIHNSVGILYSMLLEEEKEKNRQVEFQAAWVGALAMGMIFFC
Homo_sapiens GSIFGIHNSVGILYSMLLEEEKEKNRQVEFQAAWVGALAMGMIFFC
Bos_taurus GSIFGIQNSFGILYSMLLQEEREKNRQVEFQAAWVGALSMGMIFFC
Orcinus_orca GSIFGIQNSFGILYSMLLQEEREKNRQVEFQAAWVGALSMGMIFFC
190 200 210 220 230
Identity
Fukomys_anselli SPIVSIFTDRLGCRITATAGAAVAFIGLHTSSFTSSLSLRYFTYGI
Heterocephalus_glaber SPIVSIFTDRLGCRITATAGAAVAFIGLHTSSFTSSLSLRYFTYGI
Cavia_porcellus SPIVSIFTDRLGCRITATVGAAVAFIGLHTSSFTSSLSLRYFTYGI
Octodon_degus SPIVSIFTDRLGCRITATAGAAVAFIGLHTSSFTSSLSLRYFTYGI
Mus_musculus SPIVSIFTDRLGCRITATTGAAVAFIGLHTSSFTSSLSLRYFTYGI
Rattus_norvegicus SPIVSIFTDRLGCRITATTGAAVAFIGLHTSSFTSSLSLRYFTYGI
Ochotona_princeps SPIVSIFTDRLGCRITATAGAAVAYIGLHTSSFTSSLSLRYFTYGI
Otolemur_garnettii SPIVSIFTDRLGCRITATAGAAVAFIGLHTSSFTSSLSLRYFTYGI
Macaca_mulatta SPIVSIFTDRLGCRITATAGAAVAFIGLHTSSFTSSLSLRYFTYGI
Homo_sapiens SPIVSIFTDRLGCRITATAGAAVAFIGLHTSSFTSSLSLRYFTYGI
Bos_taurus SPIVSIFTDRLGCRITATAGAAVAFIGLHTSSFTSSLSLRYFTYGI
Orcinus_orca SPIVSIFTDRLGCRITATAGAAVAFIGLHTSSFTSSLSLRYFTYGI

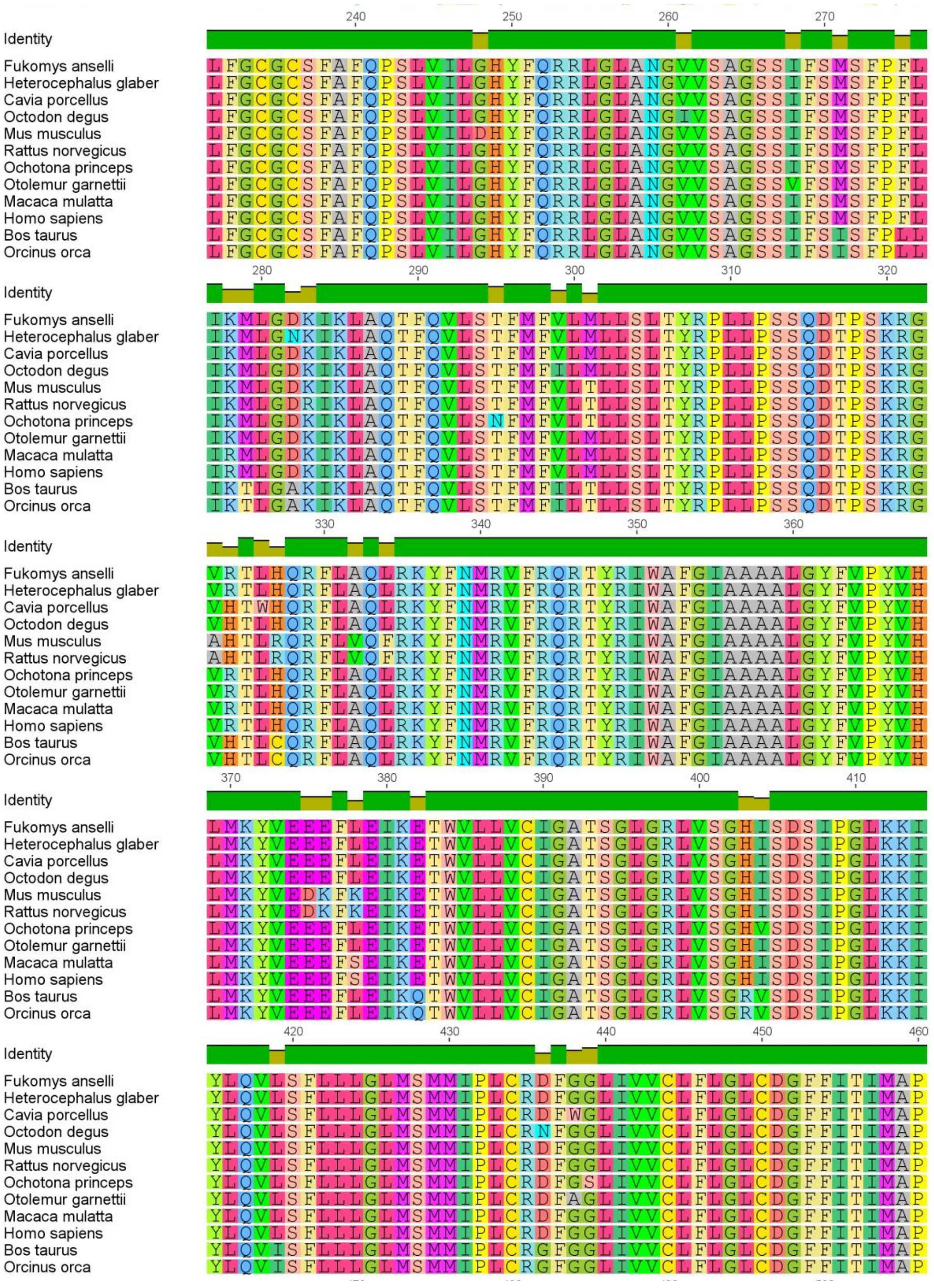

240 250 260 270
Identity
Fukomys anselli LFGCGCSFAFQPSLVILGHYFQRRLGLANGVVSAGSSIFSMSFPFL
Heterocephalus glaber LFGCGCSFAFQPSLVILGHYFQRRLGLANGVVSAGSSIFSMSFPFL
Cavia porcellus LFGCGCSFAFQPSLVILGHYFQRRLGLANGVVSAGSSIFSMSFPFL
Octodon degus LFGCGCSFAFQPSLVILGHYFQRRLGLANGIVSAGSSIFSMSFPFL
Mus musculus LFGCGCSFAFQPSLVILDHYFQRRLGLANGVVSAGSSIFSMSFPFL
Rattus norvegicus LFGCGCSFAFQPSLVILGHYFQRRLGLANGVVSAGSSIFSMSFPFL
Ochotona princeps LFGCGCSFAFQPSLVILGHYFQRRLGLANGVVSAGSSIFSMSFPFL
Otolemur garnettii LFGCGCSFAFQPSLVILGHYFQRRLGLANGVVSAGSSVFSMSFPFL
Macaca mulatta LFGCGCSFAFQPSLVILGHYFQRRLGLANGVVSAGSSIFSMSFPFL
Homo sapiens LFGCGCSFAFQPSLVILGHYFQRRLGLANGVVSAGSSIFSMSFPFL
Bos taurus LFGCGCSFAFQPSLVILGHYFQRRLGLANGVVSAGSSIFSISFPLL
Orcinus orca LFGCGCSFAFQPSLVILGHYFQRRLGLANGVVSAGSSIFSISFPLL
280 290 300 310 320
Identity
Fukomys anselli IKMLGDKIKLAQTFQVLSTFMFVLMLLSLTYRPLLPSSQDTPSKRG
Heterocephalus glaber IKMLGNKIKLAQTFQVLSTFMFVLMLLSLTYRPLLPSSQDTPSKRG
Cavia porcellus IKMLGDKIKLAQTFQVLSTFMFVLMLLSLTYRPLLPSSQDTPSKRG
Octodon degus IKMLGDKIKLAQTFQVLSTFMFILMLLSLTYRPLLPSSQDTPSKRG
Mus musculus IKMLGDKIKLAQTFQVLSTFMFVLTLLSLTYRPLLPSSQDTPSKRG
Rattus norvegicus IKMLGDRIKLAQTFQVLSTFMFVLTLLSLTYRPLLPSSQDTPSKRG
Ochotona princeps IKMLGDKIKLAQTFQVLSNFMFVLTLLSLTYRPLLPSSQDTPSKRG
Otolemur garnettii IKMLGDKIKLAQTFQVLSTFMFVLMLLSLTYRPLLPSSQDTPSKRG
Macaca mulatta IRMLGDKIKLAQTFQVLSTFMFVLMLLSLTYRPLLPSSQDTPSKRG
Homo sapiens IRMLGDKIKLAQTFQVLSTFMFVLMLLSLTYRPLLPSSQDTPSKRG
Bos taurus IKTLGAKIKLAQTFQVLSTFMFILTLLSLTYRPLLPSSQDTPSKRG
Orcinus orca IKTLGAKIKLAQTFQVLSTFMFILTLLSLTYRPLLPSSQDTPSKRG
330 340 350 360
Identity
Fukomys anselli VRTLHQRFLAQLRKYFNMRVFRQRTYRIWAFGIAAAALGYFVPYVH
Heterocephalus glaber VRTLHQRFLAQLRKYFNMRVFRQRTYRIWAFGIAAAALGYFVPYVH
Cavia porcellus VHTWHQRFLAQLRKYFNMRVFRQRTYRIWAFGIAAAALGYFVPYVH
Octodon degus VHTLHQRFLAQLRKYFNMRVFRQRTYRIWAFGIAAAALGYFVPYVH
Mus musculus AHTLRQRFLVQFRKYFNMRVFRQRTYRIWAFGIAAAALGYFVPYVH
Rattus norvegicus AHTLRQRFLVQFRKYFNMRVFRQRTYRIWAFGIAAAALGYFVPYVH
Ochotona princeps VRTLHQRFLAQLRKYFNMRVFRQRTYRIWAFGIAAAALGYFVPYVH
Otolemur garnettii VRTLHQRFLAQLRKYFNMRVFRQRTYRIWAFGIAAAALGYFVPYVH
Macaca mulatta VRTLHQRFLAQLRKYFNMRVFRQRTYRIWAFGIAAAALGYFVPYVH
Homo sapiens VRTLHQRFLAQLRKYFNMRVFRQRTYRIWAFGIAAAALGYFVPYVH
Bos taurus VHTLCQRFLAQLRKYFNMRVFRQRTYRIWAFGIAAAALGYFVPYVH
Orcinus orca VHTLCQRFLAQLRKYFNMRVFRQRTYRIWAFGIAAAALGYFVPYVH
370 380 390 400 410
Identity
Fukomys anselli LMKYVEEEFLEIKETWVLLVCIGATSGLGRLVSGHISDSIPGLKKI
Heterocephalus glaber LMKYVEEEFLEIKETWVLLVCIGATSGLGRLVSGHISDSIPGLKKI
Cavia porcellus LMKYVEEEFLEIKETWVLLVCIGATSGLGRLVSGHISDSIPGLKKI
Octodon degus LMKYVEEEFLEIKETWVLLVCIGATSGLGRLVSGHISDSIPGLKKI
Mus musculus LMKYVEDKFKEIKETWVLLVCIGATSGLGRLVSGHISDSIPGLKKI
Rattus norvegicus LMKYVEDKFKEIKETWVLLVCIGATSGLGRLVSGHISDSIPGLKKI
Ochotona princeps LMKYVEEEFLEIKETWVLLVCIGATSGLGRLVSGHVSDSIPGLKKI
Otolemur garnettii LMKYVEEEFLEIKETWVLLVCIGATSGLGRLVSGHISDSIPGLKKI
Macaca mulatta LMKYVEEEFSEIKETWVLLVCIGATSGLGRLVSGHISDSIPGLKKI
Homo sapiens LMKYVEEEFSEIKETWVLLVCIGATSGLGRLVSGHISDSIPGLKKI
Bos taurus LMKYVEEEFLEIKQTWVLLVCIGATSGLGRLVSGRVSDSIPGLKKI
Orcinus orca LMKYVEEEFLEIKQTWVLLVCIGATSGLGRLVSGRVSDSIPGLKKI
420 430 440 450 460
Identity
Fukomys anselli YLQVLSFLLLGLMSMMIPLCRDFGGLIVVCLFLGLCDGFFITIMAP
Heterocephalus glaber YLQVLSFLLLGLMSMMIPLCRDFGGLIVVCLFLGLCDGFFITIMAP
Cavia porcellus YLQVLSFLLLGLMSMMIPLCRDFWGLIVVCLFLGLCDGFFITIMAP
Octodon degus YLQVLSFLLLGLMSMMIPLCRNFGGLIVVCLFLGLCDGFFITIMAP
Mus musculus YLQVLSFLLLGLMSMMIPLCRDFGGLIVVCLFLGLCDGFFITIMAP
Rattus norvegicus YLQVLSFLLLGLMSMMIPLCRDFGGLIVVCLFLGLCDGFFITIMAP
Ochotona princeps YLQVLSFLLLGLMSMMIPLCRDFGSLIVVCLFLGLCDGFFITIMAP
Otolemur garnettii YLQVLSFLLLGLMSMMIPLCRDFAGLIVVCLFLGLCDGFFITIMAP
Macaca mulatta YLQVLSFLLLGLMSMMIPLCRDFGGLIVVCLFLGLCDGFFITIMAP
Homo sapiens YLQVLSFLLLGLMSMMIPLCRDFGGLIVVCLFLGLCDGFFITIMAP
Bos taurus YLQVISFLLLGLMSMMIPLCRGFGGLIVVCLFLGLCDGFFITIMAP
Orcinus orca YLQVISFLLLGLMSMMIPLCRGFGGLIVVCLFLGLCDGFFITIMAP

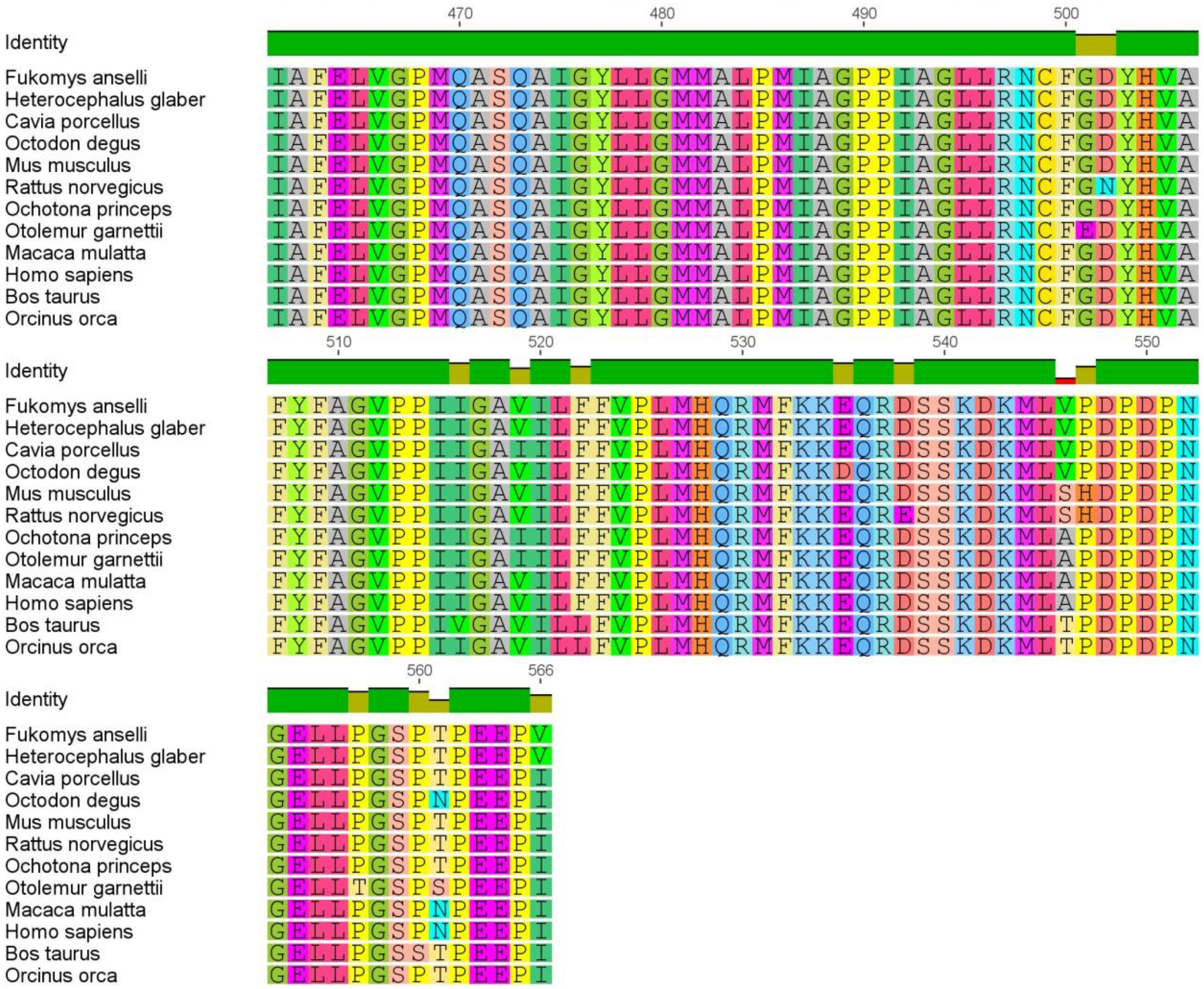

Figure S11. Protein alignment of Monocarboxylate transporter 8 (MCT8) from different mammal species. The mRNA sequence of *F. anselli* was obtained from RNA-seq and subsequently translated, other sequences were retrieved from NCBI databases with the following accession numbers: Heterocephalus glaber (XP_004905154), Cavia porcellus (XP_005004229), Octodon degus (XP_004648070), Mus musculus (AAC40078), Rattus norvegicus (EDM07172), Ochotona princeps (XP_004592817), Otolemur garnettii (XP_003802275), Macaca mulatta (XP_001096017), Homo sapiens (NP_006508), Bos taurus (NP_001193868), Orcinus orca (XP_004283857). Suggestions of NCBI for translation starts of Octodon degus (XP_004648070), Ochotona princeps (XP_004592817), Macaca mulatta (XP_001096017) and Bos taurus (NP_001193868) were changed to the position of the orthologous sequences.
