# Supplementary material for: Unusual Ratio between Free Thyroxine and Free Triiodothyronine in a Long-Lived Mole-Rat Species with Bimodal Ageing
Source: PLoS One. 2014 Nov 19;9(11):e113698. doi: 10.1371/journal.pone.0113698 (PMC4237498; doi:10.1371/journal.pone.0113698)
Supplement: Figure S12 — Protein alignment of Natrium-Iodid-Symporter (NIS) from different mammal species. The mRNA sequence of F. anselli was obtained from RNA-seq and subsequently translated, other sequences were retrieved from NCBI databases with the following accession numbers: Heterocephalus glaber (XP_004873534), Cavia porcellus (XP_003465226), Octodon degus (XP_004646953), Mus musculus (NP_444478), Rattus norvegicus (Q63008), Otolemur garnettii (XP_003796602), Macaca mulatta (EHH29802), Pan troglodytes (XP_524154), Homo sapiens (NP_000444), Canis lupus (XP_541946), Sus scrofa (NP_999575), Orcinus orca (XP_004277608). (PDF) [file pone.0113698.s012.pdf]

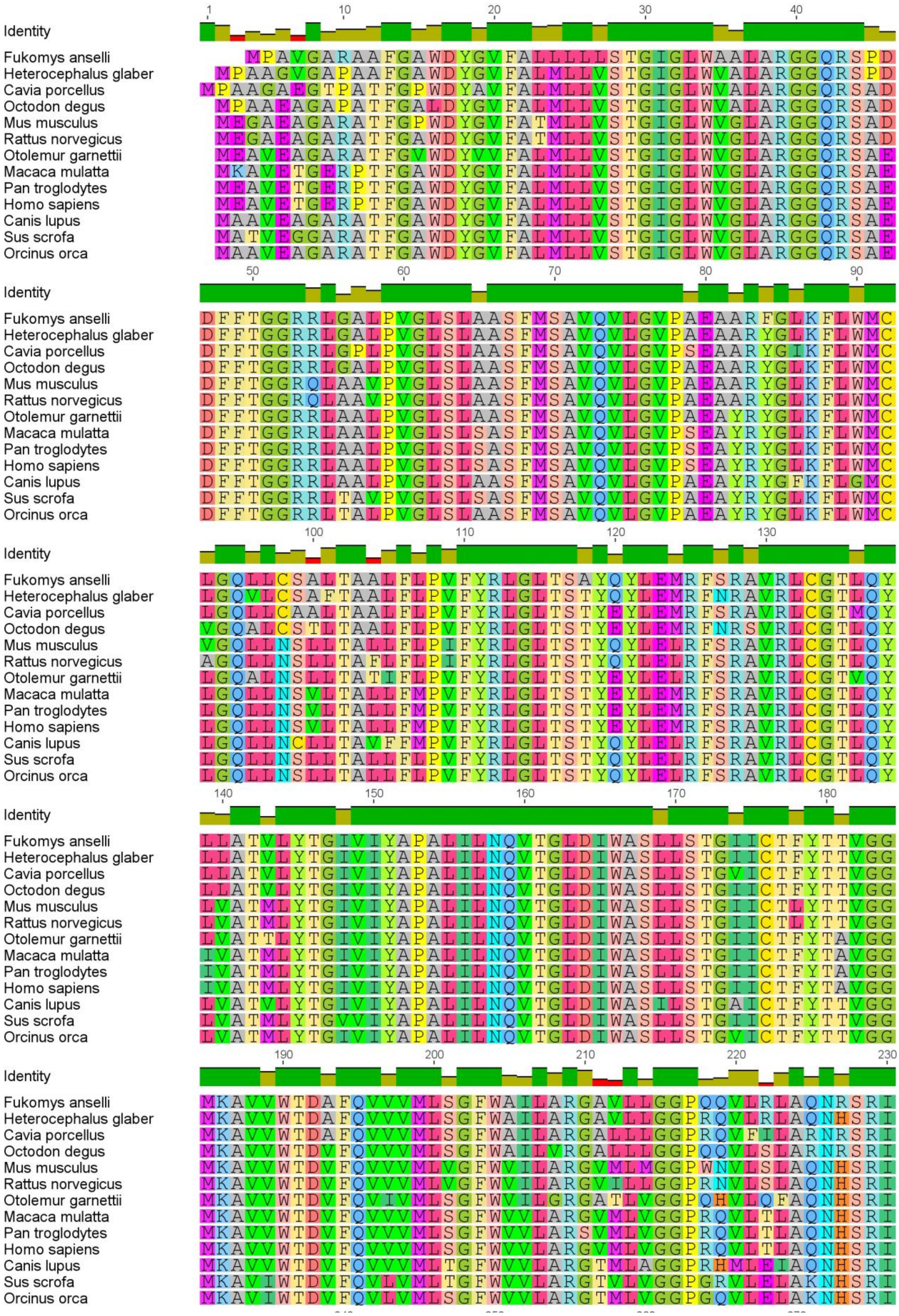
1 10 20 30 40
Identity
Fukomys anselli MPAVGARAAFGAWDYGVFALLLLLSTGIGLWAALARGGQRSPD
Heterocephalus glaber MPAAGVGAPAAFGAWDYGVFALMLLVSTGIGLWVALARGGQRSPD
Cavia porcellus MPAAGAEGTPATFGPWDYAVFALMLLVSTGIGLWVALARGGQRSAD
Octodon degus MPAAEAGAPATFGALDYGVFALMLLVSTGIGLWVALARGGQRSAD
Mus musculus MEGAEAGARATFGPWDYGVFATMLLVSTGIGLWVGLARGGQRSAD
Rattus norvegicus MEGAEAGARATFGAWDYGVFATMLLVSTGIGLWVGLARGGQRSAD
Otolemur garnettii MEAVEAGARATFGVWDYVVFALMLLVSTGIGLWVGLARGGQRSAE
Macaca mulatta MKAVETGERPTFGAWDYGVFALMLLVSTGIGLWVGLARGGQRSAE
Pan troglodytes MEAVETGERPTFGAWDYGVFALMLLVSTGIGLWVGLARGGQRSAE
Homo sapiens MEAVETGERPTFGAWDYGVFALMLLVSTGIGLWVGLARGGQRSAE
Canis lupus MAAVEAGARATFGAWDYGVFALMLLVSTGIGLWVGLARGGQRSAE
Sus scrofa MATVEGGARATFGAWDYGVFALMLLVSTGIGLWVGLARGGQRSAE
Orcinus orca MAAVEAGARATFGAWDYGVFALMLLVSTGIGLWVGLARGGQRSAE
50 60 70 80 90
Identity
Fukomys anselli DFFTGGRRLGALPVGLSLAASFMSAVQVLGVPAEAARFGLKFLWMC
Heterocephalus glaber DFFTGGRRLGALPVGLSLAASFMSAVQVLGVPAEAARYGLKFLWMC
Cavia porcellus DFFTGGRRLGPLPVGLSLAASFMSAVQVLGVPSEAARYGIKFLWMC
Octodon degus DFFTGGRRLGALPVGLSLAASFMSAVQVLGVPAEAARYGLKFLWMC
Mus musculus DFFTGGRQLAAVPVGLSLAASFMSAVQVLGVPAEAARYGLKFLWMC
Rattus norvegicus DFFTGGRQLAAVPVGLSLAASFMSAVQVLGVPAEAARYGLKFLWMC
Otolemur garnettii DFFTGGRRLAALPVGLSLAASFMSAVQVLGVPAEAYRYGLKFLWMC
Macaca mulatta DFFTGGRRLAALPVGLSLSASFMSAVQVLGVPSEAYRYGLKFLWMC
Pan troglodytes DFFTGGRRLAALPVGLSLSASFMSAVQVLGVPSEAYRYGLKFLWMC
Homo sapiens DFFTGGRRLAALPVGLSLSASFMSAVQVLGVPSEAYRYGLKFLWMC
Canis lupus DFFTGGRRLAALPVGLSLAASFMSAVQVLGVPAEAYRYGFKFLGMC
Sus scrofa DFFTGGRRLTAVPVGLSLSASFMSAVQVLGVPAEAYRYGLKFLWMC
Orcinus orca DFFTGGRRLTALPVGLSLAASFMSAVQVLGVPAEAYRYGLKFLWMC
100 110 120 130
Identity
Fukomys anselli LGQLLCSALTAALFLPVFYRLGLTSAYQYLEMRFSRAVRLCGTLQY
Heterocephalus glaber LGQVLCSAFTAALFLPVFYRLGLTSTYQYLEMRFNRAVRLCGTLQY
Cavia porcellus LGQLLCAALTAALFLPVFYRLGLTSTYEYLEMRFSRAVRLCGTMQY
Octodon degus VGQALCSTLTAALFLPVFYRLGLTSTYEYLEMRFNRSVRLCGTLQY
Mus musculus VGQLLNSLLTALLFLPIFYRLGLTSTYQYLELRFSRAVRLCGTLQY
Rattus norvegicus AGQLLNSLLTAFLFLPIFYRLGLTSTYQYLELRFSRAVRLCGTLQY
Otolemur garnettii LGQALNSLLTATIFLPVFYRLGLTSTYEYLELRFSRAVRLCGTVQY
Macaca mulatta LGQLLNSVLTALLFMPVFYRLGLTSTYEYLEMRFSRAVRLCGTLQY
Pan troglodytes LGQLLNSVLTALLFMPVFYRLGLTSTYEYLEMRFSRAVRLCGTLQY
Homo sapiens LGQLLNSVLTALLFMPVFYRLGLTSTYEYLEMRFSRAVRLCGTLQY
Canis lupus LGQLLNCLLTAVFFMPVFYRLGLTSTYQYLELRFSRAVRLCGTLQY
Sus scrofa LGQLLNSLLTALLFLPVFYRLGLTSTYQYLELRFSRAVRLCGTLQY
Orcinus orca LGQLLNSLLTALLFLPVFYRLGLTSTYQYLELRFSRAVRLCGTLQY
140 150 160 170 180
Identity
Fukomys anselli LLATVLYTGIVIYAPALILNQVTGLDIWASLLSTGIICTFYTTVGG
Heterocephalus glaber LLATVLYTGIVIYAPALILNQVTGLDIWASLLSTGIICTFYTTVGG
Cavia porcellus LLATVLYTGIVIYAPALILNQVTGLDIWASLLSTGVICTFYTTVGG
Octodon degus LLATVLYTGIVIYAPALILNQVTGLDIWASLLSTGIICTFYTTVGG
Mus musculus LVATMLYTGIVIYAPALILNQVTGLDIWASLLSTGIICTLYTTVGG
Rattus norvegicus LVATMLYTGIVIYAPALILNQVTGLDIWASLLSTGIICTLYTTVGG
Otolemur garnettii LVATTLYTGIVIYAPALILNQVTGLDIWASLLSTGIICTFYTAVGG
Macaca mulatta IVATMLYTGIVIYAPALILNQVTGLDIWASLLSTGIICTFYTAVGG
Pan troglodytes IVATMLYTGIVIYAPALILNQVTGLDIWASLLSTGIICTFYTAVGG
Homo sapiens IVATMLYTGIVIYAPALILNQVTGLDIWASLLSTGIICTFYTAVGG
Canis lupus LVATVLYTGIVIYAPALILNQVTGLDIWASILSTGAICTFYTTVGG
Sus scrofa LVATMLYTGVVIYAPALILNQVTGLDIWASLLSTGIICTFYTTVGG
Orcinus orca LVATMLYTGIVIYAPALILNQVTGLDIWASLLSTGVICTFYTTVGG
190 200 210 220 230
Identity
Fukomys anselli MKAVVWTDAFQVVVMLSGFWAILARGAVLLGGPQQVLRLAQNRSRI
Heterocephalus glaber MKAVVWTDAFQVVVMLSGFWAILARGAVLLGGPRQVLRLAQNHSRI
Cavia porcellus MKAVVWTDAFQVVVMLSGFWAILARGALLLGGPRQVFILARNRSRI
Octodon degus MKAVVWTDVFQVVVMLSGFWAILVRGALLLGGPQQVLSLARNRSRI
Mus musculus MKAVVWTDVFQVVVMLVGFWVILARGVMLMGGPWNVLSLAQNHSRI
Rattus norvegicus MKAVVWTDVFQVVVMLVGFWVILARGVILLGGPRNVLSLAQNHSRI
Otolemur garnettii MKAVVWTDVFQVIVMLSGFWVILGRGATLVGGPQHVLQFAQNHSRI
Macaca mulatta MKAVVWTDVFQVVVMLSGFWVVLARGVMLVGGPRQVLTLAQNHSRI
Pan troglodytes MKAVVWTDVFQVVVMLSGFWVVLARSVMLVGGPRQVLTLAQNHSRI
Homo sapiens MKAVVWTDVFQVVVMLSGFWVVLARGVMLVGGPRQVLTLAQNHSRI
Canis lupus MKAVVWTDVFQVVVMLTGFWVVLARGTMLAGGPRHMLEIAQNHSRI
Sus scrofa MKAVIWTDVFQVLVMLTGFWVVLARGTVLVGGPGRVLELAKNHSRI
Orcinus orca MKAVIWTDVFQVLVMLSGFWVVLARGTMLVGGPGQVLELAKNHSRI

```
                         240       250       260       270
Identity
Fukomys anselli          NLLDFDPDPRSRYTFWTFVVGGTLVWLSMYGVNQAQVQRYVSCSTE
Heterocephalus glaber    NLLDFDPDPRSRYTFWTFVVGGTLVWLSMYGVNQAQVQRYVACSTE
Cavia porcellus          NLLDFDLDPRSRYTFWTFVVGGTLVWLSMYGVNQAQVQRYVACSTE
Octodon degus            NLLDFDPDPRSRYTFWTFVVGGTLVWLSMYGVNQAQVQRYIACSTE
Mus musculus             NLMDFDPDPRSRYTFWTFVVGGSLVWLSMYGVNQAQVQRYVACHTE
Rattus norvegicus        NLMDFDPDPRSRYTFWTFIVGGTLVWLSMYGVNQAQVQRYVACHTE
Otolemur garnettii       NLMDFDPDPRRRYTFWTFVVGGTLVWLSMYGVNQAQVQRYVACRTE
Macaca mulatta           NMMDFNPDPRSRYTFWTFMVGGTLVWLSMYGVNQAQVQRYVACRTE
Pan troglodytes          NLMDFNPDPRSRYTFWTFVVGGTLVWLSMYGVNQAQVQRYVACRTE
Homo sapiens             NLMDFNPDPRSRYTFWTFVVGGTLVWLSMYGVNQAQVQRYVACRTE
Canis lupus              NLMDFDLDPRSRYTFWTFVVGGTLVWLSMYGVNQAQVQRYVACRTE
Sus scrofa               NLMDFDLDPRRRYTFWTFVVGGTLVWLSMYGVNQAQVQRYVACRTE
Orcinus orca             NLMDFDLDPRSRYTFWTFVVGGTLVWLSMYGVNQAQVQRYMACRTE
```

```
                           280       290       300       310       320
Identity
Fukomys anselli          RKAKLAVLINQLGLFLIVTSAACCGIVMFVFYSDCDPLLTGRISAP
Heterocephalus glaber    RKAKLAVLINQLGLLVIVTSAACCGILMFVFYSDCDPLLTGRISAP
Cavia porcellus          RKAKLAILINQLGLFLIVGSAACCGVIMFVFYSDCDPLLTGRISAP
Octodon degus            RKAKLAVLINQLGLFLIVISAACCGIVMFVFYSDCDPLLTGRISAP
Mus musculus             RKAKLALLVNQLGLFLIVASAACCGIVMFVYYKDCDPLLTGRIAAP
Rattus norvegicus        GKAKLALLVNQLGLFLIVASAACCGIVMFVYYKDCDPLLTGRISAP
Otolemur garnettii       GQAKLALLINQMGLFLIVSSAAVCGIVMFVFYSDCDPLLTGRISAP
Macaca mulatta           KQAKLALLINQVGLFLIVSSAACCGIVMFVFYTDCDPLLLGRISAP
Pan troglodytes          KQAKLALLINQVGLFLIVSSAACCGIVMFVFYSDCDPLLLGRISAP
Homo sapiens             KQAKLALLINQVGLFLIVSSAACCGIVMFVFYTDCDPLLLGRISAP
Canis lupus              KQAKLALLINQLGLFLIVLSAAGCGIIMFAIYIDCDPLLAGRISAP
Sus scrofa               KQAKLALLINQVGLFLIVSSAAACGIVMFALYVDCDPLLAGHISAP
Orcinus orca             KQAKLAVLVNQLGLFLIVSSAAFCGIIMFTFYLDCDPLLAGRISAP
```

```
                               330       340       350       360
Identity
Fukomys anselli          DQYMPLFVLDIFEDLPGVPGLFLACAYSGTLSTASTSINAMAAVTV
Heterocephalus glaber    DQYMPLFVLDIFEDLPGVPGLFLACAYSGTLSTASTSINAMAAVTL
Cavia porcellus          DQYMPLFVLDIFEDLPGVPGLFLACAYSGTLSTASTSINAMAAVTV
Octodon degus            DQYMPLFVLDIFEDLPGVPGLFLACAYSGTLSTASTSINAMAAVTV
Mus musculus             DQYMPLLVLDIFEDLPGVPGLFLACAYSGTLSTASTSINAMAAVTV
Rattus norvegicus        DQYMPLLVLDIFEDLPGVPGLFLACAYSGTLSTASTSINAMAAVTV
Otolemur garnettii       DEYMPLLVLDIFDDLPGVPGLFLACAYSGTLSTASTSINAMAAVTV
Macaca mulatta           DQYMPLLVLDIFEDLPGVPGLFLACAYSGTLSTASTSINAMAAVTV
Pan troglodytes          DQYMPLLVLDIFEDLPGVPGLFLACAYSGTLSTASTSINAMAAVTV
Homo sapiens             DQYMPLLVLDIFEDLPGVPGLFLACAYSGTLSTASTSINAMAAVTV
Canis lupus              DQYMPLLVLDIFEDLPGVPGLFLACAYSGTLSTASTSINAMAAVTV
Sus scrofa               DQYMPLLVLDIFEDLPGVPGLFLACAYSGTLSTASTSINAMAAVTV
Orcinus orca             DQYMPLMVLDIFVDLPGVPGLFLACAYSGTLSTASTSINAMAAVTV
```

```
                         370       380       390       400       410
Identity
Fukomys anselli          EDLIKPRLTGLAPRRLVLISKGLSLLYGSACLAVAALSSLLGGGVL
Heterocephalus glaber    EDLIKPLLPGLAPRRLVLVSKGLSLVFGSACLAVAALSSLLGGGVL
Cavia porcellus          EDLVKPCRPGLSPRRLVLVSKGLSLLYGSACLSVAALSSLLGGGVL
Octodon degus            EDLIRPRLPGLAPRRLMLISKGLSLVYGSACLTVAALSSLLGGGVL
Mus musculus             EDLIKPRMPSLAPRKLVFISKGLSFIYGSTCLTVAALSSLLGGGVL
Rattus norvegicus        EDLIKPRMPGLAPRKLVFISKGLSFIYGSACLTVAALSSLLGGGVL
Otolemur garnettii       EDLIKPRLPNLAPRKLVIISKGLSIIYGSACLTVAALSSLLGGGVL
Macaca mulatta           EDLIKPRLQNLAPRKLVIISKGLSLIYGSACLTVAALSSLLGGGVL
Pan troglodytes          EDLIKPRLRSLAPRKLVIISKGLSLIYGSACLTVAALSSLLGGGVL
Homo sapiens             EDLIKPRLRSLAPRKLVIISKGLSLIYGSACLTVAALSSLLGGGVL
Canis lupus              EDLIKPRLPSLAPRRLVLISKGLSLIYGSACLTVAALSSLLGGGVL
Sus scrofa               EDLIKPRLPNLAPRRLVIISKGLSLIYGSACLTVAALSSLLGGGVL
Orcinus orca             EDLIKPRLPSLTPRRLVIISKGLSLIYGSACLTVAALSSLLGGGVL
```

```
                              420       430       440       450       460
Identity
Fukomys anselli          QGSFTVMGVIGGPLLGAFALGMLLPASNAPGVLSGLGVGFLLSLWV
Heterocephalus glaber    QGSFTVMGVISGPLLGAFALGMFLPACNAPGVLSGLGAGFSISLWV
Cavia porcellus          QGSFTVMGVISGPLLGAFALGMFLPACNTTGVLFGLGSGFLLSLWV
Octodon degus            QGSFTVMGVISGPLLGAFALGMFLPACNAPGVLFGLGSGFLISLWV
Mus musculus             QGSFTVMGVISGPLLGAFTLGMLLPACNTPGVLSGLTAGLAVSLWV
Rattus norvegicus        QGSFTVMGVISGPLLGAFTLGMLLPACNTPGVLSGLAAGLAVSLWV
Otolemur garnettii       QGSFTVMGVISGPLLGAFVLGMFMPACNTPGVLSGLGAGVALSLWV
Macaca mulatta           QGSFTVMGVISGPLLGAFVLGMFLPACNTPXXXXXXXXXXXXSLWV
Pan troglodytes          QGSFTVMGVISGPLLGAFILGMFLPACNTPGVLAGLGAGLALSLWV
Homo sapiens             QGSFTVMGVISGPLLGAFILGMFLPACNTPGVLAGLGAGLALSLWV
Canis lupus              QGSFTVMGVISGPLLGAFILGMFLPACNIPGVLSGLASGLVLSLWV
Sus scrofa               QGSFTVMGVISGPLLGAFVLGMFLPSCNTSGVLSGLAAGLALSLWV
Orcinus orca             EGSFTIMGVISGPLLGAFILGMFLPACNTLGVLSGLAVGLALSLWV
```

```
                                 470       480       490       500
Identity
Fukomys anselli           ALGATLYPPSEQTMGVLPSSATGCMVSFSNASVLY-SLATIN--NS
Heterocephalus glaber     ALGATLYPPSEQTMGVLPSSATACVVSSSNASVLLDSLPTVNASAS
Cavia porcellus           ALGATLYPPSEQIMGVLPSSAAGCLVPSSNTSVLLDALPTLN--GS
Octodon degus             ALGATLYPPSEQTMGVLTSSAAGCVVPSSNASVLLDPLPTLN--IS
Mus musculus              AVGATLYPPGEQTMGVLPTSAAGCTNASVLPSPPG-----------
Rattus norvegicus         AVGATLYPPGEQTMGVLPTSAAGCTNDSVLLGPPG-----------
Otolemur garnettii        ALGATLYPPSAQTMGVLPSSAVRCVAPSANASGLLDPL--------
Macaca mulatta            ALGATLYPPSEQTMRVLPSSAARCLALSVNASGLLDMA--------
Pan troglodytes           ALGATLYPPSEQTMRVLPSSAARCVALSVNASGLLDPA--------
Homo sapiens              ALGATLYPPSEQTMRVLPSSAARCVALSVNASGLLDPA--------
Canis lupus               AVGATLYPPSNQSLGVLPSSSAGCAVPSANASDLMGL---------
Sus scrofa                AVGASLYPPSAQSMGVLPSSAAGCALPTANASGLQDP---------
Orcinus orca              AVGATLYPPSAQSMGVLPSSAAGCAVPSANASGILDP---------

                             510       520       530       540       550
Identity
Fukomys anselli           GLPSAGVTGSGSSSAEAPGRPALVHSFYAISYLYYGALGTLTTVLC
Heterocephalus glaber     SLPSVGVTGSGSSSAGDPGRPALAHSFYAISYLYYGALGTLTTVLC
Cavia porcellus           SLLNAEMTGFGSSSAEDPGRPTLAHSFYAISYLYYGALGTLTTMLC
Octodon degus             RXATS------SSSAEDLGRPALADSFYSISYLYYGALGTLITMLL
Mus musculus              ---AANTSRGIPSSGMDSGRPAFADTFYAVSYLYYGALGTLTTMLC
Rattus norvegicus         ---ATNASNGIPSSGMDTGRPALADTFYAISYLYYGALGTLTTMLC
Otolemur garnettii        -LLAANASNRSPSSRMDLSQPALAESFYAISYLYYGALGTLTTLLC
Macaca mulatta            -LLPANDSSRAPSSGMDASRPALADSFYAISYLYYGALGTLTTVLC
Pan troglodytes           -LLPANDSSRAPSSGMDDSRPALADSFYAISYLYYGALGTLTTVLC
Homo sapiens              -LLPANDSSRAPSSGMDASRPALADSFYAISYLYYGALGTLTTVLC
Canis lupus               -LLANNTSSRNPSPGVDLGRPALADSFYAISYLYYGALGTMSTVLC
Sus scrofa                -VLAVNASSTASSLETDPEQPILAASFYAISYLYYGALGTLSTILC
Orcinus orca              -ILAANASSRASSLEMDPDQPTLTDSFYAISYLYYGALGTVSTVLC

                                 560       570       580       590
Identity
Fukomys anselli           GALVSYLTGPTKRSSLGPGLLWWDLTRQTASVAPTEDVATLDDGLV
Heterocephalus glaber     GALVSYLTGPTKRSSLGPGLLWWDLTRKTASVAPKEDVATLGDGLV
Cavia porcellus           GALVSYLTGPTTRSMLGPGLLWWDLTRQTASVAPKEEVATLNDGL-
Octodon degus             GALVSYLTGPTKRSALGPGLLWWDLERQTASVAPREEVGTVDDGLV
Mus musculus              GALISYLTGPTKRSSLGPGLLWWDLARQTASVAPKEDTTTLEDSLV
Rattus norvegicus         GALISYLTGPTKRSSLGPGLLWWDLARQTASVAPKEDTATLEESLV
Otolemur garnettii        GALVSCLTGPTKRSDLGPGLLWWDLVRQTASVAPKEEVAVLDDSLV
Macaca mulatta            GALISCLTGPTKRSTLAPGLLWWDLARQTASVAPKEEVAILDDNLV
Pan troglodytes           GALISCLTGPTKRSTLAPGLLWWDLARQTASVAPKEEVAILDDNLV
Homo sapiens              GALISCLTGPTKRSTLAPGLLWWDLARQTASVAPKEEVAILDDNLV
Canis lupus               GALVSCLTGPTKRSALGPGLLWWDLARQTASVAPKEEVATLDDSLG
Sus scrofa                GALISCLTGPTKRSALGPGLLWWDLTRQTASVAPKEEVAALDDSLM
Orcinus orca              GALVSCLTGPTKRSALGPGLLWWDLTRQTASVAPKEEVATLDDSLM

                            600       610       620       630       640
Identity
Fukomys anselli           K-GLEEIPSGAKSAPT---FRPAAEDLPLFL-QCEAKGRGSWGPSS
Heterocephalus glaber     K-GLEEIPVGAKSAPDSRDFLPTEENRPLFL-QHEARGPGPWAPSG
Cavia porcellus           --SPEEIPAEAKSACD---FRPTEDNCPLFL-RLEGKGTGPRAPCS
Octodon degus             K-SPGEITAGAKSAPD---FQPIEEDHQLFL-QSQEKGSGRWAPSS
Mus musculus              K-GPEDIPAATKKPPG---FRPEAETHPLYLGHDAETNL
Rattus norvegicus         K-GPEDIPAVTKKPPG---LKPGAETHPLYLGHDVETNL
Otolemur garnettii        K-GPEELPPGTKKPPG---FLSTNEDHVVFLGQKEMAGVSSWTPHS
Macaca mulatta            K-GPEELPAGTKKAPG---FLPTNEDRLFFLGQKELEGAGSWTPCG
Pan troglodytes           K-GPEELPAGNKKPPG---FLPTNEDRLFFLGQKELEGAGSWTPCV
Homo sapiens              K-GPEELPTGNKKPPG---FLPTNEDRLFFLGQKELEGAGSWTPCV
Canis lupus               K-GAEELPPGTKRPPD---FLPNDEDHLLFLGQKEVNGAGSWTPRS
Sus scrofa                KQGAEELPLAIKKPPD---FLSTNEDHLLFLGQKEVNGASSKTPGS
Orcinus orca              K-GAEELPPGTKRRPD---FLPTDEDHLLFLGQKEMNGAGSRTPGS

                                  650    657
Identity
Fukomys anselli           CRDQDPRDHETN
Heterocephalus glaber     GKGKGEQDLETNL
Cavia porcellus           GKGWV-LDYETNL
Octodon degus             GKGRD-LDRETNL
Mus musculus
Rattus norvegicus
Otolemur garnettii        GSDGN-DPQETNL
Macaca mulatta            RHDGGRDQRETNL
Pan troglodytes           GHDGGRDQQETNL
Homo sapiens              GHDGGRDQQETNL
Canis lupus               ARDHGQDLRETDL
Sus scrofa                EHDKGHDLRETDL
Orcinus orca              EHDKGLNLRETDL
```

Figure S12. Protein alignment of Natrium-Iodid-Symporter (NIS) from different mammal species.
The mRNA sequence of *F. anselli* was obtained from RNA-seq and subsequently translated, other sequences were retrieved from NCBI databases with the following accession numbers: Heterocephalus glaber (XP_004873534), Cavia porcellus (XP_003465226), Octodon degus (XP_004646953), Mus musculus (NP_444478), Rattus norvegicus (Q63008), Otolemur garnettii (XP_003796602), Macaca mulatta (EHH29802), Pan troglodytes (XP_524154), Homo sapiens (NP_000444), Canis lupus (XP_541946), Sus scrofa (NP_999575), Orcinus orca (XP_004277608).
