# Supplementary material for: Unusual Ratio between Free Thyroxine and Free Triiodothyronine in a Long-Lived Mole-Rat Species with Bimodal Ageing
Source: PLoS One. 2014 Nov 19;9(11):e113698. doi: 10.1371/journal.pone.0113698 (PMC4237498; doi:10.1371/journal.pone.0113698)
Supplement: Table S1 — Intra- and inter-assay variances of the Enzyme Immunoassays for fT3, fT4, tT3 and tT4. Shown are the coefficients of variances (%CV) of the assays used in the present study, according to the manufacturer (DRG Instruments GmbH). (PDF) [file pone.0113698.s013.pdf]

Table S1. Intra- and inter-assay variances of the Enzyme Immunoassays for fT3, fT4, tT3 and tT4: Shown are the coefficients of variances (%CV) of the assays used in the present study, according to the manufacturer (DRG Instruments GmbH).

		**Intra-assay variance (in %CV)**			**Inter-assay variance (in %CV)**		
	**Model**	low	medium	high	low	medium	high
**FT3**	**EIA-2385**	4.9%	3.6%	3.1%	13.1%	7.9%	10.2%
**FT4**	**EIA-2386**	10.98%	4.26%	3.25%	10.81%	6.01%	7.90%
**TT3**	**EIA-4569**	6.61%	6.54%	3.59%	6.37%	5.23%	6.73%
**TT4**	**EIA-4568**	3.4%	2.5%	5.6%	4.9%	5.4%	8.1%
